# Smiles‐Truce Cascades Enable Heteroaryl Cyclopropane and Sultine Synthesis

**DOI:** 10.1002/anie.202512577

**Published:** 2025-07-31

**Authors:** Thomas Sephton, Zi Liu, Michael F. Greaney

**Affiliations:** ^1^ School of Chemistry University of Manchester Manchester M13 9PL UK

**Keywords:** Cascade reactions, Cyclopropanes, Heteroarylation, Smiles rearrangement, Sultines

## Abstract

We describe a triple C─C bond cascade process for heteroaryl cyclopropane synthesis, through the reaction of vinyl sulfonium salts and sulfones under mild conditions. An initial conjugate addition (C─C bond 1) sets up a Smiles–Truce rearrangement (C─C bond 2), with a final 3‐exo‐*tet* ring closure (C─C bond 3) affording cyclopropanes. Serendipitously, we uncovered a novel reaction variant that affords sultines, an under‐explored heterocycle with potential application as a bioisostere in medicinal chemistry.

## Graphical abstract

A triple C─C cascade approach to the synthesis of heteroaryl cyclopropanes is described. The reaction is metal‐free, uses simple starting materials, and presents a new retrosynthetic approach for heteroaryl cyclopropane synthesis. A change of substituent creates a new route to sultines, under‐explored motifs in medicinal chemistry.

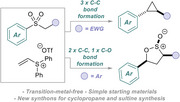

Cascade reactions that enable simultaneous assembly of several bonds in a single operation have long been at the forefront of organic chemistry. The incorporation of these sequences into synthetic routes can significantly expedite the construction of complex molecules, such as pharmaceuticals and natural products,^[^ ^1^ ^]^ and remains an area of substantial interest. An important class of cascade reactions is that involving Smiles–Truce rearrangements (STR) (Figure 1a)^[^ ^2^ ^]^ as they provide versatile ways to install arene and heteroarene rings. An initial intermolecular C─X or C─C bond formation is used to set up an in situ arylation event, via intramolecular S_N_Ar reaction characteristic of the classic Smiles rearrangement. Recent developments have established wide scope of reactivity, with common feedstocks such as alkenes, alkynes, carbonyls, and alkyl halides undergoing tandem reaction with a variety of arene donors, via both polar and radical mechanisms.^[^ ^3^, ^4^, ^5^, ^6^, ^7^, ^8^, ^9^, ^10^, ^11^, ^12^, ^13^, ^14^, ^15^, ^16^, ^17^, ^18^, ^19^, ^20^, ^21^, ^22^, ^23^, ^24^, ^25^, ^26^, ^27^ ^]^ The manifold importance of arenes in functional molecules then gives these methods a vast breadth of application.

**Figure 1:**
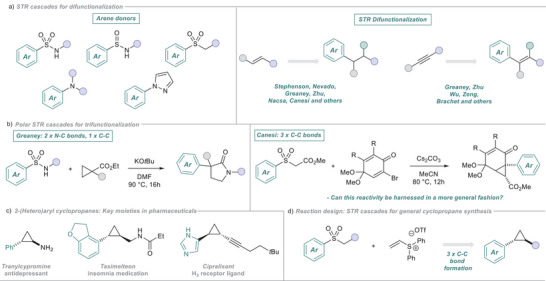
a) STR cascades used for difunctionalization. b) Polar STR cascades used for trifunctionalization. c) Cyclopropanes in medicinal chemistry. d) Our proposal.

STR cascade chemistry generally forges two new bonds, with approaches that forge three being much rarer. Such strategies are inherently more valuable as they can accelerate synthetic routes, minimizing distinct steps and maximizing efficiency and economy. In the radical regime, the Nevado lab has pioneered cascade additions to the N‐sulfonyl ‐acrylamide system, forming densely functionalized heterocycles through triple C─C bond formation.^[^ ^28^ ^]^ Within the field of polar STRs, our lab has developed a pyrrolidinone synthesis through a cyclopropane ring opening sequence,^[^ ^29^ ^]^ and the Canesi lab described a cyclopropane synthesis from sulfones and a quinone derivative (Figure 1b).^[^ ^30^ ^]^ This latter example features an impressive cascade of three sequential C─C bond formations but is restricted to a single, bespoke scaffold.

Given that the C─C bond is intrinsically the most important junction in organic chemistry, the development of a general STR cascade that simultaneously forges three C─C bonds would be a powerful advance.

Our group's longstanding interest in STR cascades^[^ ^31^, ^32^, ^33^ ^]^ led us to investigate STR tri‐functionalization as a route for cyclopropane synthesis. Cyclopropanes are the 6th most common ring system in small‐molecule drugs, heralded for their ability to improve conformational rigidity and metabolic stability within lead optimization campaigns (Figure 1c).^[^ ^34^ ^]^ To realize our desired cyclopropanation, we planned to use heteroaryl sulfones as arylating reagents. While not as common as benchmark sulfonamides in polar STR arylation, we were encouraged by several reports that highlight their ability to act as nucleophiles in tandem STR systems.^[^ ^10^, ^35^, ^36^ ^]^ We next identified diphenyl(vinyl)sulfonium triflate **2** as a reaction partner, inspired by reports by Aggarwal detailing its use in tandem chemistry.^[^ ^37^ ^]^ We proposed that the simple combination of these reagents and a base would kickstart our STR cascade, affording the desired heteroarylated cyclopropanes (Figure 1d).

Cognizant of the fact that many polar STR systems are limited to nitroarene migration, we instead looked to similarly electron‐deficient, medicinally important pyrimidines as our model migrating ring, choosing **1a** as our model substrate. Gratifyingly, we observed a large amount of product formation (**3a**) using K_2_CO_3_ as a base in DMF (Table 1, entry 1). Importantly, the reaction proved to be diastereoselective, with only the *trans*‐isomer observed. Cooling the reaction to −40 °C enhanced efficiency (entry 2), although elongated reaction time did not yield any further improvement (entries 3 and 4). Substitution of the sulfonium reaction partner with Ritter's vinyl thianthrenium salt^[^ ^38^ ^]^ gave only low yields of the product (entry 5). Finally, we developed alternative conditions using DMSO as solvent, which led to complete starting material consumption in one hour, at the expense of somewhat lowered yield (entry 6).

**Table 1:** Selected reaction optimization

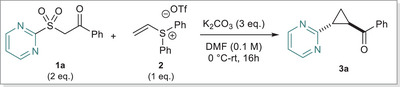		
Entry^a)^	Deviation from standard conditions	Yield (%)
1	None	50
**2**	**−40 °C to rt**	**64 (63)**
3	−40 °C to rt, 48 h	64
4	−40 °C to rt, 96 h	65
5	−40 °C to rt, Vinyl TT instead of **2**	24
6	DMSO as solvent, 50 °C, 1 h	47

^a)^ 0.1 mmol scale, NMR yields using 1,3,5‐trimethoxybenzene as internal standard, isolated yields in parentheses. Vinyl TT = Vinyl thianthrenium tetrafluoroborate.

Taking the cold reaction conditions forward, we next looked to examine the substrate scope of the transformation (Figure 2a). Using pyrimidine as our model heteroarene, we first found that the synthesis of model substrate **3a** can be effectively scaled. The reaction conditions tolerated functionalized acetophenones, furnishing halogenated products **3b** and **3c** in good yields. Electron‐rich (**3d** and **3e**) and deficient (**3f**) arenes both performed well, and we could incorporate a thiophene (**3g**) heterocycle with no significant issues. After sufficient exploration of functionalized arenes, we next looked to use our protocol to synthesize some trisubstituted cyclopropanes. Gratifyingly, introducing a methyl group to the sulfone starting materials afforded **3h** and **3i**, challenging to make through existing methods. We could successfully extend this idea to synthesize more challenging cyclopropanes such as the tetralone derivative **3j**, creating a new disconnection approach for complex cyclopropane synthesis (Figure 2b). The ethyl malonate derivative **3k** likewise proceeded in an efficient manner. However, we found that amides, carboxylic acids and nitriles were not effective electron‐withdrawing groups on the sulfone component under the reaction conditions (see Supporting Information for a list of unsuccessful substrates).

**Figure 2:**
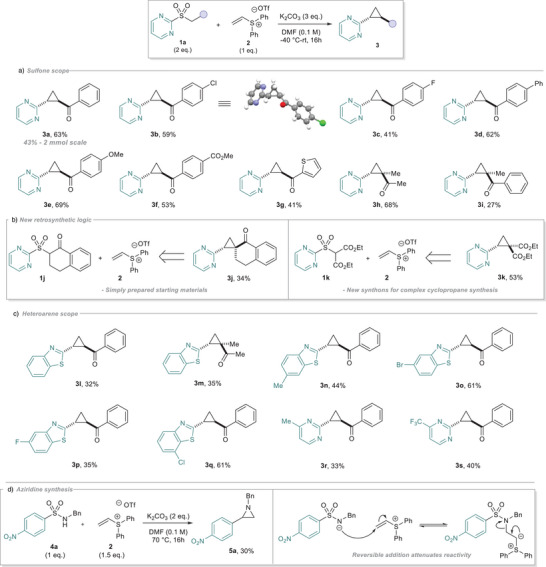
a) Substrate scope of sulfone component. b) New retrosynthetic logic for complex cyclopropane synthesis. c) Substrate scope of the heteroarene. d) Expansion to aziridine synthesis. Reactions performed on a 0.2 mmol scale, isolated yields. All reactions produced the trans isomer in a > 20:1 ratio.

Consecutively, we looked at exploring the heteroarene scope (Figure 2c). Benzothiazole was effectively migrated, producing **3l** in moderate yield. We could also synthesize tri‐substituted benzothiazole cyclopropane **3m** in reasonable efficiency. Substituted benzothiazoles were also effective migrating rings, with substitution tolerated across the ring, producing **3n**‐**3q**. **3o** is of particular note, as the effective inclusion of an aryl bromide provides an excellent functional handle for further derivatization. Furthermore, substituted pyrimidines were also tolerated, furnishing **3r** and notably **3s**, which features a medicinally important trifluoromethyl moiety. Further extensions to substituted pyridines and pyridine N‐oxides were not successful, with migrations occurring in very low yields.

Having established the cyclopropanation sequence, we then turned to sulfonamide **4a** to investigate a possible aziridination reaction (Figure 2d). We could successfully make **5a** in low yield when heated in DMF, but further optimization attempts were not productive. Electron‐poor sulfonamides (pKa of nosylamide = 9.1) are poor nucleophiles for intermolecular aza‐Michael type additions, rendering the subsequent STR step difficult.^[^ ^39^, ^40^ ^]^ Indeed, polar STR cascades between sulfonamides and alkenes are rare in the literature,^[^ ^36^, ^41^, ^42^ ^]^ and restricted to intramolecular examples where the sulfonamide is tethered to the alkene. Furthermore, aziridines are good alkylating agents, and we observed **5a** to be unstable in our reaction conditions.

During the course of our substrate scope exploration, we wondered if a nitroarene would be a viable electron‐withdrawing group on the sulfone as it would create valuable diaryl cyclopropane products. When we subjected substrate **6a** to our reaction conditions, we did not observe any cyclopropane product, but instead γ‐sultine **7a** was formed in good yield (Figure 3a, structure confirmed by X‐Ray crystallography). Sultines hold untapped potential as chiral bioisosteres of lactams and lactones, but their use in medicinal chemistry is limited, partially due to the difficulty of their preparation.^[^ ^43^, ^44^ ^]^ Excited by this result, we prepared a small selection of differentially substituted sulfones **6** and were pleased to observe successful cascade bond formation for sultine synthesis (**7b**‐**7d)**, using nitro‐ and cyano‐arenes to acidify the benzylic protons (Figure 3b).

**Figure 3:**
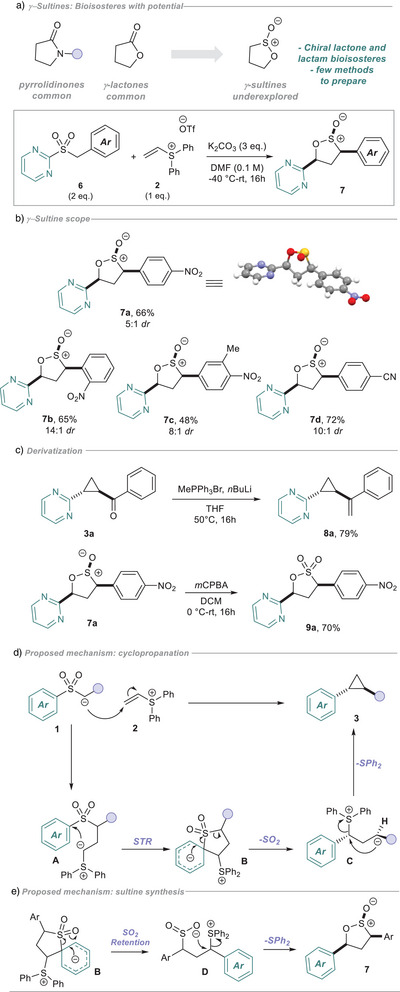
a) Sultines: Underexplored bioisosteres. b) Sultine scope. Minor diastereoisomer unassigned. c) Derivatization. d) Proposed mechanism for the cyclopropanation. e) Proposed mechanism for the sultine synthesis.

To demonstrate the synthetic utility of this method, we next looked to perform functional group interconversions on our products (Figure 3c). Under Wittig reaction conditions, cyclopropane **3a** could be methylenated to vinylcyclopropane **8a**, useful as a radical probe and as a starting motif for transition‐metal‐catalyzed annulations. Simple oxidation of sultine product **7a** with *m*CPBA gave the sultone **9a** in 70% yield.

Turning to the mechanism of our transformation (Figure 3d), we propose that Michael addition occurs after deprotonation of **1**, forming sulfur ylide **A**. Importantly, this addition is significantly less reversible than the aforementioned sulfonamide conjugate addition, owing to the attenuated acidity of the sulfone α‐protons. This can then undergo a 5‐membered STR, through Meisenheimer intermediate **B**. For the cyclopropanation, **B** then collapses, releasing SO_2_ and forming enolate intermediate **C**. This anion can then perform an intramolecular 3‐*exo*‐tet S_N_2, eliminating diphenylsulfide and forming cyclopropane **3** diastereoselectively through the sterically‐preferred *anti* conformation **C**. For the sultine synthesis, we propose that SO_2_ is retained after collapse of **B**, forming intermediate **D** (Figure 3e). Although much rarer than SO_2_ extrusion, recent reports within the radical regime have described post STR SO_2_ retention for sulfones lacking stabilizing groups.^[^ ^45^, ^46^ ^]^ Here, the aryl substituent in **D** is less capable of stabilizing the anion arising from desulfonylation (*cf* **C** in Figure 3d), promoting direct substitution to give sultine **7** with displacement of diphenylsulfide.

In conclusion, we have developed a metal‐free, stereoselective synthesis of medicinally relevant heteroaryl cyclopropanes. The procedure utilizes simple starting materials and provides a new retrosynthetic approach for cyclopropane synthesis. The transformation forges three C─C bonds simultaneously, a feature exceptionally rare in polar STR cascades. Furthermore, we can engage various important heterocycles in our protocol and also provide an example of a novel, analogous aziridine synthesis. Finally, we can leverage related reactivity to synthesize γ‐sultines, promising lactam and lactone bioisosteres for drug design.

## Supporting Information

The authors have cited additional references within the Supporting Information.^[^ ^30^, ^31^ ^]^ Synthetic methods, ^1^H NMR, ^13^C NMR, ^19^F NMR spectra, HRMS, melting points, and crystallographic data are available in the Supporting Information.^[^ ^47^ ^]^

## Conflict of Interests

The authors declare no conflict of interest.

## Supporting information



Supporting Information

Supporting Information

## Data Availability

The data that support the findings of this study are available in the Supporting Information of this article.
