# Supplementary material for: Smiles‐Truce Cascades Enable Heteroaryl Cyclopropane and Sultine Synthesis
Source: Angew Chem Int Ed Engl. 2025 Jul 31;64(38):e202512577. doi: 10.1002/anie.202512577 (PMC12435426; doi:10.1002/anie.202512577)
Supplement: Supplementary file 1 — Supporting Information [file ANIE-64-e202512577-s001.pdf]

Smiles-Truce Cascades Enable Heteroaryl Cyclopropane and Sultine Synthesis

Thomas Sephton, Zi Liu, and Michael F. Greaney*

Department of Chemistry, University of Manchester, Oxford Rd, Manchester M13 9PL, United Kingdom

Supporting Information

Table of Contents

1. General methods S2
2. General procedures S3
3. Troublesome and unsuccessful substrates S4
4. Compound characterization S5
5. References S23
6. X-ray crystallography S24
7. NMR spectra S28

1. General methods

Nuclear magnetic resonance spectra were acquired on either 500 MHz (Bruker® AVII+ 500, Bruker AVIII HD 500) or 400 MHz (Bruker AVIII HD 400, Bruker AVIII 400). All ^{1}H NMR spectra are reported in parts per million (ppm) and were measured relative to the signals at 7.26 ppm ($CHCl_3$), 2.50 ppm (DMSO). All ^{13}C NMR spectra were reported in ppm relative to residual $CHCl_3$ (77.16 ppm), DMSO (39.52 ppm) and were obtained with 1H-decoupling. All ^{19}F chemical shifts were unadjusted from raw data. Data for ^{1}H NMR are described as following: chemical shift (δ in ppm), multiplicity (s, singlet; d, doublet; t, triplet; q, quartet; quin, quintet; m, multiplet; br, broad signal), coupling constant (Hz), integration. Diastereomers were assigned with the aid of H-H-NOE spectroscopy.

High resolution mass spectra were obtained on ThermoFisher Scientific Q-ExactiveTM, Thermo Scientific Exactive plus EMR and Agilent 6530 Q-TOF instruments, using either electrospray ionisation (ESI), atmospheric-pressure chemical ionisation (APCI) as ionisation methods in the positive and negative mode.

Flash column chromatography was carried out by using re-used 10g, 25g or 50g Biotage® Snap Ultra or Biotage Sfär Silica cartridges on a Biotage Isolera Four automated column, using 35-70 µm, 60 Å silica gel for chromatography from ThermoFisher Scientific® or 40-63 µm 60 Å silica gel from Sigma-Aldrich.

Melting points (mp) were recorded on a Griffin melting point apparatus to the nearest degree and are uncorrected.

All air and/or moisture sensitive reactions were performed under an atmosphere of dry nitrogen using anhydrous solvents. All solvents for air sensitive reactions were degassed by bubbling N_2 on the Schlenk line. All commercially available reagents and solvents were used as received without further purification.

2. General procedures

General procedure A

Sulfone Synthesis

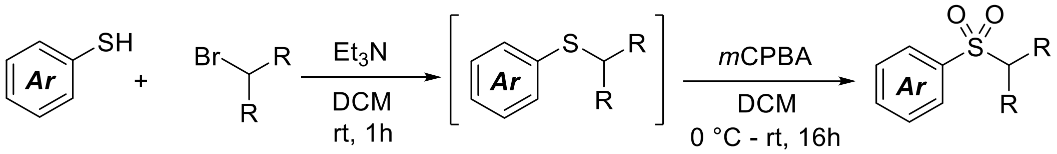

A 100 mL round bottom flask was charged with the corresponding thiophenol (1 eq.) and alkyl bromide (1 eq.). DCM (0.1 M) was then added via syringe, followed by triethylamine (1 eq.). The resulting solution was then left to stir for one hour. The reaction mixture was then diluted (DCM) and washed (aq. $NaHCO_3$). The organic phase was then dried ($MgSO_4$), filtered and concentrated in vacuo. The crude sulfide was then used immediately without purification. DCM (0.1 M) was added to a 100 mL round bottom flask containing the crude sulfide. This was cooled to 0 °C using an ice bath, and then *m*CPBA (77%, 3 eq.) was added portion-wise. The resulting mixture was then left to stir overnight. After this, the mixture was quenched (aq. $Na_2S_2O_3$), the organic phase was extracted (DCM x3) and the combined organic phase was washed (aq. $NaHCO_3$). The organic phase was then dried ($MgSO_4$), filtered and concentrated in vacuo. If necessary, the crude product was purified using flash column chromatography.

General procedure B

Cyclopropane Synthesis

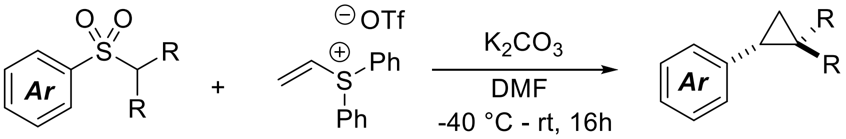

A 2-5 mL oven-dried microwave vial was charged with the corresponding sulfone (0.4 mmol, 2 eq.), diphenyl(vinyl)sulfonium trifluoromethanesulfonate (72 mg, 0.2 mmol, 1 eq.) and K_2CO_3 (83 mg, 0.6 mmol, 3 eq.). The vial was sealed, evacuated and backfilled with nitrogen. The vial was cooled to -40 °C using a MeCN/dry ice cooling bath, and then anhydrous DMF (2 mL, 0.1 M) was added via syringe. The resulting mixture was then left to stir overnight. The reaction mixture was then diluted (EtOAc) and washed (aq. LiCl x3). The organic phase was then dried ($MgSO_4$), filtered and concentrated *in vacuo*. The crude product was purified using flash column chromatography.

3. Troublesome and unsuccessful substrates

Heteroarenes

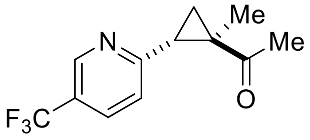

0%

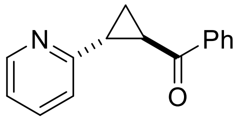

0%

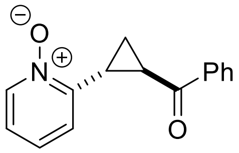

12%

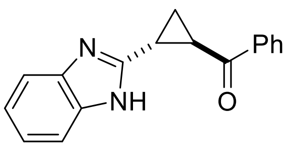

0%

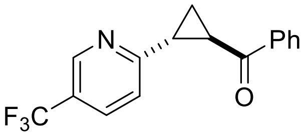

14%

Electron-withdrawing groups

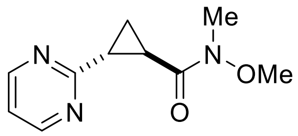

0%

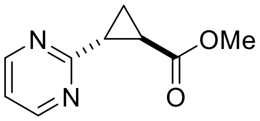

0%

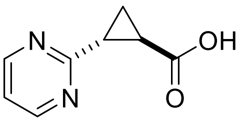

0%

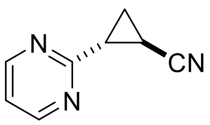

0%

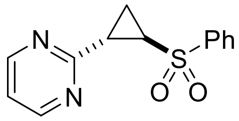

0%

Sultines

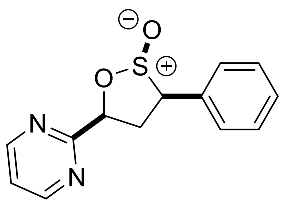

<5%

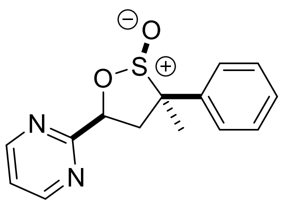

<5%

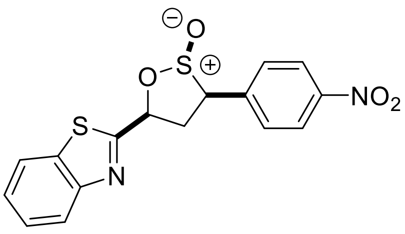

<5%

4. Compound characterization

1-phenyl-2-(pyrimidin-2-ylsulfonyl)ethan-1-one (1a)

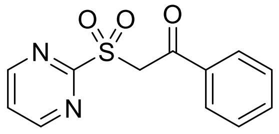

Prepared according to general procedure A (10 mmol scale, no column chromatography needed). The pure product was afforded as a yellow solid (1.86 g, 71% yield).

^{1}H NMR (400 MHz, CDCl$_3$) δ 8.94 (d, *J* = 4.8 Hz, 2H), 7.94 – 7.91 (m, 2H), 7.66 – 7.61 (m, 1H), 7.57 (t, *J* = 4.9 Hz, 1H), 7.52 – 7.48 (m, 2H), 5.23 (s, 2H).

^{13}C NMR (100 MHz, CDCl$_3$) δ 188.5, 165.5, 158.8, 135.6, 134.7, 129.1, 128.9, 123.9, 58.3.

HRMS (ESI) m/z: $[M+Na]^+$ Calculated for $[C_{12}H_{10}O_3N_2SNa]^+$ 285.0304; found 285.0296.

Data are in accordance with literature.[1]

1-(4-chlorophenyl)-2-(pyrimidin-2-ylsulfonyl)ethan-1-one (1b)

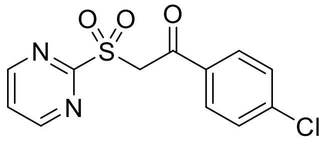

Prepared according to general procedure A (2.5 mmol scale, no column chromatography needed). The pure product was afforded as a yellow solid (541 mg, 73% yield).

^{1}H NMR (400 MHz, DMSO) δ 9.07 (d, *J* = 4.9 Hz, 2H), 8.03 – 8.00 (m, 2H), 7.86 (t, *J* = 4.9 Hz, 1H), 7.65 – 7.62 (m, 2H), 5.71 (s, 2H).

^{13}C NMR (100 MHz, DMSO) δ 188.7, 165.0, 159.2, 139.4, 134.1, 130.8, 129.0, 124.7, 58.1.

HRMS (ESI) m/z: $[M+Na]^+$ Calculated for $[C_{12}H_9O_3N_2SClNa]^+$ 318.9915; found 318.9907.

Data are in accordance with literature.[1]

1-(4-fluorophenyl)-2-(pyrimidin-2-ylsulfonyl)ethan-1-one (1c)

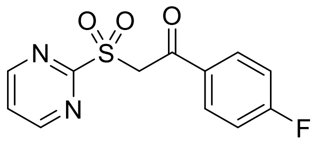

Prepared according to general procedure A (2.5 mmol scale, no column chromatography needed). The pure product was afforded as an off-white solid (512 mg, 74% yield).

^{1}H NMR (400 MHz, CDCl$_3$) δ 8.96 (d, *J* = 4.8 Hz, 2H), 8.01 – 7.96 (m, 2H), 7.58 (t, *J* = 4.8 Hz, 1H), 7.20 – 7.15 (m, 2H), 5.19 (s, 2H).

^{13}C NMR (100 MHz, CDCl$_3$) δ 186.8, 166.7 (d, *J* = 258.1 Hz), 165.4, 158.8, 132.1 (d, *J* = 3.0 Hz), 131.9 (d, *J* = 9.8 Hz), 124.0, 116.3, 58.3.

^{19}F NMR (376 MHz, CDCl$_3$) δ -101.87 – -101.94 (m).

HRMS (APCI) m/z: $[M+H]^+$ Calculated for $[C_{12}H_{10}O_3N_2SF]^+$ 281.0391; found 281.0384.

Data are in accordance with literature.[1]

1-([1,1'-biphenyl]-4-yl)-2-(pyrimidin-2-ylsulfonyl)ethan-1-one (1d)

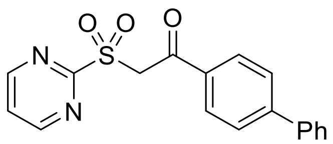

Prepared according to general procedure A (2.5 mmol scale, no column chromatography needed). The pure product was afforded as a yellow solid (565 mg, 67% yield).

^{1}**H NMR (400 MHz, CDCl$_3$)** δ 8.96 (d, *J* = 4.8 Hz, 2H), 8.02 – 7.99 (m, 2H), 7.74 – 7.71 (m, 2H), 7.64 – 7.61 (m, 2H), 7.58 (t, *J* = 4.8 Hz, 1H), 7.48 (tt, *J* = 6.5, 1.1 Hz, 2H), 7.44 – 7.40 (m, 1H), 5.26 (s, 2H).

^{13}**C NMR (100 MHz, CDCl$_3$)** δ 187.9, 165.5, 158.8, 147.5, 139.5, 134.3, 129.6, 129.2, 128.8, 127.7, 127.5, 123.9, 58.4.

HRMS (ESI) m/z: $[M+Na]^+$ Calculated for $[C_{18}H_{14}O_3N_2SNa]^+$ 361.0617; found 361.0604.

mp: 158-160 °C.

1-(4-methoxyphenyl)-2-(pyrimidin-2-ylsulfonyl)ethan-1-one (1e)

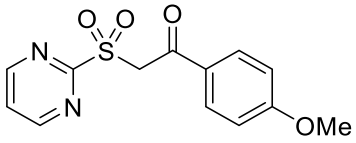

Prepared according to general procedure A (2.5 mmol scale, no column chromatography needed). The pure product was afforded as a yellow solid (561 mg, 77% yield).

^{1}**H NMR (400 MHz, CDCl$_3$)** δ 8.95 (d, *J* = 4.9 Hz, 2H), 7.92 – 7.89 (m, 2H), 7.56 (t, *J* = 4.8 Hz, 1H), 6.97 – 6.94 (m, 2H), 5.17 (s, 2H), 3.88 (s, 3H).

^{13}**C NMR (100 MHz, CDCl$_3$)** δ 186.7, 165.7, 164.9, 158.8, 131.5, 128.7, 123.9, 114.4, 58.2, 55.8.

HRMS (APCI) m/z: $[M+H]^+$ Calculated for $[C_{13}H_{13}O_4N_2S]^+$ 293.0591; found 293.0582.

Data are in accordance with literature.[1]

methyl-4-(2-(pyrimidin-2-ylsulfonyl)acetyl)benzoate (1f)

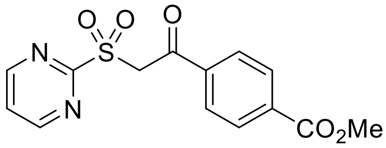

Prepared according to general procedure A (2.5 mmol scale, no column chromatography needed). The pure product was afforded as an off-white solid (522 mg, 65% yield).

^{1}**H NMR (400 MHz, CDCl$_3$)** δ 8.95 (d, *J* = 4.9 Hz, 2H), 8.17 – 8.14 (m, 2H), 8.01 – 7.98 (m, 2H), 7.59 (t, *J* = 4.9 Hz, 1H), 5.24 (s, 2H), 3.96 (s, 3H).

^{13}**C NMR (100 MHz, CDCl$_3$)** δ 188.1, 166.0, 165.4, 158.8, 138.7, 135.3, 130.3, 128.9, 124.0, 58.5, 52.8.

HRMS (APCI) m/z: $[M+H]^+$ Calculated for $[C_{14}H_{13}O_5N_2S]^+$ 321.0540; found 321.0537.

mp: 130-134 °C.

2-(pyrimidin-2-ylsulfonyl)-1-(thiophen-2-yl)ethan-1-one (1g)

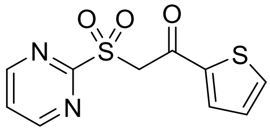

Prepared according to general procedure A (2.5 mmol scale, no column chromatography needed). The pure product was afforded as a yellow solid (344 mg, 51% yield).

^{1}**H NMR (400 MHz, CDCl$_3$)** δ 8.96 (d, *J* = 4.9 Hz, 2H), 7.84 (dd, *J* = 4.0, 1.1 Hz, 1H), 7.77 (dd, *J* = 4.9, 1.1 Hz, 1H), 7.58 (t, *J* = 4.9 Hz, 1H), 7.20 (dd, *J* = 4.9, 3.9 Hz, 1H), 5.12 (s, 2H).

^{13}**C NMR (100 MHz, CDCl$_3$)** δ 180.4, 165.3, 158.8, 142.9, 136.7, 134.8, 128.9, 124.0, 59.0.

HRMS (APCI) m/z: $[M+H]^+$ Calculated for $[C_{10}H_9O_3N_2S_2]^+$ 269.0049; found 269.0045.

mp: 91-94 °C.

3-(pyrimidin-2-ylsulfonyl)butan-2-one (1h)

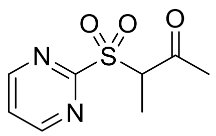

Prepared according to general procedure A (2.5 mmol scale, no column chromatography needed). The pure product was afforded as a brown oil (319 mg, 60% yield).

^{1}H NMR (400 MHz, CDCl$_3$) δ 8.96 (d, *J* = 4.8 Hz, 2H), 7.59 (t, *J* = 4.8 Hz, 1H), 4.67 (d, *J* = 7.2 Hz, 1H), 2.48 (s, 3H), 1.65 (d, *J* = 7.2 Hz, 3H).

^{13}C NMR (100 MHz, CDCl$_3$) δ 199.8, 164.7, 158.8, 124.1, 67.5, 30.0, 10.9.

HRMS (APCI) m/z: $[M+H]^+$ Calculated for $[C_8H_{11}O_3N_2S]^+$ 215.0485; found 215.0495.

1-phenyl-2-(pyrimidin-2-ylsulfonyl)ethan-1-one (1l)

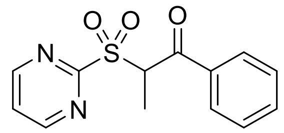

Prepared according to general procedure A (2.5 mmol scale, column conditions 20-100% EtOAc in hexane). The pure product was afforded as a yellow solid (541 mg, 78% yield).

^{1}H NMR (400 MHz, CDCl$_3$) δ 8.90 (d, *J* = 4.8 Hz, 2H), 7.99 – 7.96 (m, 2H), 7.64 – 7.59 (m, 1H), 7.54 – 7.47 (m, 3H), 5.70 (q, *J* = 7.1 Hz, 1H), 1.82 (d, *J* = 7.2 Hz, 3H).

^{13}C NMR (100 MHz, CDCl$_3$) δ 192.5, 165.0, 158.7, 135.7, 134.3, 129.1, 129.0, 124.0, 62.3, 12.6.

HRMS (ESI) m/z: $[M+Na]^+$ Calculated for $[C_{13}H_{12}O_3NS_2Na]^+$ 299.0461; found 299.0453.

mp: 79-82 °C.

2-(pyrimidin-2-ylsulfonyl)-3,4-dihydronaphthalen-1(2H)-one (1j)

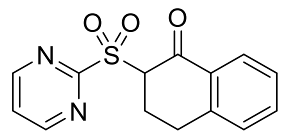

Prepared according to general procedure A (2.5 mmol scale, column conditions 20-100% EtOAc in hexane). The pure product was afforded as a white solid (213 mg, 30% yield).

^{1}H NMR (400 MHz, CDCl$_3$) δ 8.97 (d, *J* = 4.9 Hz, 2H), 7.89 (dd, *J* = 8.1, 1.5 Hz, 1H), 7.58 – 7.51 (m, 2H), 7.32 – 7.28 (m, 2H), 5.11 (dd, *J* = 10.3, 5.8 Hz, 1H), 3.37 (dt, *J* = 16.8, 5.0 Hz, 1H), 3.14 (ddd, *J* = 16.3, 10.0, 5.1 Hz, 1H), 2.94 – 2.80 (m, 2H).

^{13}C NMR (100 MHz, CDCl$_3$) δ 189.3, 166.3, 158.7, 143.7, 134.9, 131.6, 129.0, 128.0, 127.4, 123.6, 65.38, 27.7, 21.5.

HRMS (APCI) m/z: $[M+H]^+$ Calculated for $[C_{14}H_{13}O_3N_2S]^+$ 289.0641; found 289.0634.

mp: 132-134 °C.

diethyl 2-(pyrimidin-2-ylsulfonyl)malonate (1k)

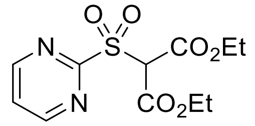

Prepared according to general procedure A (2.5 mmol scale, no column chromatography needed) Alkyl chloride used in place of alkyl bromide. The pure product was afforded as an off-white oil (261 mg, 35% yield).

^{1}H NMR (400 MHz, CDCl$_3$) δ 8.96 (d, *J* = 4.8 Hz, 2H), 7.60 (t, *J* = 4.9 Hz, 1H), 5.81 (s, 1H), 4.29 (q, *J* = 7.2 Hz, 4H), 1.25 (t, *J* = 7.1 Hz, 6H).

^{13}C NMR (100 MHz, CDCl$_3$) δ 165.3, 160.8, 158.8, 124.1, 69.6, 63.5, 13.9.

HRMS (APCI) m/z: $[M+H]^+$ Calculated for $[C_{11}H_{15}O_6N_2S]^+$ 303.0645; found 303.0640.

2-(benzo[d]thiazol-2-ylsulfonyl)-1-phenylethan-1-one (1l)

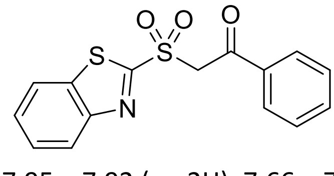

Prepared according to general procedure A (2.5 mmol scale, no column chromatography needed). The pure product was afforded as an off-white solid (636 mg, 80% yield).

^{1}H NMR (400 MHz, CDCl$_3$) δ 8.21 – 8.19 (m, 1H), 8.02 – 8.00 (m, 1H), 7.95 – 7.92 (m, 2H), 7.66 – 7.58 (m, 3H), 7.50 – 7.45 (m, 2H), 5.20 (s, 2H).

^{13}C NMR (100 MHz, CDCl$_3$) δ 187.3, 165.5, 152.6, 137.2, 135.6, 134.8, 129.2, 129.1, 128.3, 127.8, 125.7, 122.5, 61.3.

HRMS (ESI) m/z: $[M+Na]^+$ Calculated for $[C_{15}H_{11}O_3NS_2Na]^+$ 340.0073; found 340.0065.

Data are in accordance with literature.[2]

3-(benzo[d]thiazol-2-ylsulfonyl)butan-2-one (1m)

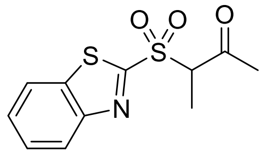

Prepared according to general procedure A (2.5 mmol scale, no column chromatography needed). The pure product was afforded as a yellow solid (569 mg, 85% yield).

^{1}H NMR (400 MHz, CDCl$_3$) δ 8.24 – 8.21 (m, 1H), 8.03 – 8.00 (m, 1H), 7.67 – 7.59 (m, 2H), 4.63 (q, *J* = 7.1 Hz, 1H), 2.52 (s, 3H), 1.63 (d, *J* = 7.1 Hz, 3H).

^{13}C NMR (100 MHz, CDCl$_3$) δ 198.6, 163.8, 152.7, 137.2, 128.5, 128.0, 125.9, 122.5, 70.1, 30.7, 11.6.

HRMS (APCI) m/z: $[M+H]^+$ Calculated for $[C_{11}H_{12}O_3NS_2]^+$ 270.0253; found 270.0243.

mp: 76-78 °C.

2-((6-methylbenzo[d]thiazol-2-yl)sulfonyl)-1-phenylethan-1-one (1n)

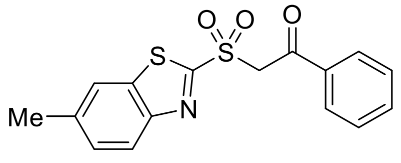

Prepared according to general procedure A (2.0 mmol scale, column conditions 0-15% EtOAc in hexane). The pure product was afforded as a yellow solid (595 mg, 90% yield).

^{1}H NMR (400 MHz, DMSO-d$_6$) δ 8.09 (d, *J* = 8.7 Hz, 2H), 8.00 – 7.98 (m, 2H), 7.69 – 7.65 (m, 1H), 7.54 – 7.50 (m, 3H), 5.81 (s, 2H), 2.08 (s, 3H).

^{13}C NMR (100 MHz, DMSO-d$_6$) δ 188.6, 165.2, 150.1, 138.5, 136.6, 135.3, 134.5, 129.6, 129.0, 128.8, 124.4, 122.6, 61.3, 21.3.

HRMS (APCI) m/z: $[M+Na]^+$ Calculated for $[C_{16}H_{13}O_3NS_2Na]^+$ 354.0229; found 354.0225.

mp: 68-72 °C.

2-((5-bromobenzo[d]thiazol-2-yl)sulfonyl)-1-phenylethan-1-one (3o)

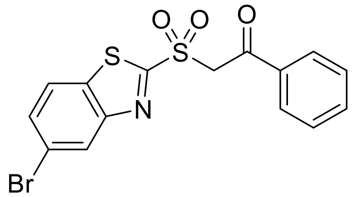

Prepared according to general procedure A (2.0 mmol scale, column conditions 0-15% EtOAc in hexane). The pure product was afforded as a yellow solid (561 mg, 71% yield).

^{1}H NMR (400 MHz, DMSO-d$_6$) δ 8.48 (d, *J* = 1.9 Hz, 1H), 8.32 (d, *J* = 8.8 Hz, 1H), 8.00 – 7.98 (m, 2H), 7.86 (dd, *J* = 8.8, 1.9 Hz, 1H), 7.69 (t, *J* = 7.3 Hz, 1H), 7.53 (t, *J* = 7.8 Hz, 2H), 5.87 (s, 2H).

^{13}C NMR (100 MHz, DMSO-d$_6$) δ 188.7, 168.4, 152.9, 135.6, 135.2, 134.6, 131.1, 129.0, 128.9, 127.1, 125.4, 120.8, 61.4.

HRMS (APCI) m/z: $[M+H]^+$ Calculated for $[C_{15}H_{10}O_3NS_2Br]^+$ 395.9358; found 395.9355.

mp: 156-158 °C.

2-((5-fluorobenzo[d]thiazol-2-yl)sulfonyl)-1-phenylethan-1-one (3p)

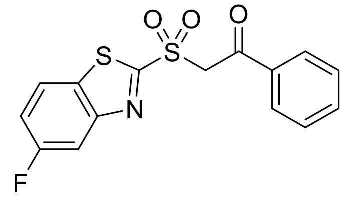

Prepared according to general procedure A (2.0 mmol scale, column conditions 0-15% EtOAc in hexane). The pure product was afforded as a white solid (334 mg, 50% yield).

^{1}H NMR (400 MHz, DMSO-d$_6$) δ 8.25 (ddd, *J* = 12.0, 8.9, 3.8 Hz, 2H), 8.01 – 7.98 (m, 2H), 7.70 – 7.65 (m, 1H), 7.60 (td, *J* = 9.0, 2.7 Hz, 1H), 7.52 (t, *J* = 7.8 Hz, 2H), 5.86 (s, 2H).

^{19}F NMR (376 MHz, DMSO-d$_6$) δ -110.91 (td, *J* = 8.8, 4.9 Hz).

^{13}C NMR (100 MHz, DMSO-d$_6$) δ 188.7, 166.5 (d, J = 3.5 Hz), 162.3, 159.9, 148.8, 137.9 (d, J = 12.5 Hz), 135.2, 134.5, 128.9 (d, J = 12.5 Hz), 126.6 (d, J = 9.9 Hz), 117.2 (d, J = 25.4 Hz), 109.6 (d, J = 27.7 Hz), 61.4.

HRMS (APCI) m/z: $[M+H]^+$ Calculated for $[C_{15}H_{11}O_3NFS_2]^+$ 336.0159; found 336.0155.

mp: 110-114 °C.

2-((7-chlorobenzo[d]thiazol-2-yl)sulfonyl)-1-phenylethan-1-one (3q)

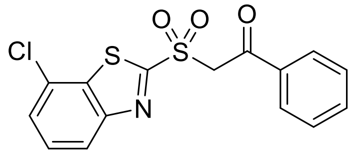

Prepared according to general procedure A (2.0 mmol scale, column conditions 0-15% EtOAc in hexane). The pure product was afforded as a white solid (545 mg, 77% yield).

^{1}H NMR (400 MHz, DMSO-d$_6$) δ 8.24 (dd, *J* = 8.2, 1.0 Hz, 1H), 8.00 – 7.98 (m, 2H), 7.84 (dd, *J* = 7.9, 1.0 Hz, 1H), 7.76 (t, *J* = 8.0 Hz, 1H), 7.70 – 7.66 (m, 1H), 7.52 (t, *J* = 7.8 Hz, 2H), 5.94 (s, 2H).

^{13}C NMR (100 MHz, DMSO-d$_6$) δ 188.8, 166.8, 152.4, 136.3, 135.2, 134.7, 129.6, 129.0, 128.9, 128.9, 128.0, 126.2, 124.0, 61.5.

HRMS (APCI) m/z: $[M]^+$ Calculated for $[C_{15}H_{11}O_3NS_2Cl]^+$ 351.9863; found 351.9862.

mp: 104-106 °C.

2-((4-methylpyrimidin-2-yl)sulfonyl)-1-phenylethan-1-one (1r)

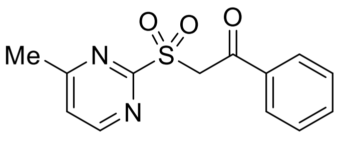

Prepared according to general procedure A (5.0 mmol scale, column conditions 0-80% EtOAc in hexane). The pure product was afforded as a yellow solid (803 mg, 58% yield).

^{1}H NMR (400 MHz, DMSO-d$_6$) δ 8.91 (d, *J* = 5.1 Hz, 1H), 8.05 – 8.03 (m, 2H), 7.76 – 7.72 (m, 2H), 7.59 (t, *J* = 7.8 Hz, 2H), 5.68 (s, 2H), 2.58 (s, 3H).

^{13}C NMR (100 MHz, DMSO-d$_6$) δ 189.5, 169.8, 164.6, 158.3, 135.5, 134.3, 128.8, 124.0, 58.0, 23.5.

HRMS (APCI) m/z: $[M+H]^+$ Calculated for $[C_{13}H_{13}O_3N_2S]^+$277.0641; found 277.0639.

mp: 98-100 °C.

1-phenyl-2-((4-(trifluoromethyl)pyrimidin-2-yl)sulfonyl)ethan-1-one (1s)

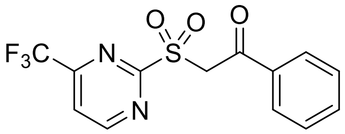

Prepared according to general procedure A (2.0 mmol scale, column conditions 0-45% EtOAc in hexane). The pure product was afforded as a white solid (608 mg, 92% yield).

^{1}H NMR (400 MHz, CDCl$_3$) δ 9.23 (d, *J* = 5.0 Hz, 1H), 7.91 – 7.88 (m, 3H), 7.64 (ddt, *J* = 8.7, 7.1, 1.3 Hz, 1H), 7.52 – 7.47 (m, 2H), 5.30 (s, 2H).

^{19}F NMR (376 MHz, CDCl$_3$) δ -69.38.

^{13}C NMR (100 MHz, CDCl$_3$) δ 188.5, 166.0, 161.7, 157.2 (q, *J* = 38 Hz), 135.3, 134.9, 129.2, 128.8, 119.7 (q, *J* = 274 Hz), 119.8 (q, *J* = 3 Hz), 58.2.

HRMS (APCI) m/z: $[M+H]^+$ Calculated for $[C_{13}H_{10}O_3N_2SF_3]^+$ 331.0359; found 331.0354.

mp: 94-96 °C.

N-benzyl-4-nitrobenzenesulfonamide (4a)

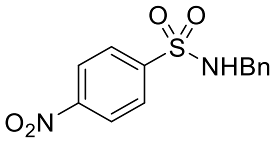

A 100 mL round bottom flask was charged with 4-Nitrobenzenesulfonyl chloride (1 eq., 10 mmol, 2.21 g) and DCM (0.5 M, 20 mL) was added *via* syringe. Benzylamine (2.5 eq., 25 mmol, 2.73 mL) was then added *via* syringe and the resulting solution was left to stir overnight. The reaction mixture was then diluted (DCM) and washed (aq. HCl). The organic phase was then dried ($MgSO_4$), filtered and concentrated *in vacuo,* affording the pure product as an off-white solid (2.39 g, 82% yield).

^{1}H NMR (400 MHz, CDCl$_3$) δ 8.33 – 8.29 (m, 2H), 8.01 – 7.99 (m, 2H), 7.29 – 7.26 (m, 3H), 7.20 – 7.16 (m, 2H), 4.99 (t, J = 6.1 Hz, 1H), 4.24 (d, J = 5.9 Hz, 2H).

^{13}C NMR (100 MHz, CDCl$_3$) δ 150.1, 146.2, 135.6, 129.0, 128.5, 128.4, 128.0, 124.4, 47.6.

HRMS (APCI) m/z: $[M+H]^+$ Calculated for $[C_{13}H_{11}O_4N_2S]^+$ 291.0445; found 291.0438.

Data are in accordance with literature.[3]

2-((4-nitrobenzyl)sulfonyl)pyrimidine (6a)

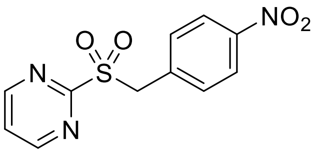

Prepared according to general procedure A (2.5 mmol scale, no column chromatography needed). The pure product was afforded as an off-white solid (463 mg, 66% yield).

^{1}H NMR (400 MHz, CDCl$_3$) δ 8.96 (d, J = 4.9 Hz, 2H), 8.21 – 8.17 (m, 2H), 7.63 – 7.58 (m, 3H), 4.90 (s, 2H).

^{13}C NMR (100 MHz, CDCl$_3$) δ 165.3, 158.9, 148.4, 134.1, 132.5, 124.2, 124.0, 56.9.

HRMS (APCI) m/z: $[M+H]^+$ Calculated for $[C_{11}H_{10}O_4N_3S]^+$ 280.0387; found 280.0378.

mp: 167-170 °C.

2-((2-nitrobenzyl)sulfonyl)pyrimidine (6b)

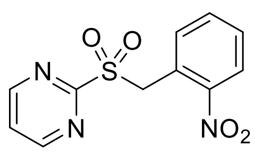

Prepared according to general procedure A (5.0 mmol scale, column conditions 0-10% MeOH in EA). The pure product was afforded as a white solid (1.2 g, 86% yield).

^{1}H NMR (400 MHz, DMSO-d$_6$) δ 9.13 (d, *J* = 4.9 Hz, 2H), 8.10 (dd, *J* = 8.1, 1.4 Hz, 1H), 7.92 (t, *J* = 4.9 Hz, 1H), 7.78 (td, *J* = 7.5, 1.4 Hz, 1H), 7.70 (ddd, *J* = 13.2, 7.5, 1.6 Hz, 2H), 5.51 (s, 2H).

^{13}C NMR (100 MHz, DMSO-d$_6$) δ 164.5, 159.3, 149.8, 134.8, 133.5, 130.5, 125.4, 125.0, 121.8, 52.8.

HRMS (APCI) m/z: $[M+H]^+$ Calculated for $[C_{11}H_{10}O_4N_3S]^+$ 280.0387; found 280.0384.

mp: 138-142 °C.

4-((pyrimidin-2-ylsulfonyl)methyl)benzonitrile (6c)

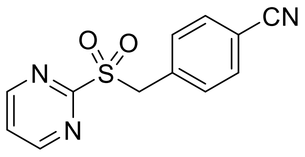

Prepared according to general procedure A (5.0 mmol scale, column conditions 0-100% EtOAc in hexane). The pure product was afforded as a white solid (1.1 g, 88% yield).

^{1}H NMR (400 MHz, DMSO-d$_6$) δ 9.15 (d, *J* = 4.9 Hz, 2H), 7.92 (t, *J* = 4.9 Hz, 1H), 7.87 (d, *J* = 8.3 Hz, 2H), 7.60 (d, *J* = 8.2 Hz, 2H), 5.18 (s, 2H).

^{13}C NMR (100 MHz, DMSO-d$_6$) δ 164.6, 159.3, 133.3, 132.4, 132.2, 124.9, 118.5, 111.4, 56.1.

HRMS (+APCI) m/z: $[M+H]^+$ Calculated for $[C_{12}H_{10}O_2N_3S]^+$ 260.0488; found 260.0488.

2-((3-methyl-4-nitrobenzyl)sulfonyl)pyrimidine (6d)

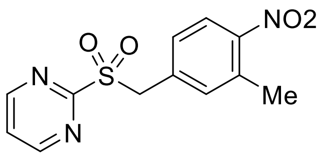

Prepared according to general procedure A (4.0 mmol scale, column conditions 0-10% MeOH in EA). The pure product was afforded as a white solid (952 mg, 81% yield).

^{1}H NMR (400 MHz, DMSO-d$_6$) δ 9.16 (d, *J* = 4.9 Hz, 2H), 8.00 (d, *J* = 8.4 Hz, 1H), 7.93 (t, *J* = 4.9 Hz, 1H), 7.51 (s, 1H), 7.47 (dd, *J* = 8.3, 1.9 Hz, 1H), 5.15 (s, 2H), 2.52 (s, 3H).

^{13}C NMR (100 MHz, DMSO-d$_6$) δ 164.6, 159.3, 148.8, 135.6, 133.3, 132.7, 130.3, 125.0, 124.5, 55.7, 19.4.

HRMS (APCI) m/z: $[M+H]^+$ Calculated for $[C_{12}H_{12}O_4N_3S]^+$ 294.0543; found 294.0541.

mp: 130-132 °C.

phenyl(2-(pyrimidin-2-yl)cyclopropyl)methanone (3a)

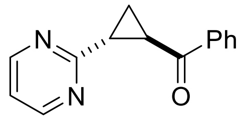

Prepared according to general procedure B (0.2 mmol scale, column conditions 0-70% EtOAc in hexane). The pure product was afforded as an off-white solid (28 mg, 63% yield).

2 mmol scale reaction:

A 100 mL oven-dried round bottom flask was charged with 1-phenyl-2-(pyrimidin-2-ylsulfonyl)ethan-1-one (1.06 g, 4 mmol, 2 eq.), Diphenyl(vinyl)sulfonium trifluoromethanesulfonate (725 mg, 2 mmol, 1 eq.) and K_2CO_3 (828 mg, 6 mmol, 3 eq.). The flask was sealed, evacuated and backfilled with nitrogen. The flask was cooled to -40 °C using a MeCN/dry ice cooling bath, and then anhydrous DMF (20 mL, 0.1 M) was added *via* syringe. The resulting mixture was then left to stir overnight. The reaction mixture was then diluted (EtOAc) and washed (aq. LiCl x3). The organic phase was then dried ($MgSO_4$), filtered and concentrated *in vacuo.* The crude product was purified using flash column chromatography, affording an off-white solid (191 mg, 43% yield).

^{1}H NMR (400 MHz, CDCl$_3$) δ 8.62 (d, *J* = 4.9 Hz, 2H), 8.04 – 8.01 (m, 2H), 7.58 – 7.54 (m, 1H), 7.48 – 7.44 (m, 2H), 7.13 (t, *J* = 4.9 Hz, 1H), 3.37 (ddd, *J* = 8.4, 5.6, 3.8 Hz, 1H), 3.01 (ddd, *J* = 8.8, 6.0, 3.8 Hz, 1H), 1.93 (ddd, *J* = 8.8, 5.5, 3.3 Hz, 1H), 1.81 (ddd, *J* = 8.3, 6.0, 3.3 Hz, 1H).

^{13}C NMR (100 MHz, CDCl$_3$) δ 198.1, 169.3, 157.1, 137.7, 133.1, 128.7, 128.4, 118.9, 31.1, 29.1, 20.6.

HRMS (APCI) m/z: $[M+H]^+$ Calculated for $[C_{14}H_{13}ON_2]^+$ 225.1022; found 225.1015.

mp: 41-45 °C.

(4-chlorophenyl)(2-(pyrimidin-2-yl)cyclopropyl)methanone (3b)

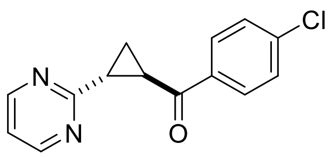

Prepared according to general procedure B (0.2 mmol scale, column conditions 0-80% EtOAc in hexane). The pure product was afforded as a white solid (30 mg, 59% yield).

^{1}H NMR (400 MHz, CDCl$_3$) δ 8.62 (d, *J* = 4.8 Hz, 2H), 7.98 – 7.95 (m, 2H), 7.45 – 7.41 (m, 2H), 7.13 (t, *J* = 4.9 Hz, 1H), 3.31 (ddd, *J* = 8.3, 5.5, 3.8 Hz, 1H), 3.01 (ddd, *J* = 8.7, 6.0, 3.7 Hz, 1H), 1.93 (ddd, *J* = 8.8, 5.4, 3.3 Hz, 1H), 1.82 (ddd, *J* = 8.3, 6.1, 3.3 Hz, 1H).

^{13}C NMR (100 MHz, CDCl$_3$) δ 196.9, 169.1, 157.2, 139.6, 136.0, 129.8, 129.0, 118.9, 31.2, 29.0, 20.7.

HRMS (APCI) m/z: $[M+H]^+$ Calculated for $[C_{14}H_{12}ON_2Cl]^+$ 259.0633; found 259.0627.

mp: 95-98 °C.

XRD Recrystallized from EtOAc/Hexane as white crystals. Single crystal analysis confirms the structure drawn (CCDC deposition number: 2374654)

(4-fluorophenyl)(2-(pyrimidin-2-yl)cyclopropyl)methanone (3c)

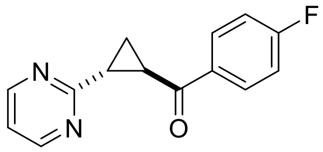

Prepared according to general procedure B (0.2 mmol scale, column conditions 0-80% EtOAc in hexane). The pure product was afforded as a white solid (20 mg, 41% yield).

^{1}H NMR (500 MHz, CDCl$_3$) δ 8.62 (d, *J* = 4.9 Hz, 2H), 8.08 – 8.04 (m, 2H), 7.15 – 7.10 (m, 3H), 3.32 (ddd, *J* = 8.3, 5.5, 3.8 Hz, 1H), 3.00 (ddd, *J* = 8.7, 6.0, 3.8 Hz, 1H), 1.92 (ddd, *J* = 8.8, 5.6, 3.4 Hz, 1H), 1.81 (ddd, *J* = 8.3, 6.1, 3.3 Hz, 1H).

^{19}F NMR (471 MHz, CDCl$_3$) δ -105.45.

^{13}C NMR (126 MHz, CDCl$_3$) δ 196.5, 169.2, 165.9 (d, J = 254.7 Hz), 157.2, 134.1 (d, J = 2.9 Hz), 131.0 (d, J = 9.3 Hz), 118.9, 115.8 (d, J = 21.7 Hz), 31.1, 29.0, 20.6.

HRMS (APCI) m/z: $[M+H]^+$ Calculated for $[C_{14}H_{12}ON_2F]^+$ 243.0928; found 243.0919.

mp: 80-82 °C.

[1,1'-biphenyl]-4-yl(2-(pyrimidin-2-yl)cyclopropyl)methanone (3d)

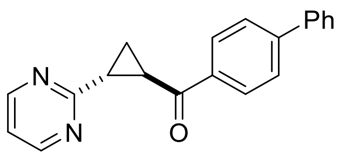

Prepared according to general procedure B (0.2 mmol scale, column conditions 0-60% EtOAc in hexane). The pure product was afforded as an off-white solid (37 mg, 62% yield).

^{1}H NMR (500 MHz, CDCl$_3$) δ 8.63 (d, *J* = 4.9 Hz, 2H), 8.11 (d, *J* = 8.5 Hz, 2H), 7.70 – 7.67 (m, 2H), 7.63 – 7.61 (m, 2H), 7.48 – 7.45 (m, 2H), 7.41 – 7.37 (m, 1H), 7.13 (t, *J* = 4.9

Hz, 1H), 3.41 (ddd, *J* = 8.4, 5.5, 3.8 Hz, 1H), 3.04 (ddd, *J* = 8.9, 6.0, 3.8 Hz, 1H), 1.96 (ddd, *J* = 8.8, 5.6, 3.3 Hz, 1H), 1.87 – 1.82 (m, 1H).

^{13}C NMR (126 MHz, CDCl$_3$) δ 197.6, 169.3, 157.1, 145.8, 140.0, 136.4, 129.1, 129.0, 128.3, 127.4, 127.3, 118.9, 31.1, 29.2, 20.6.

HRMS (APCI) m/z: [M+H]$^+$ Calculated for [$C_{20}H_{17}ON_2$]$^+$ 301.1335; found 301.1326.

mp: 120-123 °C.

(4-methoxyphenyl)(2-(pyrimidin-2-yl)cyclopropyl)methanone (3e)

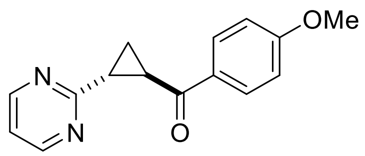

Prepared according to general procedure B (0.2 mmol scale, column conditions 0-80% EtOAc in hexane). The pure product was afforded as an off-white solid (35 mg, 69% yield).

^{1}H NMR (500 MHz, CDCl$_3$) δ 8.62 (d, *J* = 4.9 Hz, 2H), 8.03 – 8.01 (m, 2H), 7.12 (t, *J* = 4.9 Hz, 1H), 6.96 – 6.90 (m, 2H), 3.86 (s, 3H), 3.32 (ddd, *J* = 8.4, 5.5, 3.8 Hz, 1H), 2.98 (ddd, *J* = 8.8, 6.0, 3.9 Hz, 1H), 1.91 (td, *J* = 5.6, 2.8 Hz, 1H), 1.77 (ddd, *J* = 8.4, 6.0, 3.3 Hz, 1H).

^{13}C NMR (126 MHz, CDCl$_3$) δ 196.4, 169.5, 163.6, 157.1, 130.8, 130.7, 118.8, 113.8, 55.6, 30.7, 28.8, 20.3.

HRMS (APCI) m/z: [M+H]$^+$ Calculated for [$C_{15}H_{15}O_2N_2$]$^+$ 255.1128; found 255.1116.

mp: 79-81 °C.

methyl 4-(2-(pyrimidin-2-yl)cyclopropane-1-carbonyl)benzoate (3f)

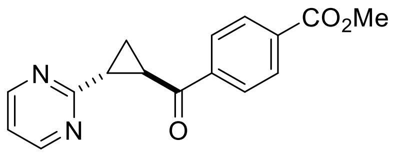

Prepared according to general procedure B (0.2 mmol scale, column conditions 0-70% EtOAc in hexane). The pure product was afforded as a yellow solid (30 mg, 53% yield).

^{1}H NMR (400 MHz, CDCl$_3$) δ 8.63 (d, *J* = 4.9 Hz, 2H), 8.13 – 8.04 (m, 4H), 7.14 (t, *J* = 4.8 Hz, 1H), 3.94 (s, 3H), 3.37 (ddd, *J* = 8.6, 5.5, 3.8 Hz, 1H), 3.04 (ddd, *J* = 9.1, 6.1, 3.7 Hz, 1H), 1.95 (ddd, *J* = 9.0, 5.5, 3.3 Hz, 1H), 1.88 – 1.84 (m, 1H).

^{13}C NMR (100 MHz, CDCl$_3$) δ 197.8, 169.0, 166.4, 157.2, 140.9, 133.9, 129.9, 128.3, 119.0, 52.6, 31.6, 29.4, 21.1.

HRMS (APCI) m/z: [M+H]$^+$ Calculated for [$C_{16}H_{15}O_3N_2$]$^+$ 283.1077; found 283.1070.

mp: 88-92 °C.

(2-(pyrimidin-2-yl)cyclopropyl)(thiophen-2-yl)methanone (3g)

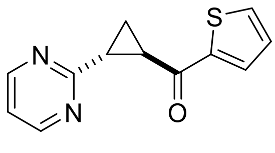

Prepared according to general procedure B (0.2 mmol scale, column conditions 0-70% EtOAc in hexane). The pure product was afforded as an off-white solid (29 mg, 41% yield).

^{1}H NMR (400 MHz, CDCl$_3$) δ 8.62 (d, *J* = 4.9 Hz, 2H), 7.84 (dd, *J* = 3.8, 1.1 Hz, 1H), 7.64 (dd, *J* = 5.0, 1.1 Hz, 1H), 7.14 – 7.11 (m, 2H), 3.22 (ddd, *J* = 8.3, 5.5, 3.8 Hz, 1H), 3.02 (ddd, *J* = 8.8, 6.1, 3.8 Hz, 1H), 1.92 (ddd, *J* = 8.8, 5.5, 3.4 Hz, 1H), 1.80 (ddd, *J* = 8.3, 6.1, 3.4 Hz, 1H).

^{13}C NMR (100 MHz, CDCl$_3$) δ 190.4, 169.2, 157.1, 144.9, 133.9, 132.3, 128.3, 118.9, 30.8, 30.0, 20.3.

HRMS (APCI) m/z: [M+H]$^+$ Calculated for [$C_{12}H_{11}ON_2S$]$^+$ 231.0587; found 231.0577.

mp: 100-103 °C.

1-(1-methyl-2-(pyrimidin-2-yl)cyclopropyl)ethan-1-one (3h)

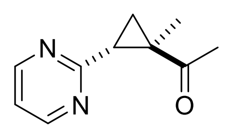

Prepared according to general procedure B (0.2 mmol scale, column conditions 0-80% EtOAc in hexane). The pure product was afforded as a yellow oil (24 mg, 68% yield).

^{1}H NMR (400 MHz, CDCl$_3$) δ 8.65 (d, *J* = 4.9 Hz, 2H), 7.13 (t, *J* = 4.9 Hz, 1H), 3.00 (t, *J* = 7.8 Hz, 1H), 2.23 (s, 3H), 1.78 (d, *J* = 7.8 Hz, 2H), 1.29 (s, 3H).

^{13}C NMR (100 MHz, CDCl$_3$) δ 208.2, 167.3, 156.7, 118.7, 35.8, 35.3, 26.8, 21.9, 13.5.

HRMS (APCI) m/z: [M+H]$^+$ Calculated for $[C_{10}H_{13}ON_2]^+$ 177.1022; found 177.1017.

(1-methyl-2-(pyrimidin-2-yl)cyclopropyl)(phenyl)methanone (3i)

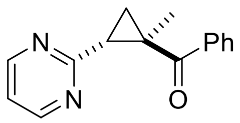

Prepared according to general procedure B (0.2 mmol scale, column conditions 0-60% EtOAc in hexane). The pure product was afforded as a yellow oil (13 mg, 27% yield).

^{1}H NMR (400 MHz, CDCl$_3$) δ 8.73 (d, *J* = 4.9 Hz, 2H), 7.90 – 7.88 (m, 2H), 7.53 – 7.49 (m, 1H), 7.46 – 7.42 (m, 2H), 7.18 (t, *J* = 4.9 Hz, 1H), 2.92 (dd, *J* = 8.7, 6.5 Hz, 1H), 1.98 (dd, *J* = 8.8, 4.5 Hz, 1H), 1.81 (dd, *J* = 6.5, 4.5 Hz, 1H), 1.33 (s, 3H).

^{13}C NMR (100 MHz, CDCl$_3$) δ 202.4, 167.5, 156.9, 136.8, 132.2, 128.9, 128.5, 118.7, 34.8, 32.3, 18.17, 15.7.

HRMS (APCI) m/z: [M+H]$^+$ Calculated for $[C_{15}H_{15}ON_2]^+$ 239.1179; found 239.1170.

2-(pyrimidin-2-yl)-3',4'-dihydro-1'H-spiro[cyclopropane-1,2'-naphthalen]-1'-one (3j)

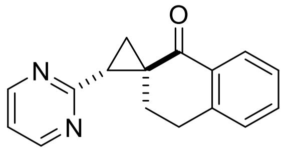

Prepared according to general procedure B (0.2 mmol scale, column conditions 0-80% EtOAc in hexane). The pure product was afforded as a yellow oil (17 mg, 34% yield).

^{1}H NMR (400 MHz, CDCl$_3$) δ 8.66 (d, *J* = 4.9 Hz, 2H), 8.04 (dd, *J* = 7.9, 1.4 Hz, 1H), 7.44 (td, *J* = 7.5, 1.5 Hz, 1H), 7.31 (t, *J* = 7.6 Hz, 1H), 7.18 – 7.13 (m, 2H), 3.17 (dd, *J* = 8.7, 6.8 Hz, 1H), 2.79 (ddd, *J* = 15.9, 7.3, 4.3 Hz, 1H), 2.64 (ddd, *J* = 15.9, 9.1, 4.3 Hz, 1H), 2.25 (ddd, *J* = 13.5, 9.1, 4.3 Hz, 1H), 2.07 – 2.00 (m, 2H), 1.91 (dd, *J* = 6.8, 3.7 Hz, 1H).

^{13}C NMR (100 MHz, CDCl$_3$) δ 196.8, 167.6, 156.7, 144.3, 133.4, 132.8, 128.6, 127.5, 126.8, 118.7, 36.7, 35.5, 28.5, 25.1, 21.1.

HRMS (APCI) m/z: [M+H]$^+$ Calculated for $[C_{16}H_{15}ON_2]^+$ 251.1179; found 251.1172.

diethyl 2-(pyrimidin-2-yl)cyclopropane-1,1-dicarboxylate (3k)

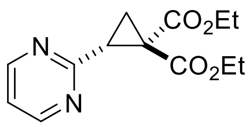

Prepared according to general procedure B (0.1 mmol scale, column conditions 20-100% EtOAc in hexane). The pure product was afforded as a yellow amorphous solid (14 mg, 54% yield).

^{1}H NMR (400 MHz, CDCl$_3$) δ 8.60 (d, *J* = 4.8 Hz, 2H), 7.12 (t, *J* = 4.9 Hz, 1H), 4.22 (qd, *J* = 7.1, 4.4 Hz, 2H), 4.06 (dq, *J* = 10.7, 7.1 Hz, 1H), 3.99 – 3.91 (m, 1H), 3.27 (dd, *J* = 8.9, 7.2 Hz, 1H), 2.32 (dd, *J* = 7.2, 4.5 Hz, 1H), 1.27 (t, *J* = 7.2 Hz, 5H), 1.03 (t, *J* = 7.1 Hz, 3H).

^{13}C NMR (100 MHz, CDCl$_3$) δ 169.4, 166.7, 166.4, 156.8, 119.1, 62.1, 61.3, 39.0, 33.5, 20.9, 14.2, 14.0.

HRMS (ESI) m/z: [M+Na]$^+$ Calculated for [$C_{13}H_{16}O_4N_2Na$]$^+$ 287.1002; found 287.0993.

(2-(benzo[d]thiazol-2-yl)cyclopropyl)(phenyl)methanone (3l)

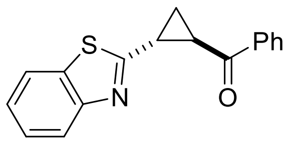

Prepared according to general procedure B (0.2 mmol scale, column conditions 0-10% EtOAc in hexane). The pure product was afforded as a yellow oil (18 mg, 32% yield).

^{1}H NMR (500 MHz, CDCl$_3$) δ 8.07 – 8.05 (m, 2H), 7.95 (dt, *J* = 8.2, 0.9 Hz, 1H), 7.83 (dt, *J* = 8.0, 0.9 Hz, 1H), 7.60 – 7.57 (m, 1H), 7.48 (dtd, *J* = 8.2, 7.1, 1.5 Hz, 3H), 7.36 (ddd, *J* = 8.2, 7.3, 1.2 Hz, 1H), 3.52 (ddd, *J* = 8.3, 5.8, 3.9 Hz, 1H), 3.09 (ddd, *J* = 8.7, 6.2, 3.9 Hz, 1H), 2.04 – 1.99 (m, 2H).

^{13}C NMR (126 MHz, CDCl$_3$) δ 197.4, 170.4, 153.2, 137.3, 134.7, 133.5, 128.8, 128.5, 126.4, 125.0, 122.6, 121.6, 29.8, 26.9, 21.0.

HRMS (APCI) m/z: [M+H]$^+$ Calculated for [$C_{17}H_{14}ONS$]$^+$ 280.0791; found 280.0797.

1-(2-(benzo[d]thiazol-2-yl)-1-methylcyclopropyl)ethan-1-one (3m)

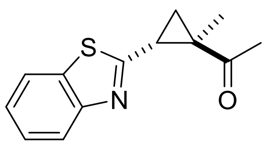

Prepared according to general procedure B (0.2 mmol scale, column conditions 0-20% EtOAc in hexane). The pure product was afforded as a yellow oil (16 mg, 35% yield).

^{1}H NMR (400 MHz, CDCl$_3$) δ 7.97 (d, *J* = 8.0 Hz, 1H), 7.84 (d, *J* = 7.9 Hz, 1H), 7.47 (ddd, *J* = 8.3, 7.2, 1.3 Hz, 1H), 7.37 (ddd, *J* = 8.2, 7.2, 1.2 Hz, 1H), 3.08 (dd, *J* = 8.9, 6.7 Hz, 1H), 2.31 (s, 3H), 1.88 (dd, *J* = 8.9, 4.3 Hz, 1H), 1.75 (dd, *J* = 6.7, 4.3 Hz, 1H), 1.38 (s, 3H).

^{13}C NMR (100 MHz, CDCl$_3$) δ 208.2, 167.9, 153.2, 135.3, 126.3, 125.1, 122.9, 121.6, 35.5, 30.8, 27.7, 23.7, 14.5.

HRMS (APCI) m/z: [M+H]$^+$ Calculated for [$C_{13}H_{14}ONS$]$^+$ 232.0791; found 232.0784.

2-(6-methylbenzo[d]thiazol-2-yl)cyclopropyl)(phenyl)methanone (3n)

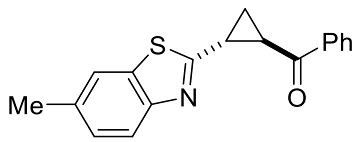

Prepared according to general procedure B (0.2 mmol scale, column conditions 0-10% EtOAc in hexane). The pure product was afforded as a light yellow oil (26.0 mg, 44% yield).

^{1}H NMR (400 MHz, CDCl$_3$) δ 8.06 – 8.04 (m, 2H), 7.82 (d, *J* = 8.3 Hz, 1H), 7.62 – 7.56 (m, 2H), 7.48 (t, *J* = 7.7 Hz, 2H), 7.27 (dd, *J* = 8.0, 1.6 Hz, 1H), 3.49 (ddd, *J* = 8.2, 5.8, 3.9 Hz, 1H), 3.06 (ddd, *J* = 8.7, 6.3, 3.9 Hz, 1H), 2.48 (s, 3H), 1.99 (tdd, *J* = 8.6, 6.0, 3.8 Hz, 2H),

^{13}C NMR (100 MHz, CDCl$_3$) δ 197.4, 169.4, 151.1, 137.3, 135.2, 134.7, 133.5, 128.8, 128.5, 128.0, 122.0, 121.4, 29.8, 26.8, 21.6, 21.0.

HRMS (APCI) m/z: [M+H]$^+$ Calculated for [$C_{18}H_{16}ONS$]$^+$ 294.0947; found 294.0945.

2-(5-bromobenzo[d]thiazol-2-yl)cyclopropyl)(phenyl)methanone (3o)

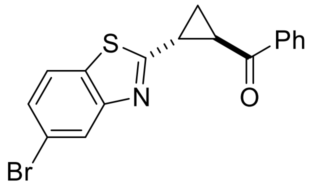

Prepared according to general procedure B (0.2 mmol scale, column conditions 0-10% EtOAc in hexane). The pure product was afforded as a light yellow solid (43.4 mg, 61% yield).

^{1}H NMR (400 MHz, CDCl$_3$) δ 8.00 – 7.96 (m, 3H), 7.58 (d, *J* = 8.5 Hz, 1H), 7.53 – 7.48 (m, 1H), 7.42 – 7.36 (m, 3H), 3.44 (ddd, *J* = 8.4, 5.8, 3.8 Hz, 1H), 2.99 (ddd, *J* = 8.7, 6.1, 3.9 Hz, 1H), 1.91 (ddt, *J* = 12.0, 6.1, 3.7 Hz, 2H).

^{13}C NMR (100 MHz, CDCl$_3$) δ 197.2, 172.1, 154.5, 137.2, 133.53, 133.47, 128.8, 128.5, 128.0, 125.5, 122.6, 120.0, 29.9, 26.7, 21.2.

HRMS (APCI) m/z: [M+H]$^+$ Calculated for [$C_{17}H_{13}BrONS$]$^+$ 357.9896; found 357.9894.

mp: 58-60 °C.

2-(5-fluorobenzo[d]thiazol-2-yl)cyclopropyl)(phenyl)methanone (3p)

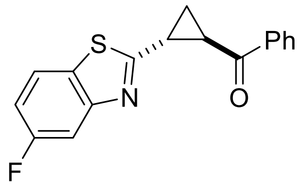

Prepared according to general procedure B (0.2 mmol scale, column conditions 0-5% EtOAc in hexane). The pure product was afforded as a light yellow solid (20.5 mg, 35% yield).

^{1}H NMR (400 MHz, CDCl$_3$) δ 8.06 – 8.04 (m, 2H), 7.87 (dd, *J* = 9.0, 4.8 Hz, 1H), 7.61 – 7.57 (m, 1H), 7.52 – 7.46 (m, 3H), 7.19 (td, *J* = 8.9, 2.6 Hz, 1H), 3.50 (ddd, *J* = 8.4, 5.8, 3.9 Hz, 1H), 3.06 (ddd, *J* = 8.7, 6.1, 3.9 Hz, 1H), 2.05 – 1.94 (m, 2H).

^{19}F NMR (376 MHz, CDCl$_3$) δ -116.55 (td, *J* = 8.5, 4.8 Hz).

^{13}C NMR (100 MHz, CDCl$_3$) δ 197.3, 170.0 (d, 3.3 Hz), 161.6, 159.2, 149.9, 137.2, 135.6 (d, 11.1 Hz), 133.5, 128.6 (d, 35.1 Hz), 123.4 (d, 9.3 Hz), 114.9 (d, 24.4 Hz), 107.9 (d, 26.7 Hz), 29.7, 26.7, 20.9.

HRMS (APCI) m/z: [M+H]$^+$ Calculated for [$C_{17}H_{13}FONS$]$^+$ 298.0688; found 298.0692.

mp: 62-64 °C.

2-(7-chlorobenzo[d]thiazol-2-yl)cyclopropyl)(phenyl)methanone (3q)

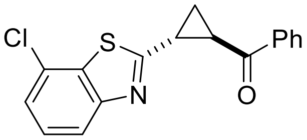

Prepared according to general procedure B (0.2 mmol scale, column conditions 0-5% EtOAc in hexane). The pure product was afforded as a yellow oil (38.0 mg, 61% yield).

^{1}H NMR (400 MHz, CDCl$_3$) δ 8.06 – 8.04 (m, 2H), 7.83 (dd, *J* = 8.0, 1.1 Hz, 1H), 7.61 – 7.57 (m, 1H), 7.48 (t, *J* = 7.7 Hz, 2H), 7.40 (t, *J* = 7.9 Hz, 1H), 7.34 (dd, *J* = 7.8, 1.0 Hz, 1H), 3.51 (ddd, *J* = 8.5, 5.7, 3.9 Hz, 1H), 3.09 (ddd, *J* = 8.7, 6.1, 3.9 Hz, 1H), 2.01 (dddd, *J* = 18.2, 8.4, 5.9, 3.8 Hz, 2H)

^{13}C NMR (100 MHz, CDCl$_3$) δ 197.1, 171.2, 153.9, 137.2, 134.9, 133.5, 128.8, 128.5, 127.3, 126.8, 124.6, 120.9, 29.9, 26.7, 21.1.

HRMS (APCI) m/z: [M+H]$^+$ Calculated for [$C_{17}H_{13}ClONS$]$^+$ 314.0401; found 314.0399.

2-(4-methylpyrimidin-2-yl)cyclopropyl)(phenyl)methanone (3r)

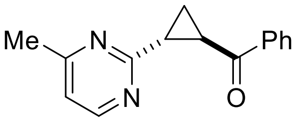

Prepared according to general procedure B (0.2 mmol scale, column conditions 0-40% EtOAc in hexane). The pure product was afforded as a light yellow oil (15.5 mg, 33% yield).

^{1}H NMR (400 MHz, CDCl$_3$) δ 8.45 (d, *J* = 5.1 Hz, 1H), 8.02 (d, *J* = 7.0 Hz, 2H), 7.55 (t, *J* = 7.3 Hz, 1H), 7.45 (t, *J* = 7.5 Hz, 2H), 6.98 (d, *J* = 5.1 Hz, 1H), 3.35 (ddd, *J* = 8.5, 5.6, 3.9 Hz, 1H), 2.96 (ddd, *J* = 9.0, 6.1, 3.9 Hz, 1H), 2.48 (s, 3H), 1.91 (ddd, *J* = 8.7, 5.5, 3.1 Hz, 1H), 1.81 (ddd, *J* = 8.3, 6.0, 3.2 Hz, 1H).

^{13}C NMR (100 MHz, CDCl$_3$) δ 198.1, 168.7 167.3, 156.6, 137.8, 133.1, 128.6, 128.4, 118.4, 31.1, 29.0, 24.3, 20.4.

HRMS (APCI) m/z: [M+H]$^+$ Calculated for [$C_{15}H_{15}ON_2$]$^+$ 239.1179; found 239.1177.

phenyl-2-(4-(trifluoromethyl)pyrimidin-2-yl)cyclopropyl)methanone (3s)

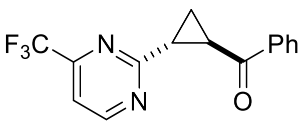

Prepared according to general procedure B (0.2 mmol scale, column conditions 0-5% EtOAc in hexane). The pure product was afforded as a light yellow solid (23.2 mg, 40% yield).

^{1}H NMR (400 MHz, CDCl$_3$) δ 8.87 (d, *J* = 5.0 Hz, 1H), 8.04 – 8.01 (m, 2H), 7.59 – 7.55 (m, 1H), 7.49 – 7.45 (m, 3H), 3.42 (ddd, *J* = 8.5, 5.6, 3.8 Hz, 1H), 3.12 (ddd, *J* = 8.8, 5.9, 3.8 Hz, 1H), 1.98 (ddd, *J* = 8.9, 5.6, 3.3 Hz, 1H), 1.87 (ddd, *J* = 8.4, 5.9, 3.3 Hz, 1H).

^{19}F NMR (376 MHz, CDCl$_3$) δ -70.11.

^{13}C NMR (100 MHz, CDCl$_3$) δ 197.5, 170.7, 159.5, 155.8 (q, 36.0 Hz), 137.5, 133.3, 128.8, 128.4, 120.5 (q, 273.6 Hz), 114.3 (q, 2.4 Hz), 30.6, 29.7, 21.1.

HRMS (APCI) m/z: [M+H]$^+$ Calculated for [$C_{15}H_{12}ONF_3$]$^+$ 293.0896; found 293.0892.

mp: 50-52 °C.

1-benzyl-2-(4-nitrophenyl)aziridine (5a)

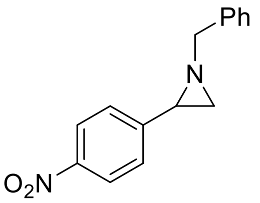

An oven-dried 2-5 mL microwave vial was charged with N-benzyl-4-nitrobenzenesulfonamide (58 mg, 0.2 mmol, 1 eq.), diphenyl(vinyl)sulfonium trifluoromethanesulfonate (108 mg, 0.3 mmol, 1.5 eq.) and K_2CO_3 (55 mg, 0.4 mmol, 2 eq.). The vial was sealed, evacuated and backfilled with nitrogen. Anhydrous DMF (2 mL, 0.1 M) was added *via* syringe and the reaction was heated to 70 °C and left to stir overnight. The reaction mixture was then diluted (EtOAc) and washed (aq. LiCl x3). The organic phase was then dried ($MgSO_4$), filtered and concentrated *in vacuo.* The crude product was purified using flash column chromatography (column conditions: 0-30% EtOAc in hexane). The pure product was afforded as a yellow oil (15 mg, 30% yield).

^{1}H NMR (400 MHz, CDCl$_3$) δ 8.19 – 8.10 (m, 2H), 7.44 – 7.39 (m, 2H), 7.37 – 7.25 (m, 5H), 3.74 (d, J = 13.7 Hz, 1H), 3.61 (d, J = 13.6 Hz, 1H), 2.58 (dd, J = 6.5, 3.2 Hz, 1H), 2.02 – 1.95 (m, 2H).

^{13}C NMR (100 MHz, CDCl$_3$) δ 148.3, 147.1, 138.7, 128.6, 127.9, 127.4, 127.1, 123.8, 64.6, 40.8, 39.3.

HRMS (APCI) m/z: [M+H]$^+$ Calculated for [$C_{15}H_{15}O_2N_2$]$^+$ 255.1128; found 255.1123.

3-(4-nitrophenyl)-5-(pyrimidin-2-yl)-1,2-oxathiolane 2-oxide (7a)

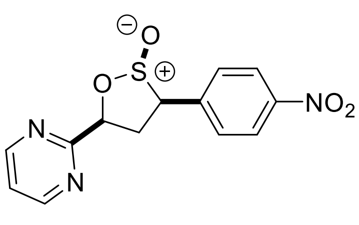

Prepared according to general procedure B (0.2 mmol scale, column conditions 0-80% EtOAc in hexane). The crude product was a 5:1 mixture of diastereoisomers (see below), from which the pure product **7a** was isolated after chromatography as an off-white solid (40 mg, 66% yield).

^{1}H NMR (400 MHz, CDCl$_3$) δ 8.86 (d, *J* = 4.9 Hz, 2H), 8.28 – 8.24 (m, 2H), 7.71 – 7.67 (m, 2H), 7.33 (t, *J* = 4.9 Hz, 1H), 5.89 (dd, *J* = 10.8, 5.8 Hz, 1H), 4.57 (dd, *J* = 13.9, 6.0 Hz, 1H), 3.55 – 3.46 (m, 1H), 3.05 (dt, *J* = 13.2, 5.9 Hz, 1H).

^{13}C NMR (100 MHz, CDCl$_3$) δ 166.0, 157.9, 148.4, 138.2, 130.3, 124.1, 120.8, 90.6, 73.3, 33.6.

HRMS (ESI) m/z: $[M+Na]^+$ Calculated for $[C_{13}H_{11}O_4N_3SNa]^+$ 328.0362; found 328.0355.

mp: 160-162 °C.

XRD Recrystallized from EtOAc/Hexane as off-white crystals. Single crystal analysis confirms the structure drawn (CCDC deposition number: 2374655).

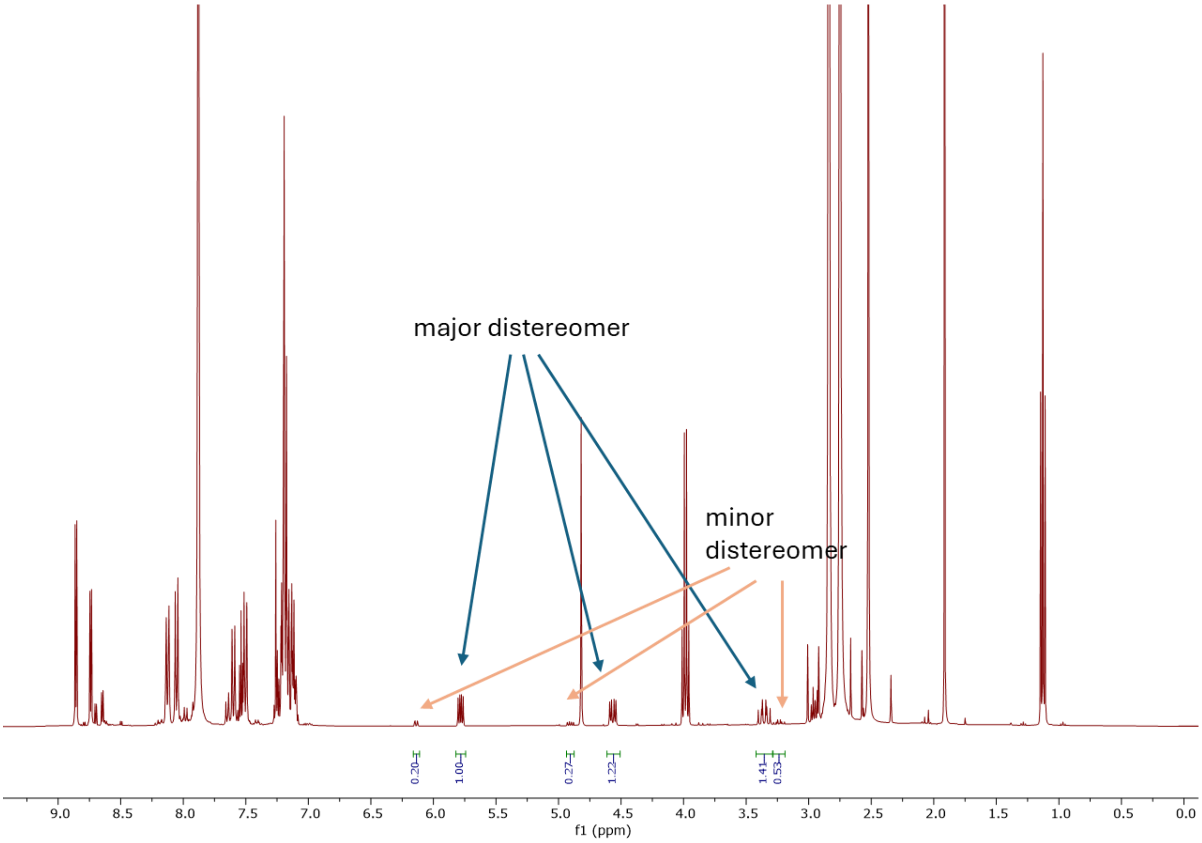

4-(2-oxido-5-(pyrimidin-2-yl)-1,2-oxathiolan-3-yl)benzonitrile (7b)

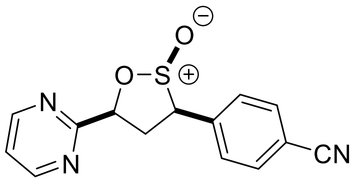

Prepared according to general procedure B (0.1 mmol scale, column conditions 0-65% EtOAc in hexane). The pure product was afforded as a light yellow solid (20.5 mg, 72% yield).

^{1}H NMR (400 MHz, CDCl$_3$) δ 8.85 (d, *J* = 4.9 Hz, 2H), 7.70 (d, *J* = 8.1 Hz, 2H), 7.63 (d, *J* = 8.0 Hz, 2H), 7.33 (t, *J* = 4.9 Hz, 1H), 5.88 (dd, *J* = 10.8, 5.9 Hz, 1H), 4.50 (dd, *J* = 14.0, 6.0 Hz, 1H), 3.48 (td, *J* = 13.6, 10.8 Hz, 1H), 3.02 (dt, *J* = 12.6, 5.9 Hz, 1H).

^{13}C NMR (100 MHz, CDCl$_3$) δ 166.0, 157.9, 136.3, 132.6, 130.1, 120.8, 118.5, 113.0, 90.5, 73.6, 33.4.

HRMS (APCI) m/z: $[M+H]^+$ Calculated for $[C_{14}H_{12}O_2N_3S]^+$ 286.0645; found 286.0642.

mp: 172-174 °C.

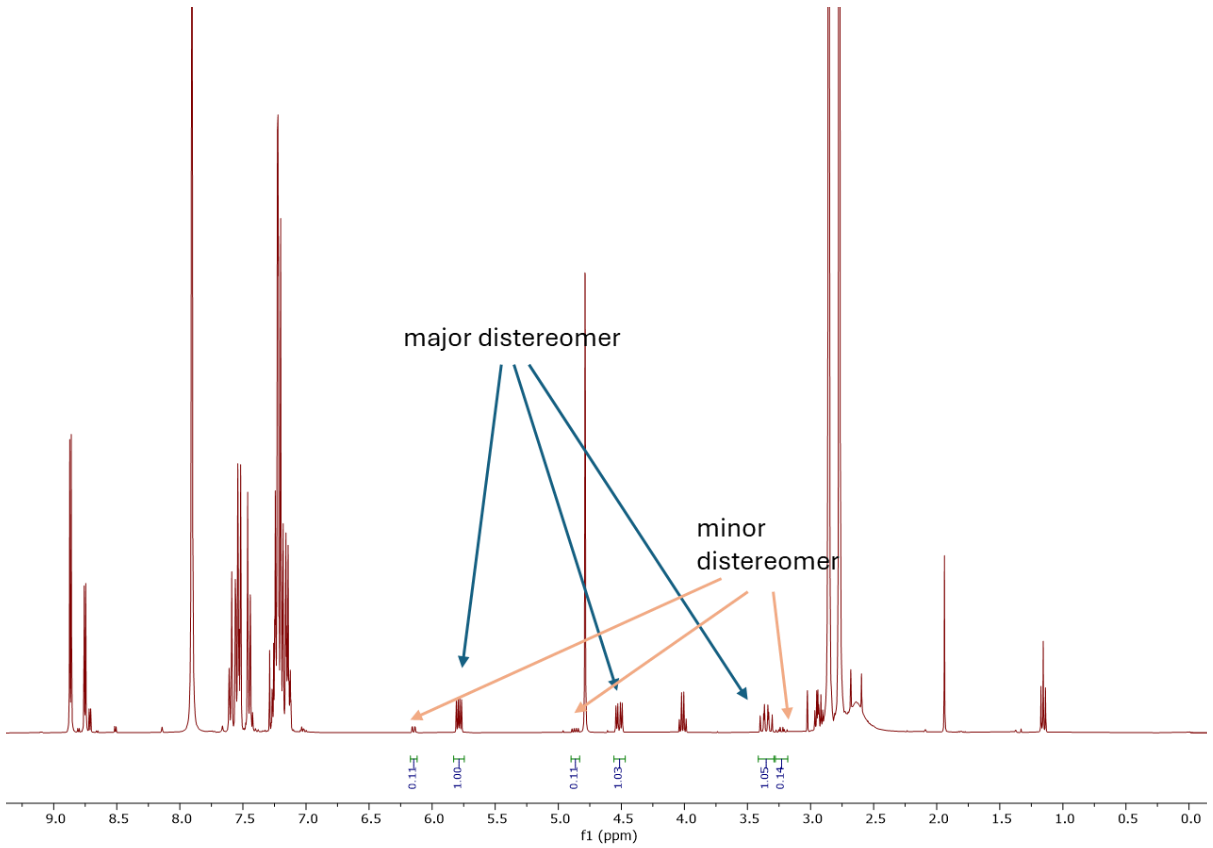

3-(3-methyl-4-nitrophenyl)-5-(pyrimidin-2-yl)-1,2-oxathiolane 2-oxide (7c)

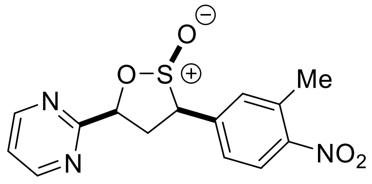

Prepared according to general procedure B (0.2 mmol scale, column conditions 0-65% EtOAc in hexane). The pure product was afforded as a light yellow solid (30.9 mg, 48% yield).

^{1}H NMR (400 MHz, CDCl$_3$) δ 8.86 (d, *J* = 4.9 Hz, 2H), 8.00 (d, *J* = 8.3 Hz, 1H), 7.47 (d, *J* = 9.2 Hz, 2H), 7.33 (t, *J* = 4.9 Hz, 1H), 5.87 (dd, *J* = 10.9, 5.8 Hz, 1H), 4.49 (dd, *J* = 14.0, 5.9 Hz, 1H), 3.48 (td, *J* = 13.6, 10.8 Hz, 1H), 3.02 (dt, *J* = 13.2, 5.9 Hz, 1H), 2.62 (s, 3H).

^{13}C NMR (100 MHz, CDCl$_3$) δ 166.1, 157.9, 149.4, 136.3, 134.4, 133.5, 127.8, 125.3, 120.8, 90.5, 73.2, 33.4, 20.7.

HRMS (APCI) m/z: $[M+H]^+$ Calculated for $[C_{14}H_{14}O_4N_3S]^+$ 320.0700; found 320.0703.

mp: 164-166 °C.

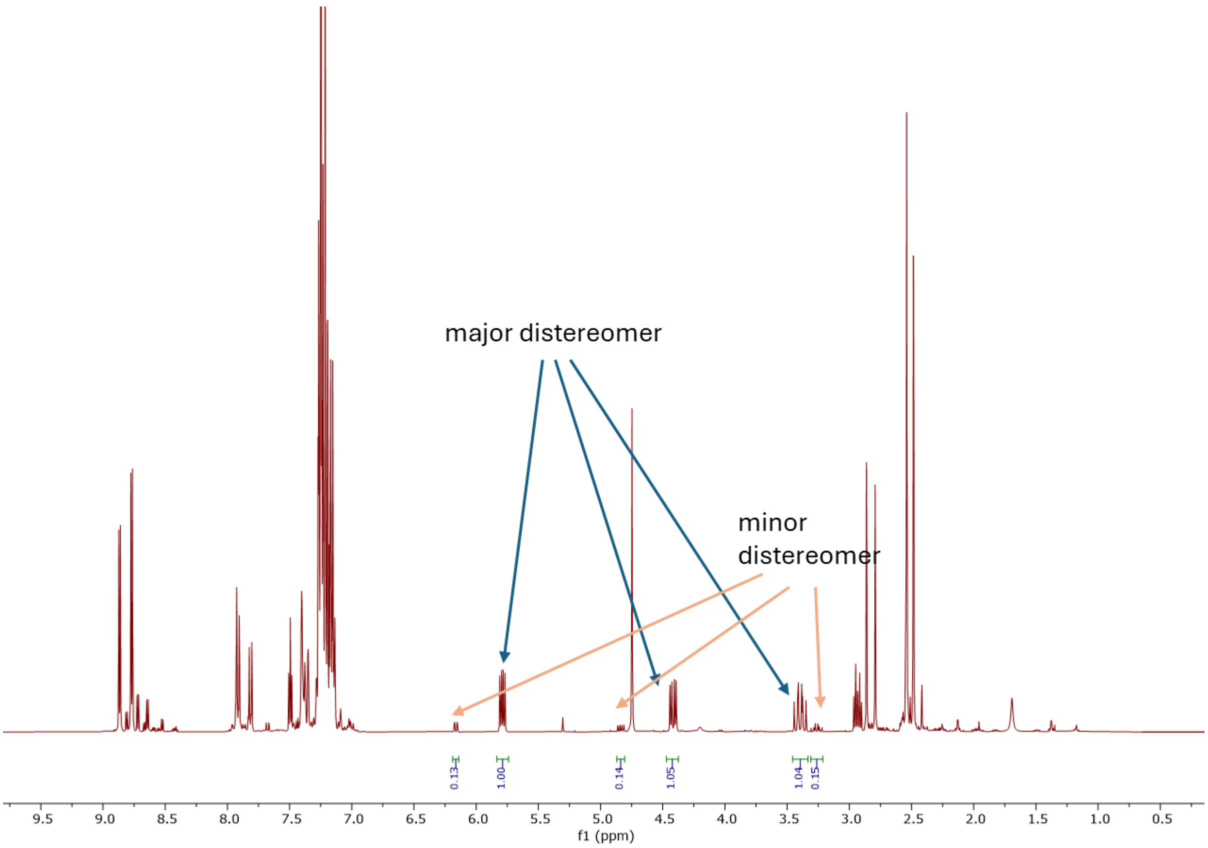

3-(2-nitrophenyl)-5-(pyrimidin-2-yl)-1,2-oxathiolane 2-oxide (7d)

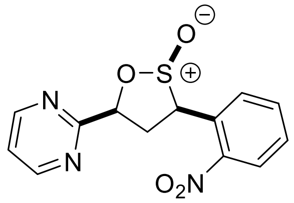

Prepared according to general procedure B (0.2 mmol scale, column conditions 0-65% EtOAc in hexane). The pure product was afforded as a light yellow solid (39.8 mg, 65% yield).

^{1}H NMR (400 MHz, CDCl$_3$) δ 8.85 (d, *J* = 4.9 Hz, 2H), 8.01 (d, *J* = 7.5 Hz, 1H), 7.83 (d, *J* = 7.9 Hz, 1H), 7.66 (t, *J* = 7.7 Hz, 1H), 7.53 (t, *J* = 7.8 Hz, 1H), 7.32 (t, *J* = 4.9 Hz, 1H), 5.82 (dd, *J* = 10.9, 5.6 Hz, 1H), 5.05 (dd, *J* = 13.9, 5.7 Hz, 1H), 3.54 (td, *J* = 13.4, 10.8 Hz, 1H), 2.93 (dt, *J* = 12.9, 5.7 Hz, 1H).

^{13}C NMR (100 MHz, CDCl$_3$) δ 166.1, 157.8, 149.8, 133.5, 130.9, 129.7, 125.9, 125.0, 120.8, 89.6, 68.3, 33.1.

HRMS (APCI) m/z: $[M+H]^+$ Calculated for $[C_{13}H_{12}O_3N_4S]^+$ 306.0543; found 306.0543.

mp: 137-140 °C.

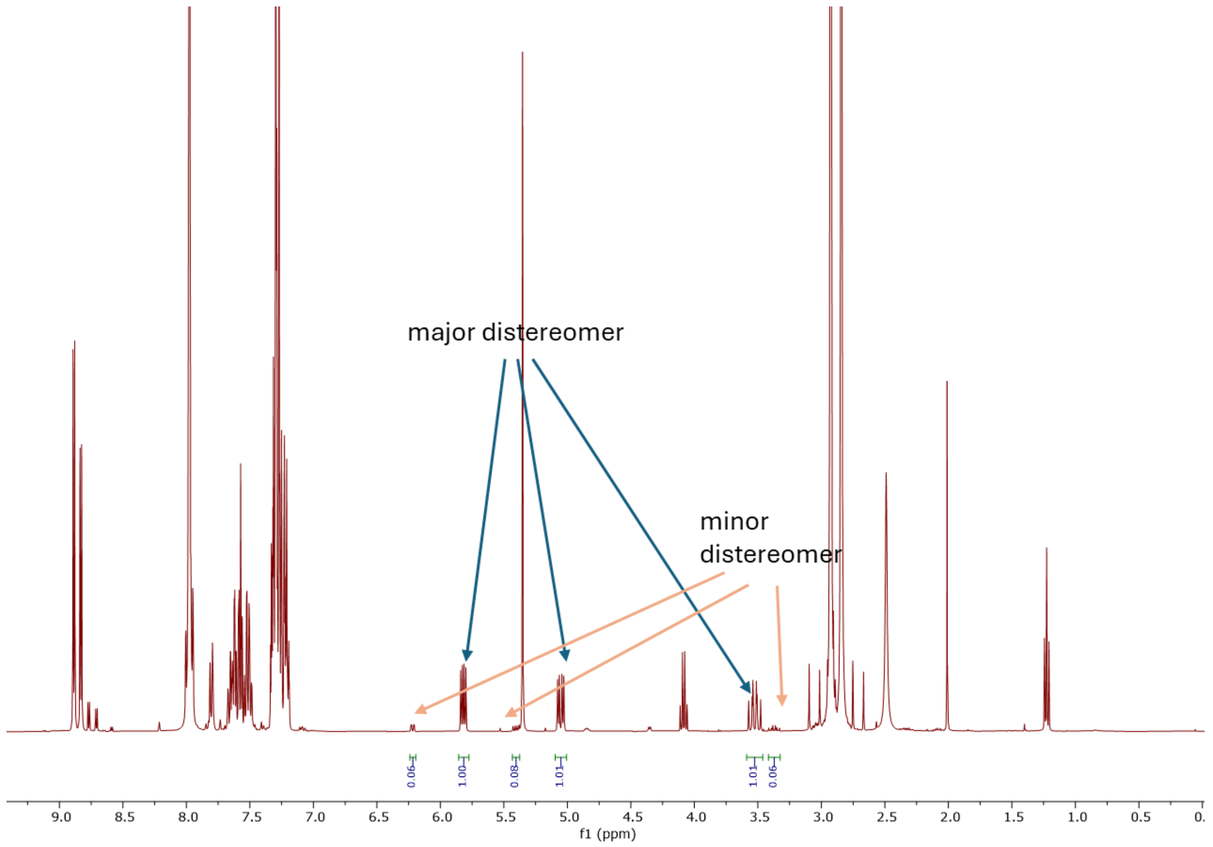

2-(1-phenylvinyl)cyclopropyl)pyrimidine (8a)

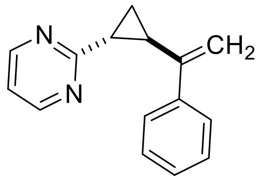

n-BuLi (1.6 M in Hexane, 0.3 mmol) was added dropwise to a suspension of methyltriphenylphosphonium bromide(0.3 mmol) in anhydrous THF (1 mL) at 0 °C and allowed to warm to room temperature and stirred for 30 min. Phenyl((1R,2R)-2-(pyrimidin-2-yl)cyclopropyl)methanone (0.2 mmol, 44.8 mg) in THF (1 mL) at 50 °C overnight. The reaction was cooled and quenched with saturated NH_4Cl, followed by extracting with Et_2O twice. The combined organic phases were dried over Na_2SO_4, filtered and concentrated under reduced pressure. The crude residue was purified by flash column chromatography to afford the corresponding product as a light yellow oil (35.2 mg, 79% yield).

^{1}H NMR (400 MHz, CDCl$_3$) δ 8.60 (d, *J* = 4.9 Hz, 2H), 7.53 – 7.50 (m, 2H), 7.32 – 7.23 (m, 3H), 7.07 (t, *J* = 4.9 Hz, 1H), 5.38 (s, 1H), 5.12 (s, 1H), 2.48 – 2.42 (m, 2H), 1.66 (ddd, *J* = 7.6, 6.2, 4.1 Hz, 1H), 1.45 – 1.41 (m, 1H).

^{13}C NMR (100 MHz, CDCl$_3$) δ 171.0, 157.0, 147.6, 141.0, 128.3, 127.7, 126.3, 118.2, 110.5, 28.9, 28.1, 18.0.

HRMS (APCI) m/z: $[M+H]^+$ Calculated for $[C_{15}H_{12}O_{15}N_2]^+$ 223.1230; found 223.1231.

3-(4-nitrophenyl)-5-(pyrimidin-2-yl)-1,2-oxathiolane 2,2-dioxide (9a)

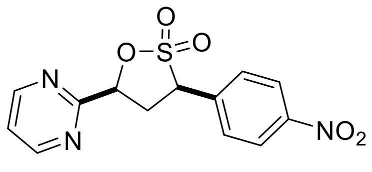

To a solution of 3-(4-nitrophenyl)-5-(pyrimidin-2-yl)-1,2-oxathiolane 2-oxide (18.3 mg, 0.06 mmol) in dichloromethane (1 mL) was added mCPBA (13.4 mg, 0.072 mmol, 1.2 equiv.) in one portion at 0 °C. and then stirred at room temperature for 24 h. The resulting mixture was washed with saturated aqueous $NaHCO_3$ solution then H_2O. The

organic phase was dried over Na2SO4, filtered and concentrated in vacuo to give a residue which was purified by flash column chromatography to give the corresponding product as a white solid (13.5 mg, 70%).

^{1}H NMR (400 MHz, CDCl$_3$) δ 8.89 (d, *J* = 4.9 Hz, 2H), 8.30 (d, *J* = 8.8 Hz, 2H), 7.77 (d, *J* = 8.8 Hz, 2H), 7.41 (t, *J* = 4.9 Hz, 1H), 5.83 (dd, *J* = 10.3, 6.3 Hz, 1H), 4.84 (dd, *J* = 12.0, 7.4 Hz, 1H), 3.49 (ddd, *J* = 13.6, 12.1, 10.3 Hz, 1H), 3.28 (ddd, *J* = 13.7, 7.5, 6.4 Hz, 1H).

^{13}C NMR (100 MHz, CDCl$_3$) δ 163.9, 158.1, 148.8, 137.1, 130.3, 124.4, 121.6, 80.0, 62.3, 35.2.

HRMS (APCI) m/z: $[M+H]^+$ Calculated for $[C_{13}H_{12}O_5N_3S]^+$ 322.0492; found 322.0491.

mp: 77-80 °C.

5. References

1) L. Kang, F. Wang, J. Zhang, H. Yang, C. Xia, J. Qian and G. Jiang, *Org. Lett.*, 2021, **23**, 1669–1674.

2) M. Nielsen, C. B. Jacobsen, M. W. Paixão, N. Holub and K. A. Jørgensen, *J. Am. Chem. Soc.*, 2009, **131**, 10581–10586.

3) S. Johnson, E. Kovács and M. F. Greaney, *Chem. Commun.*, 2020, **56**, 3222–3224.

6. X-ray crystallography

All data collections, crystal structure determinations and refinements were done by the X-ray crystallography service (George F. S. Whitehead) at The University of Manchester.

Data collection

X-ray data was collected at a temperature of 100 K on a Rigaku® FR-X DW rotating anode diffractometer using CuKα radiation, (λ = 1.54184 Å) with an AFC-11 RINC goniometer and a Rigaku Hypix 6000 HE photon counting detector. The diffractometer was equipped with an Oxford Cryosystems® Cryostream 800 plus nitrogen flow gas system.

Crystal structure determinations and refinements

X-ray data were processed and reduced using CrysAlis^{Pro} suite of programs. The crystal structures were solved and refined against all F2 values using the SHELX and Olex 2 suite of programs. All the non-hydrogen atoms were refined anisotropically. Hydrogen atoms were placed in a calculated position refined using idealised geometries (riding model) and assigned fixed isotropic displacement parameters.

Data availability

Crystallographic data for **3b** and **7a** have been deposited in the Cambridge Crystallographic Data Centre, with deposition numbers CCDC 2374654 (**3b**), 2374655 (**7a**), and are available free of charge via https://www.ccdc.cam.ac.uk/structures/.

X-Ray Structure of (4-chlorophenyl)(2-(pyrimidin-2-yl)cyclopropyl)methanone (3b)

CCDC Deposition Number: 2374654

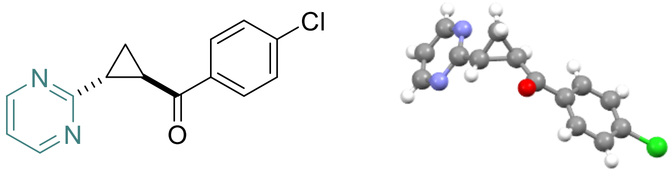

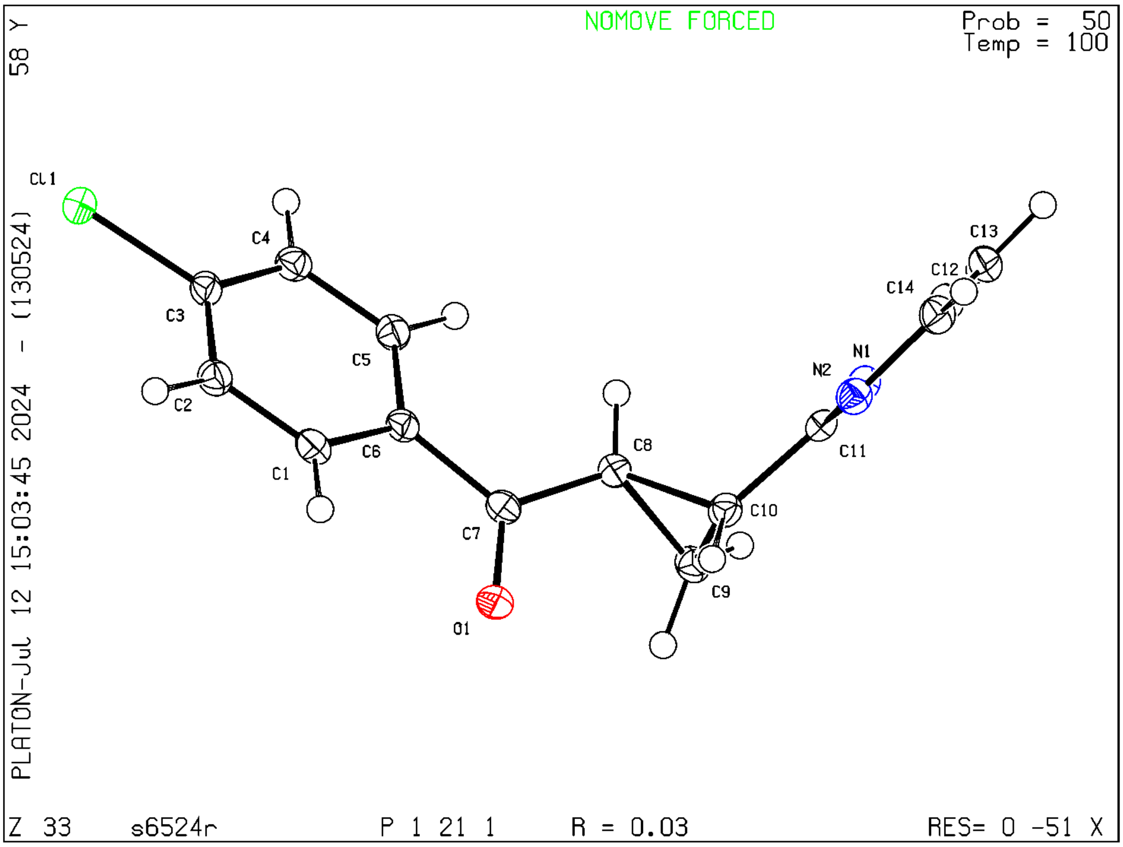

Empirical formula	$C_{14}H_{11}ClN_2O$
Formula weight	258.7
Temperature (K)	100
Space group	P 1 21 1
a (Å)	5.40193 (7)
b (Å)	9.51693 (15)
c (Å)	11.31814 (13)
α (°)	90
β (°)	93.7065 (10)
γ (°)	90
Volume (Å^3)	580.646 (13)
Z	2
P_{calc} (g/cm^3)	1.480
μ (mm^{-1})	2.809
F(000)	268.0

Radiation (Å)	CuKα (λ = 1.54184)
Independent reflections	2443
Data/parameters	2443/164
Goodness-of-fit on F^2	1.099
Final R indexes [I>=2σ (I)]	wR_2 = 0.0745
Final R indexes [all data]	R_1 = 0.0277
Ellipsoid contour probability levels (%)	50

X-Ray Structure of 3-(4-nitrophenyl)-5-(pyrimidin-2-yl)-1,2-oxathiolane 2-oxide (7a)

CCDC Deposition Number: 2374655

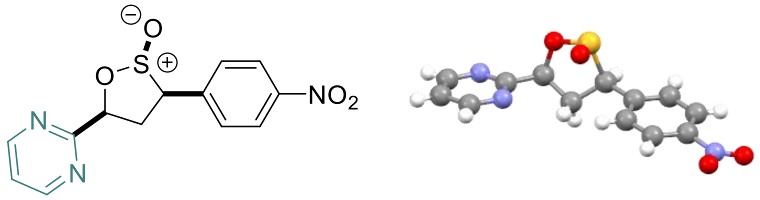

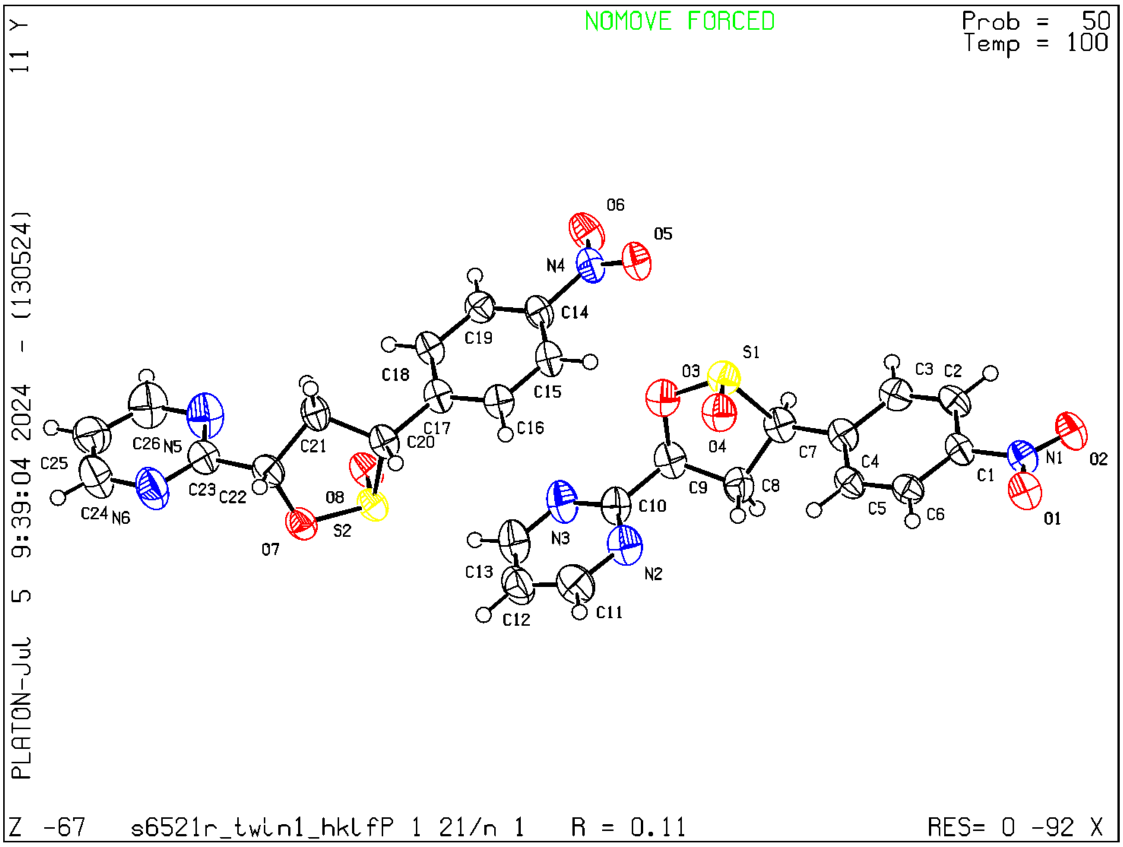

Empirical formula	$C_{13}H_{11}N_3O_4S$

Formula weight	305.31
Temperature (K)	100
Space group	P 1 21/n 1
a (Å)	22.2458 (16)
b (Å)	5.3410 (3)
c (Å)	22.5524 (18)
α (°)	90
β (°)	98.851 (7)
γ (°)	90
Volume ($Å^3$)	2647.7 (3)
Z	8
P_{calc} (g/cm^3)	1.532
μ (mm^{-1})	2.381
F(000)	1264.0
Radiation (Å)	CuKα (λ = 1.54184)
Independent reflections	10818
Data/parameters	10818/380
Goodness-of-fit on F^2	1.055
Final R indexes [I>=2σ (I)]	wR_2 = 0.3534
Final R indexes [all data]	R_1 = 0.1089
Ellipsoid contour probability levels (%)	50

7. NMR spectra

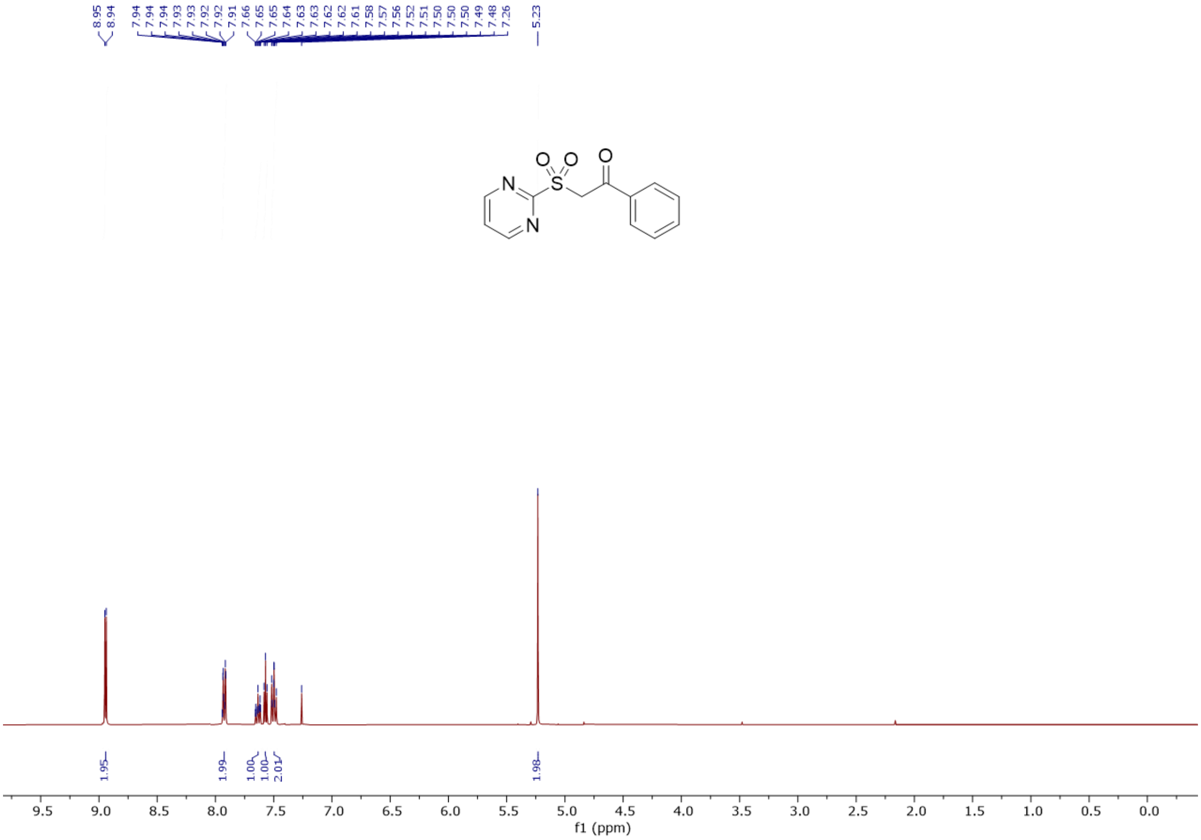

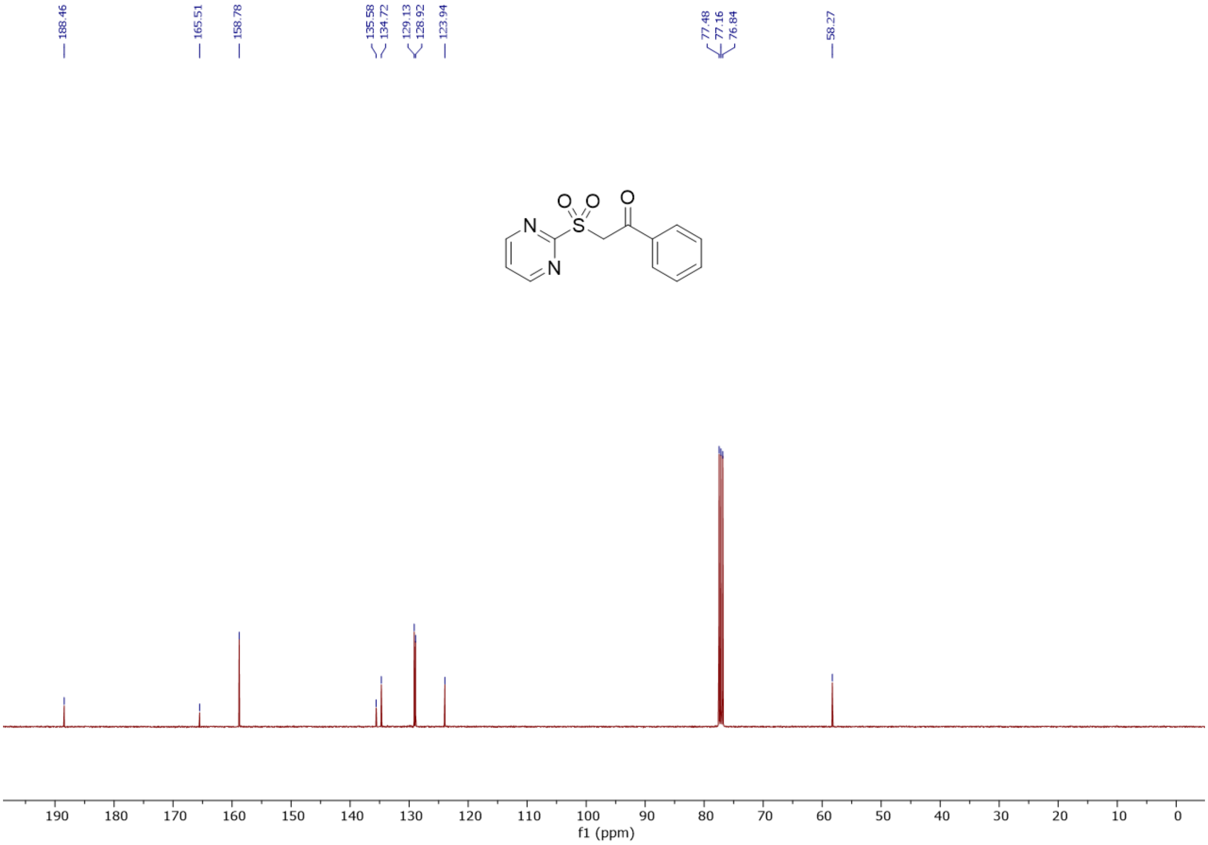

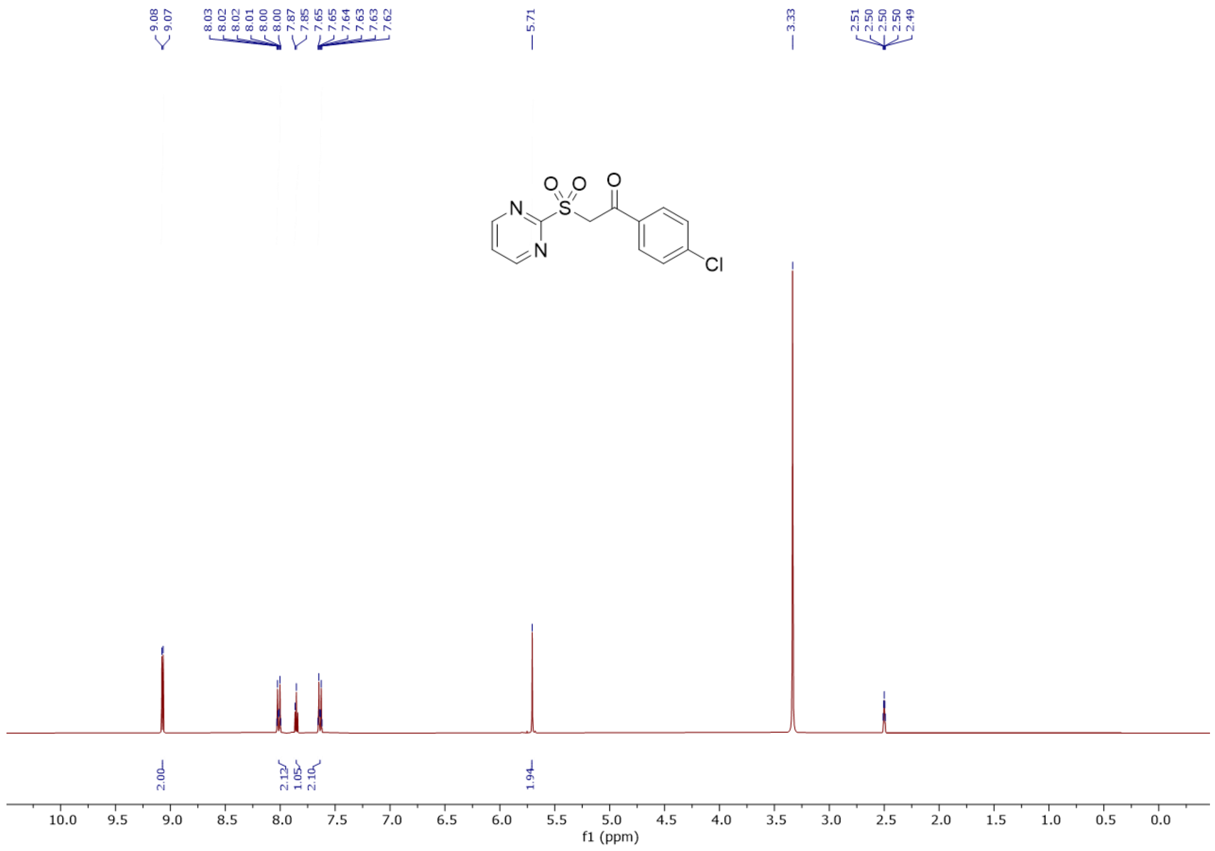

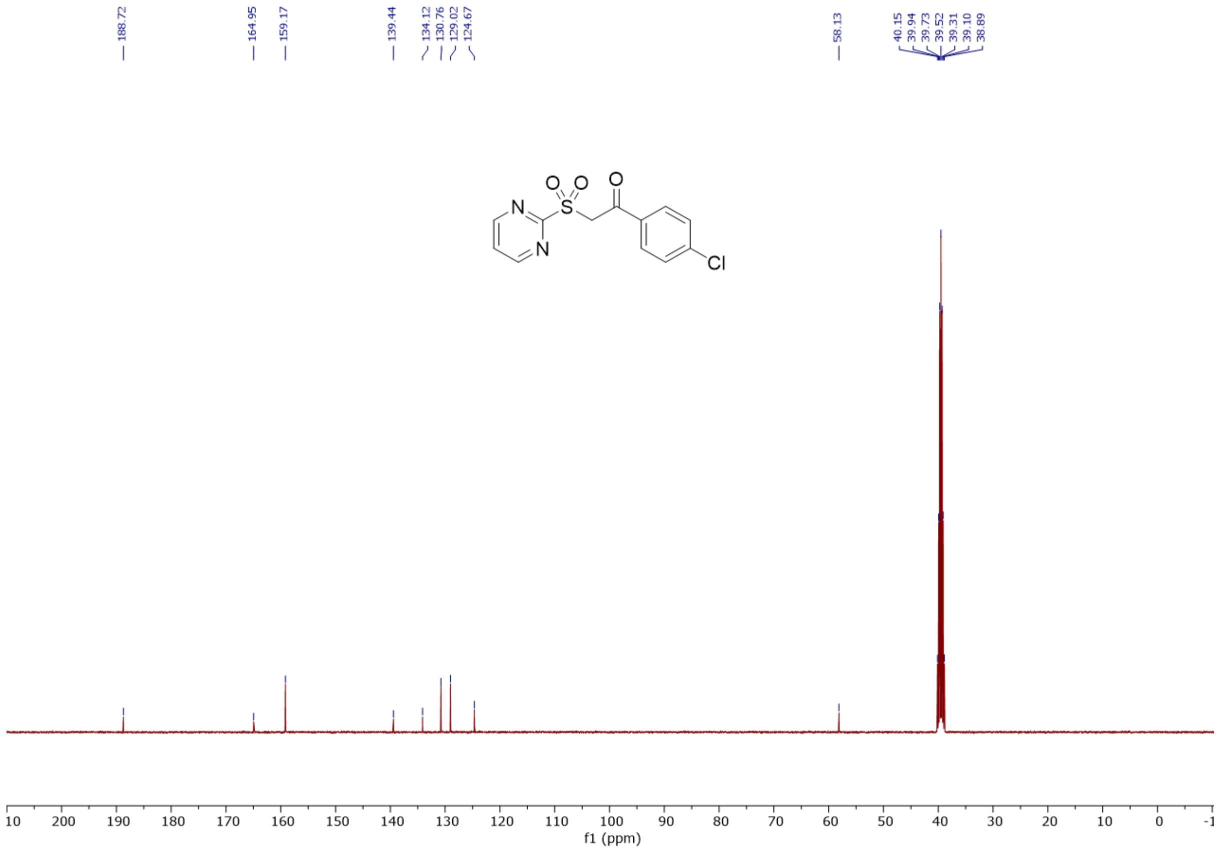

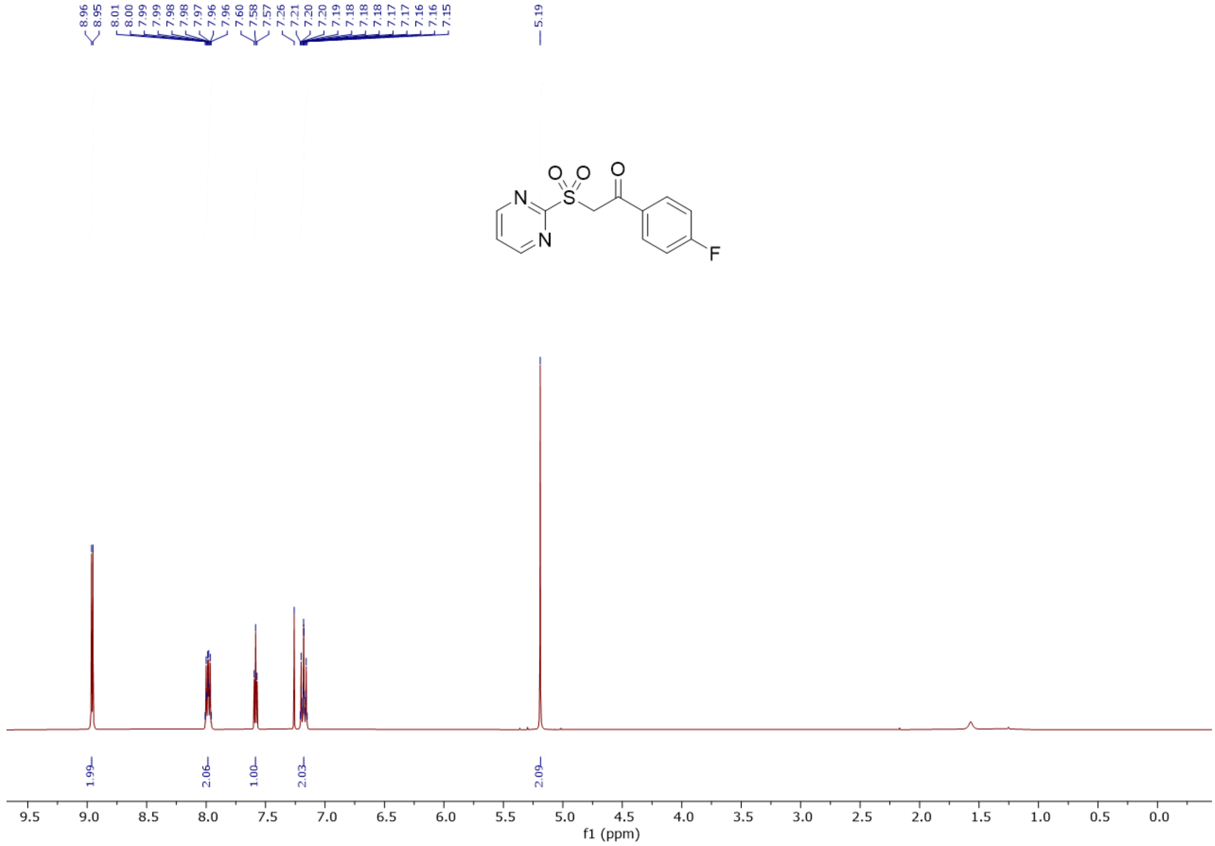

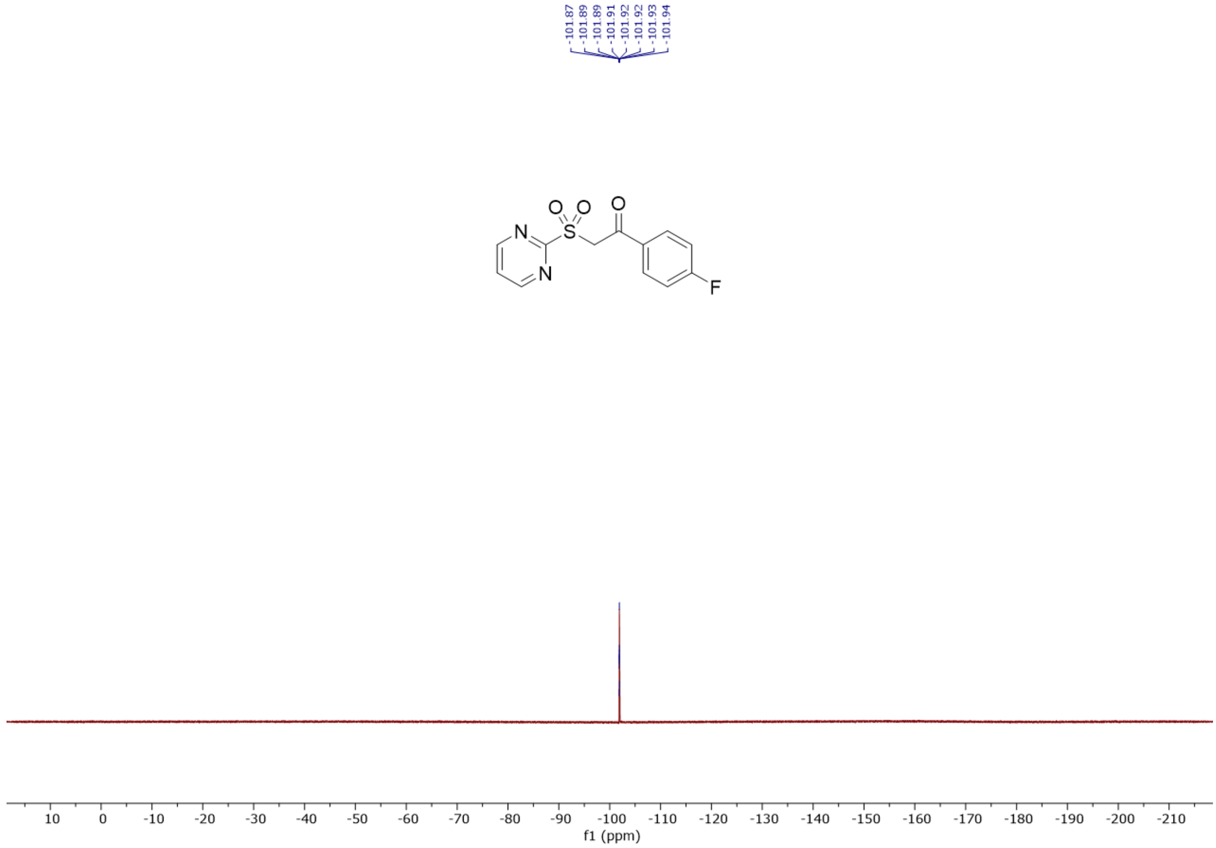

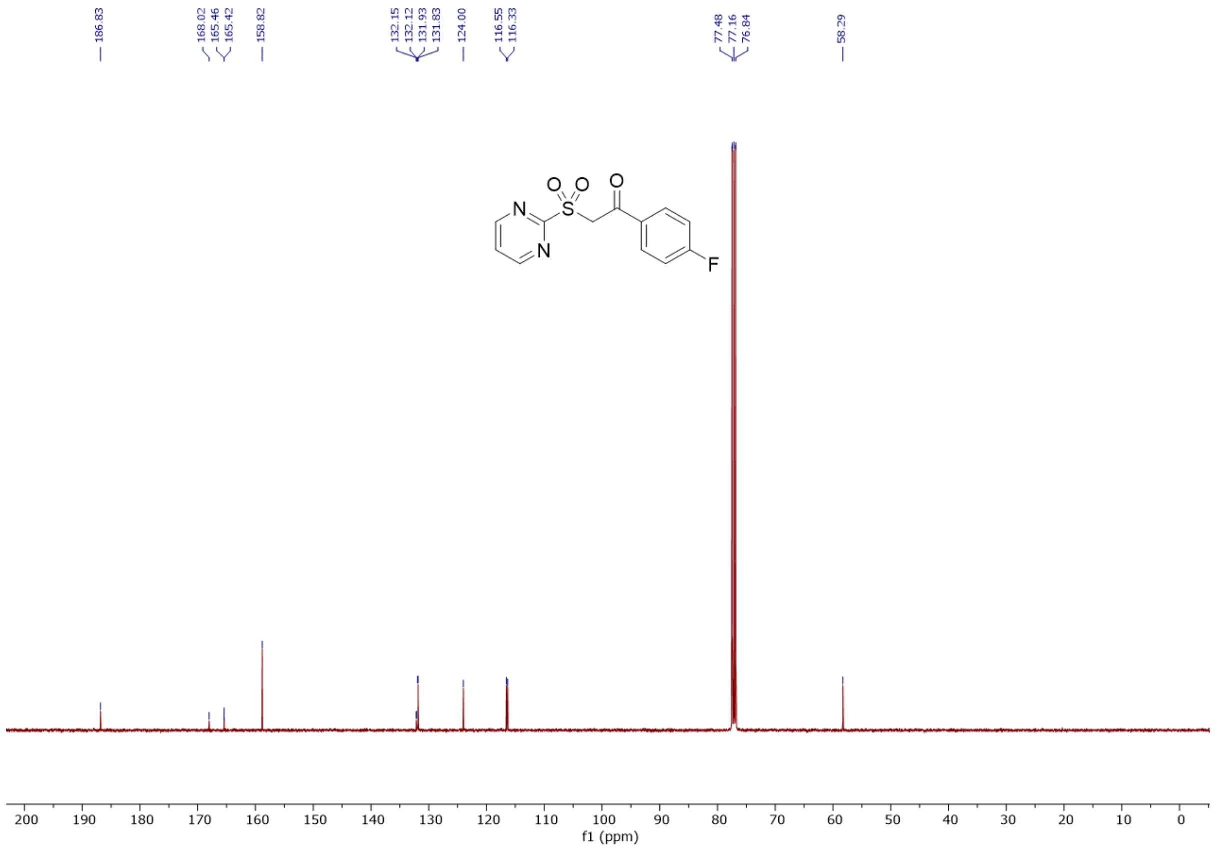
186.83
168.02
165.46
165.42
158.82
132.15
132.12
131.93
131.83
124.00
116.55
116.33
77.48
77.16
76.84
58.29
O
O
O
S
N
N
F
200 190 180 170 160 150 140 130 120 110 100 90 80 70 60 50 40 30 20 10 0
f1 (ppm)

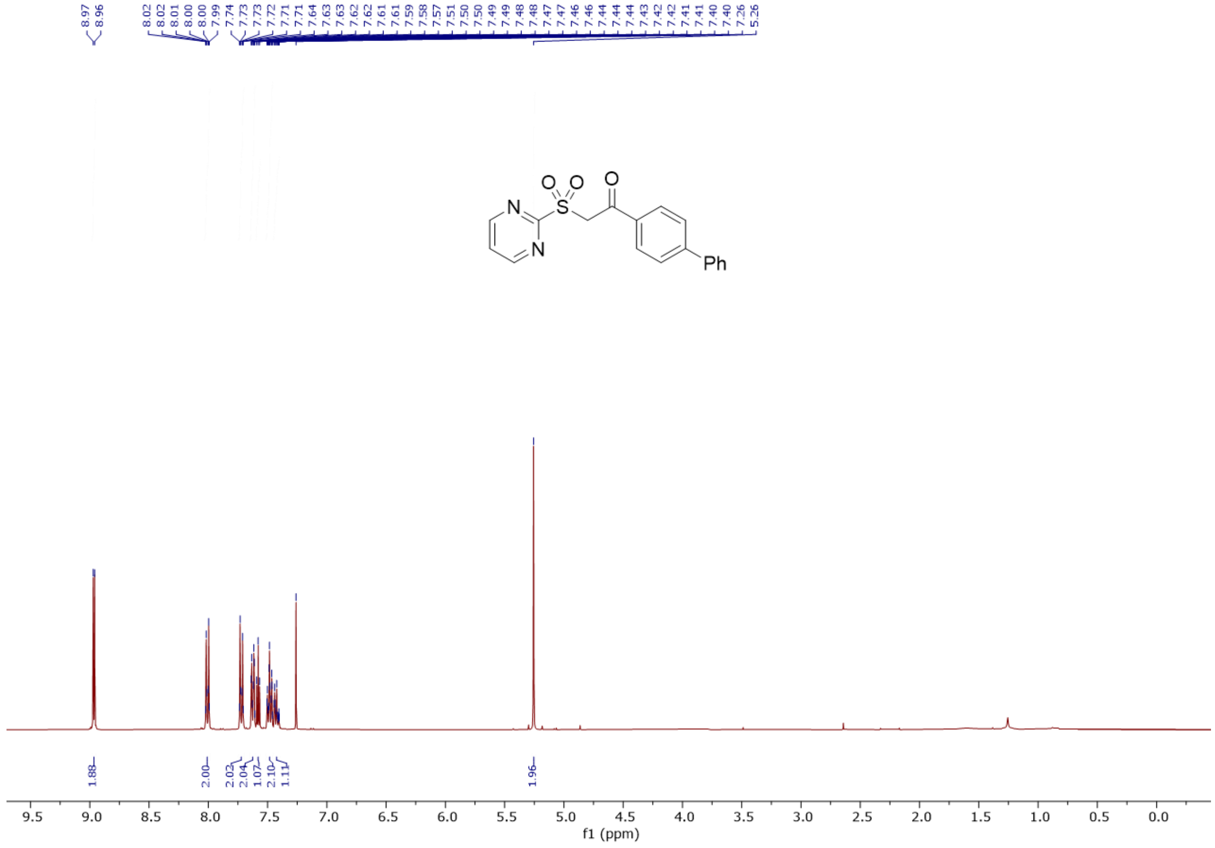
8.97
8.96
8.02
8.02
8.01
8.00
8.00
7.99
7.74
7.73
7.73
7.72
7.71
7.71
7.64
7.63
7.63
7.62
7.62
7.61
7.61
7.59
7.58
7.57
7.51
7.50
7.50
7.49
7.49
7.48
7.48
7.47
7.47
7.46
7.46
7.44
7.44
7.43
7.42
7.42
7.41
7.41
7.40
7.40
7.26
5.26
O
O
O
S
N
N
Ph
1.88
2.00
2.03
2.04
1.07
2.10
1.11
1.96
9.5 9.0 8.5 8.0 7.5 7.0 6.5 6.0 5.5 5.0 4.5 4.0 3.5 3.0 2.5 2.0 1.5 1.0 0.5 0.0
f1 (ppm)

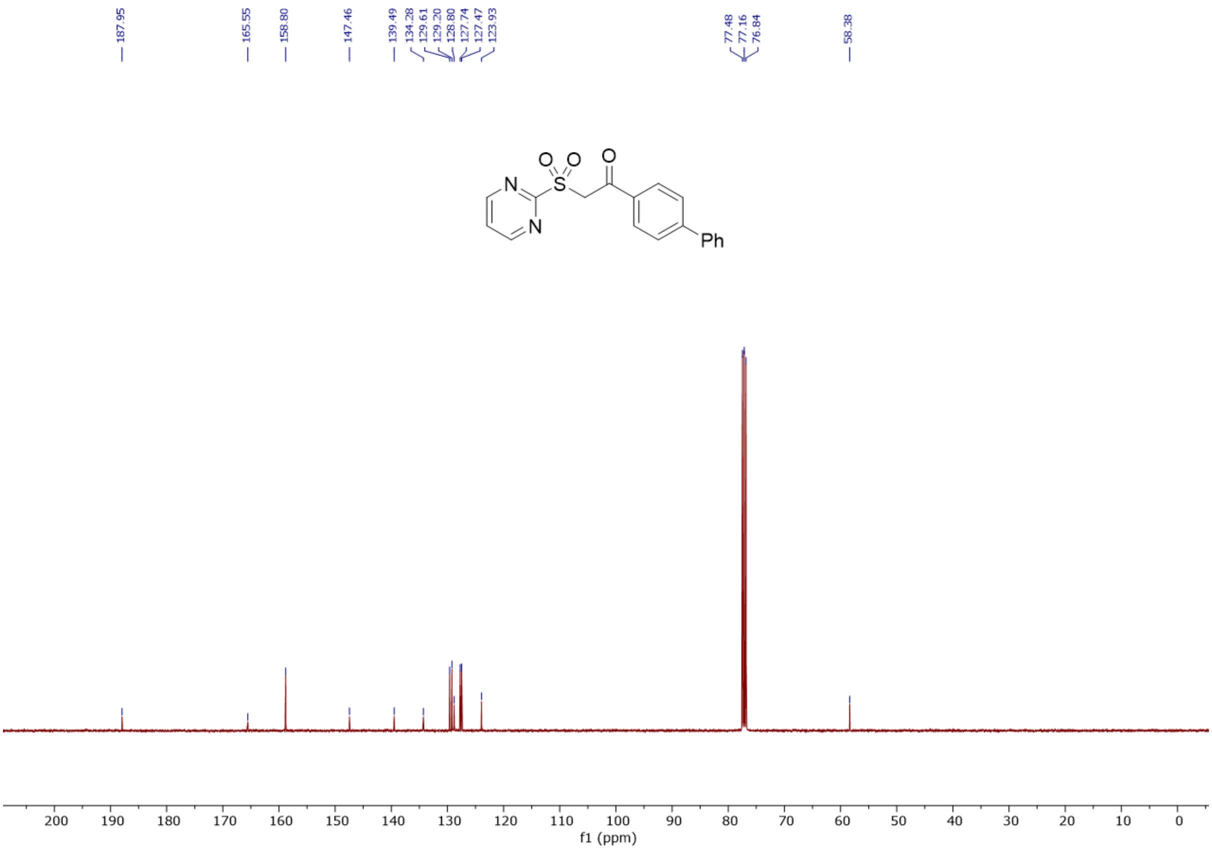
187.95
165.55
158.80
147.46
139.49
134.28
129.61
129.20
128.80
127.74
127.47
123.93
77.48
77.16
76.84
58.38
Ph
f1 (ppm)

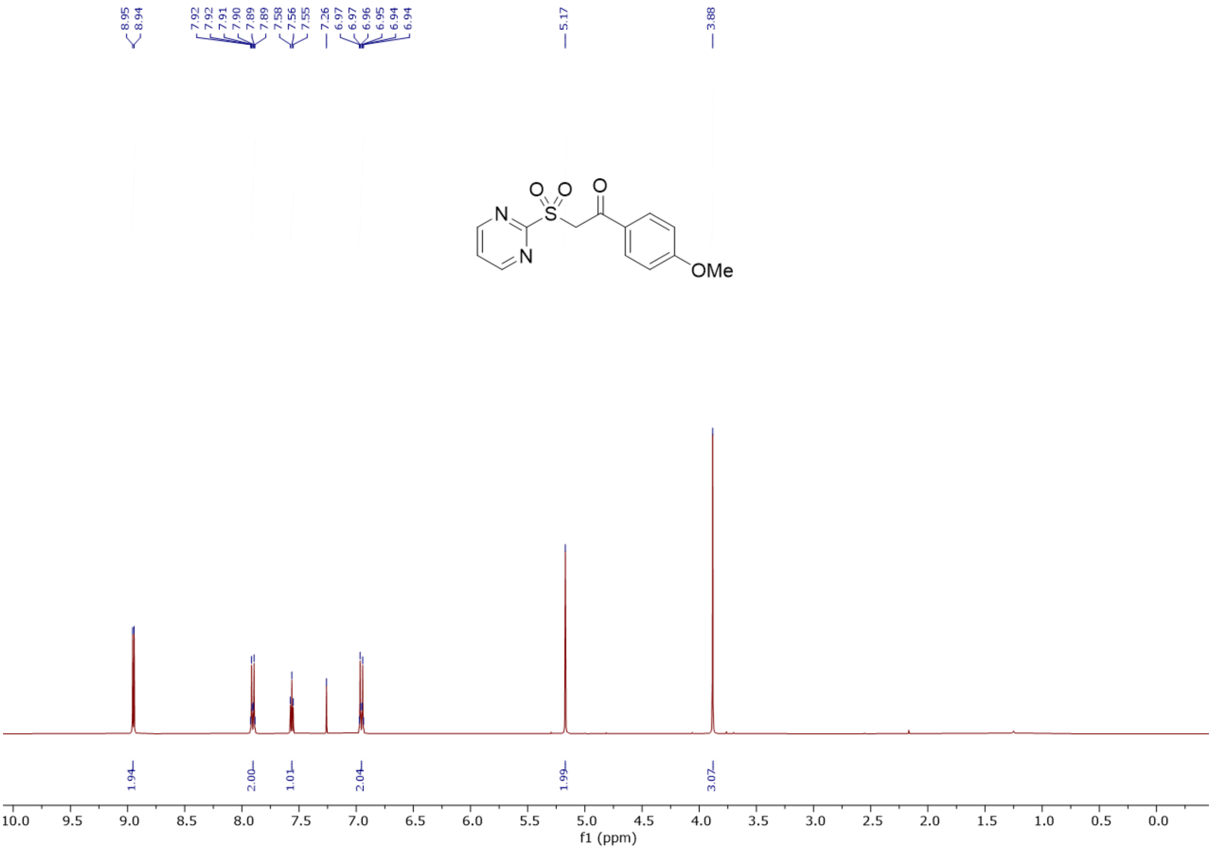
8.95
8.94
7.92
7.92
7.91
7.90
7.89
7.89
7.58
7.56
7.55
7.26
6.97
6.97
6.96
6.95
6.94
6.94
5.17
3.88
OMe
1.94
2.00
1.01
2.04
1.99
3.07
f1 (ppm)

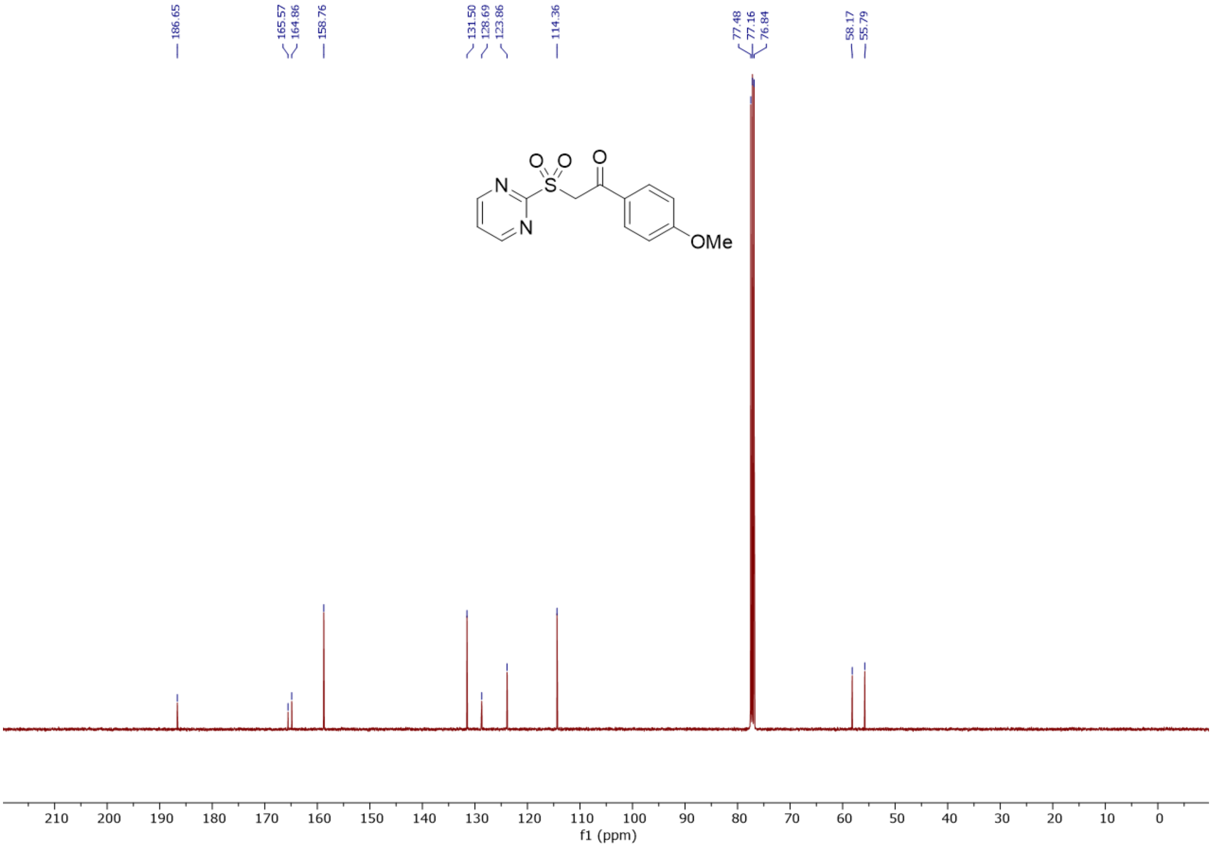
186.65
165.57
164.86
158.76
131.50
128.69
123.86
114.36
77.48
77.16
76.84
58.17
55.79
OMe
f1 (ppm)

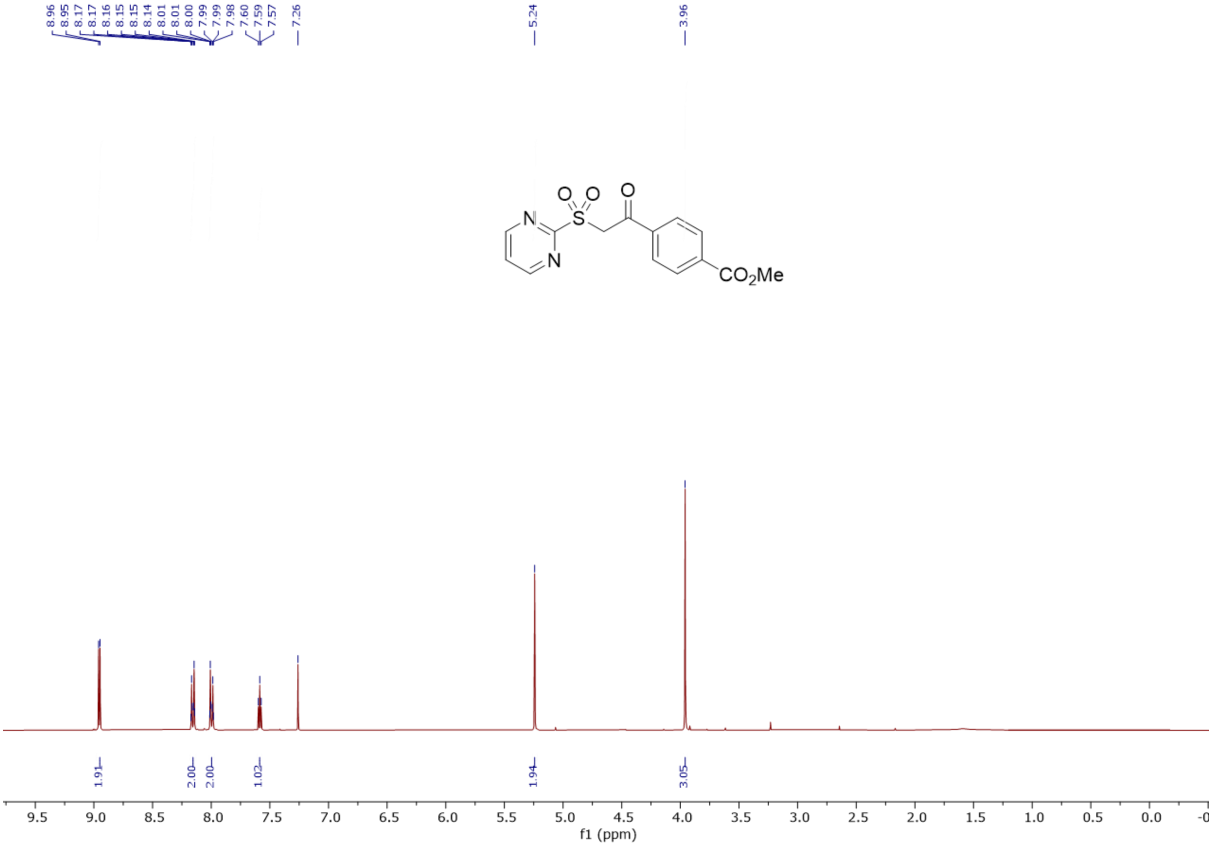
8.96
8.95
8.17
8.17
8.16
8.15
8.15
8.14
8.01
8.01
8.00
7.99
7.99
7.98
7.60
7.59
7.57
7.26
5.24
3.96
CO_2Me
1.91
2.00
2.00
1.02
1.94
3.05
f1 (ppm)

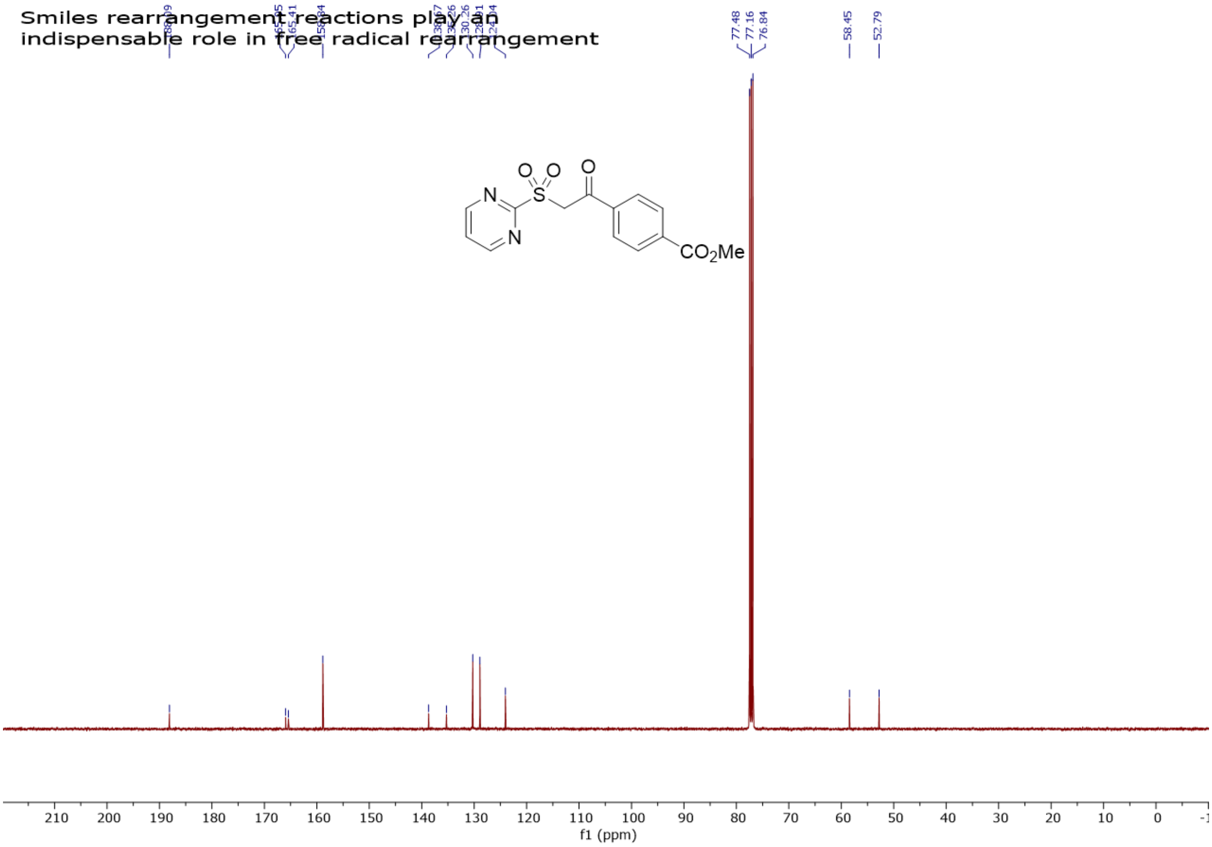
Smiles rearrangement reactions play an indispensable role in free radical rearrangement
165.41
77.48
77.16
76.84
58.45
52.79
CO2Me
210 200 190 180 170 160 150 140 130 120 110 100 90 80 70 60 50 40 30 20 10 0
f1 (ppm)

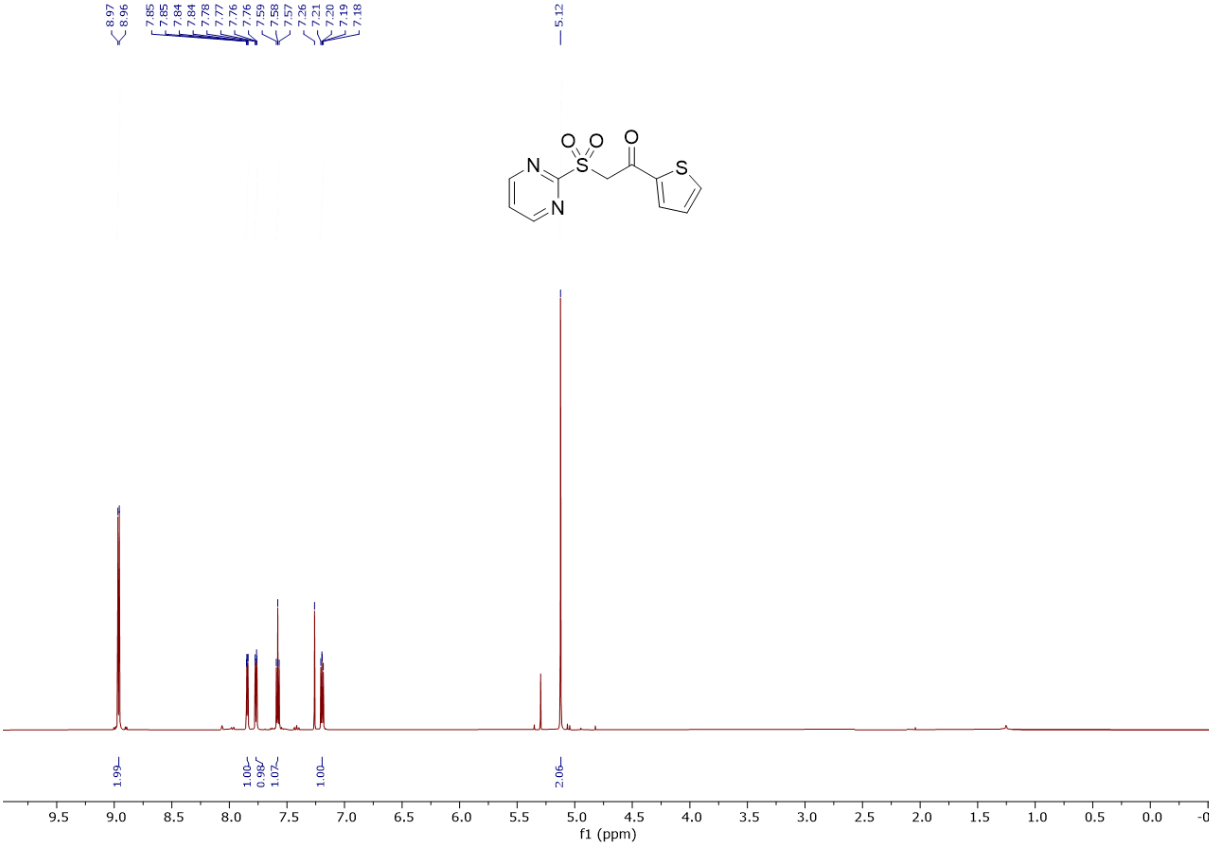
8.97
8.96
7.85
7.85
7.84
7.84
7.78
7.77
7.76
7.76
7.59
7.58
7.57
7.26
7.21
7.20
7.19
7.18
5.12
1.99
1.00
0.98
1.07
1.00
2.06
9.5 9.0 8.5 8.0 7.5 7.0 6.5 6.0 5.5 5.0 4.5 4.0 3.5 3.0 2.5 2.0 1.5 1.0 0.5 0.0
f1 (ppm)

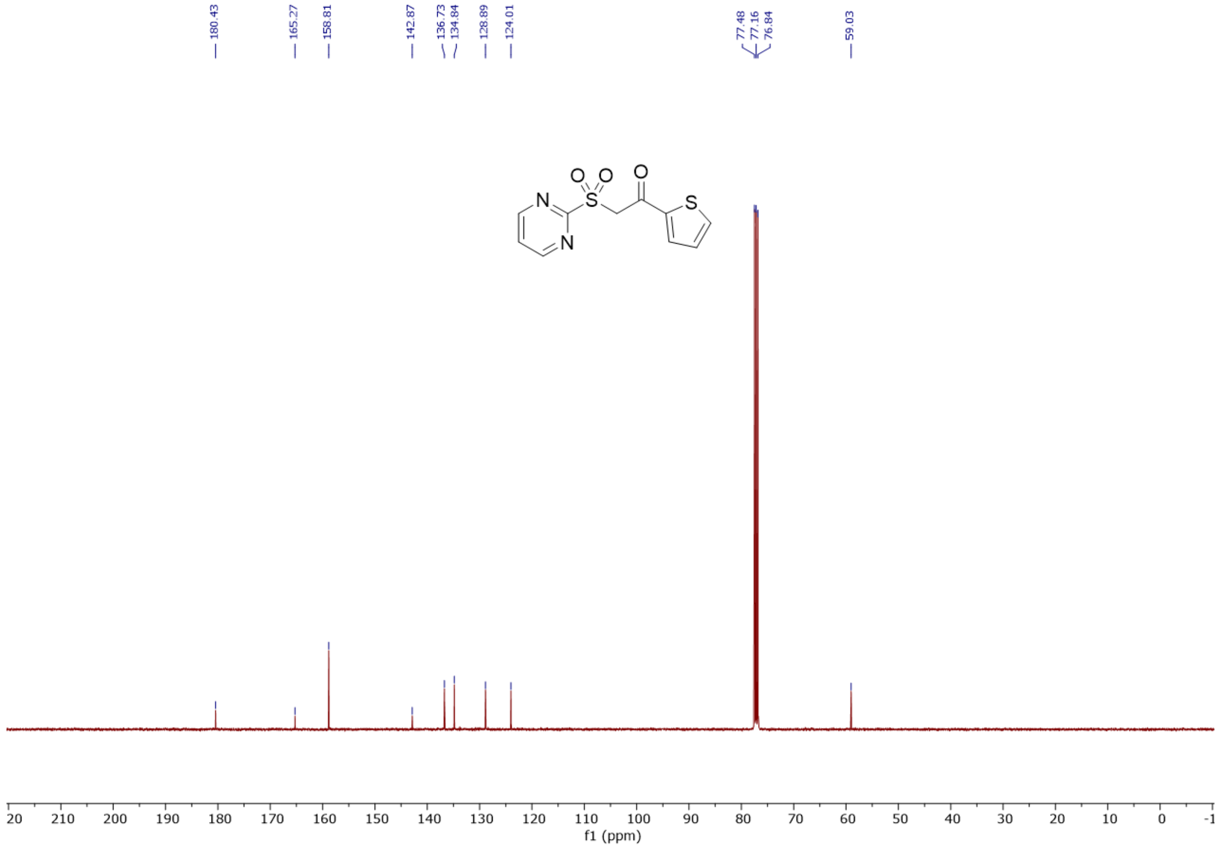
180.43
165.27
158.81
142.87
136.73
134.84
128.89
124.01
77.48
77.16
76.84
59.03
f1 (ppm)

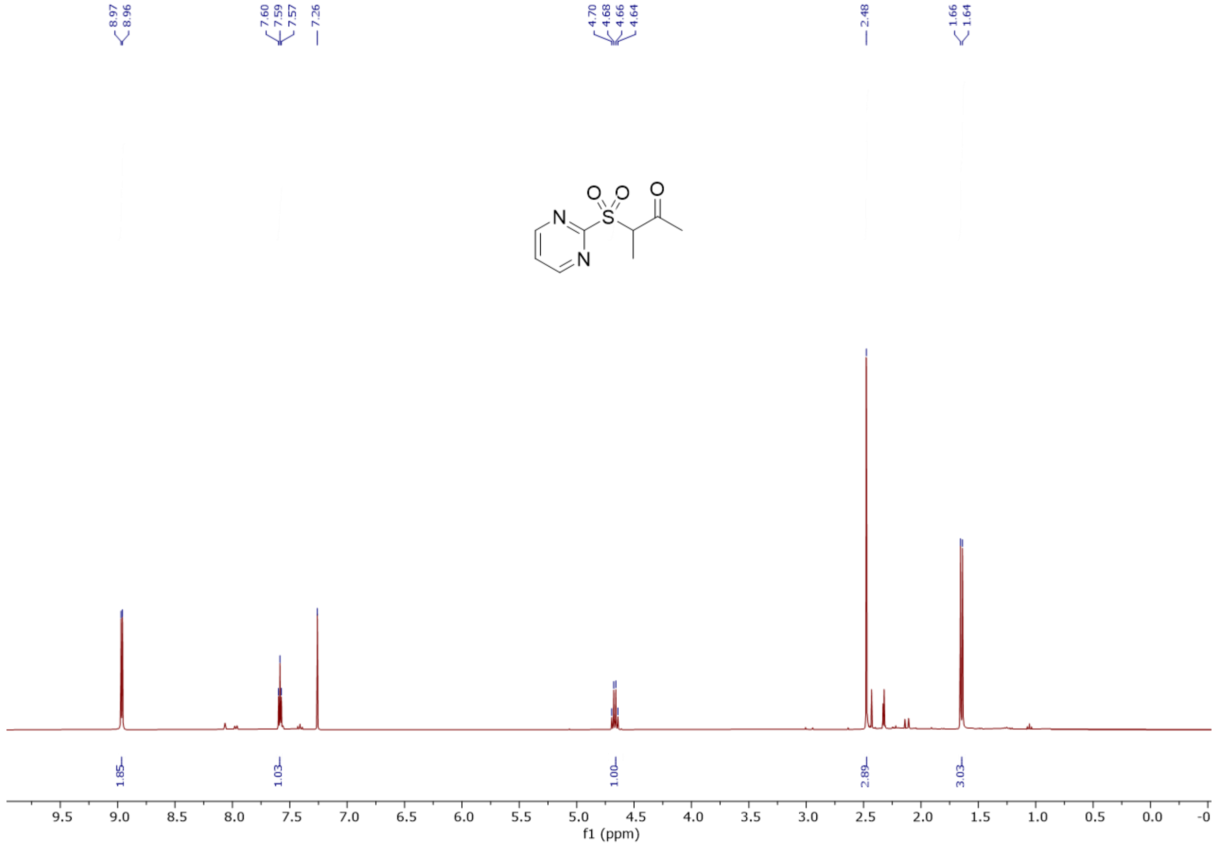
8.97
8.96
7.60
7.59
7.57
7.26
4.70
4.68
4.66
4.64
2.48
1.66
1.64
1.85
1.03
1.00
2.89
3.03
f1 (ppm)

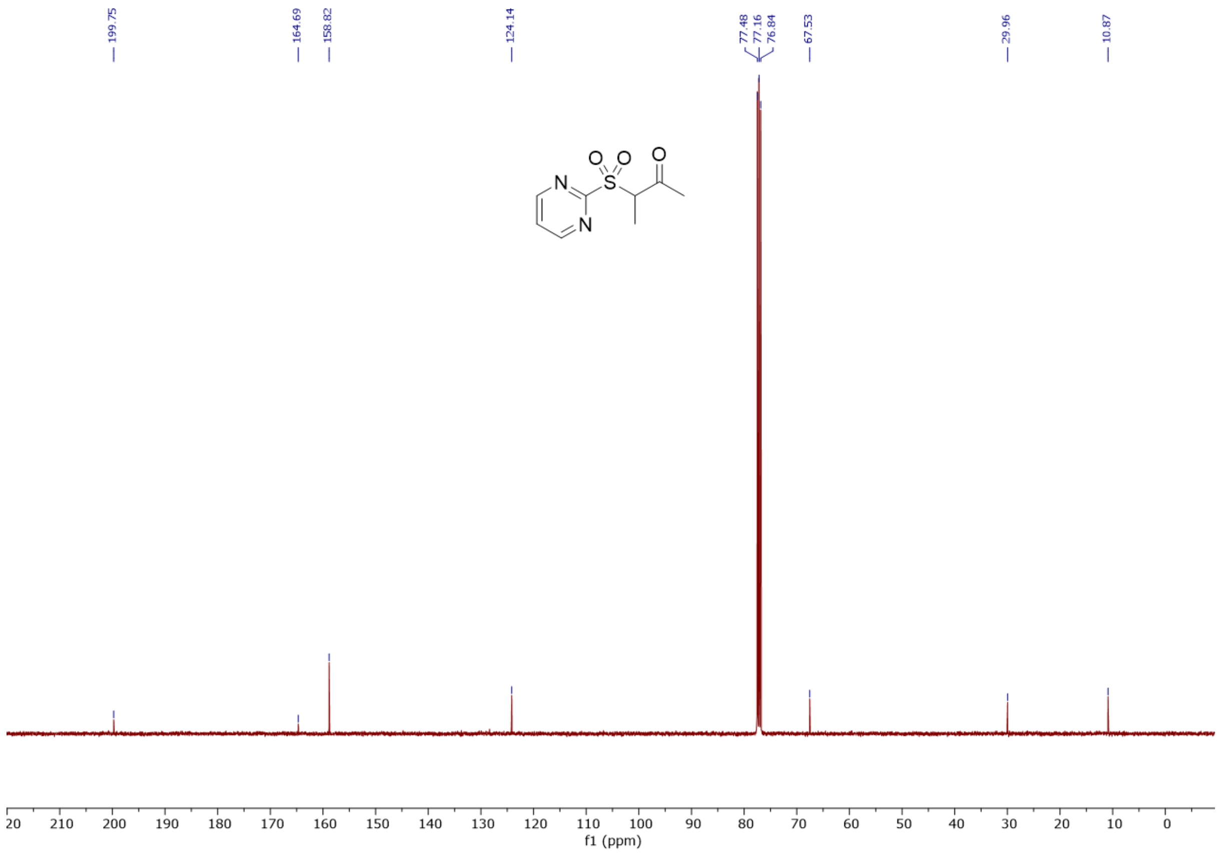

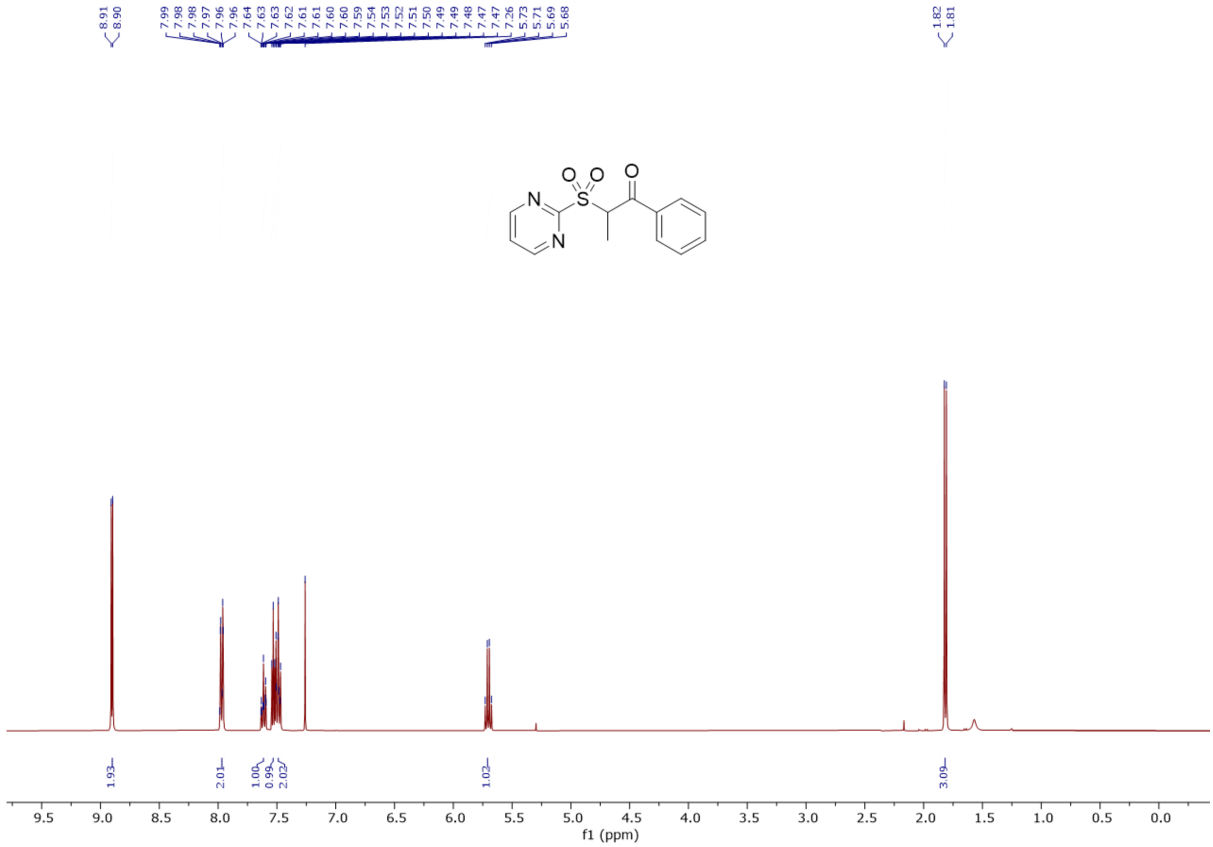

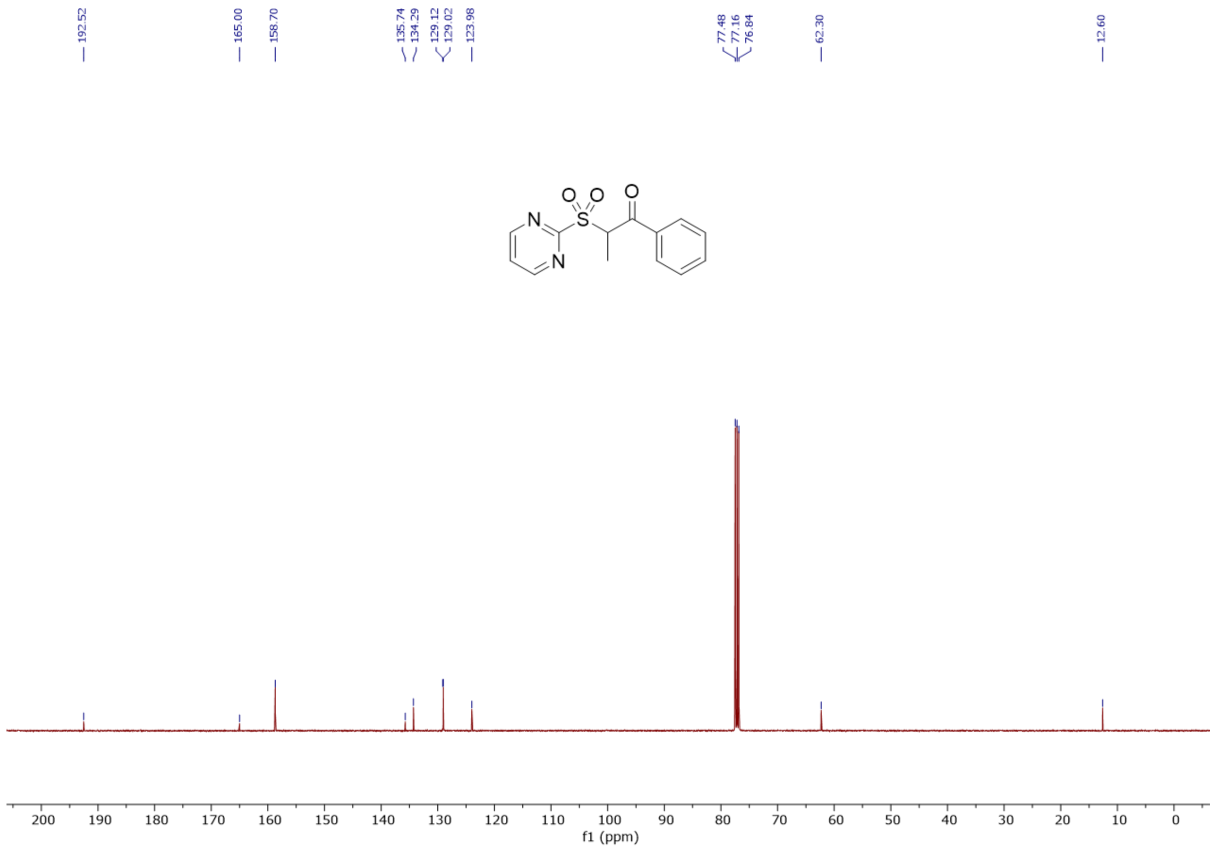

192.52
165.00
158.70
135.74
134.29
129.12
129.02
123.98
77.48
77.16
76.84
62.30
12.60
f1 (ppm)

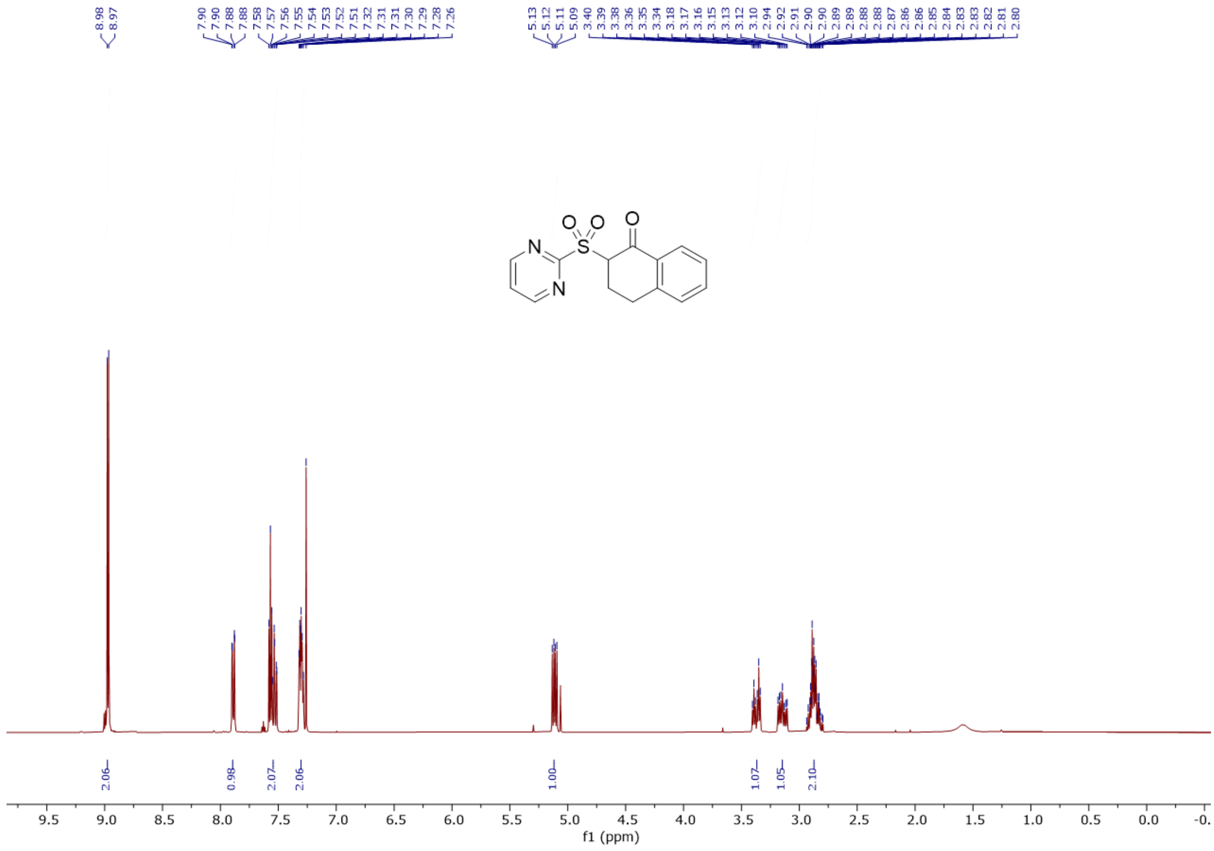

8.98
8.97
7.90
7.90
7.88
7.88
7.58
7.57
7.56
7.55
7.54
7.53
7.52
7.51
7.32
7.31
7.31
7.30
7.29
7.28
7.26
5.13
5.12
5.11
5.09
3.40
3.39
3.38
3.36
3.35
3.34
3.18
3.17
3.16
3.15
3.13
3.12
3.10
2.94
2.92
2.91
2.90
2.90
2.89
2.89
2.88
2.88
2.87
2.86
2.86
2.85
2.84
2.83
2.83
2.82
2.81
2.80
2.06
0.98
2.07
2.06
1.00
1.07
1.05
2.10
f1 (ppm)

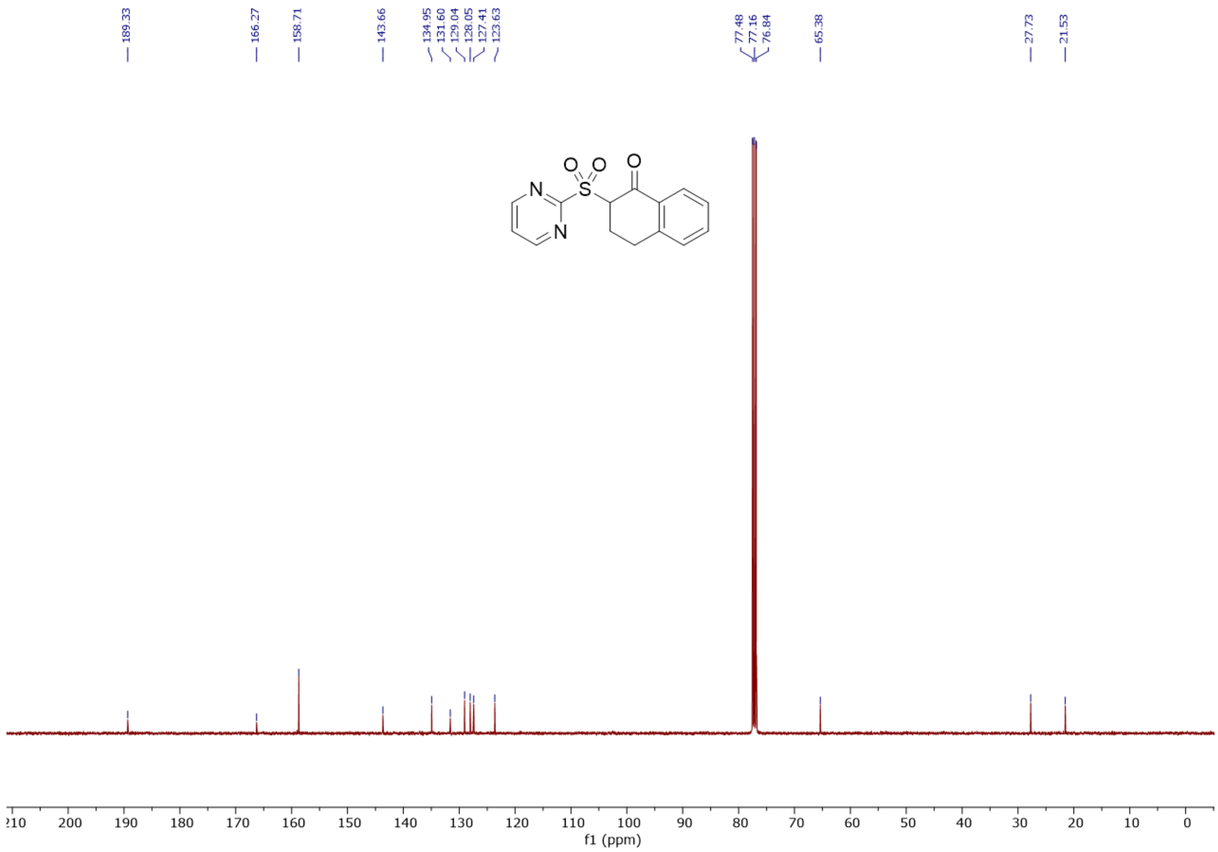
189.33
166.27
158.71
143.66
134.95
131.60
129.04
128.05
127.41
123.63
77.48
77.16
76.84
65.38
27.73
21.53
f1 (ppm)

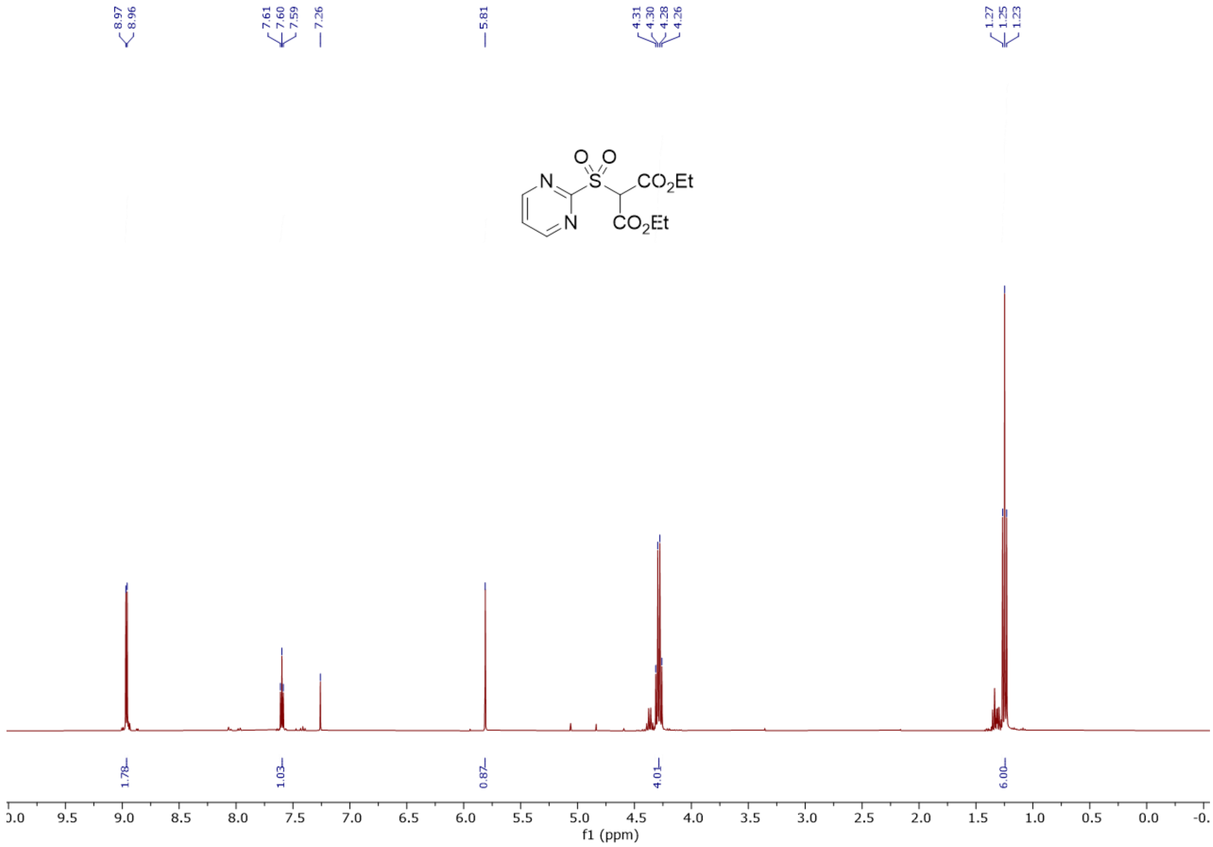
8.97
8.96
7.61
7.60
7.59
7.26
5.81
4.31
4.30
4.28
4.26
1.27
1.25
1.23
1.78
1.03
0.87
4.01
6.00
f1 (ppm)

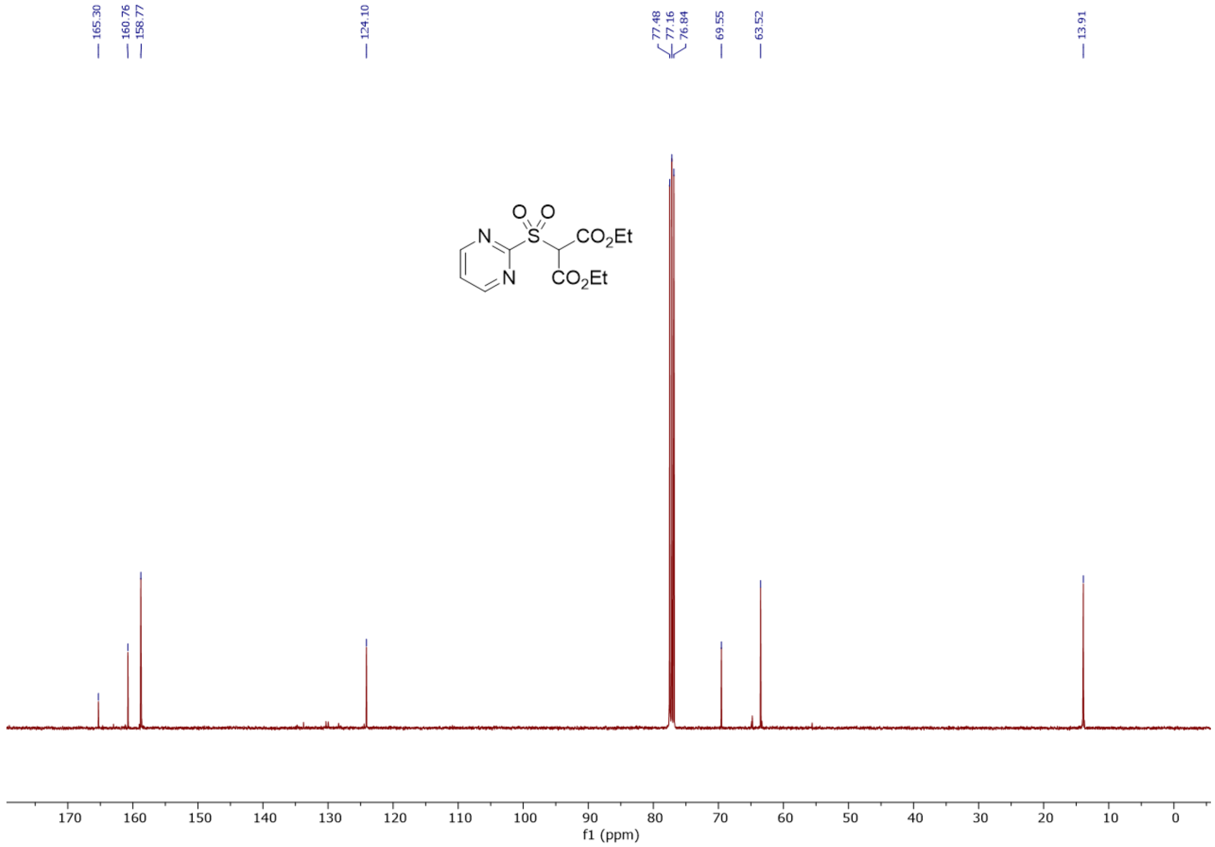
165.30
160.76
158.77
124.10
77.48
77.16
76.84
69.55
63.52
13.91
O O
N
S
CO2Et
N
CO2Et
170 160 150 140 130 120 110 100 90 80 70 60 50 40 30 20 10 0
f1 (ppm)

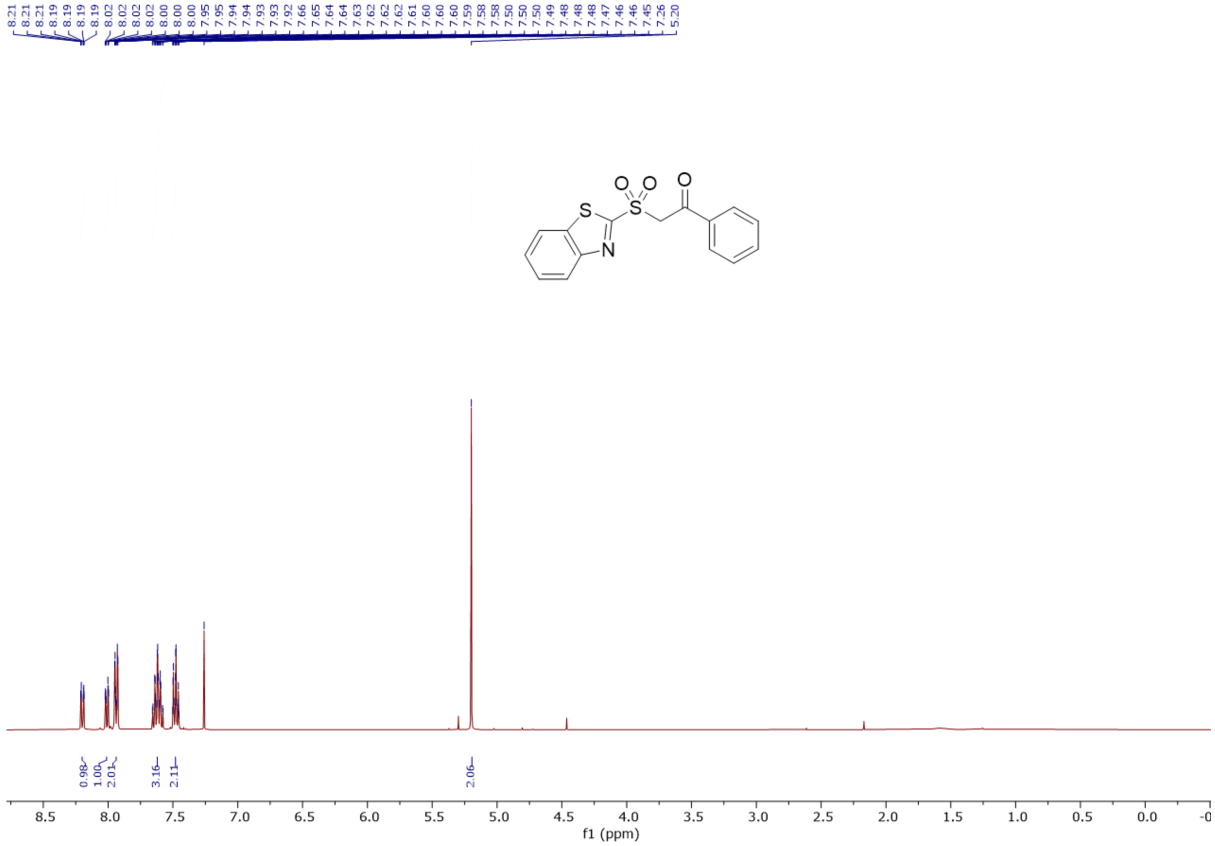
8.21 8.21 8.21 8.19 8.19 8.19 8.19 8.02 8.02 8.02 8.02 8.00 8.00 8.00 7.95 7.95 7.94 7.93 7.93 7.92 7.66 7.65 7.64 7.63 7.62 7.62 7.61 7.60 7.60 7.59 7.58 7.58 7.50 7.50 7.50 7.49 7.48 7.48 7.47 7.46 7.45 7.26 5.20
O O O
S
S
N
0.98
1.00
2.01
3.16
2.11
2.06
8.5 8.0 7.5 7.0 6.5 6.0 5.5 5.0 4.5 4.0 3.5 3.0 2.5 2.0 1.5 1.0 0.5 0.0
f1 (ppm)

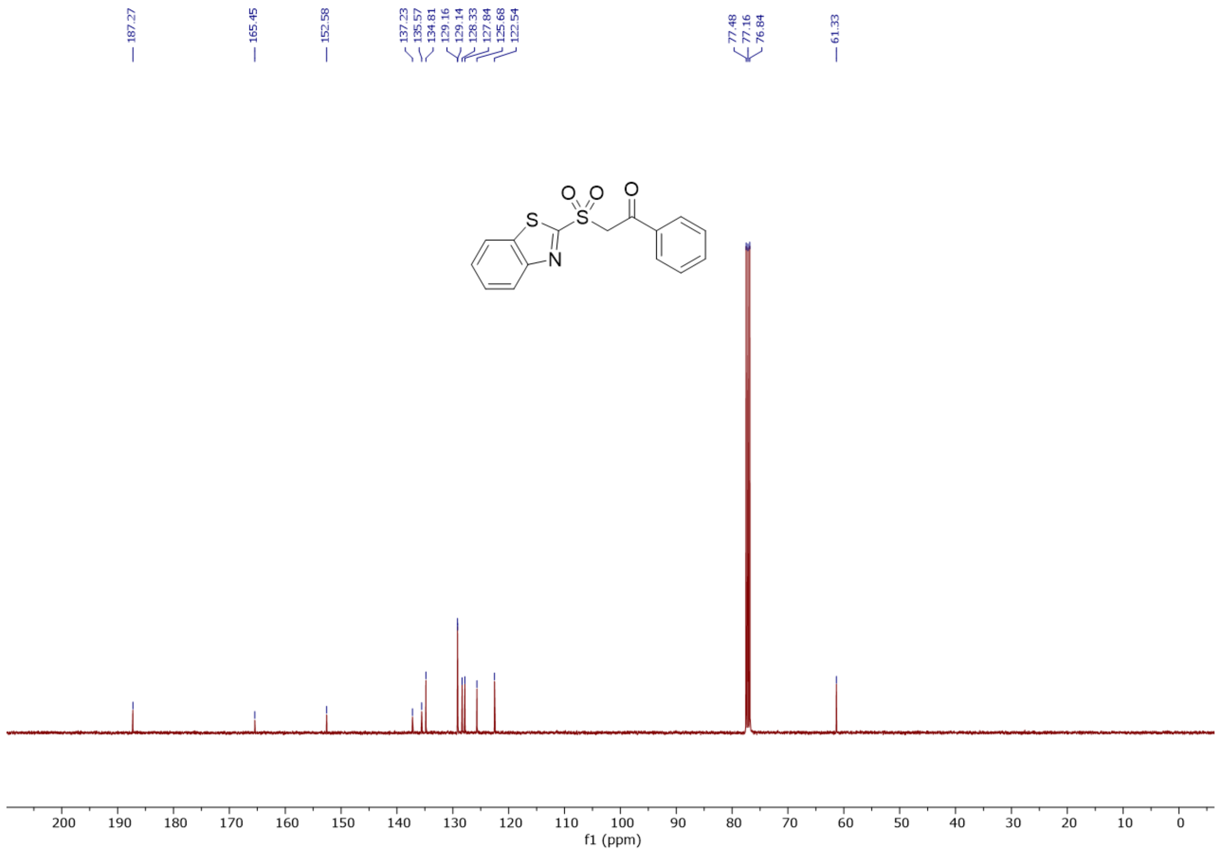
187.27
165.45
152.58
137.23
135.57
134.81
129.16
129.14
128.33
127.84
125.68
122.54
77.48
77.16
76.84
61.33
f1 (ppm)

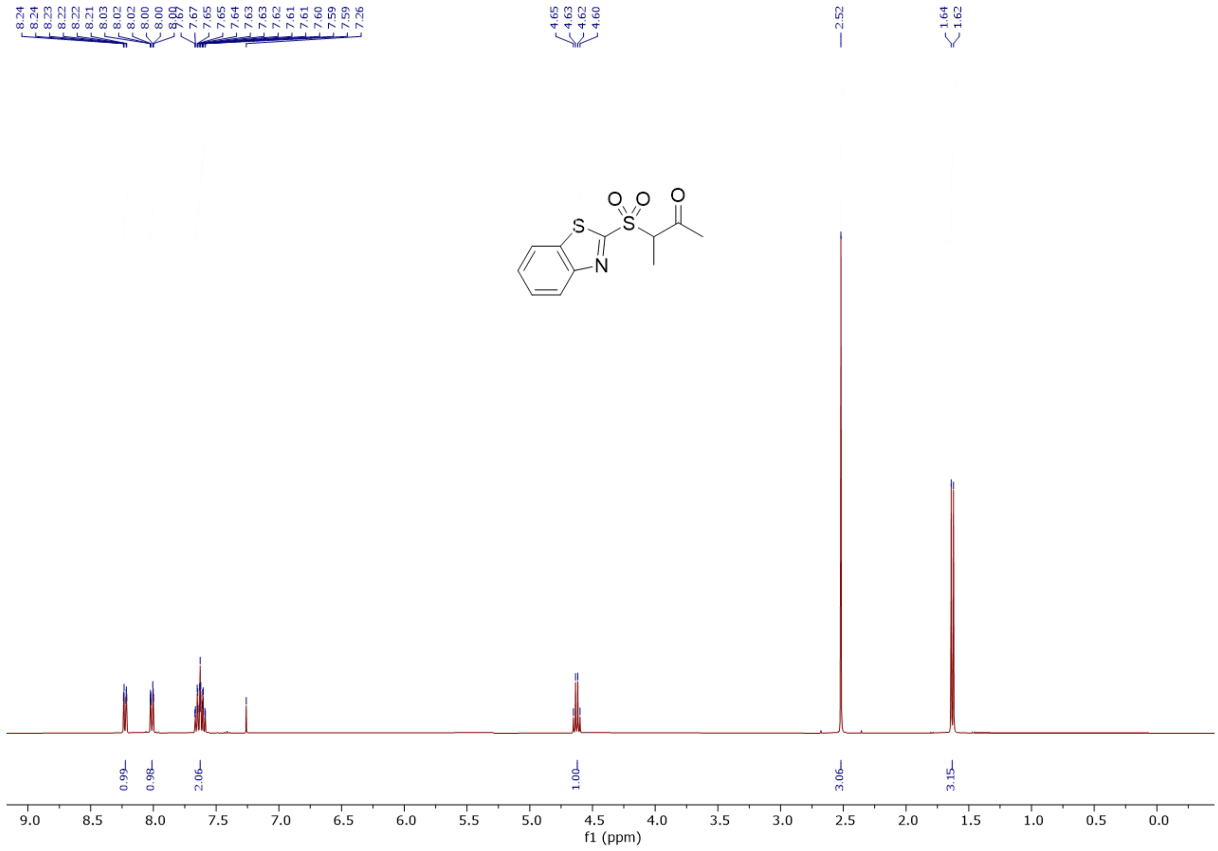
8.24
8.24
8.23
8.22
8.22
8.21
8.03
8.02
8.02
8.00
8.00
7.67
7.65
7.65
7.64
7.63
7.63
7.62
7.61
7.61
7.60
7.59
7.59
7.26
4.65
4.63
4.62
4.60
2.52
1.64
1.62
0.99
0.98
2.06
1.00
3.06
3.15
f1 (ppm)

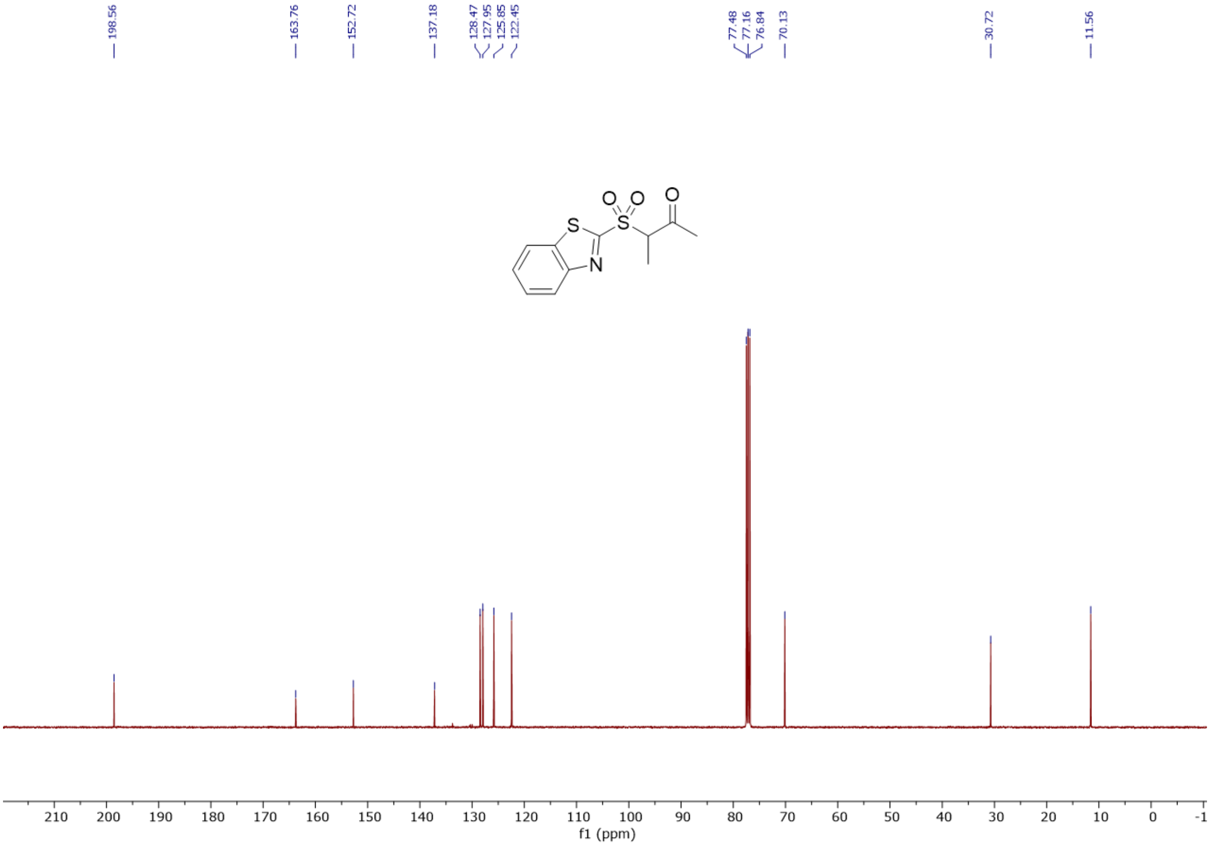
198.56
163.76
152.72
137.18
128.47
127.95
125.85
122.45
77.48
77.16
76.84
70.13
30.72
11.56
210 200 190 180 170 160 150 140 130 120 110 100 90 80 70 60 50 40 30 20 10 0 -1
f1 (ppm)

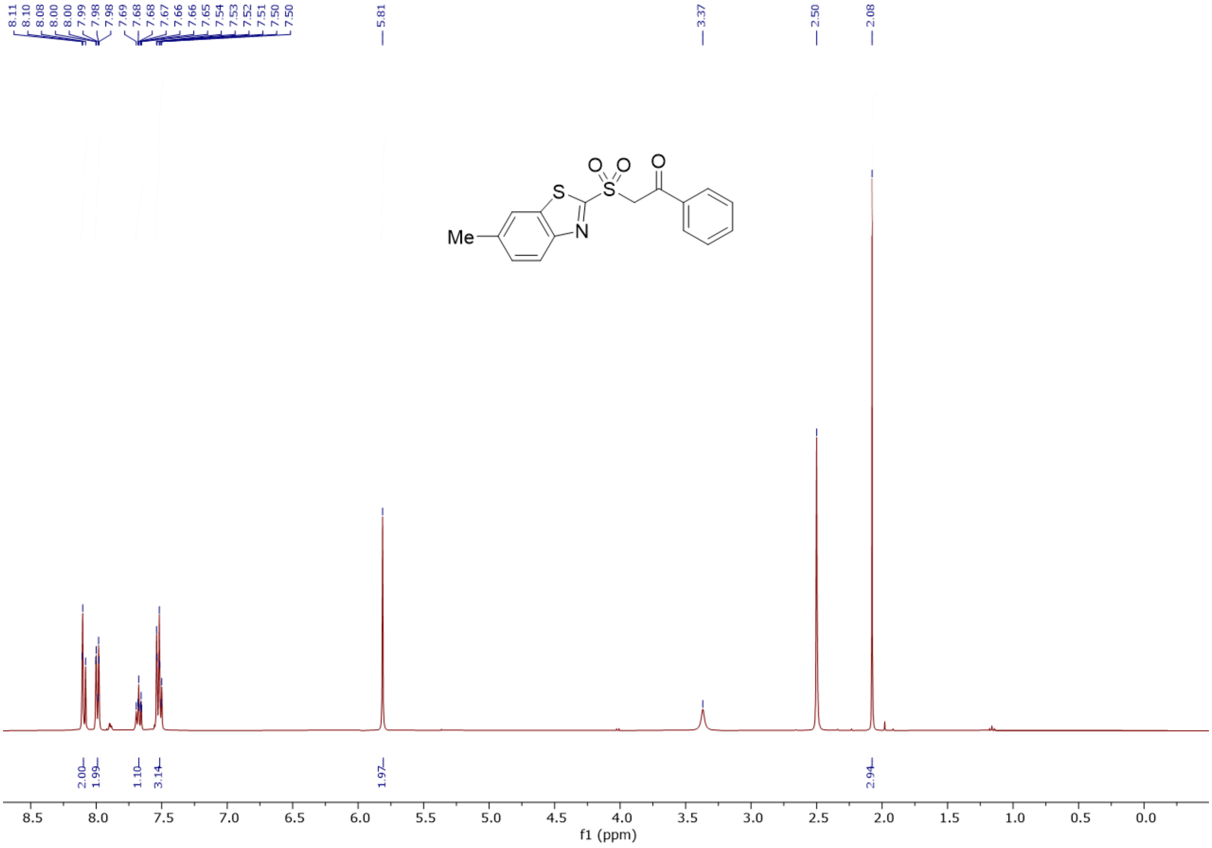
8.11 8.10 8.08 8.00 8.00 7.99 7.98 7.98 7.69 7.68 7.68 7.67 7.66 7.66 7.65 7.54 7.53 7.52 7.51 7.50 7.50
5.81
3.37
2.50
2.08
Me
2.00
1.99
1.10
3.14
1.97
2.94
8.5 8.0 7.5 7.0 6.5 6.0 5.5 5.0 4.5 4.0 3.5 3.0 2.5 2.0 1.5 1.0 0.5 0.0
f1 (ppm)

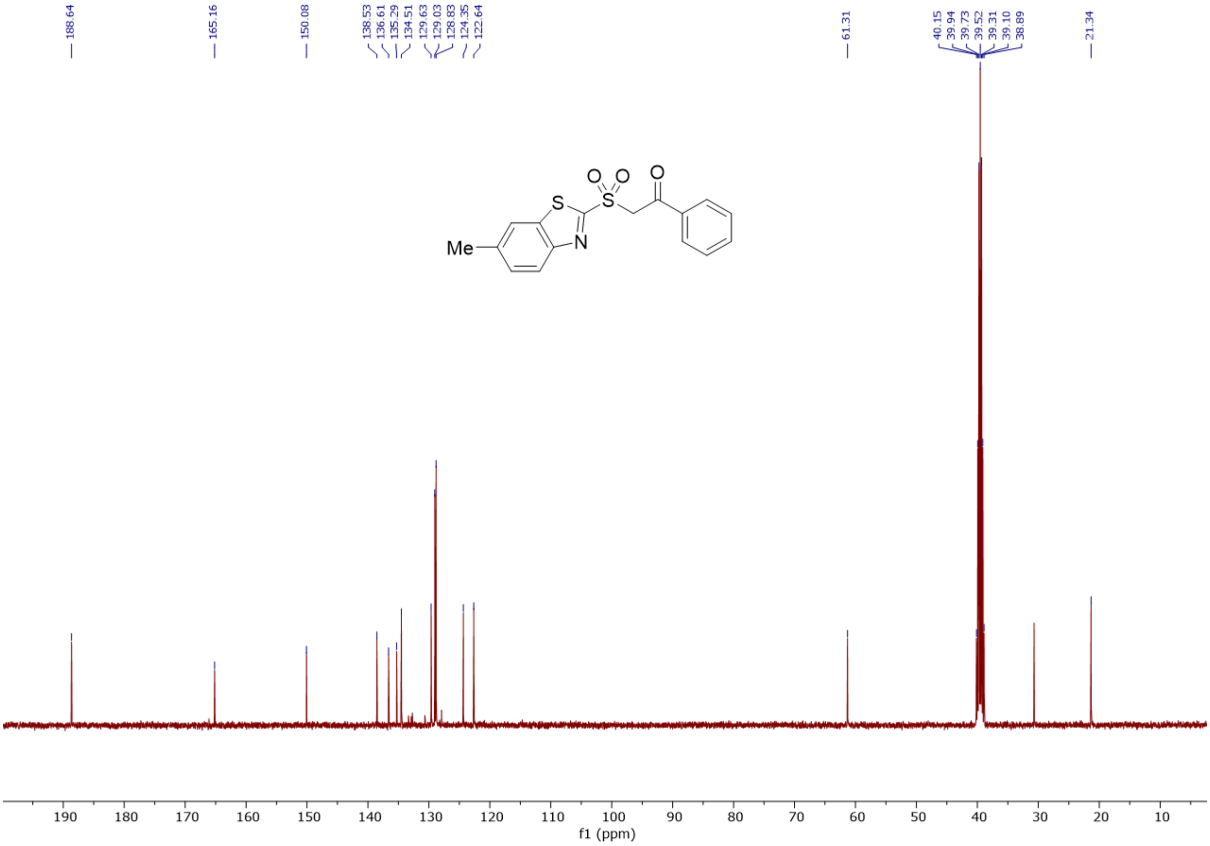
188.64
165.16
150.08
138.53
136.61
135.29
134.51
129.63
129.03
128.83
124.35
122.64
61.31
40.15
39.94
39.73
39.52
39.31
39.10
38.89
21.34
Me
S
N
O
190 180 170 160 150 140 130 120 110 100 90 80 70 60 50 40 30 20 10
f1 (ppm)

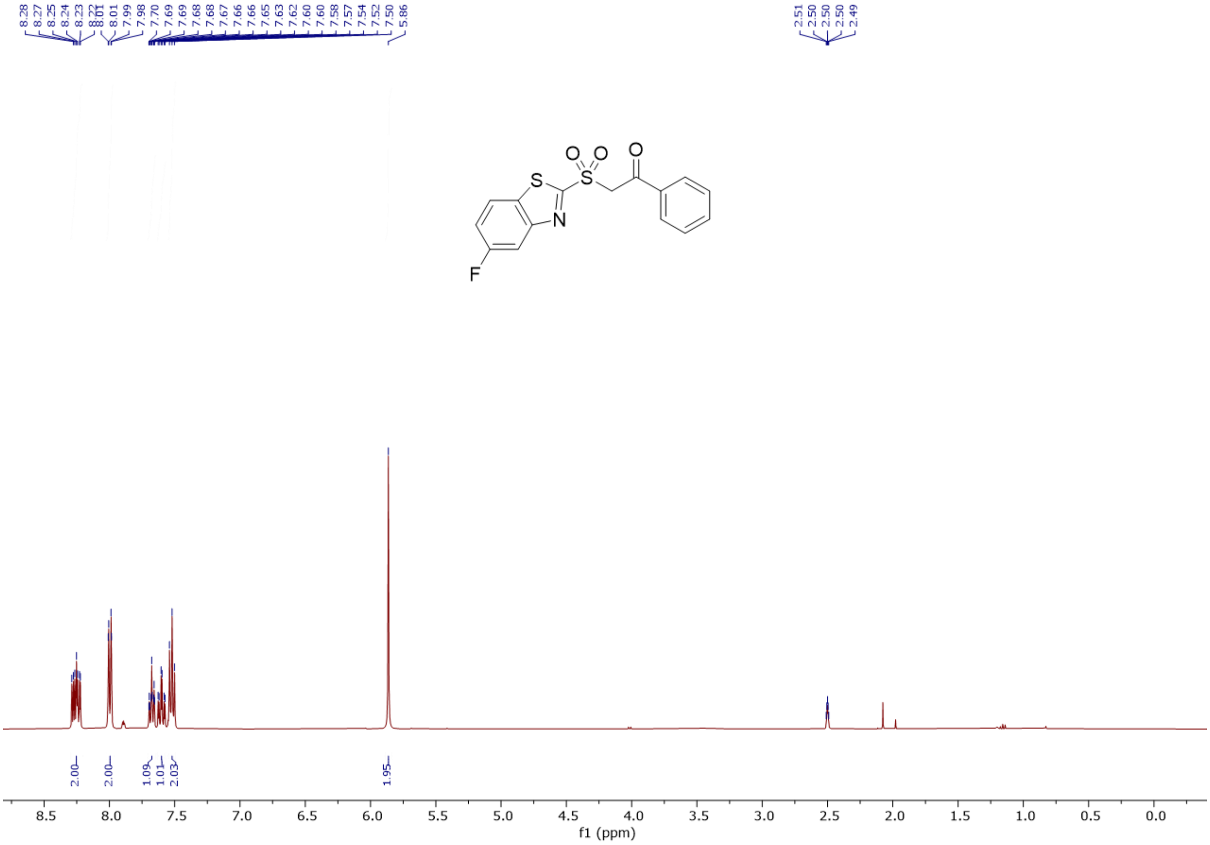
8.28
8.27
8.25
8.24
8.23
8.22
8.01
8.01
7.99
7.98
7.70
7.69
7.69
7.68
7.68
7.67
7.66
7.66
7.65
7.63
7.62
7.60
7.60
7.58
7.57
7.54
7.52
7.50
5.86
2.51
2.50
2.50
2.50
2.49
S
N
O
F
2.00
2.00
1.09
1.01
2.03
1.95
8.5 8.0 7.5 7.0 6.5 6.0 5.5 5.0 4.5 4.0 3.5 3.0 2.5 2.0 1.5 1.0 0.5 0.0
f1 (ppm)

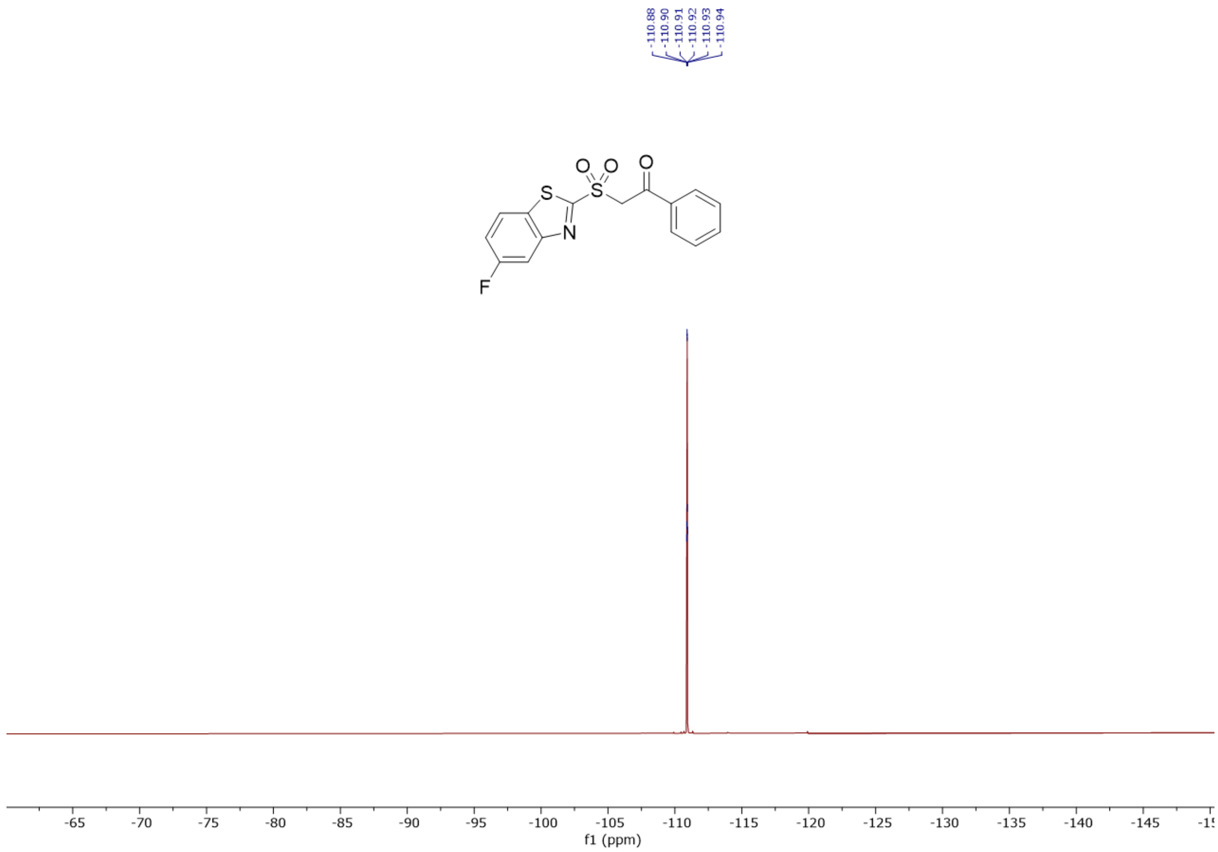
-110.88
-110.90
-110.91
-110.92
-110.93
-110.94
S
N
O
F
-65 -70 -75 -80 -85 -90 -95 -100 -105 -110 -115 -120 -125 -130 -135 -140 -145
f1 (ppm)

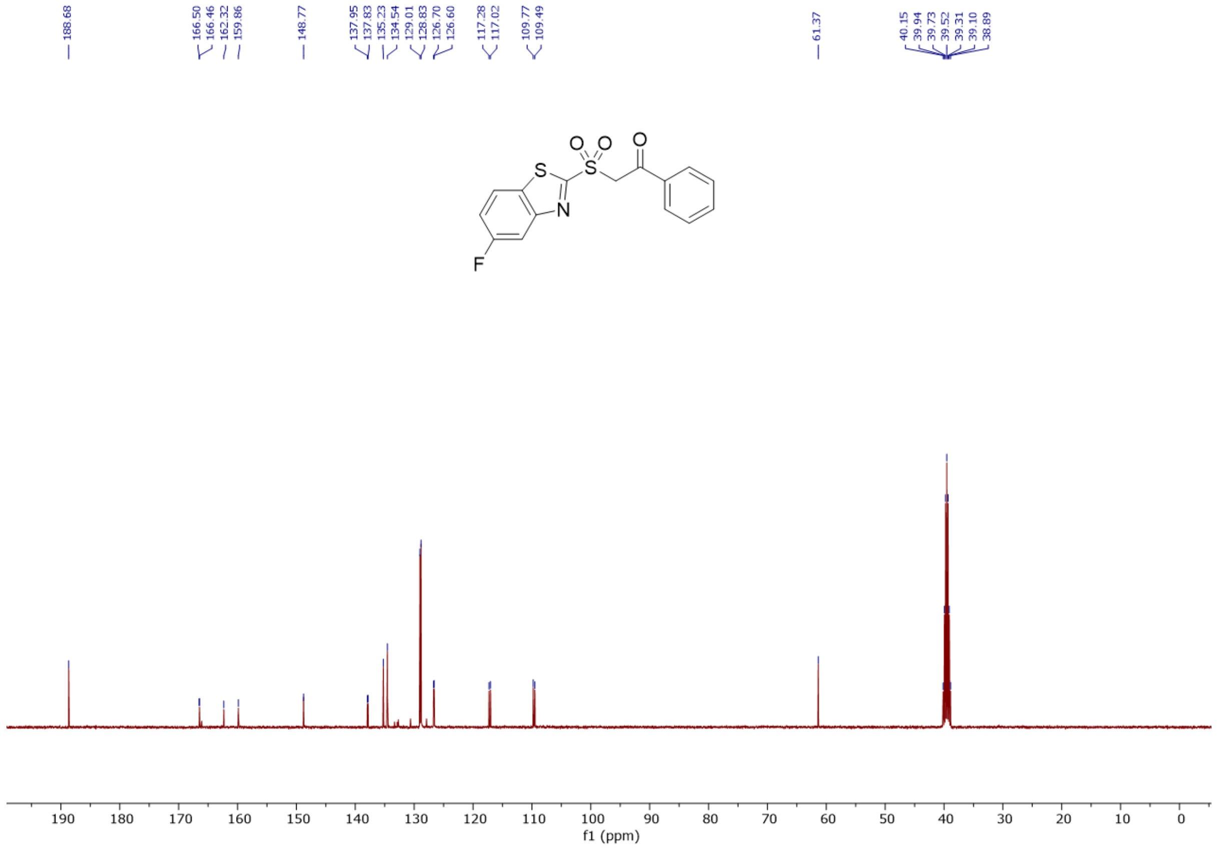
188.68
166.50
166.46
162.32
159.86
148.77
137.95
137.83
135.23
134.54
129.01
128.83
126.70
126.60
117.28
117.02
109.77
109.49
61.37
40.15
39.94
39.73
39.52
39.31
39.10
38.89
S
N
O
F
190 180 170 160 150 140 130 120 110 100 90 80 70 60 50 40 30 20 10 0
f1 (ppm)

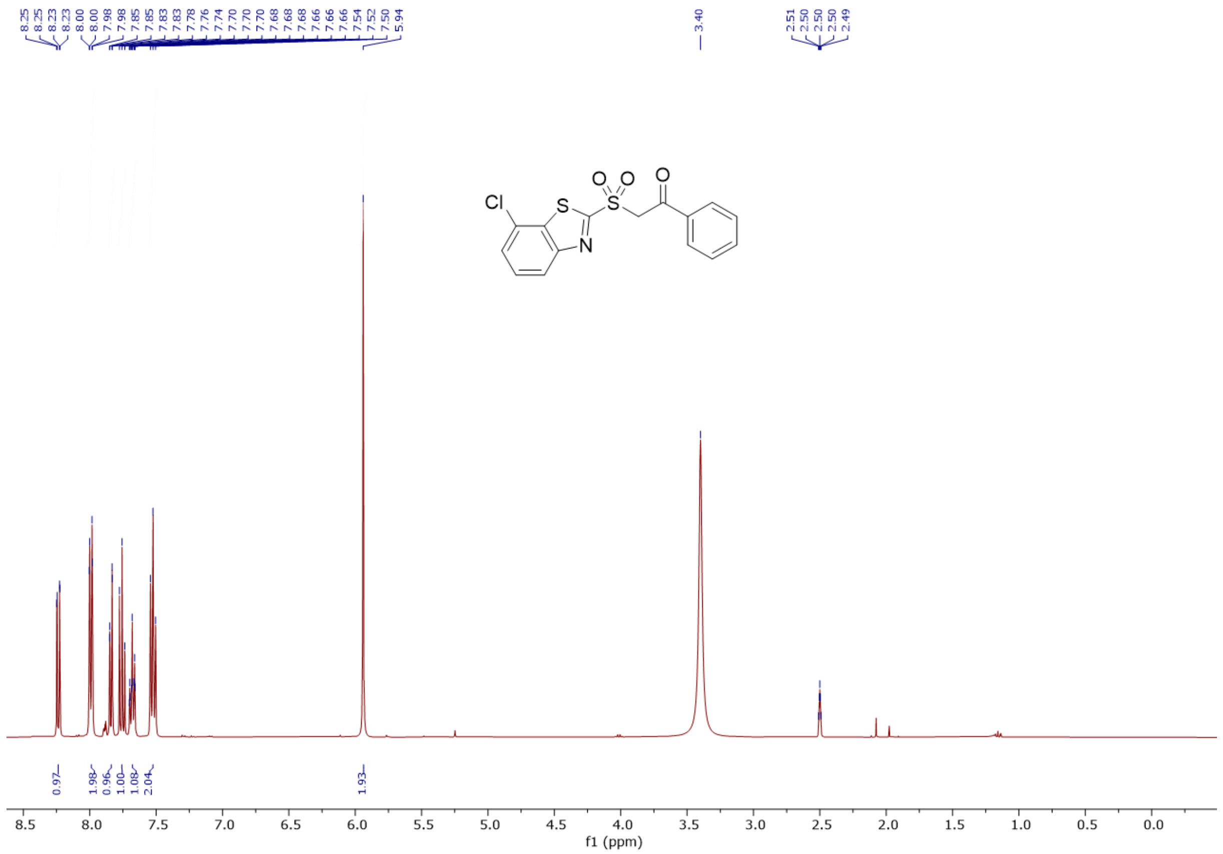

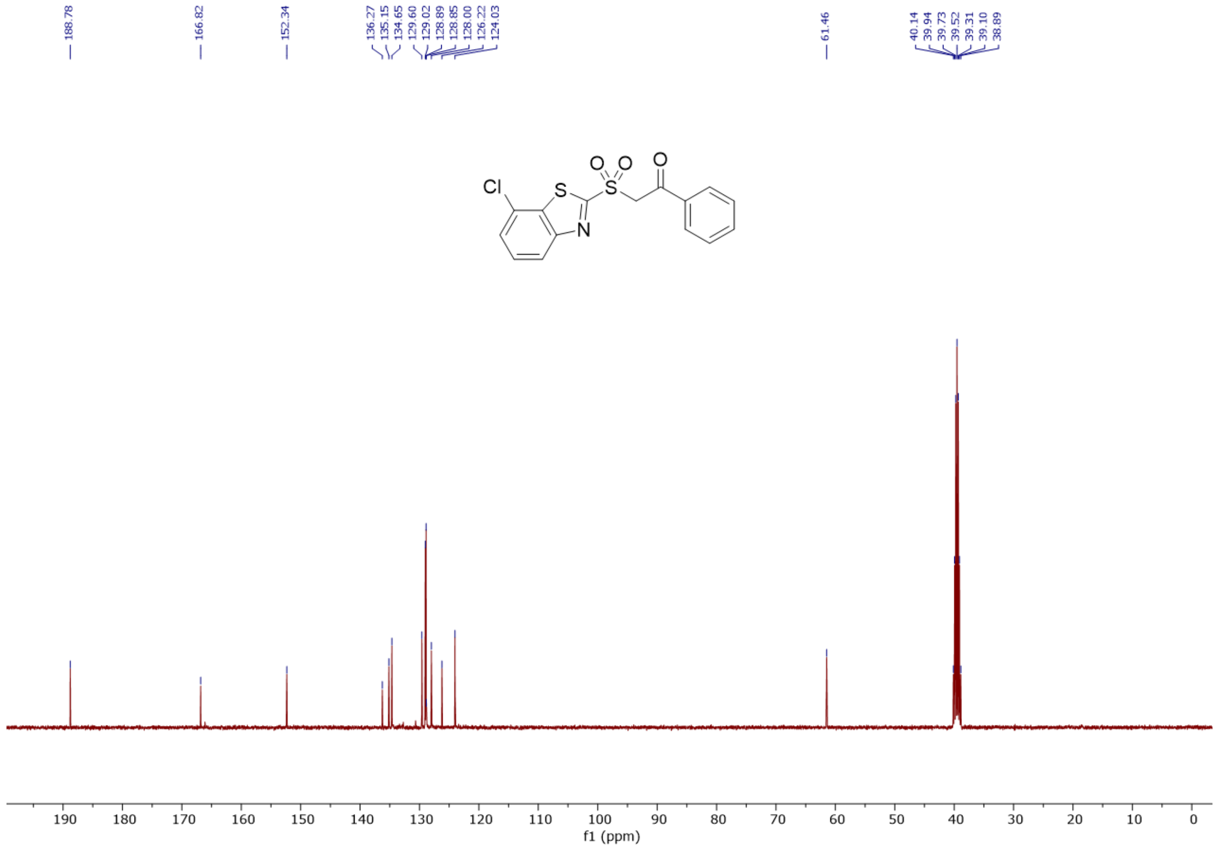

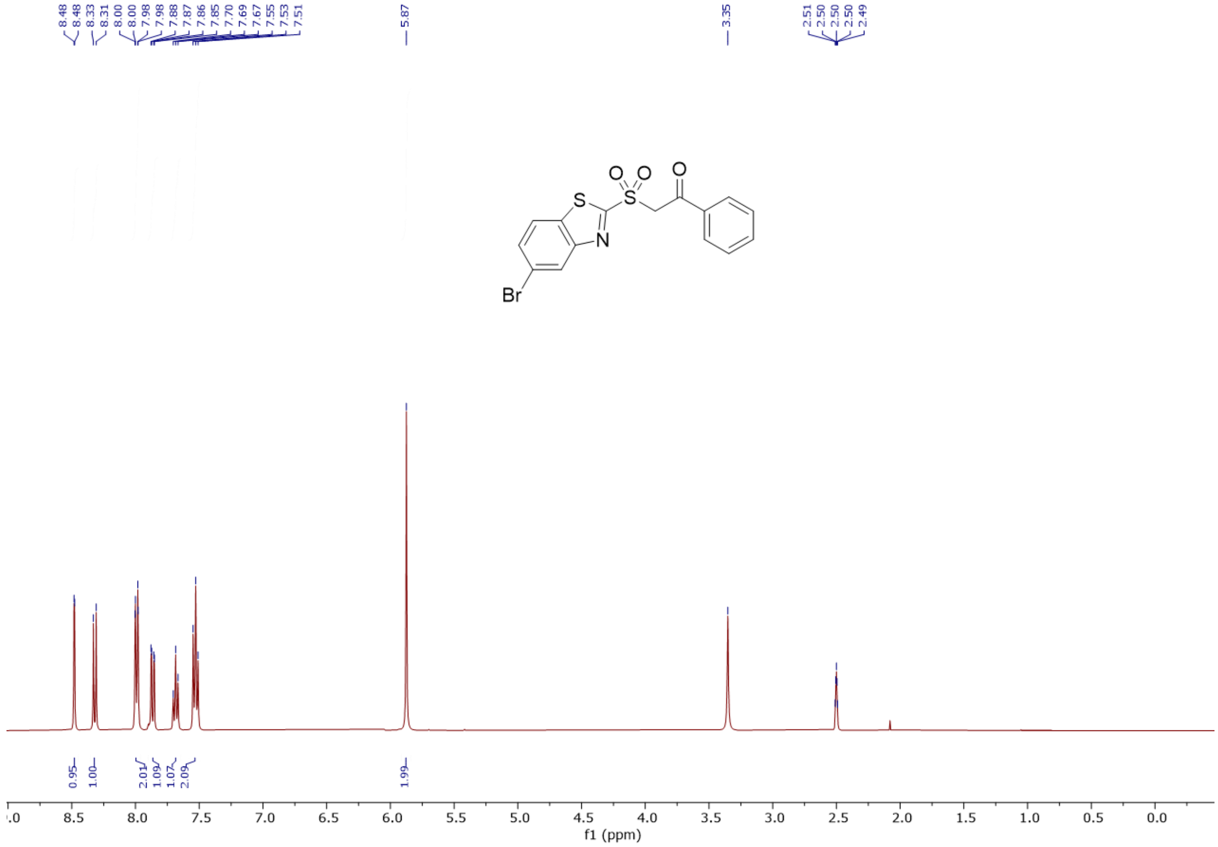
8.48
8.48
8.33
8.31
8.00
8.00
7.98
7.98
7.88
7.87
7.86
7.85
7.70
7.69
7.67
7.55
7.53
7.51
5.87
3.35
2.51
2.50
2.50
2.50
2.49
S
N
O
O
O
Br
0.95
1.00
2.01
1.09
1.07
2.09
1.99
f1 (ppm)

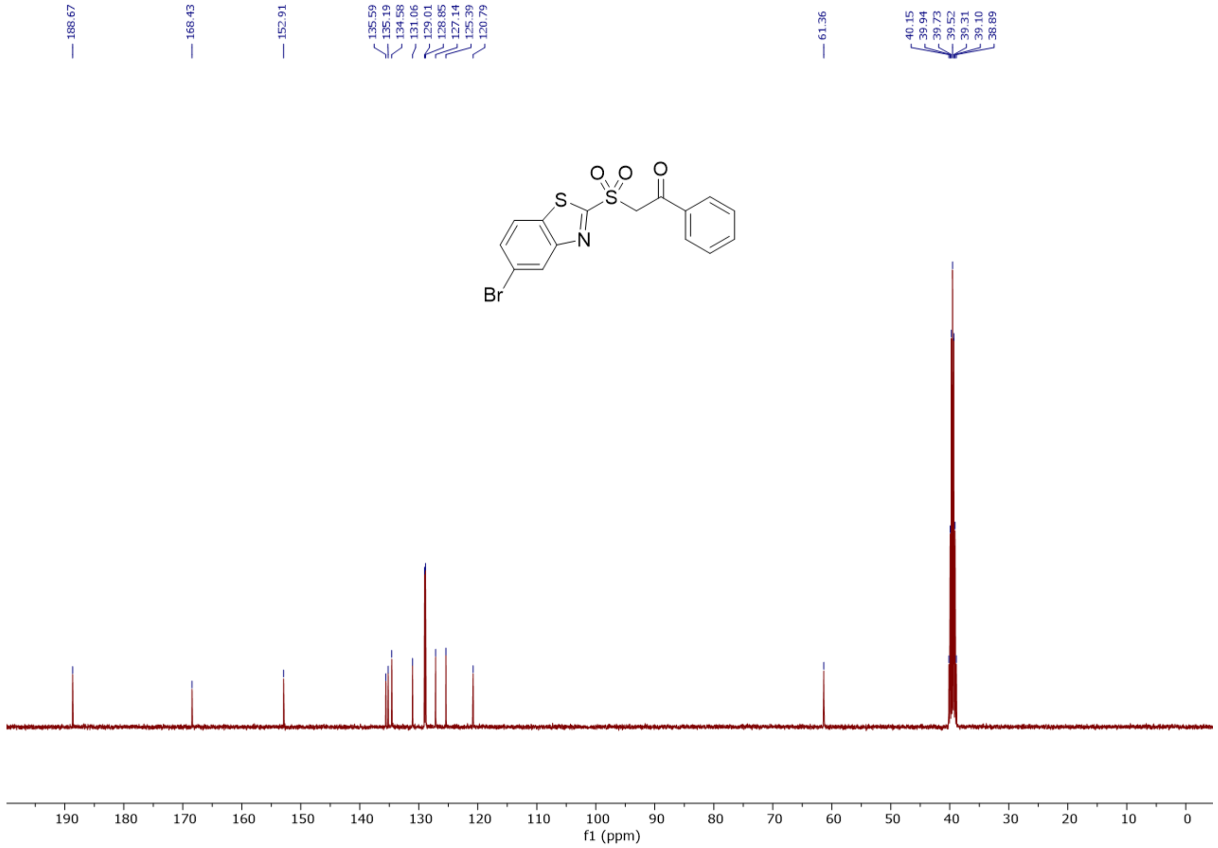
188.67
168.43
152.91
135.59
135.19
134.58
131.06
129.01
128.85
127.14
125.39
120.79
61.36
40.15
39.94
39.73
39.52
39.31
39.10
38.89
S
N
O
O
O
Br
f1 (ppm)

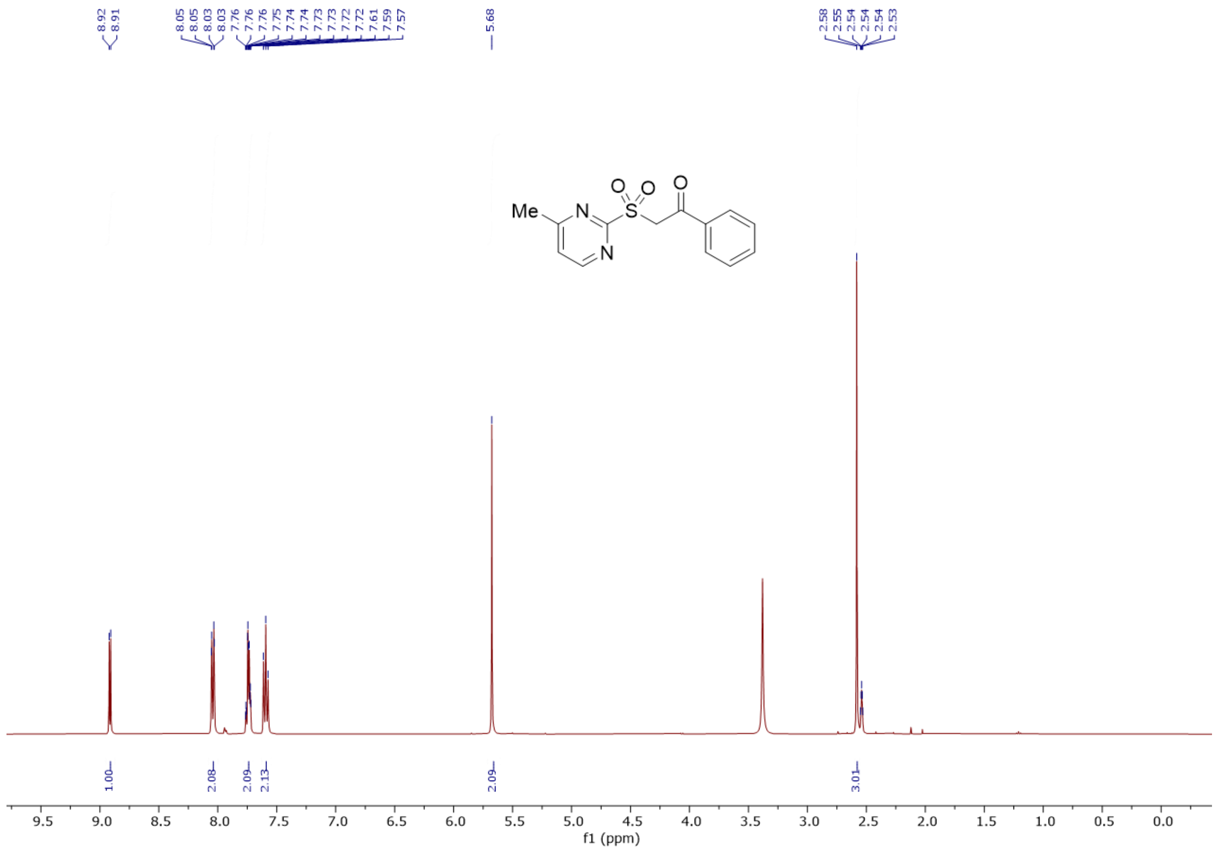
8.92
8.91
8.05
8.05
8.03
8.03
7.76
7.76
7.75
7.74
7.74
7.73
7.73
7.72
7.72
7.61
7.59
7.57
5.68
2.58
2.55
2.54
2.54
2.54
2.53
Me
N
N
S
O
O
O
1.00
2.08
2.09
2.13
2.09
3.01
9.5
9.0
8.5
8.0
7.5
7.0
6.5
6.0
5.5
5.0
4.5
4.0
3.5
3.0
2.5
2.0
1.5
1.0
0.5
0.0
f1 (ppm)

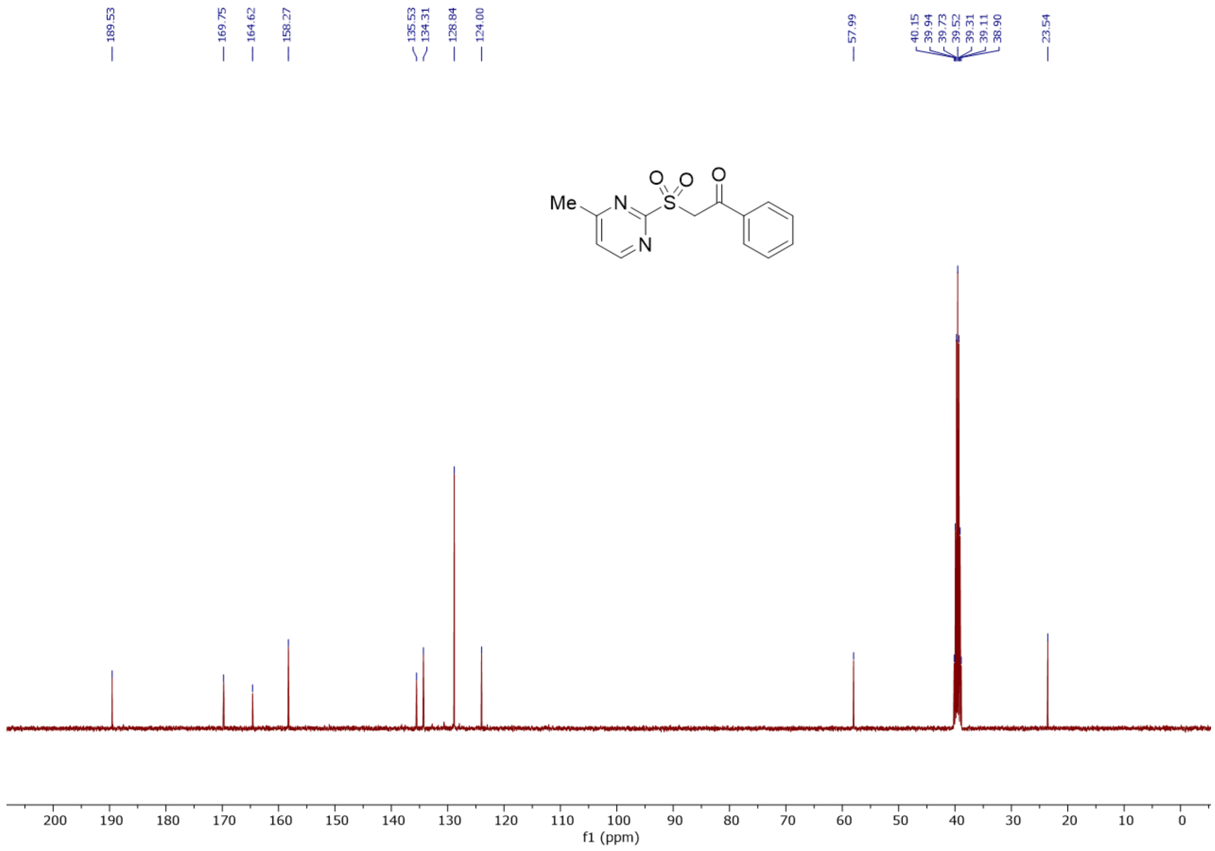
189.53
169.75
164.62
158.27
135.53
134.31
128.84
124.00
57.99
40.15
39.94
39.73
39.52
39.31
39.11
38.90
23.54
Me
N
N
S
O
O
O
200
190
180
170
160
150
140
130
120
110
100
90
80
70
60
50
40
30
20
10
0
f1 (ppm)

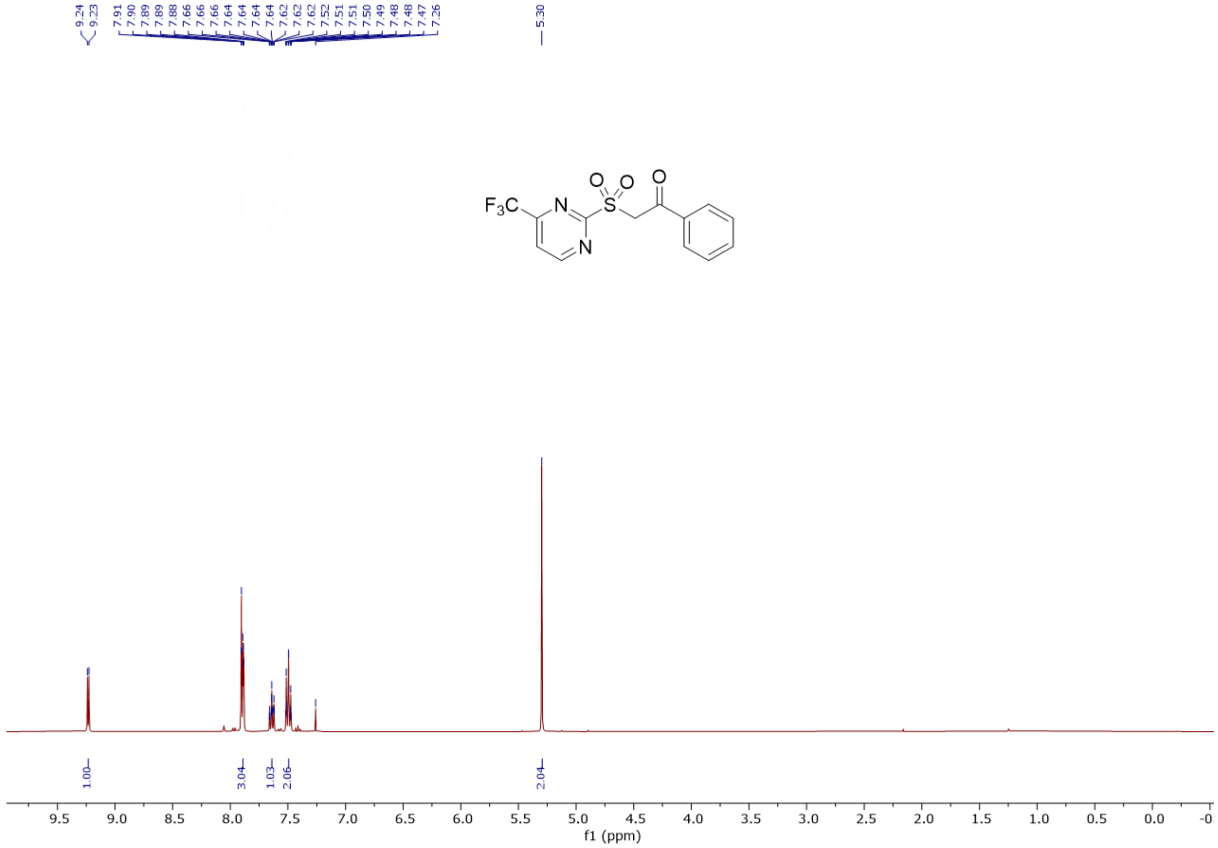
9.24
9.23
7.91
7.90
7.89
7.89
7.88
7.66
7.66
7.66
7.64
7.64
7.64
7.62
7.62
7.62
7.52
7.51
7.51
7.50
7.49
7.48
7.47
7.26
5.30
F3C
N
N
S
O
O
O
1.00
3.04
1.03
2.06
2.04
9.5 9.0 8.5 8.0 7.5 7.0 6.5 6.0 5.5 5.0 4.5 4.0 3.5 3.0 2.5 2.0 1.5 1.0 0.5 0.0 -0
f1 (ppm)

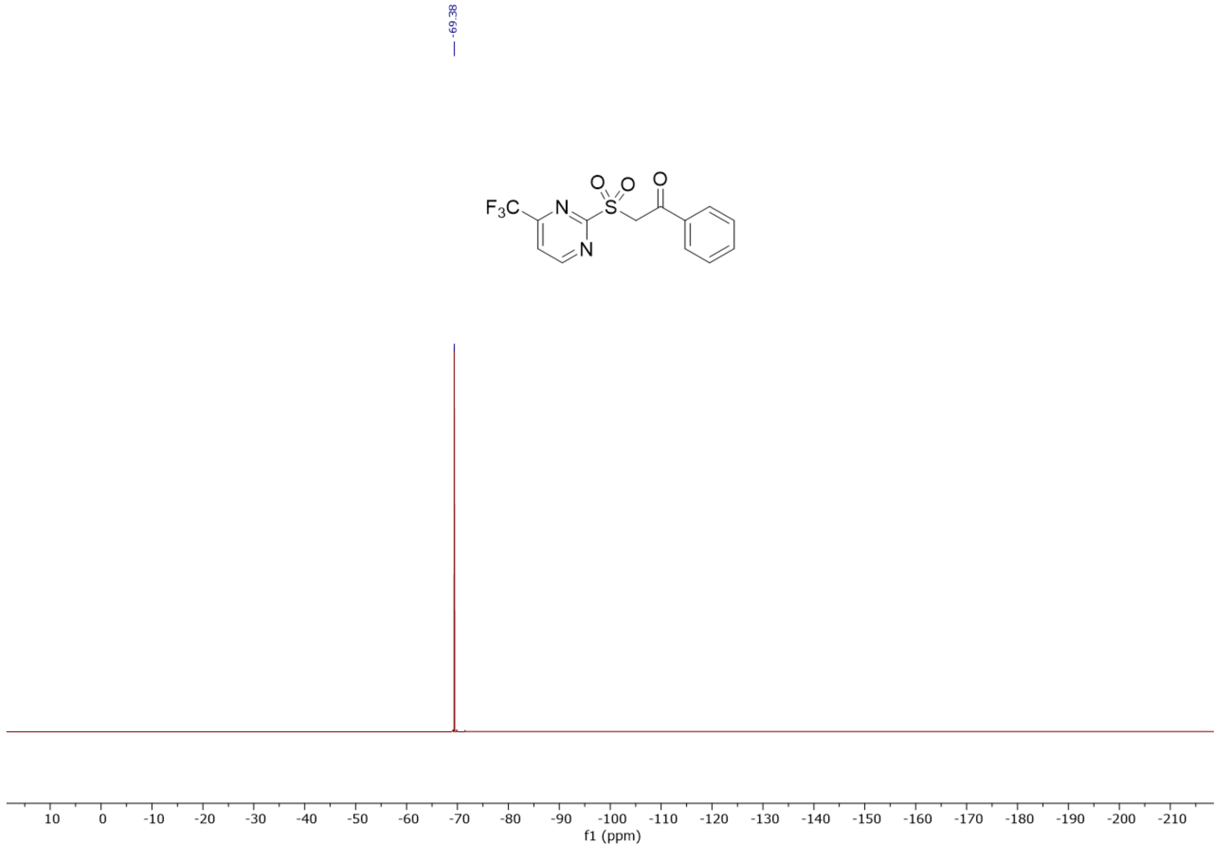
-69.38
F3C
N
N
S
O
O
O
10 0 -10 -20 -30 -40 -50 -60 -70 -80 -90 -100 -110 -120 -130 -140 -150 -160 -170 -180 -190 -200 -210
f1 (ppm)

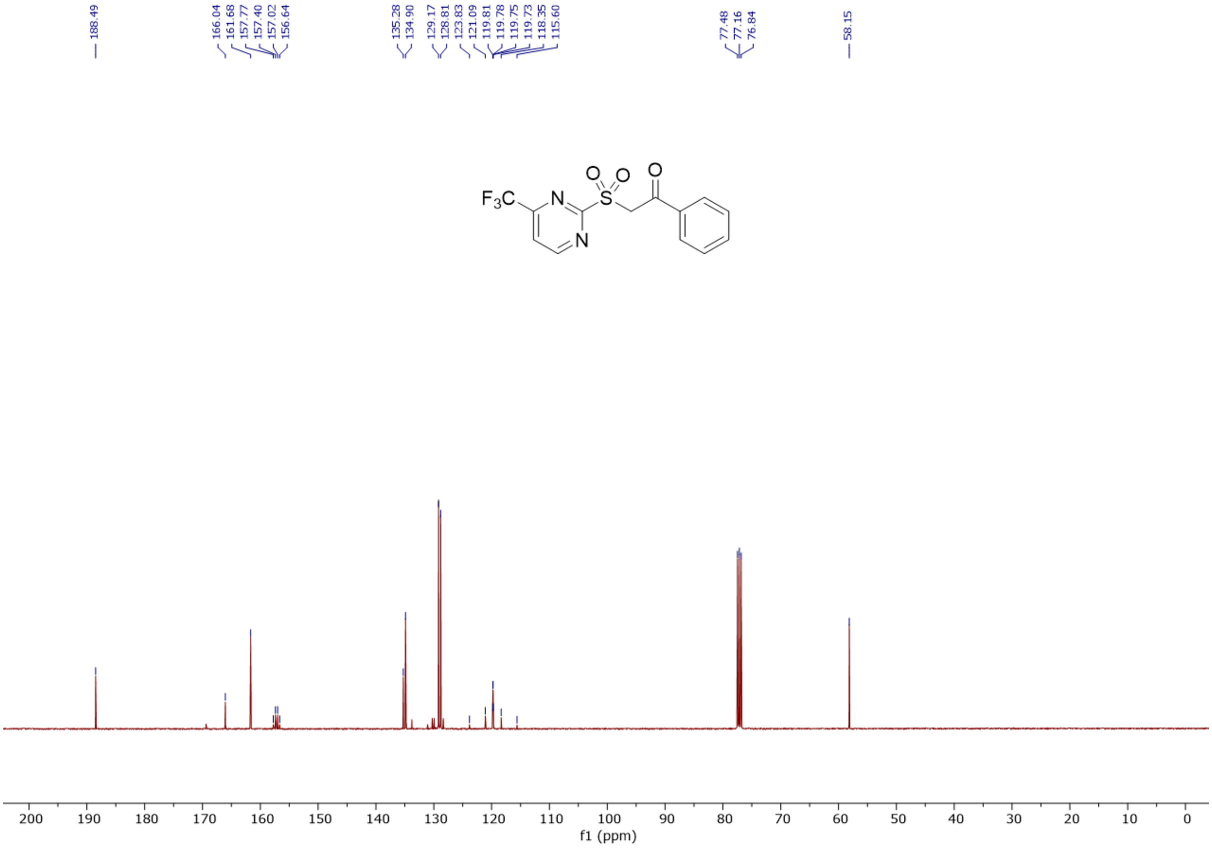
188.49
166.04
161.68
157.77
157.40
157.02
156.64
135.28
134.90
129.17
128.81
123.83
121.09
119.81
119.78
119.75
119.73
118.35
115.60
77.48
77.16
76.84
58.15
F3C
N
N
O
O
S
O
f1 (ppm)

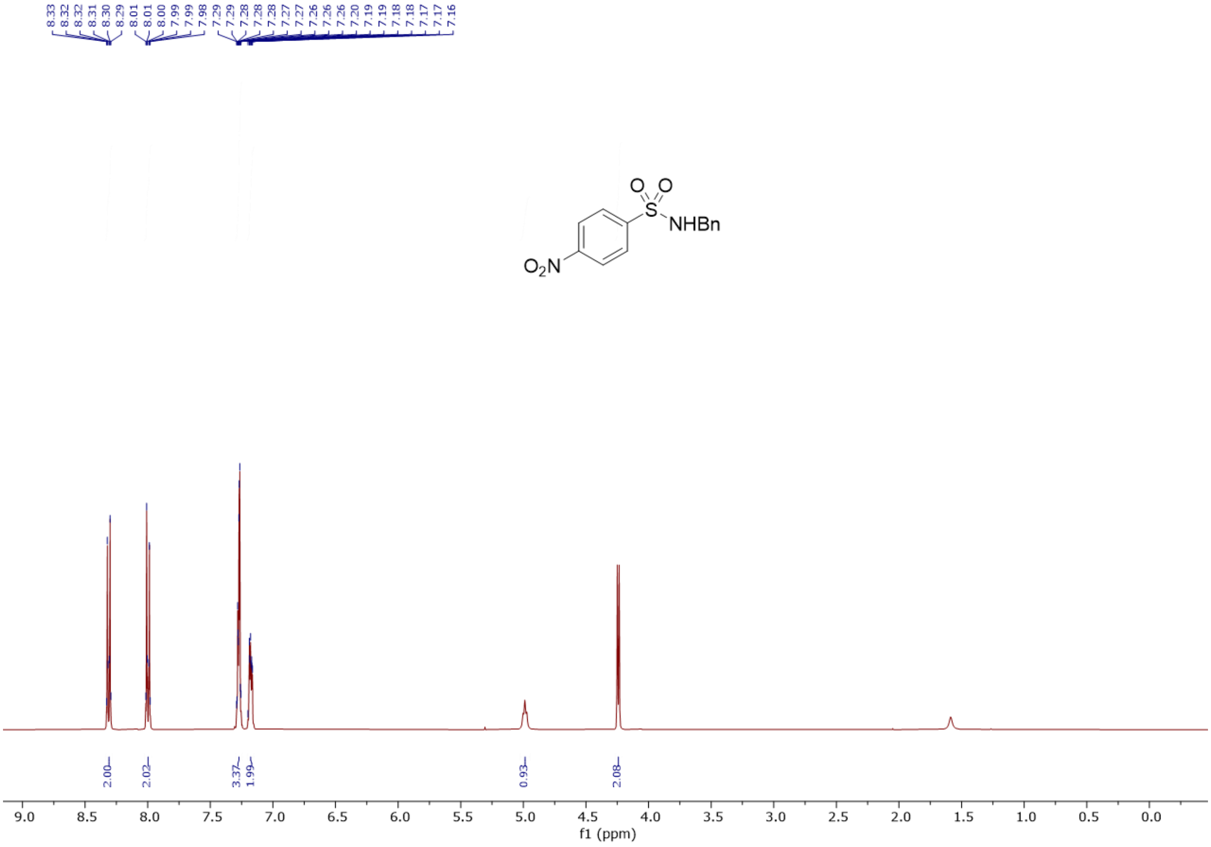
8.33
8.32
8.32
8.31
8.30
8.29
8.01
8.01
8.00
7.99
7.99
7.98
7.29
7.29
7.28
7.28
7.28
7.27
7.27
7.26
7.26
7.20
7.19
7.19
7.18
7.18
7.17
7.17
7.16
O
O
S
NHBn
O2N
2.00
2.02
3.37
1.99
0.93
2.08
f1 (ppm)

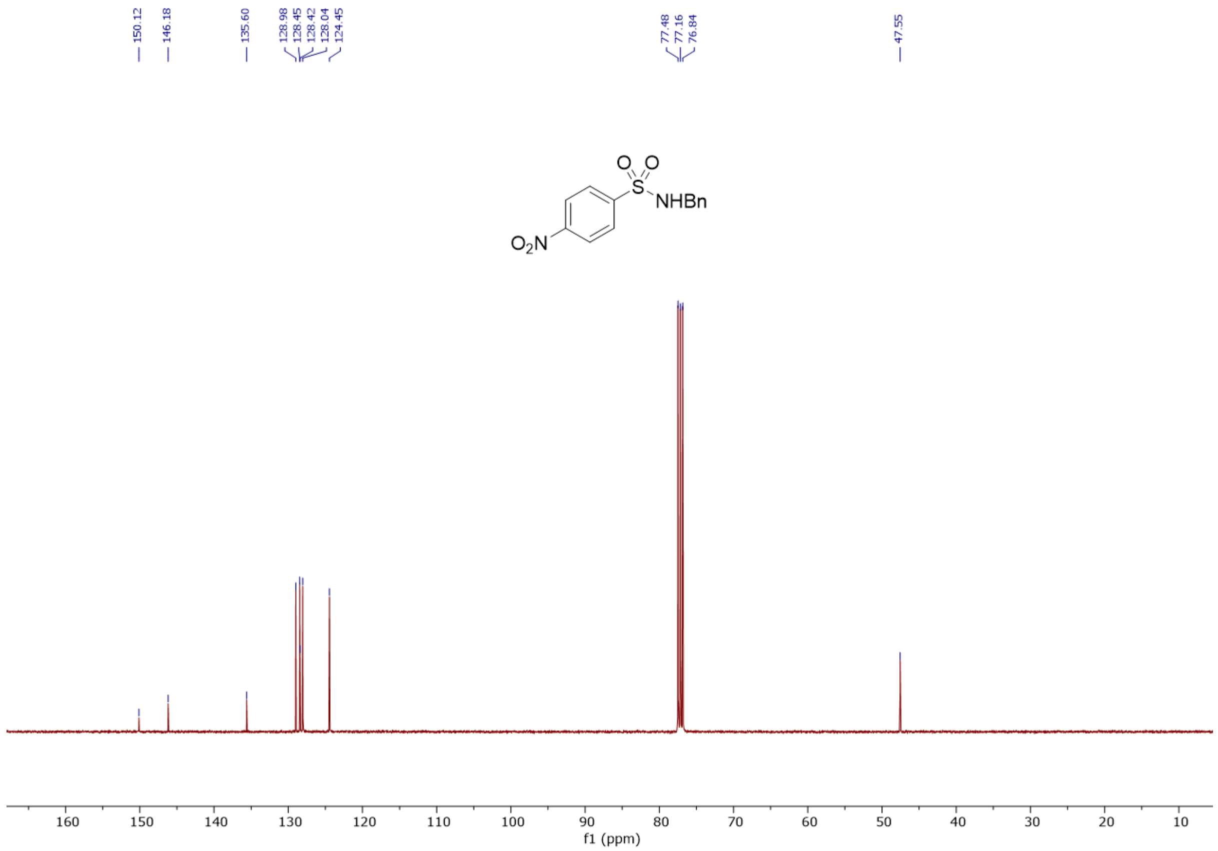

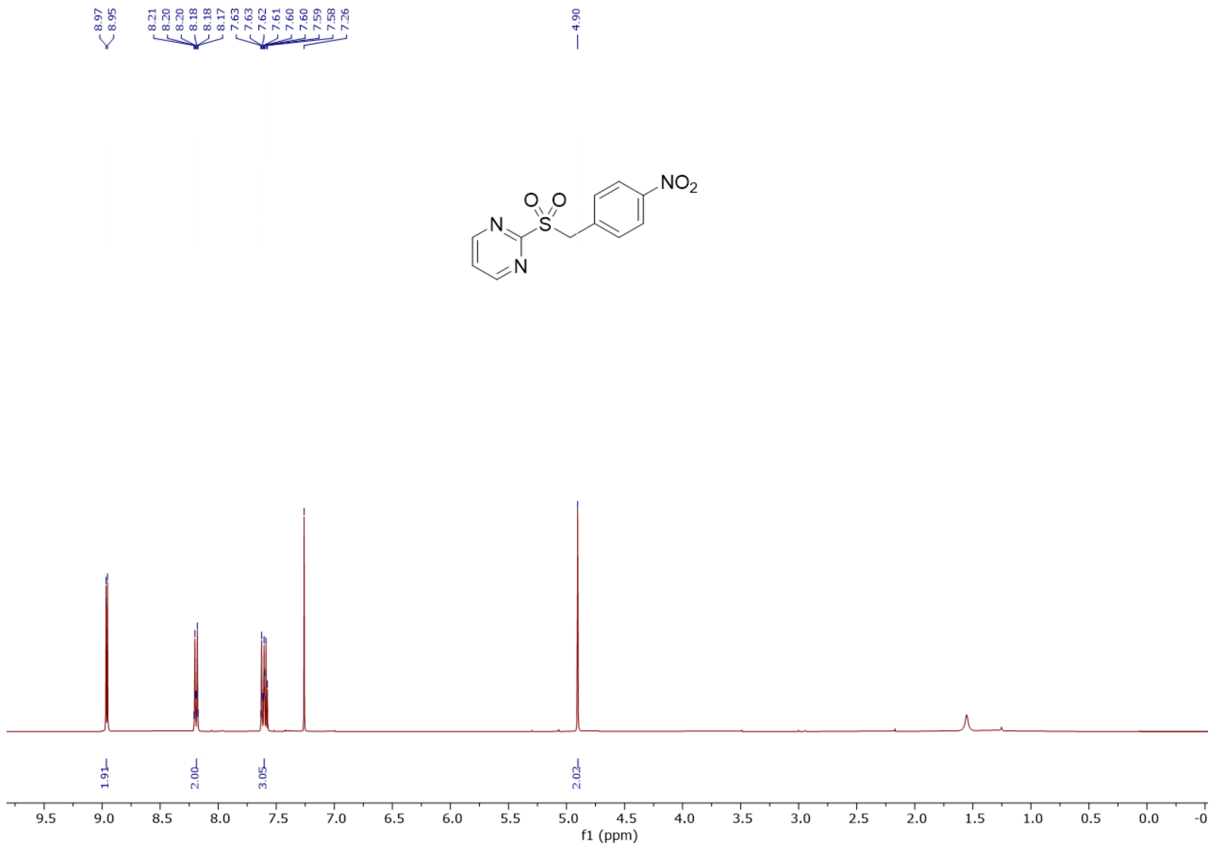

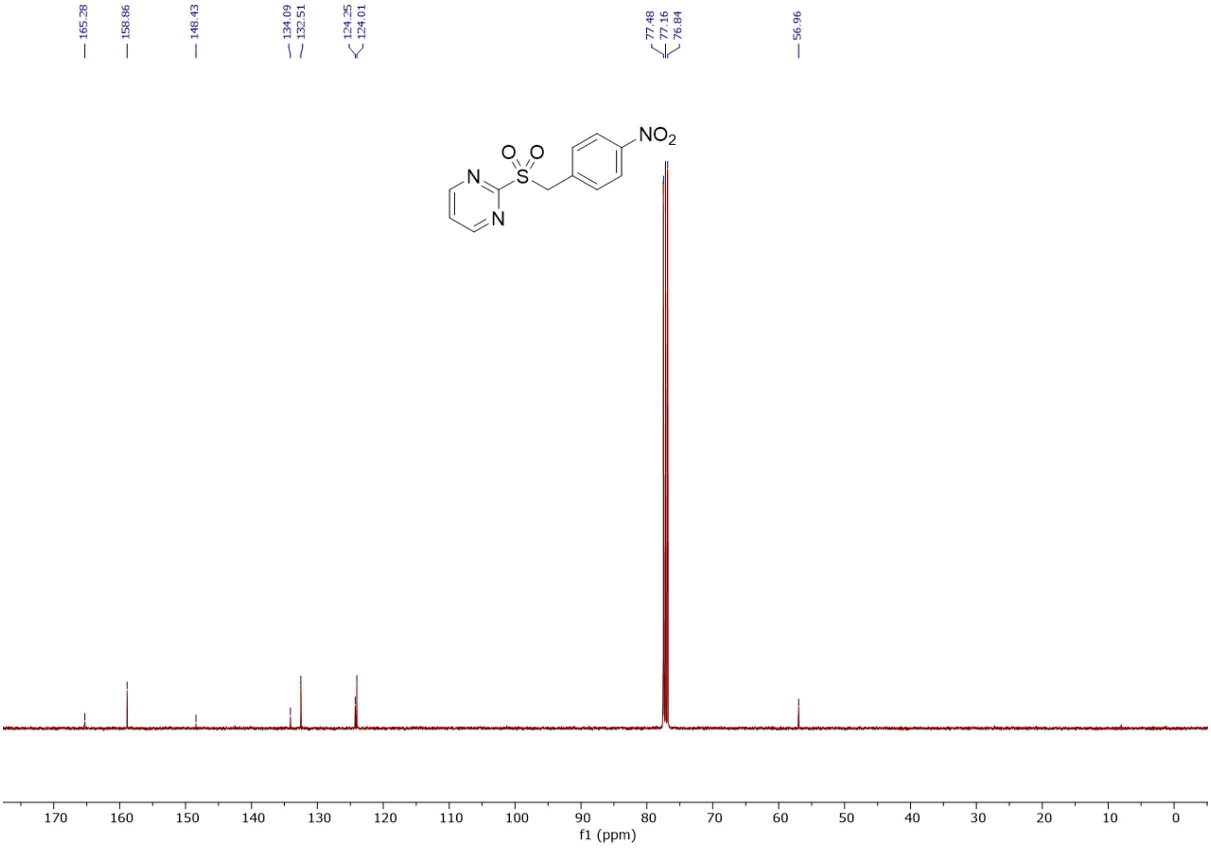
165.28
158.86
148.43
134.09
132.51
124.25
124.01
77.48
77.16
76.84
56.96
NO2
O O
S
N
N
f1 (ppm)

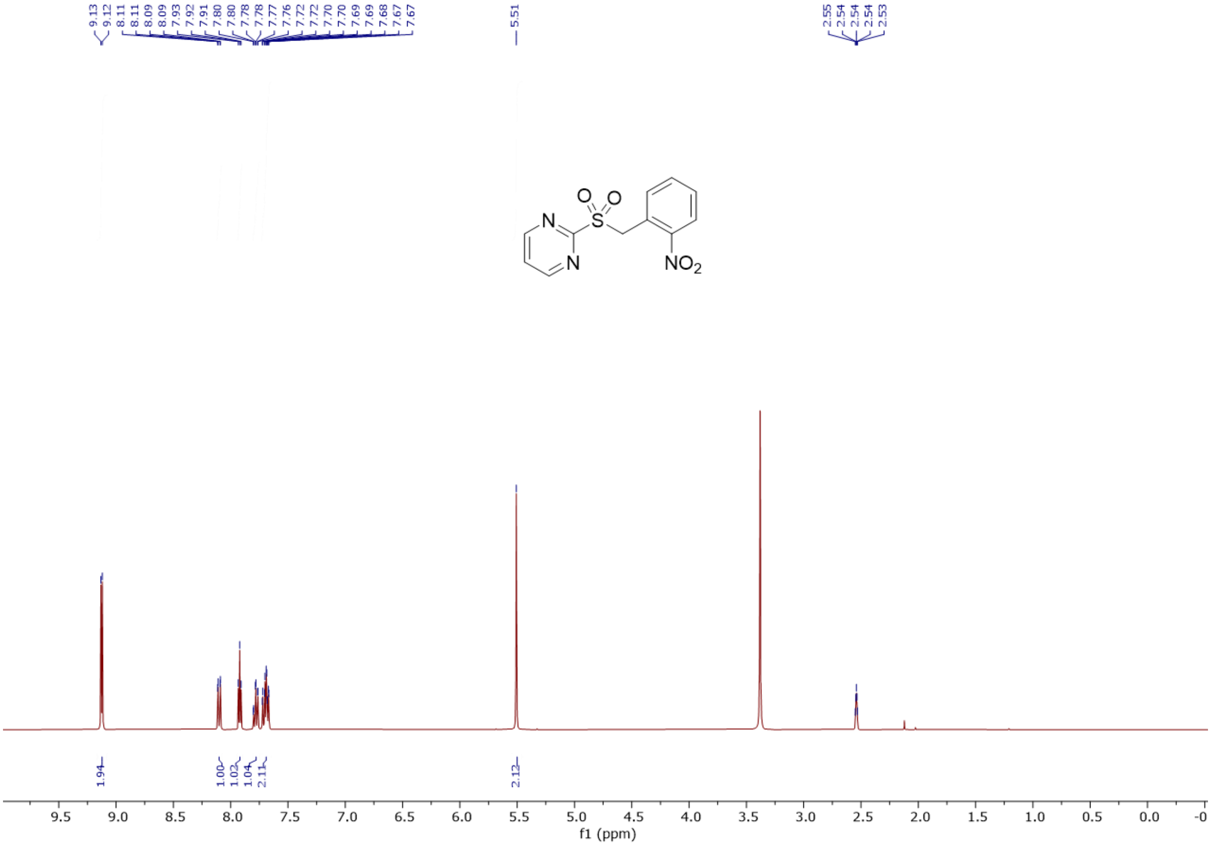
9.13
9.12
8.11
8.11
8.09
8.09
7.93
7.92
7.91
7.80
7.80
7.78
7.77
7.76
7.72
7.72
7.70
7.70
7.69
7.69
7.68
7.67
7.67
5.51
2.55
2.54
2.54
2.54
2.53
O O
S
N
N
NO2
1.94
1.00
1.02
1.04
2.11
2.12
f1 (ppm)

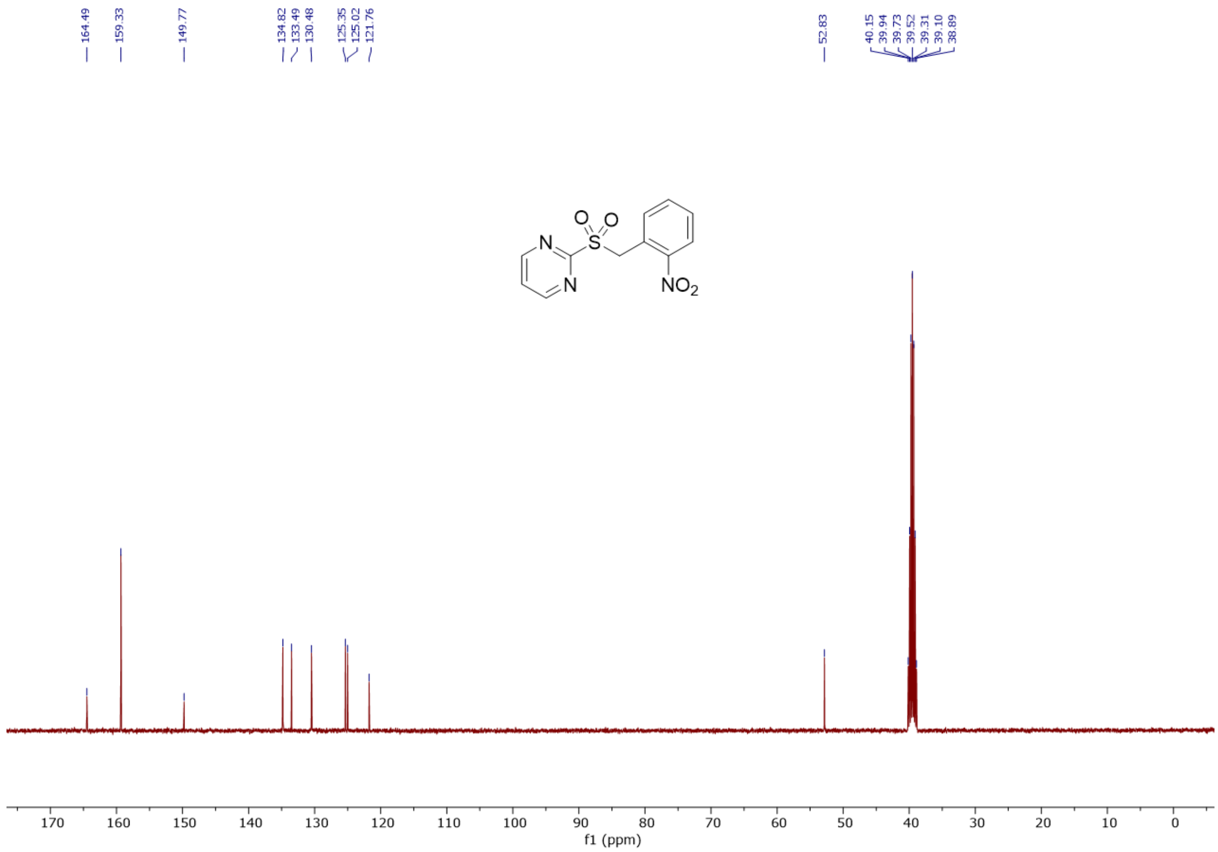

164.49
159.33
149.77
134.82
133.49
130.48
125.35
125.02
121.76
52.83
40.15
39.94
39.73
39.52
39.31
39.10
38.89
N
N
S
O
O
NO2
170
160
150
140
130
120
110
100
90
80
70
60
50
40
30
20
10
0
f1 (ppm)

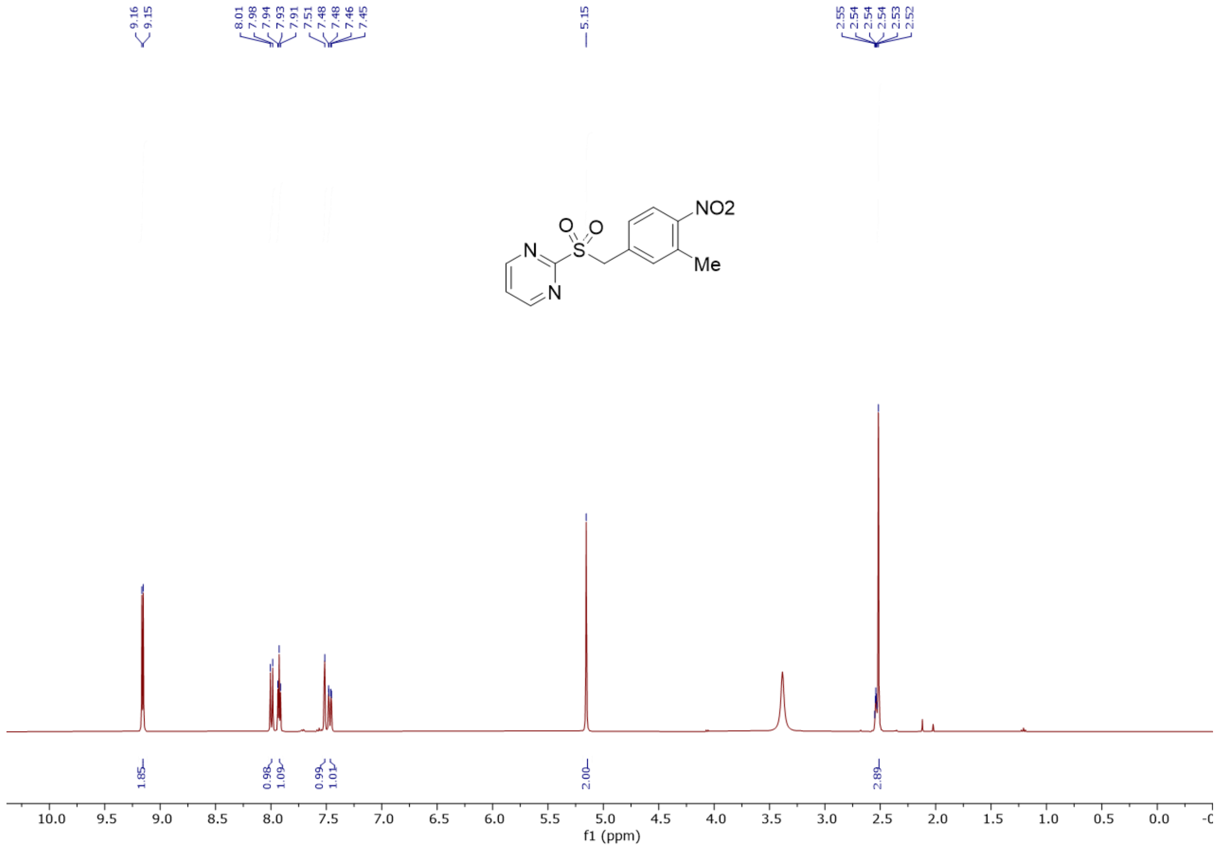

9.16
9.15
8.01
7.98
7.94
7.93
7.91
7.51
7.48
7.48
7.46
7.45
5.15
2.55
2.54
2.54
2.54
2.53
2.52
N
N
S
O
O
NO2
Me
1.85
0.98
1.09
0.99
1.01
2.00
2.89
10.0
9.5
9.0
8.5
8.0
7.5
7.0
6.5
6.0
5.5
5.0
4.5
4.0
3.5
3.0
2.5
2.0
1.5
1.0
0.5
0.0
f1 (ppm)

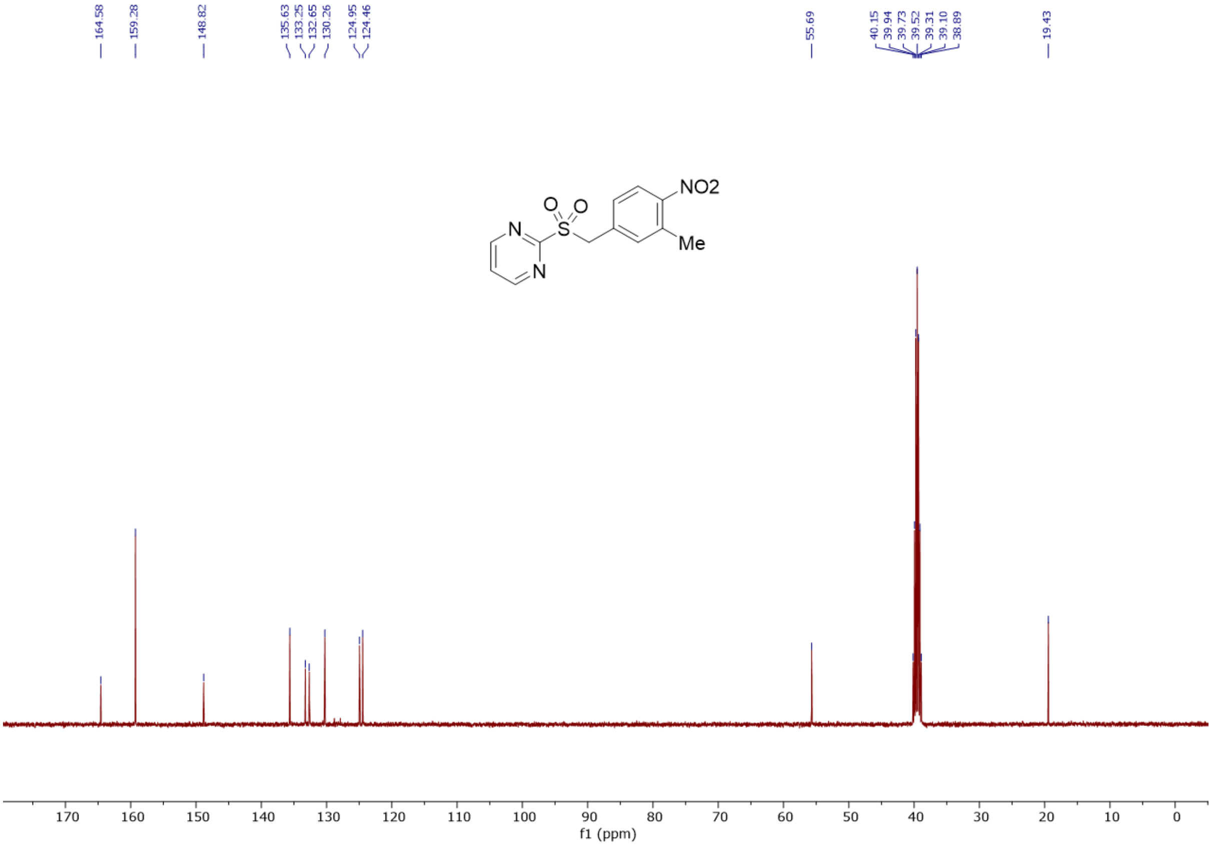
164.58
159.28
148.82
135.63
133.25
132.65
130.26
124.95
124.46
55.69
40.15
39.94
39.73
39.52
39.31
39.10
38.89
19.43
NO2
O
O
S
N
N
Me
170 160 150 140 130 120 110 100 90 80 70 60 50 40 30 20 10 0
f1 (ppm)

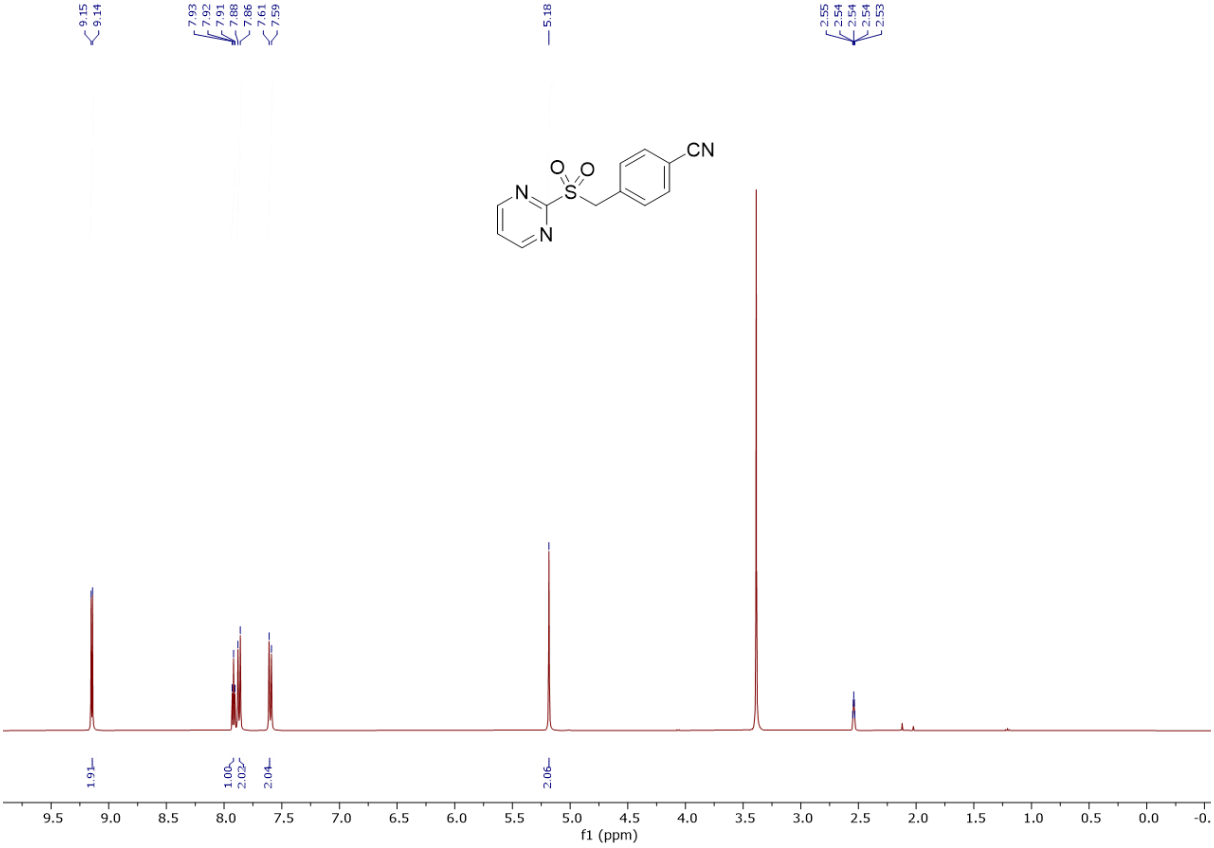
9.15
9.14
7.93
7.92
7.91
7.88
7.86
7.61
7.59
5.18
2.55
2.54
2.54
2.54
2.53
CN
O
O
S
N
N
1.91
1.00
2.02
2.04
2.06
9.5 9.0 8.5 8.0 7.5 7.0 6.5 6.0 5.5 5.0 4.5 4.0 3.5 3.0 2.5 2.0 1.5 1.0 0.5 0.0 -0.
f1 (ppm)

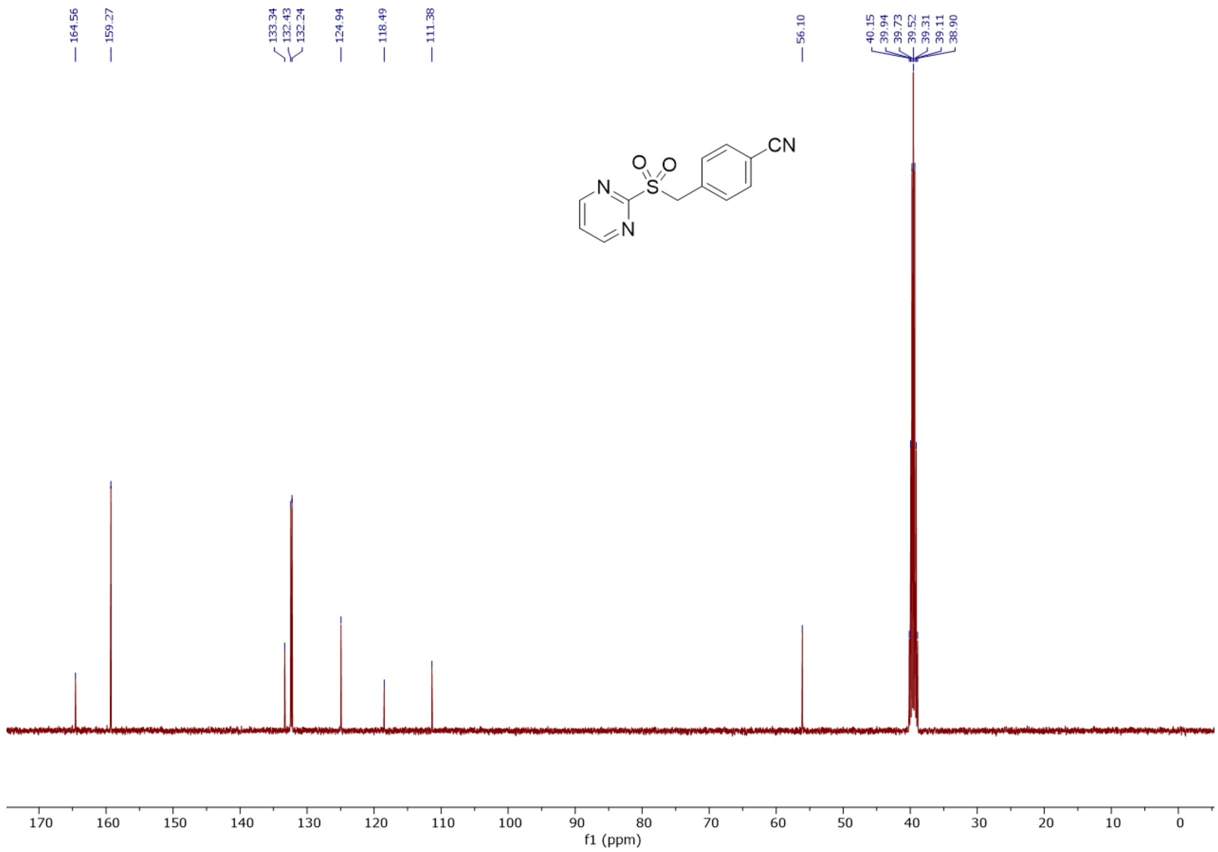
164.56
159.27
133.34
132.43
132.24
124.94
118.49
111.38
56.10
40.15
39.94
39.73
39.52
39.31
39.11
38.90
CN
O
O
S
N
N
f1 (ppm)

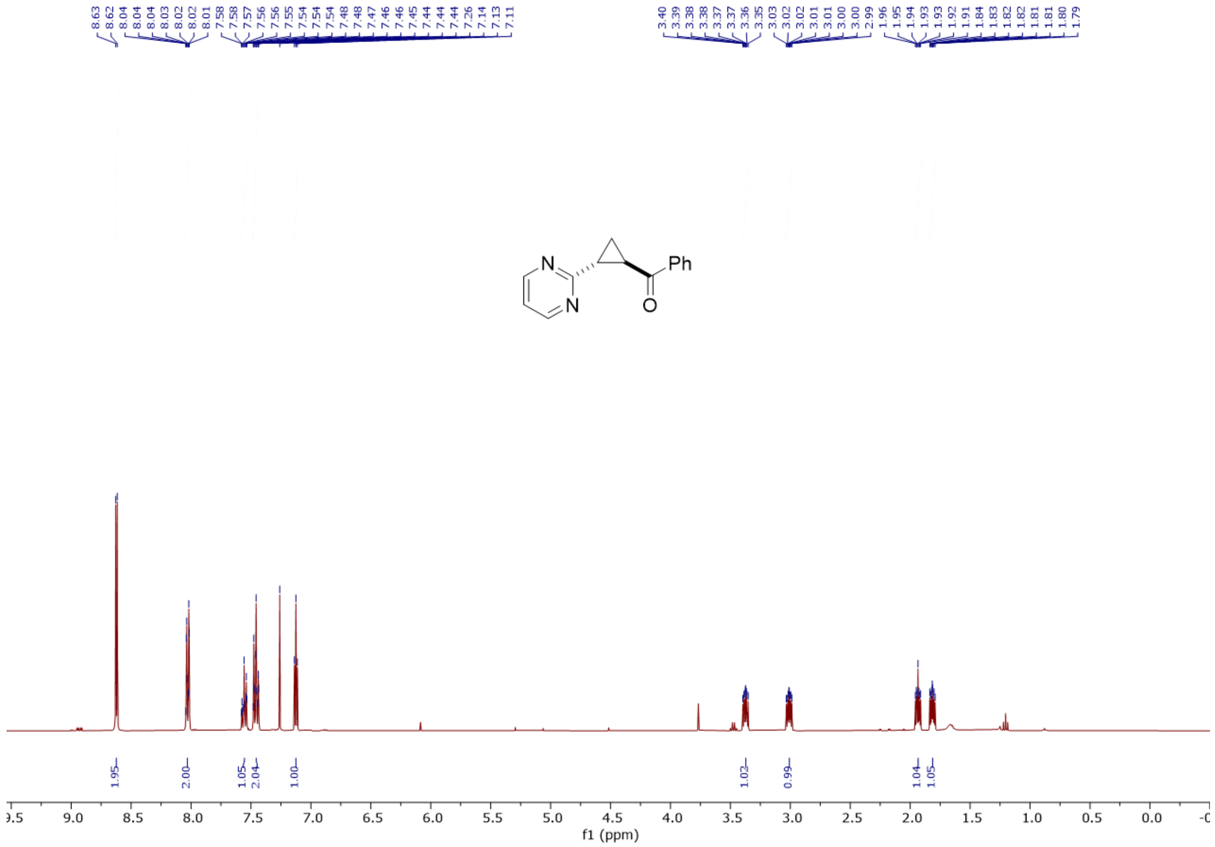
N
N
Ph
O
f1 (ppm)

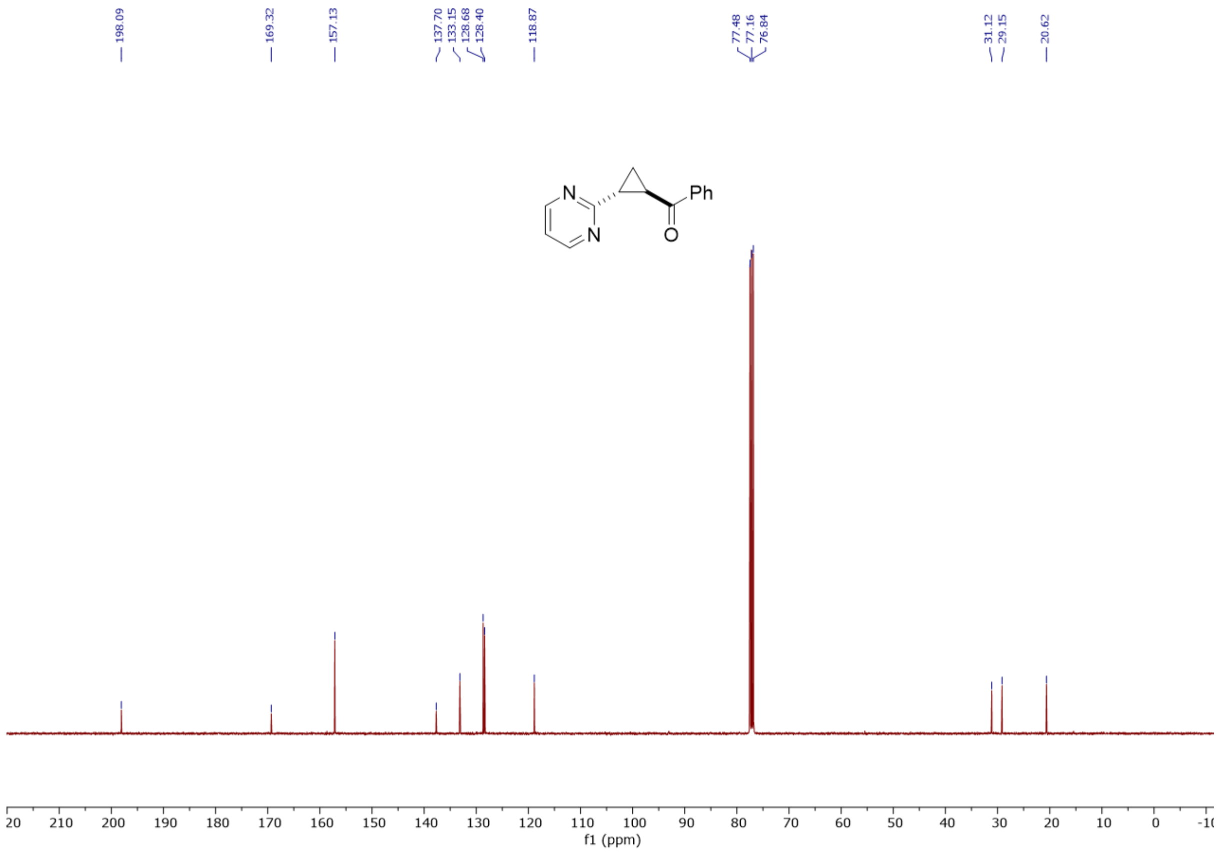
198.09
169.32
157.13
137.70
133.15
128.68
128.40
118.87
77.48
77.16
76.84
31.12
29.15
20.62
Ph
N
N
O
f1 (ppm)

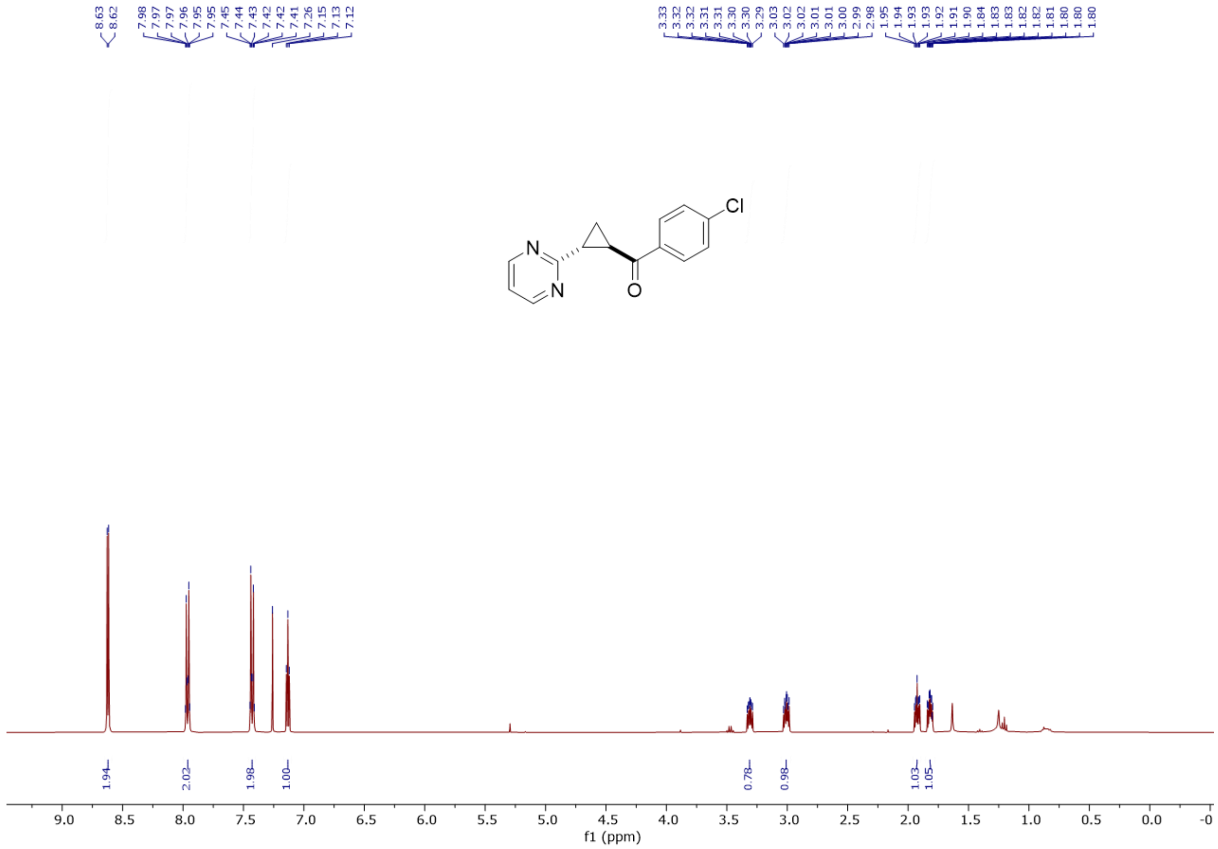
8.63
8.62
7.98
7.97
7.97
7.96
7.95
7.95
7.45
7.44
7.43
7.42
7.42
7.41
7.26
7.15
7.13
7.12
3.33
3.32
3.32
3.31
3.31
3.30
3.30
3.29
3.03
3.02
3.02
3.01
3.01
3.00
2.99
2.98
1.95
1.94
1.93
1.93
1.92
1.91
1.90
1.84
1.83
1.83
1.82
1.82
1.81
1.80
1.80
1.80
Cl
N
N
O
1.94
2.03
1.98
1.00
0.78
0.98
1.03
1.05
f1 (ppm)

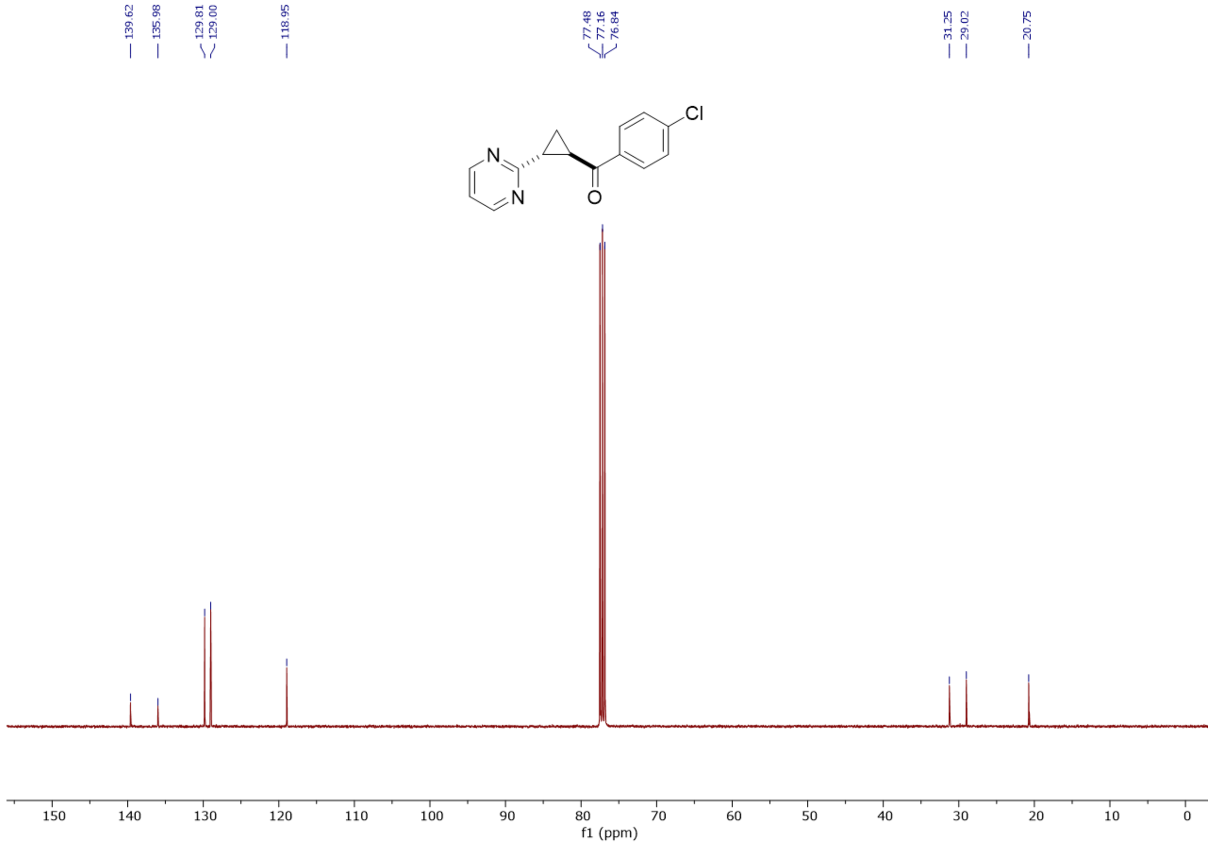

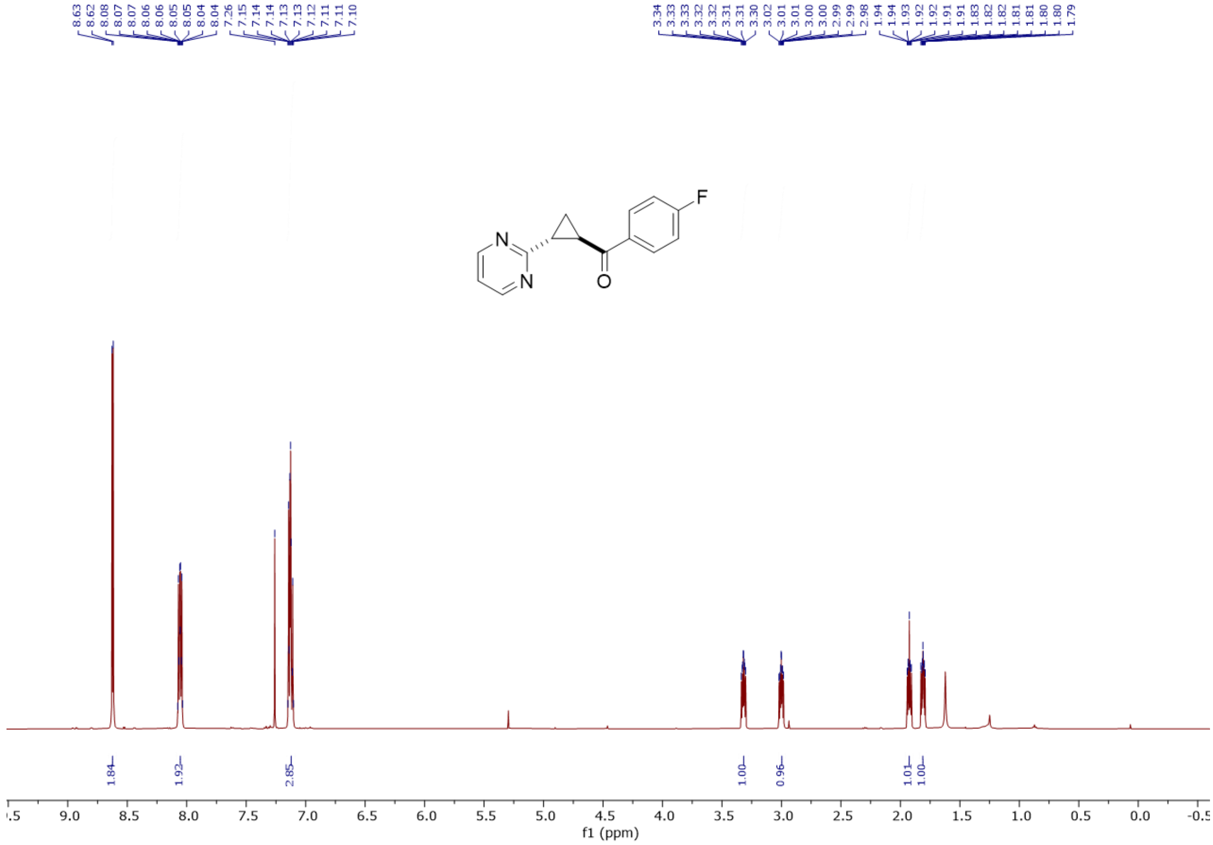

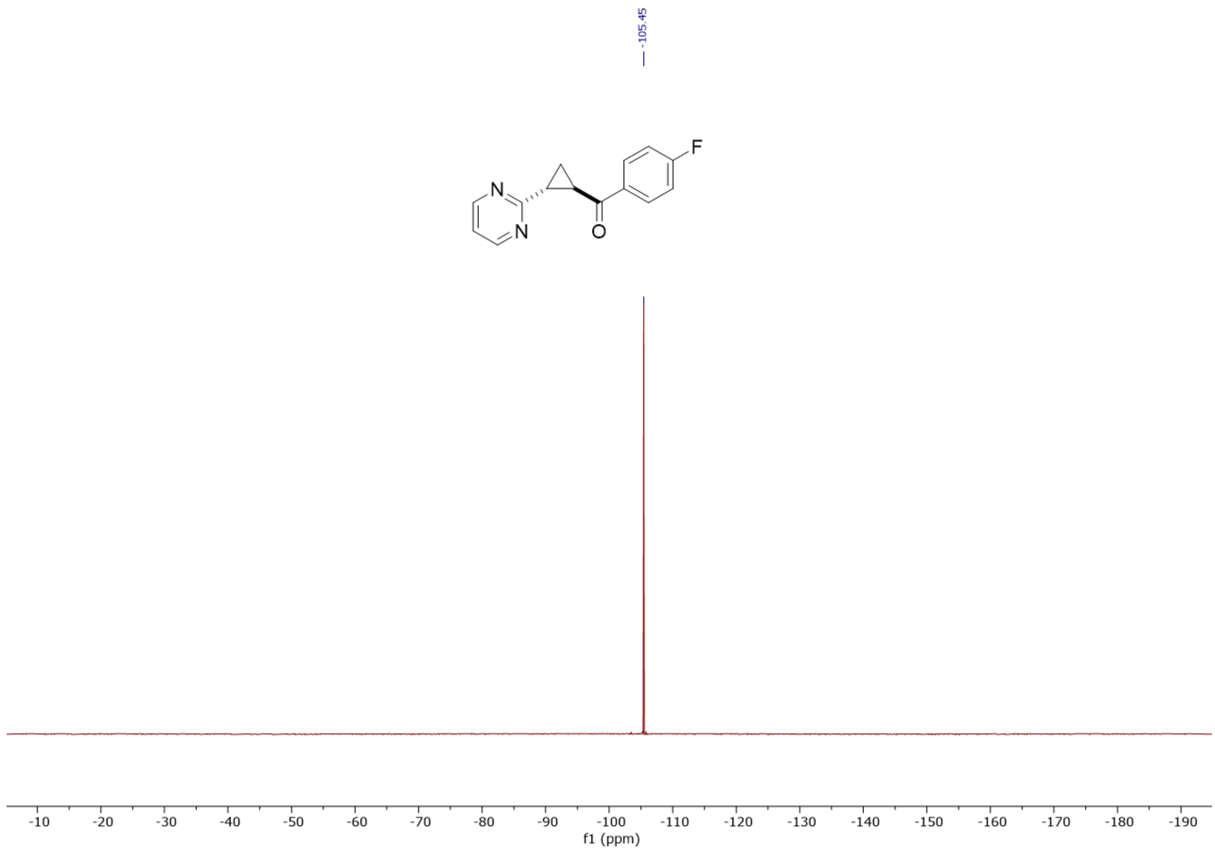
-105.45
F
N
N
O
-10
-20
-30
-40
-50
-60
-70
-80
-90
-100
-110
-120
-130
-140
-150
-160
-170
-180
-190
f1 (ppm)

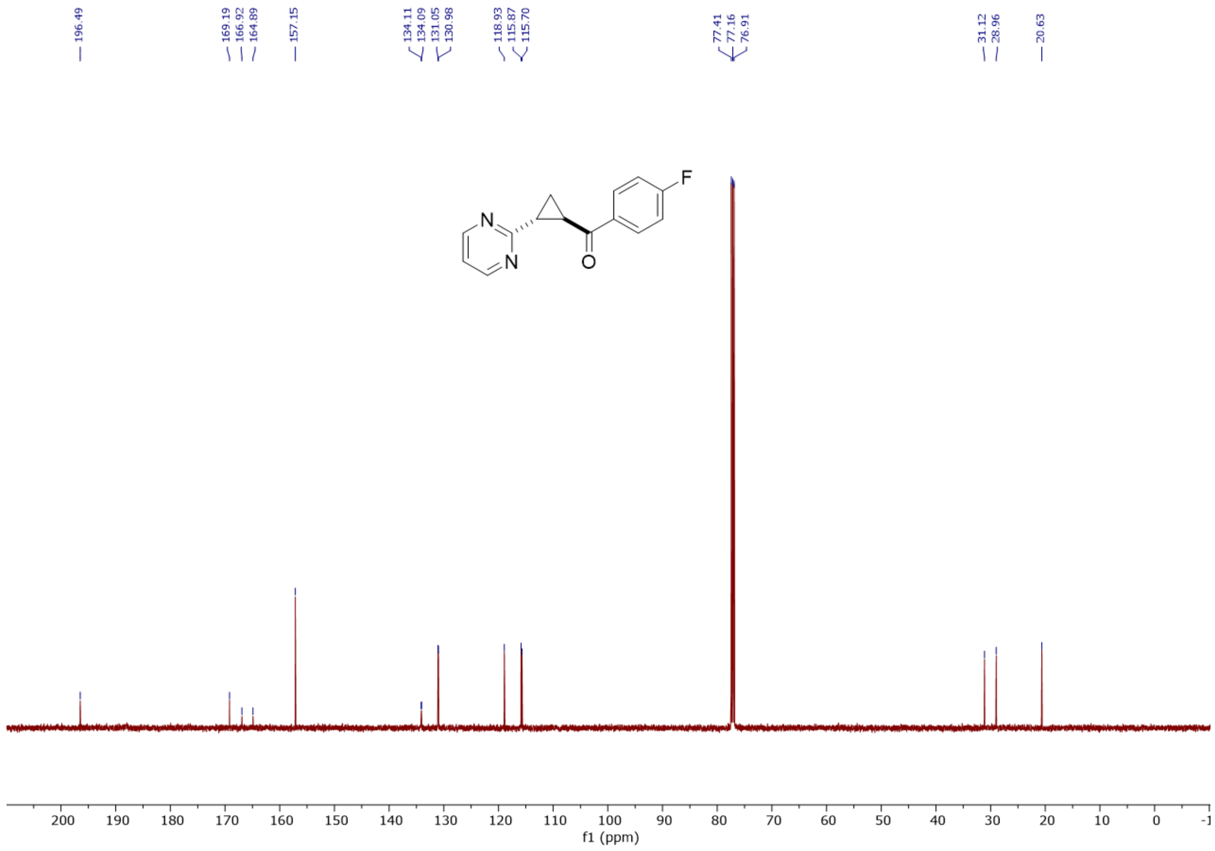
196.49
169.19
166.92
164.89
157.15
134.11
134.09
131.05
130.98
118.93
115.87
115.70
77.41
77.16
76.91
31.12
28.96
20.63
F
N
N
O
200
190
180
170
160
150
140
130
120
110
100
90
80
70
60
50
40
30
20
10
0
f1 (ppm)

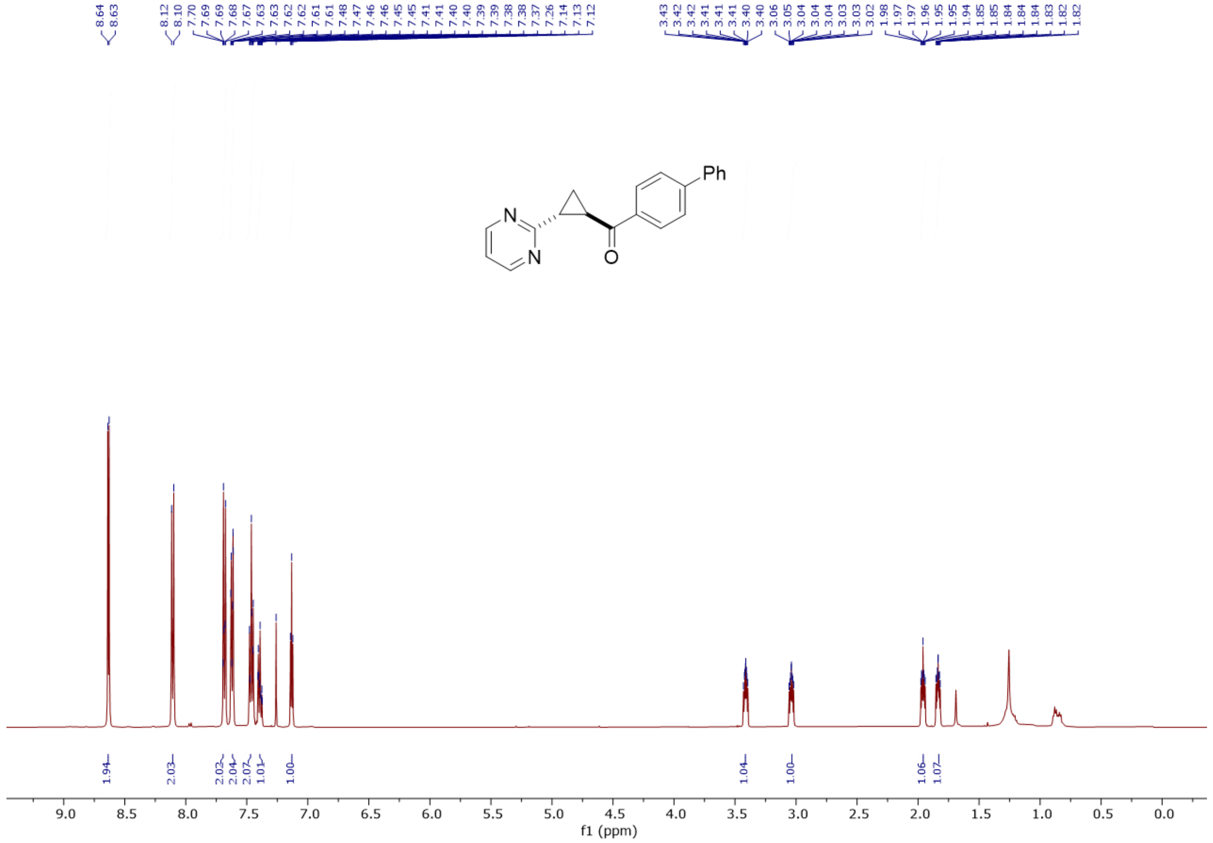
8.64
8.63
8.12
8.10
7.70
7.69
7.68
7.67
7.63
7.63
7.62
7.61
7.61
7.48
7.47
7.46
7.46
7.45
7.45
7.41
7.41
7.40
7.40
7.39
7.39
7.38
7.38
7.37
7.26
7.14
7.13
7.12
3.43
3.42
3.42
3.41
3.41
3.41
3.40
3.40
3.06
3.05
3.04
3.04
3.04
3.03
3.03
3.02
1.98
1.97
1.97
1.96
1.95
1.95
1.94
1.85
1.85
1.84
1.84
1.84
1.83
1.82
1.82
Ph
N
N
O
1.94
2.03
2.02
2.04
2.07
1.01
1.00
1.04
1.00
1.06
1.07
9.0 8.5 8.0 7.5 7.0 6.5 6.0 5.5 5.0 4.5 4.0 3.5 3.0 2.5 2.0 1.5 1.0 0.5 0.0
f1 (ppm)

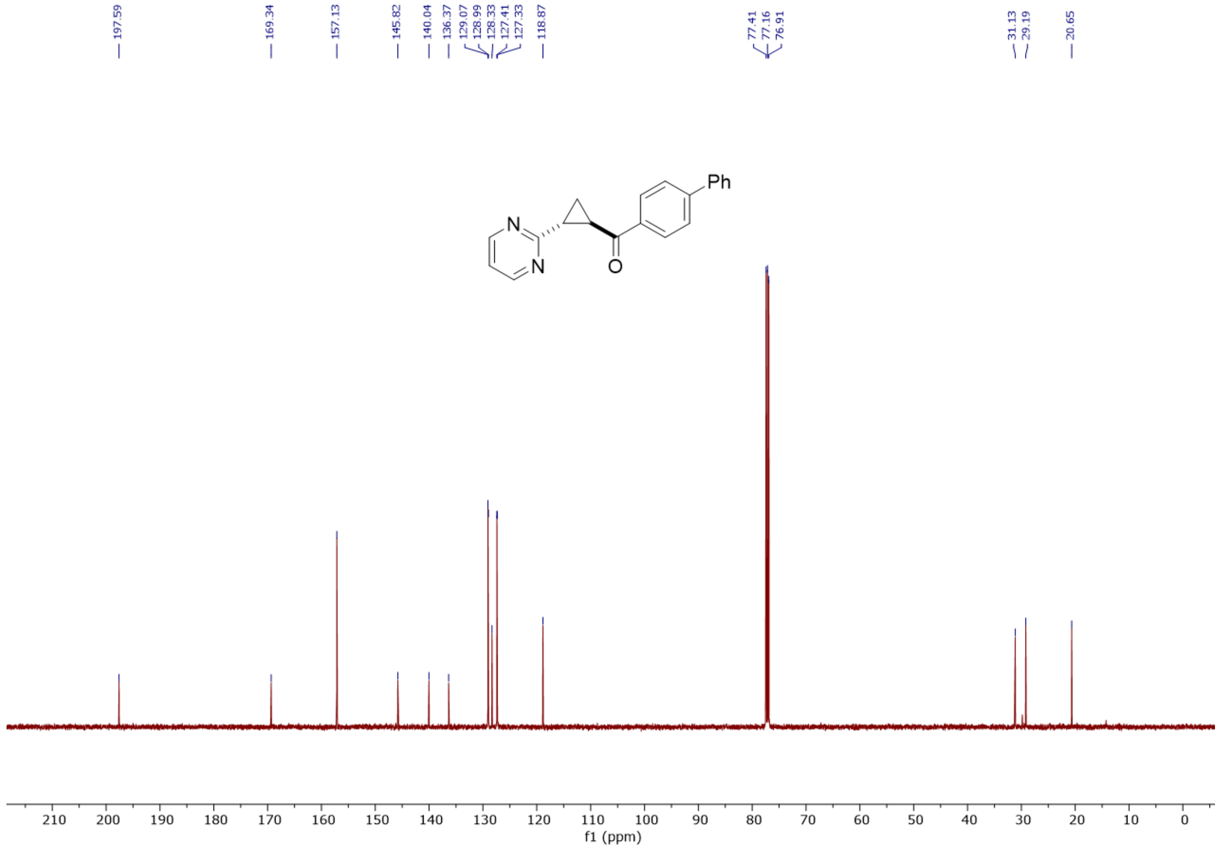
197.59
169.34
157.13
145.82
140.04
136.37
129.07
128.99
128.33
127.41
127.33
118.87
77.41
77.16
76.91
31.13
29.19
20.65
Ph
N
N
O
210 200 190 180 170 160 150 140 130 120 110 100 90 80 70 60 50 40 30 20 10 0
f1 (ppm)

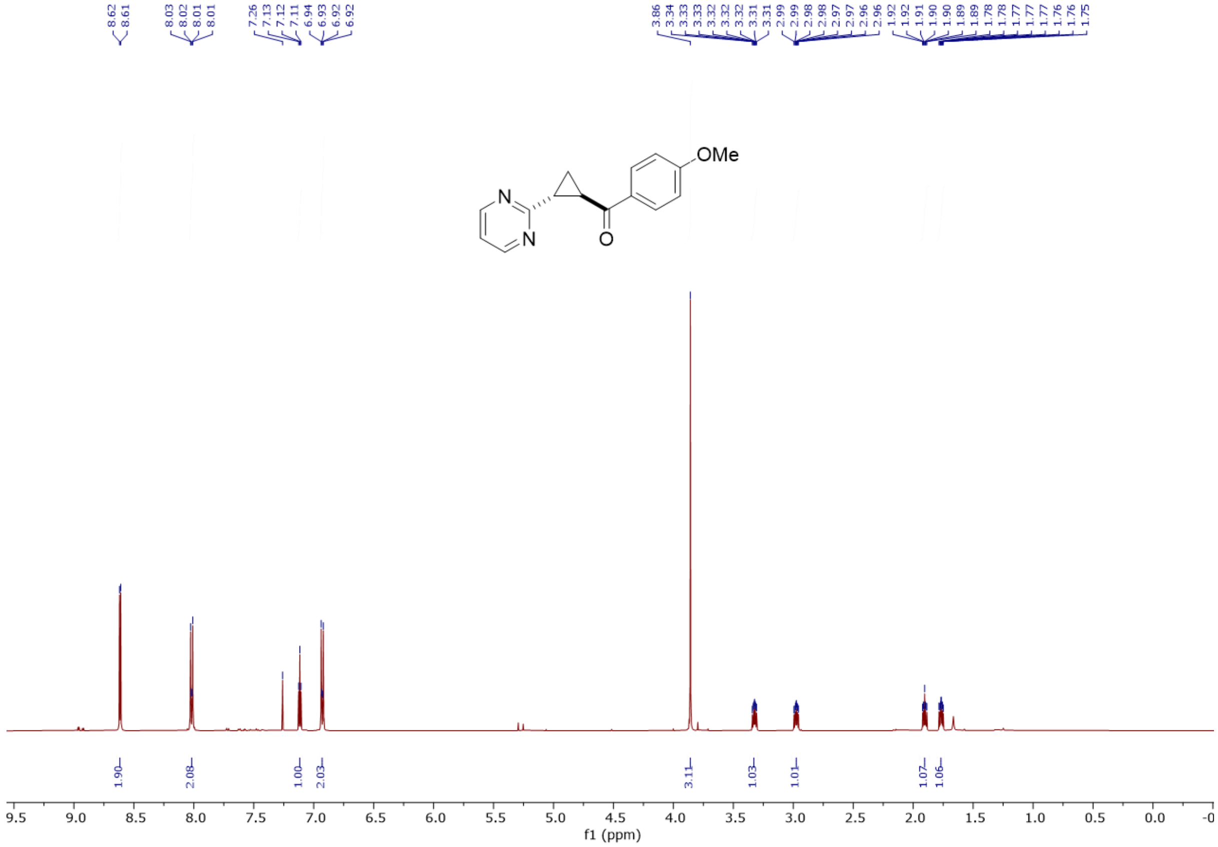
OMe
N
N
O
f1 (ppm)

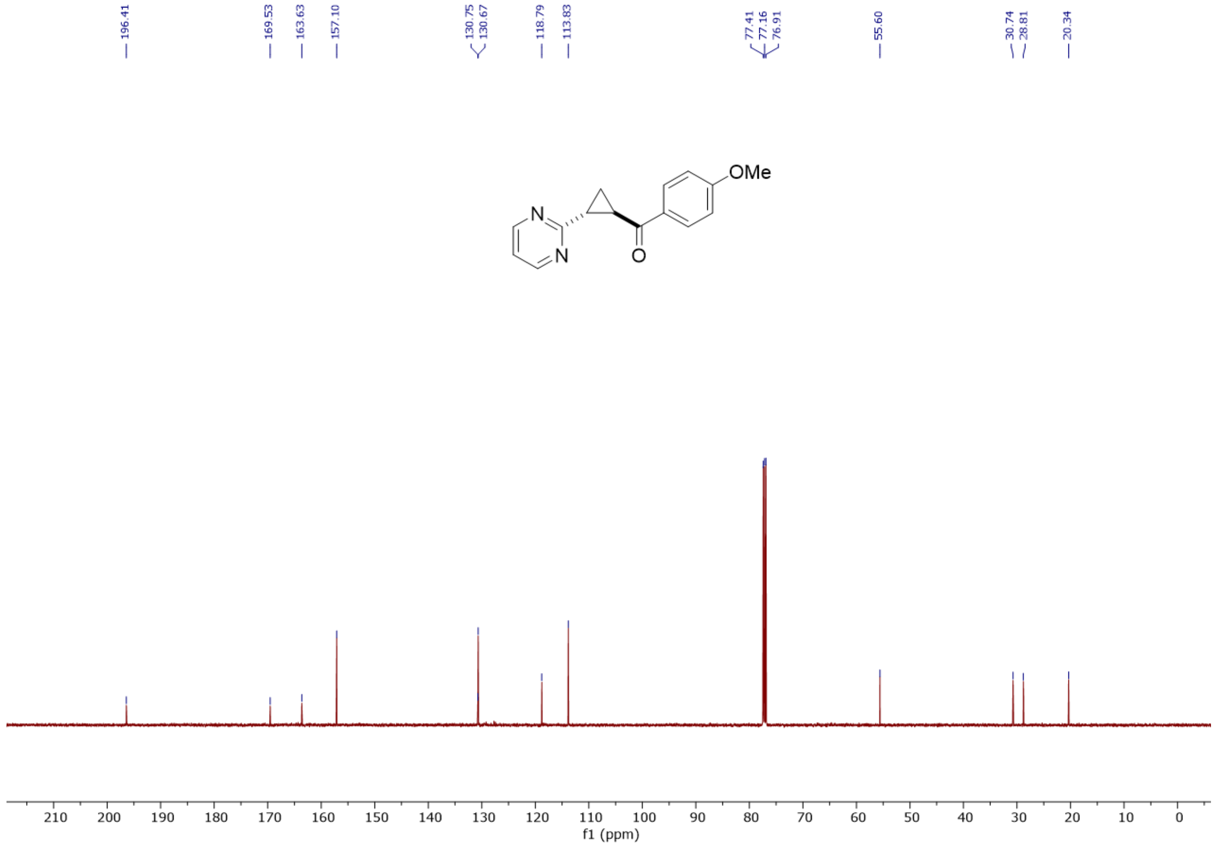
OMe
N
N
O
f1 (ppm)

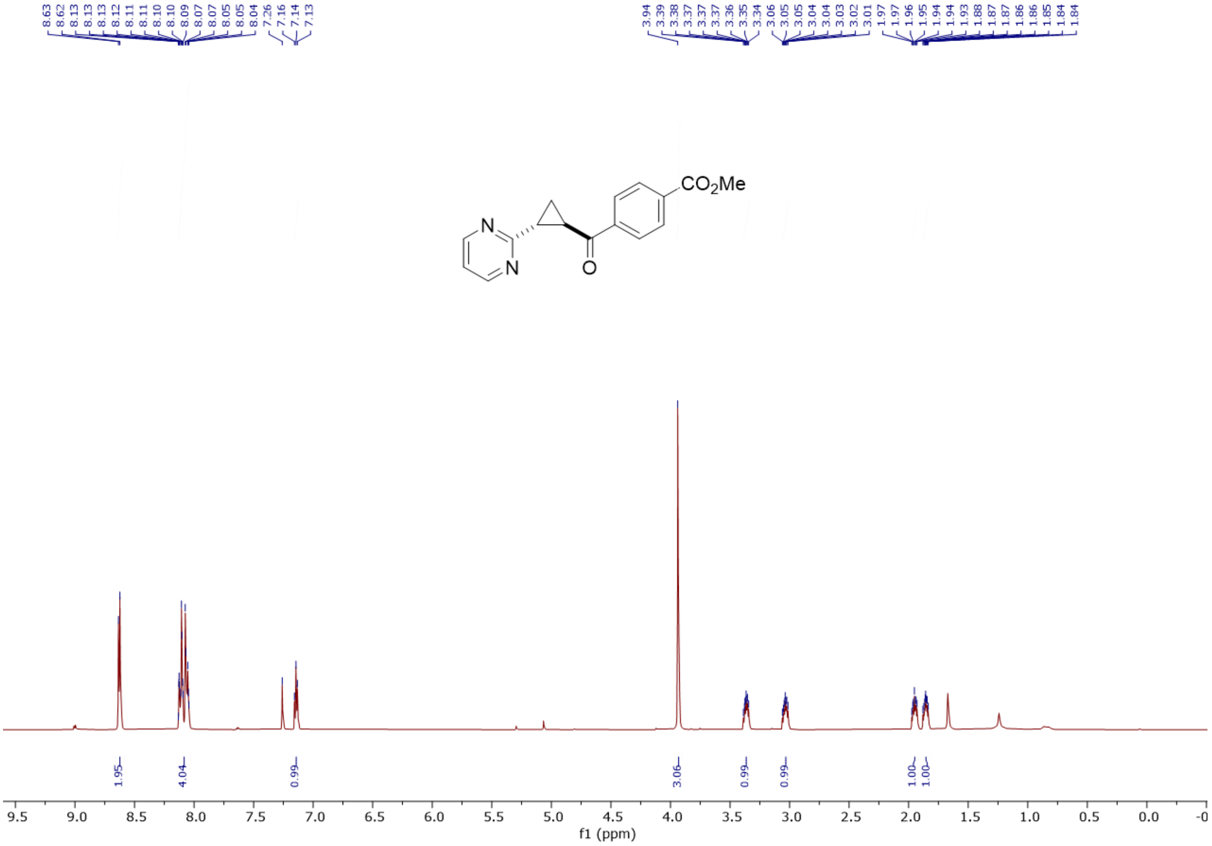
8.63 8.62 8.13 8.13 8.13 8.12 8.11 8.11 8.10 8.09 8.07 8.05 8.05 8.04 7.26 7.16 7.14 7.13
3.94 3.39 3.38 3.37 3.37 3.37 3.36 3.35 3.34 3.06 3.05 3.05 3.04 3.04 3.03 3.02 3.01 1.97 1.97 1.96 1.95 1.94 1.94 1.93 1.88 1.87 1.87 1.86 1.86 1.85 1.84 1.84
CO2Me
N
N
O
1.95
4.04
0.99
3.06
0.99
0.99
1.00
1.00
f1 (ppm)

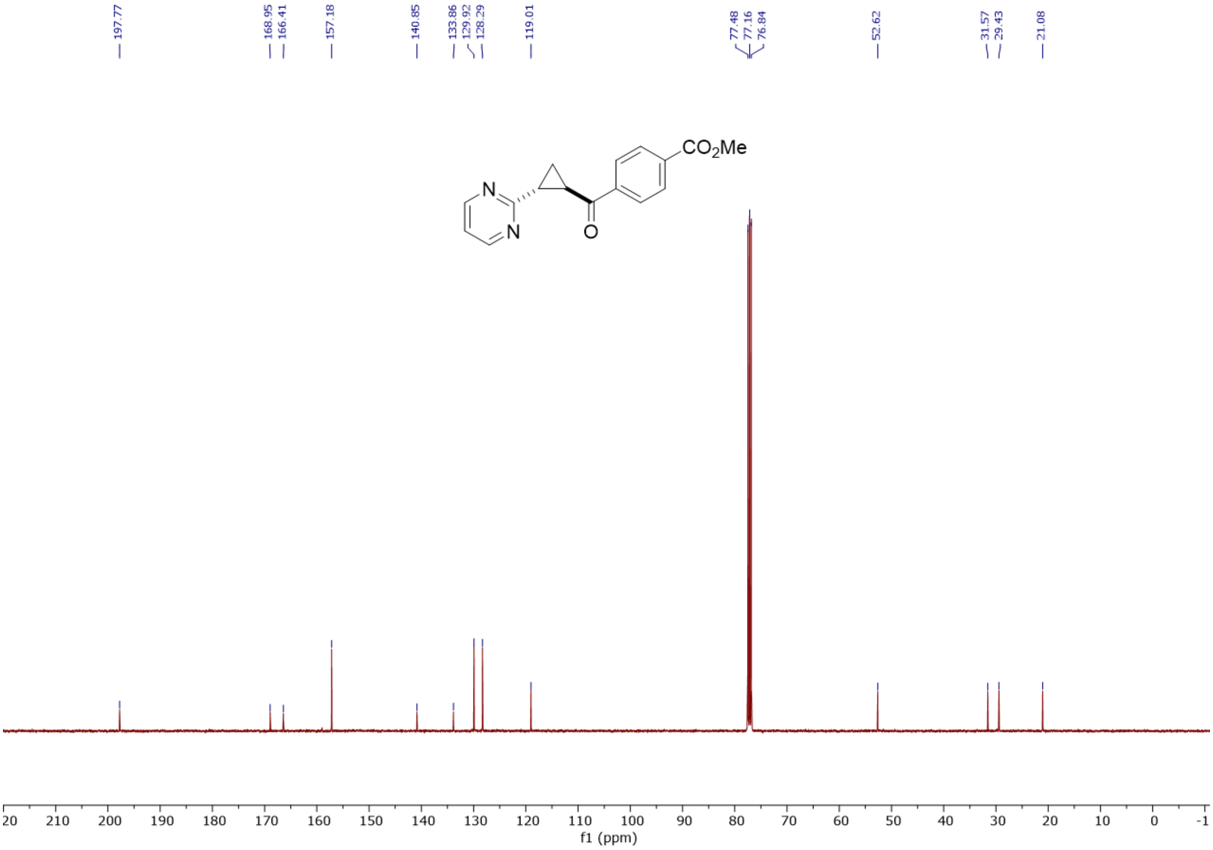
197.77
168.95 166.41
157.18
140.85
133.86 129.92 128.29
119.01
77.48 77.16 76.84
52.62
31.57 29.43
21.08
CO2Me
N
N
O
f1 (ppm)

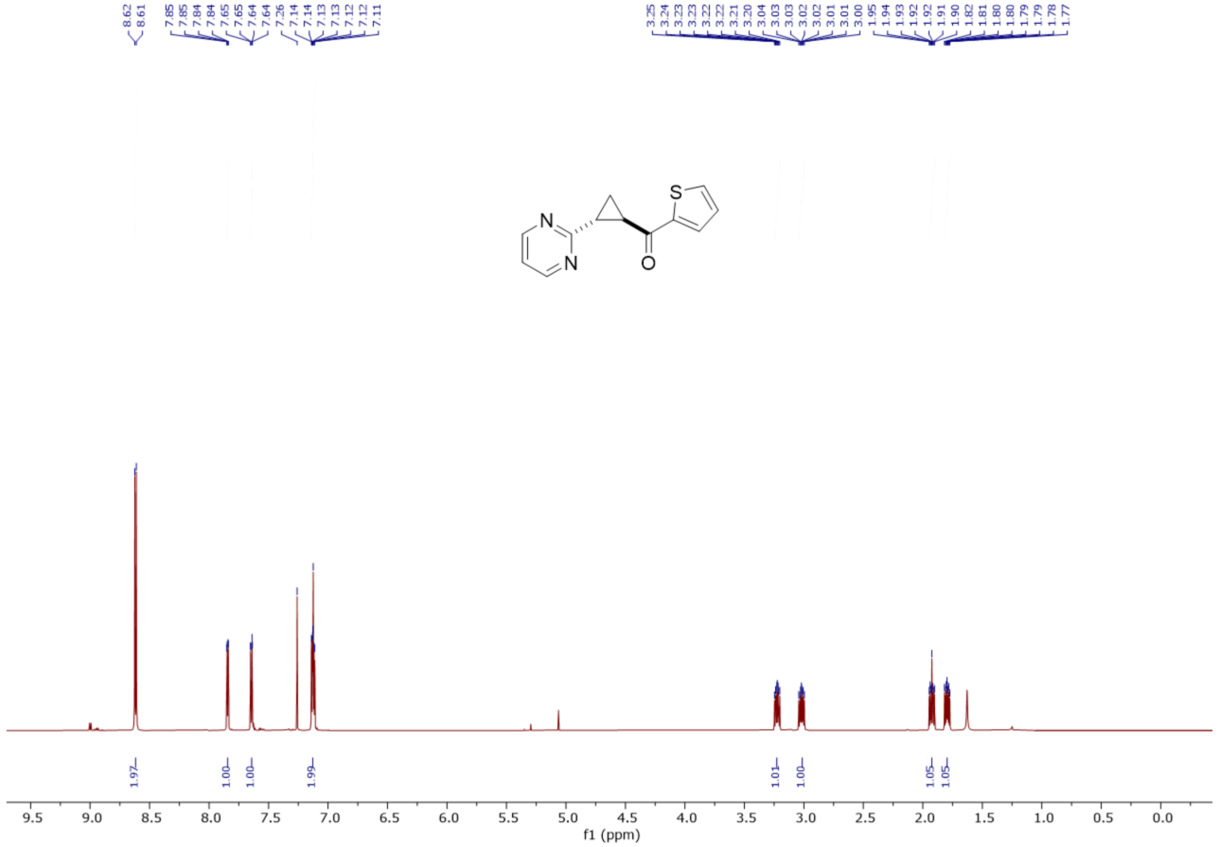
8.62
8.61
7.85
7.85
7.84
7.84
7.65
7.65
7.64
7.64
7.26
7.14
7.14
7.13
7.13
7.12
7.12
7.11
3.25
3.24
3.23
3.23
3.22
3.22
3.21
3.20
3.04
3.03
3.03
3.02
3.02
3.01
3.01
3.00
1.95
1.94
1.93
1.92
1.92
1.91
1.90
1.82
1.81
1.80
1.80
1.79
1.79
1.78
1.77
N
N
S
O
1.97
1.00
1.00
1.99
1.01
1.00
1.05
1.05
f1 (ppm)

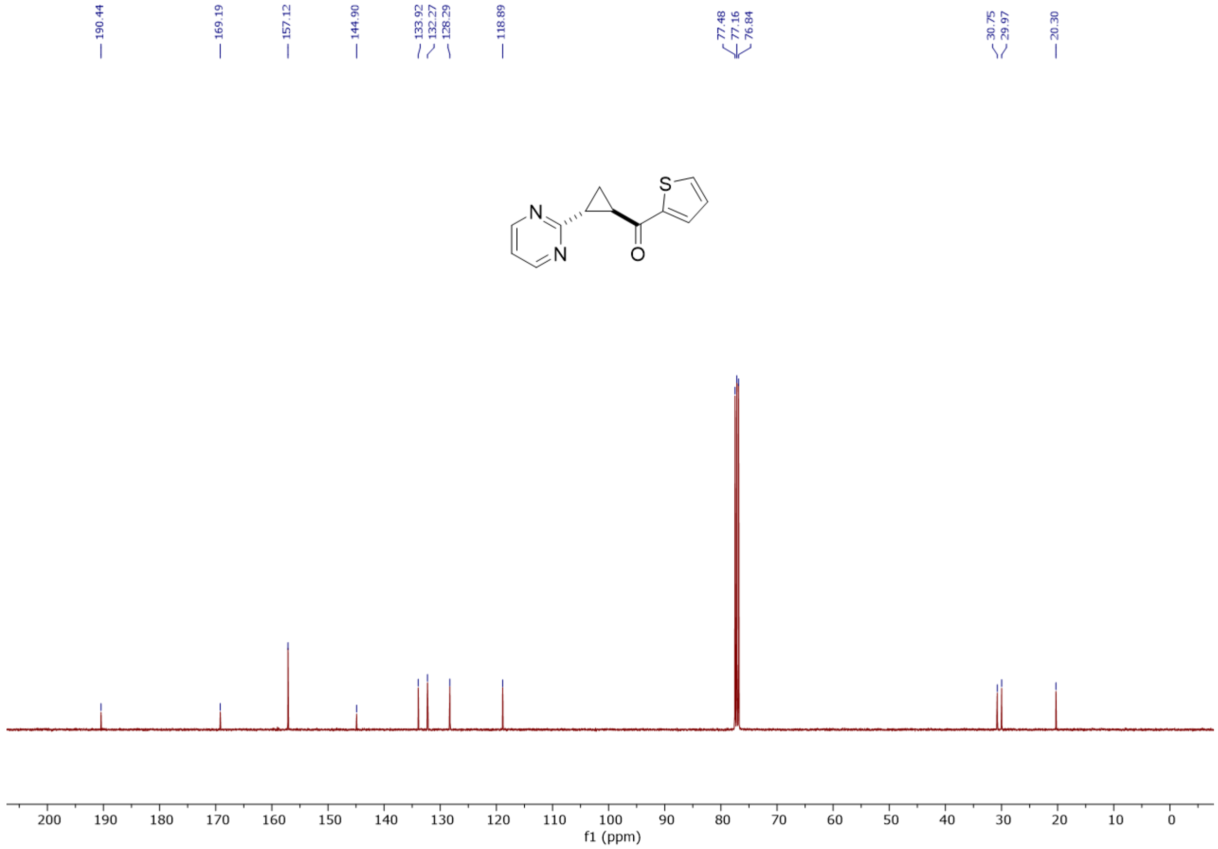
190.44
169.19
157.12
144.90
133.92
132.27
128.29
118.89
77.48
77.16
76.84
30.75
29.97
20.30
N
N
S
O
f1 (ppm)

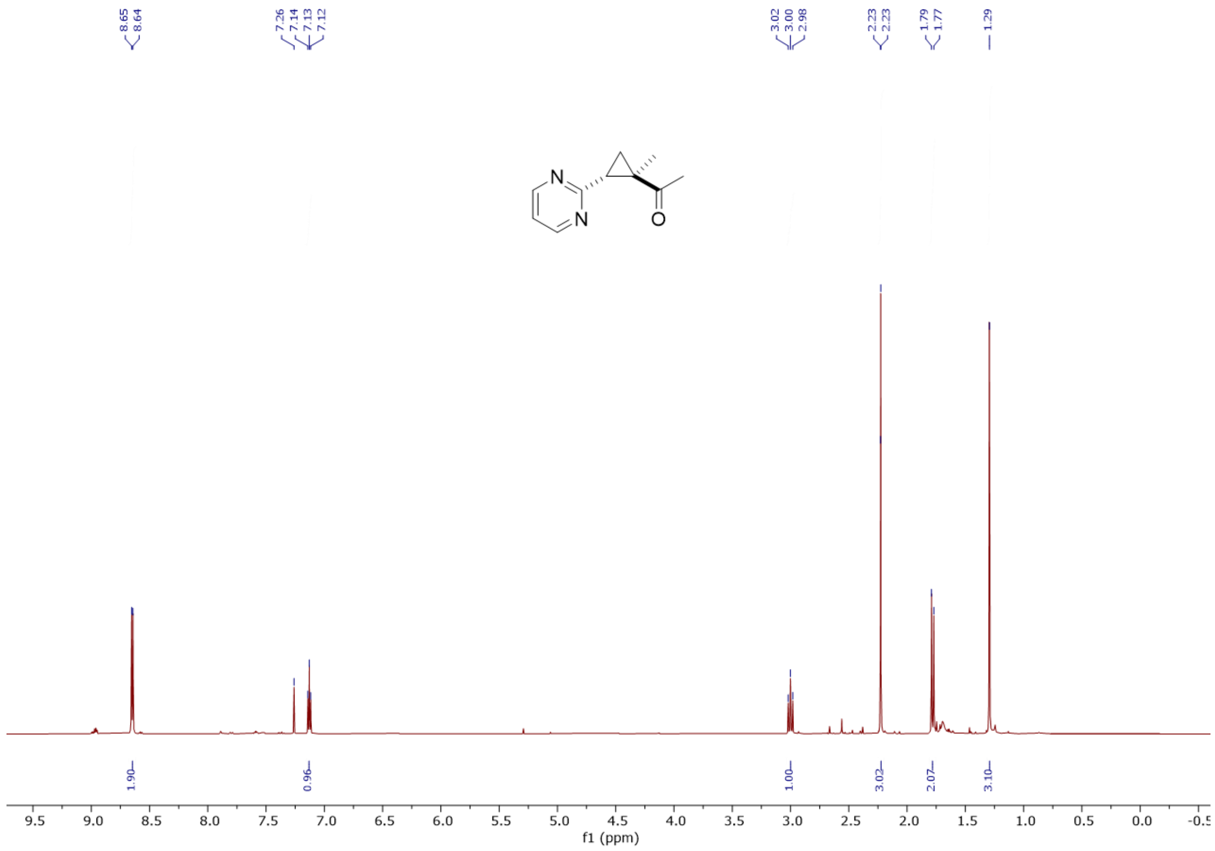
8.65
8.64
7.26
7.14
7.13
7.12
3.02
3.00
2.98
2.23
2.23
1.79
1.77
1.29
N
N
O
1.90
0.96
1.00
3.02
2.07
3.10
f1 (ppm)

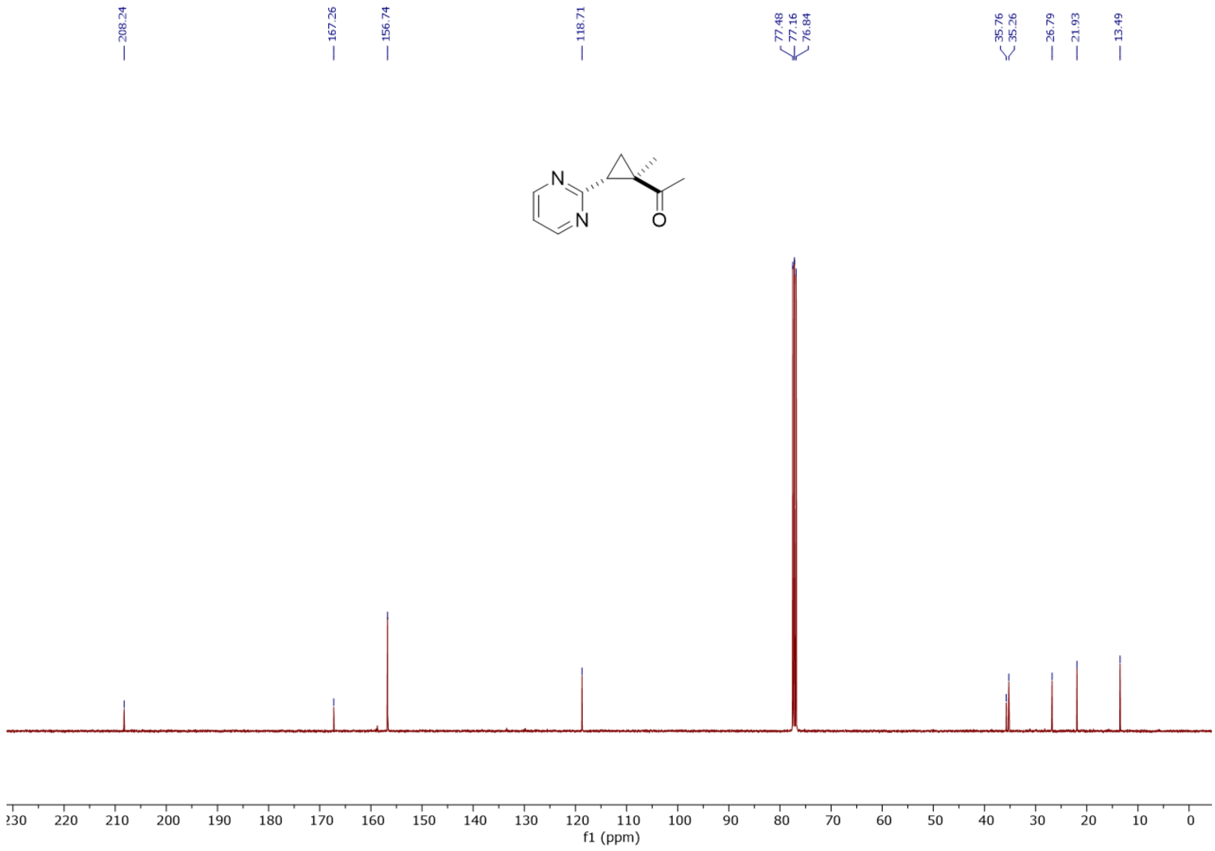
208.24
167.26
156.74
118.71
77.48
77.16
76.84
35.76
35.26
26.79
21.93
13.49
N
N
O
f1 (ppm)

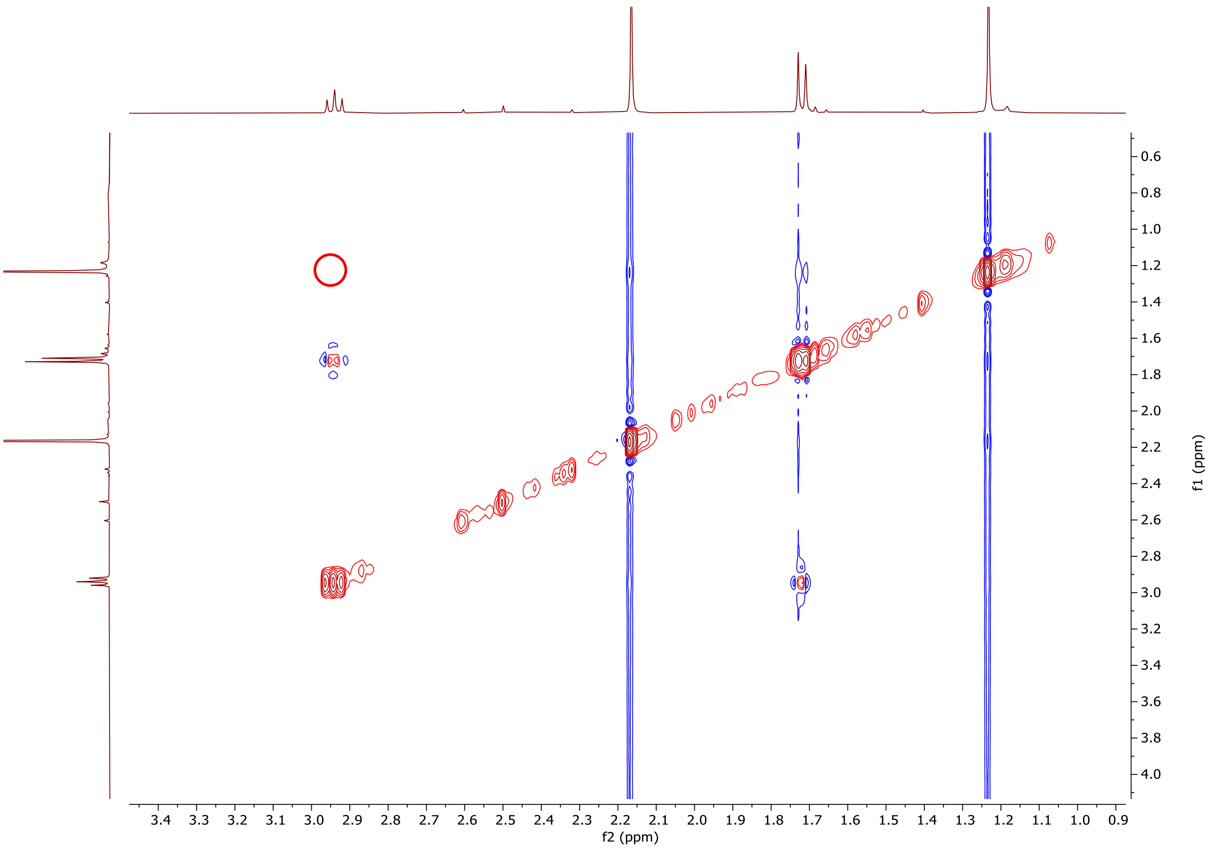
f2 (ppm)
f1 (ppm)

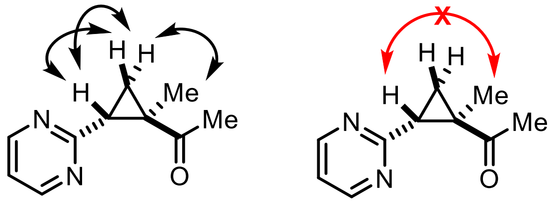
H
H
H
Me
Me
N
N
O

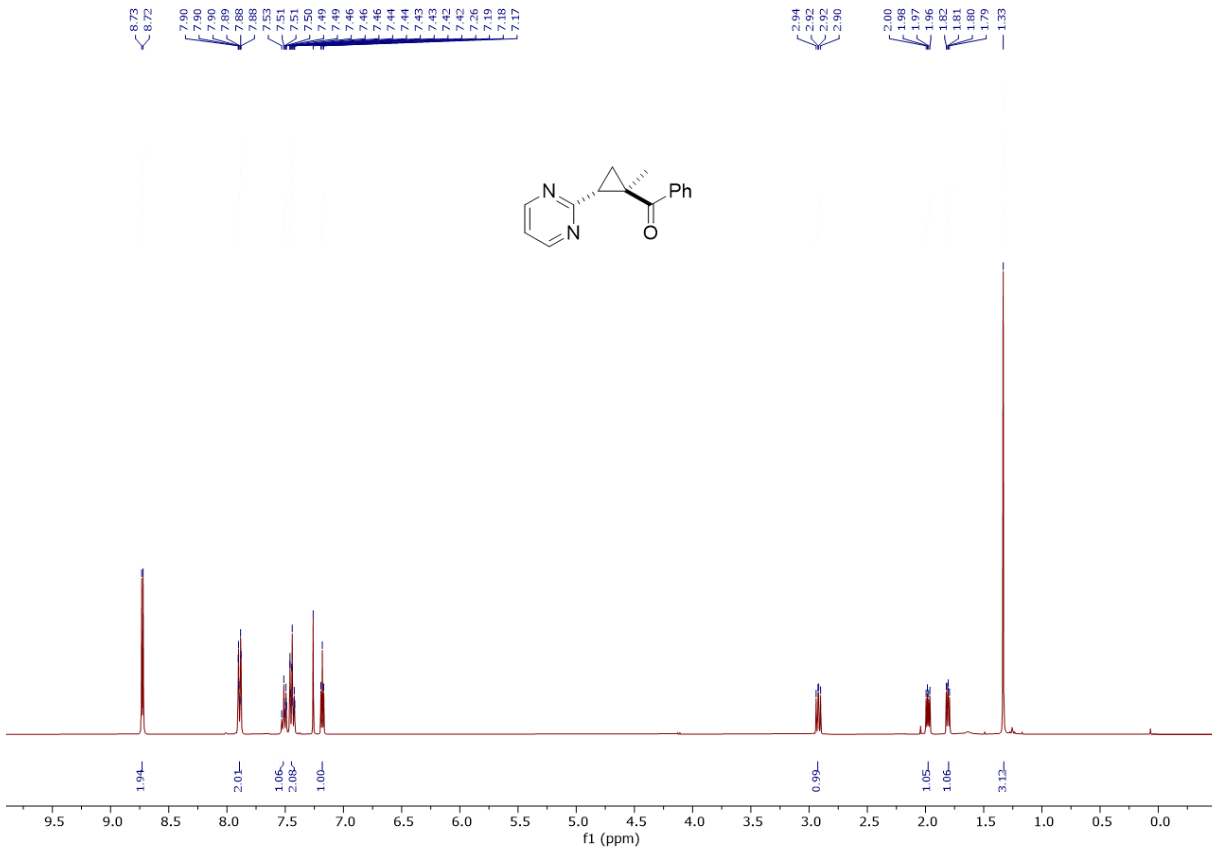
8.73
8.72
7.90
7.90
7.90
7.89
7.88
7.88
7.53
7.51
7.51
7.50
7.49
7.49
7.46
7.46
7.44
7.43
7.43
7.42
7.42
7.26
7.19
7.18
7.17
2.94
2.92
2.92
2.90
2.00
1.98
1.97
1.96
1.82
1.81
1.80
1.79
1.33
N
N
Ph
O
1.94
2.01
1.06
2.08
1.00
0.99
1.05
1.06
3.13
9.5
9.0
8.5
8.0
7.5
7.0
6.5
6.0
5.5
5.0
4.5
4.0
3.5
3.0
2.5
2.0
1.5
1.0
0.5
0.0
f1 (ppm)

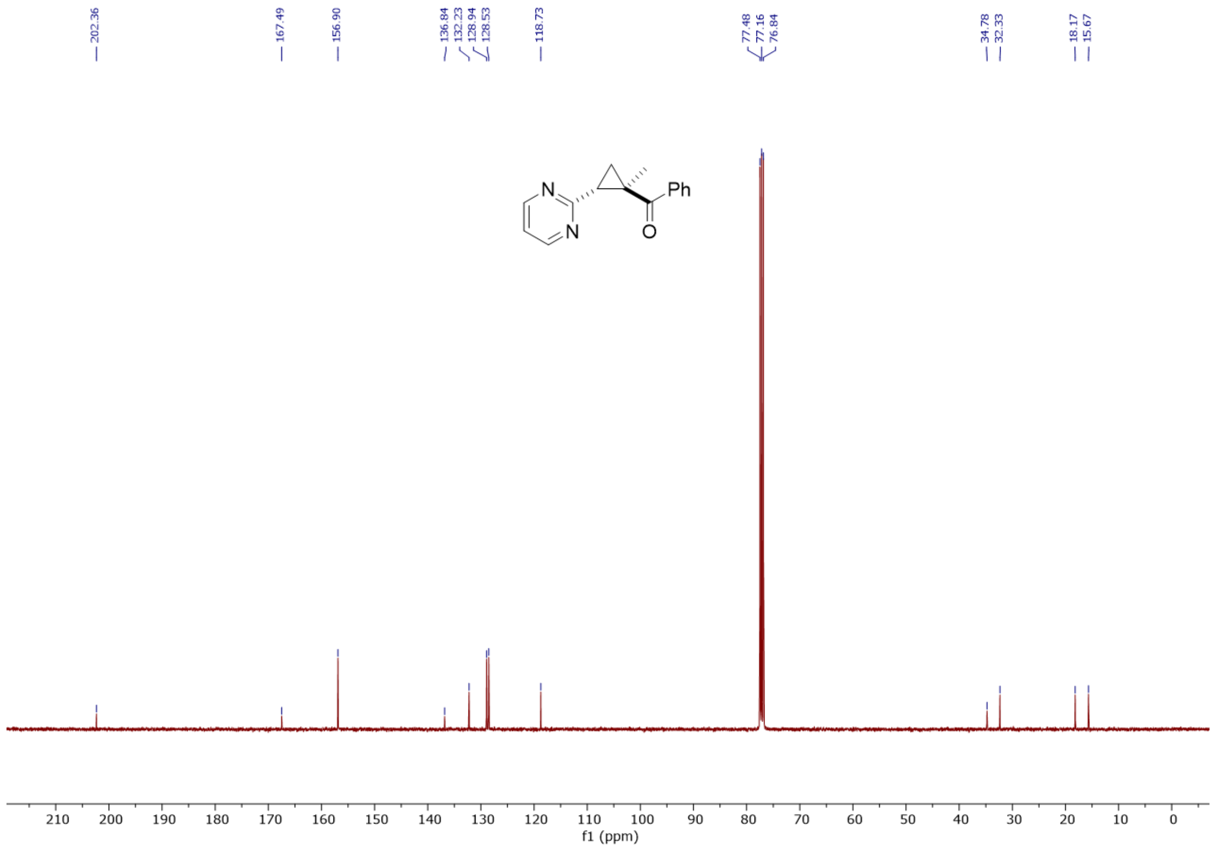
202.36
167.49
156.90
136.84
132.23
128.94
128.53
118.73
77.48
77.16
76.84
34.78
32.33
18.17
15.67
N
N
Ph
O
210
200
190
180
170
160
150
140
130
120
110
100
90
80
70
60
50
40
30
20
10
0
f1 (ppm)

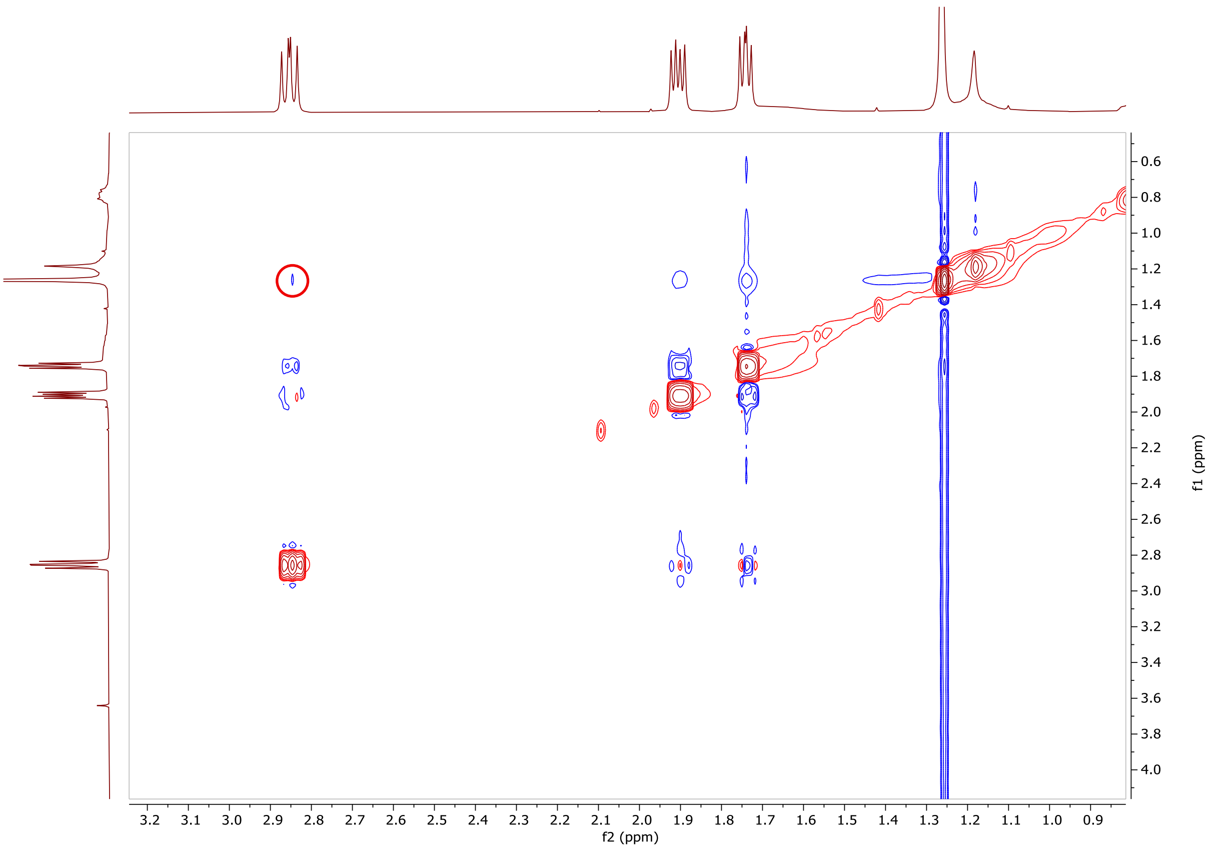
f2 (ppm)
f1 (ppm)

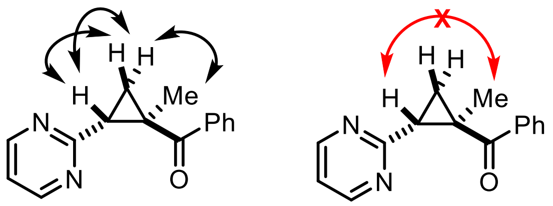
H
H
H
Me
Ph
N
N
O

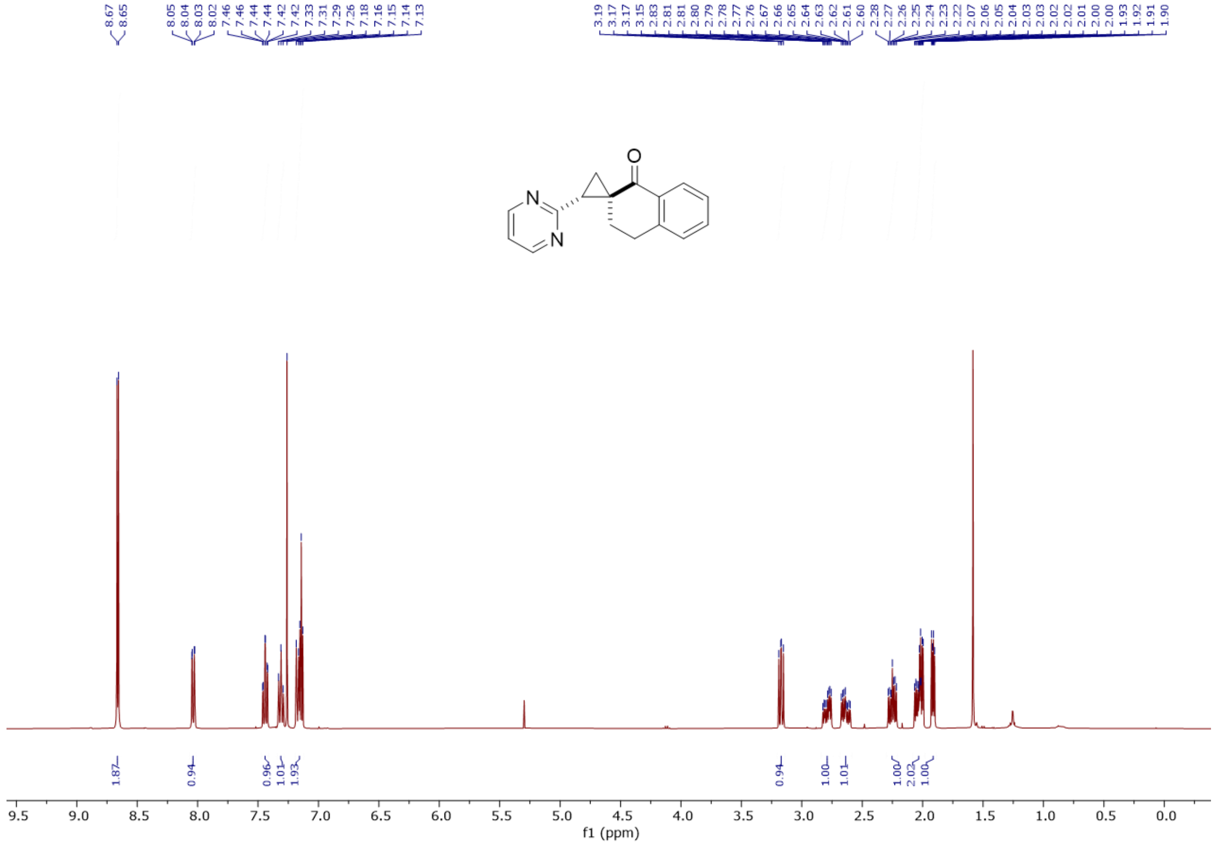
f1 (ppm)

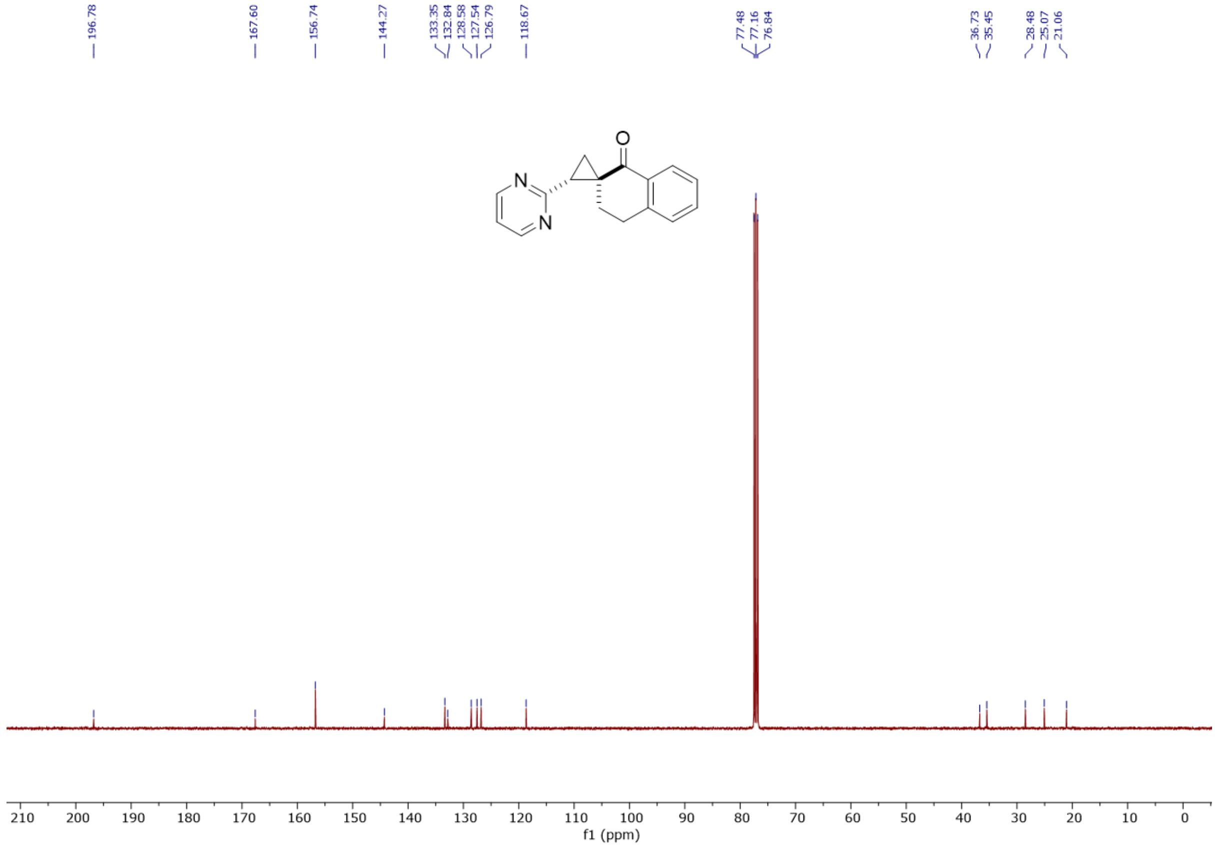
f1 (ppm)

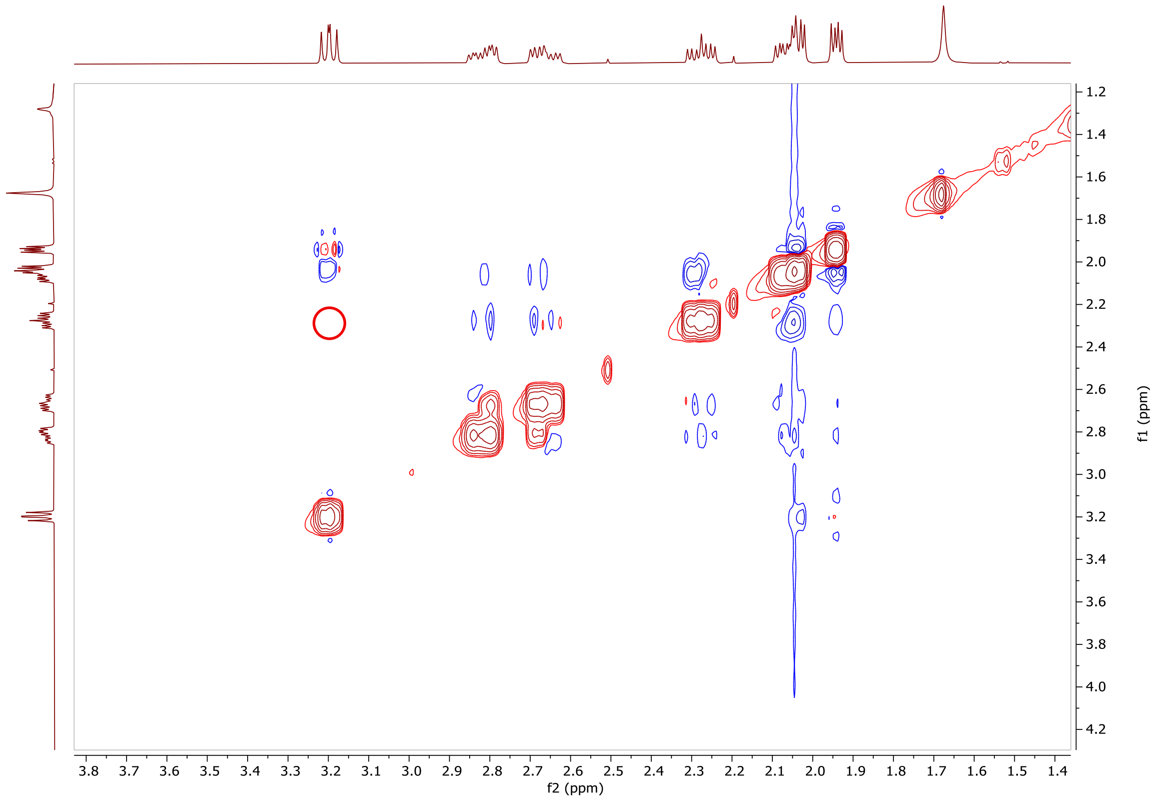
f2 (ppm)
f1 (ppm)

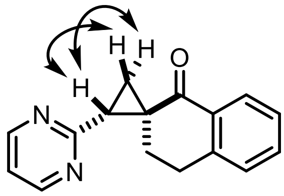

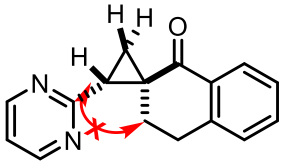

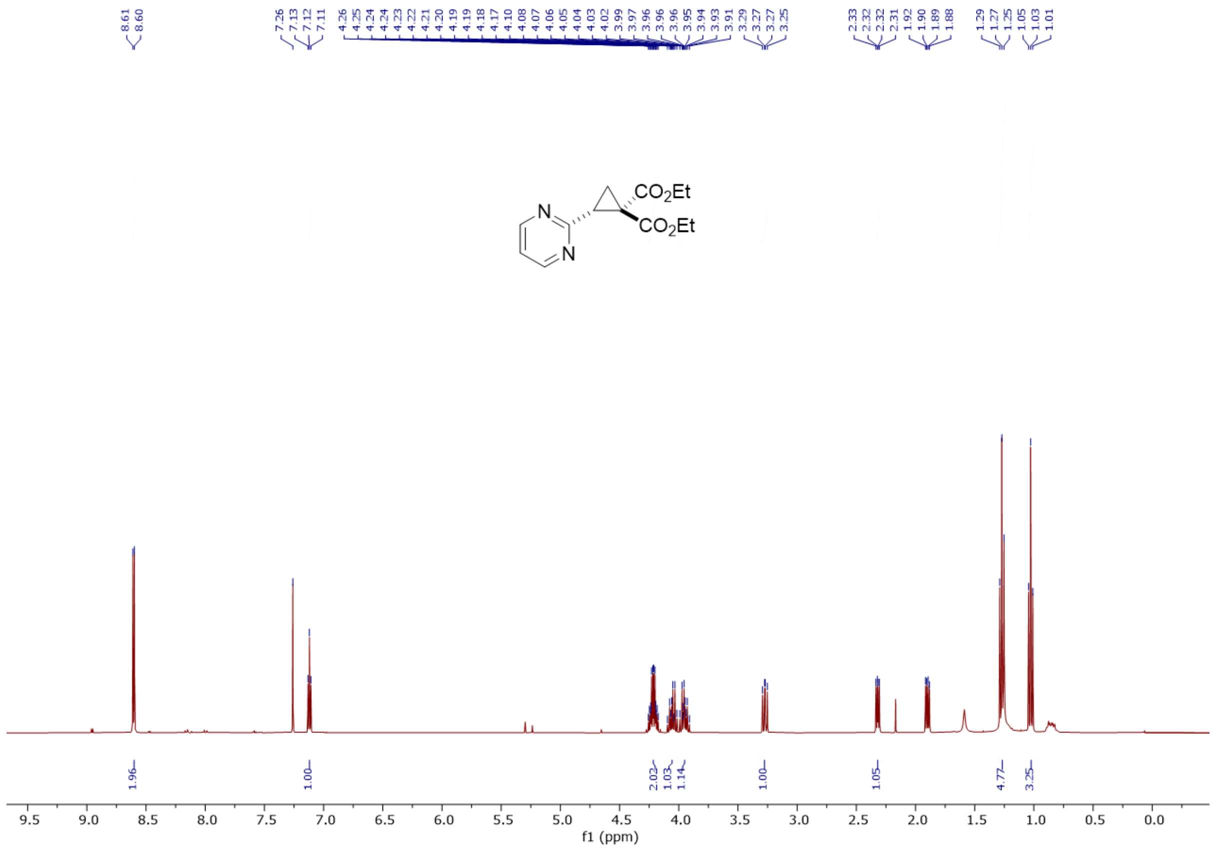
8.61
8.60
7.26
7.13
7.12
7.11
4.26
4.25
4.24
4.24
4.23
4.22
4.21
4.20
4.19
4.19
4.18
4.17
4.10
4.08
4.07
4.06
4.05
4.04
4.03
4.02
3.99
3.97
3.97
3.96
3.96
3.96
3.95
3.94
3.93
3.91
3.29
3.27
3.27
3.25
2.33
2.32
2.32
2.31
1.92
1.90
1.89
1.88
1.29
1.27
1.25
1.05
1.03
1.01
CO2Et
CO2Et
N
N
1.96
1.00
2.02
1.03
1.14
1.00
1.05
4.77
3.25
f1 (ppm)

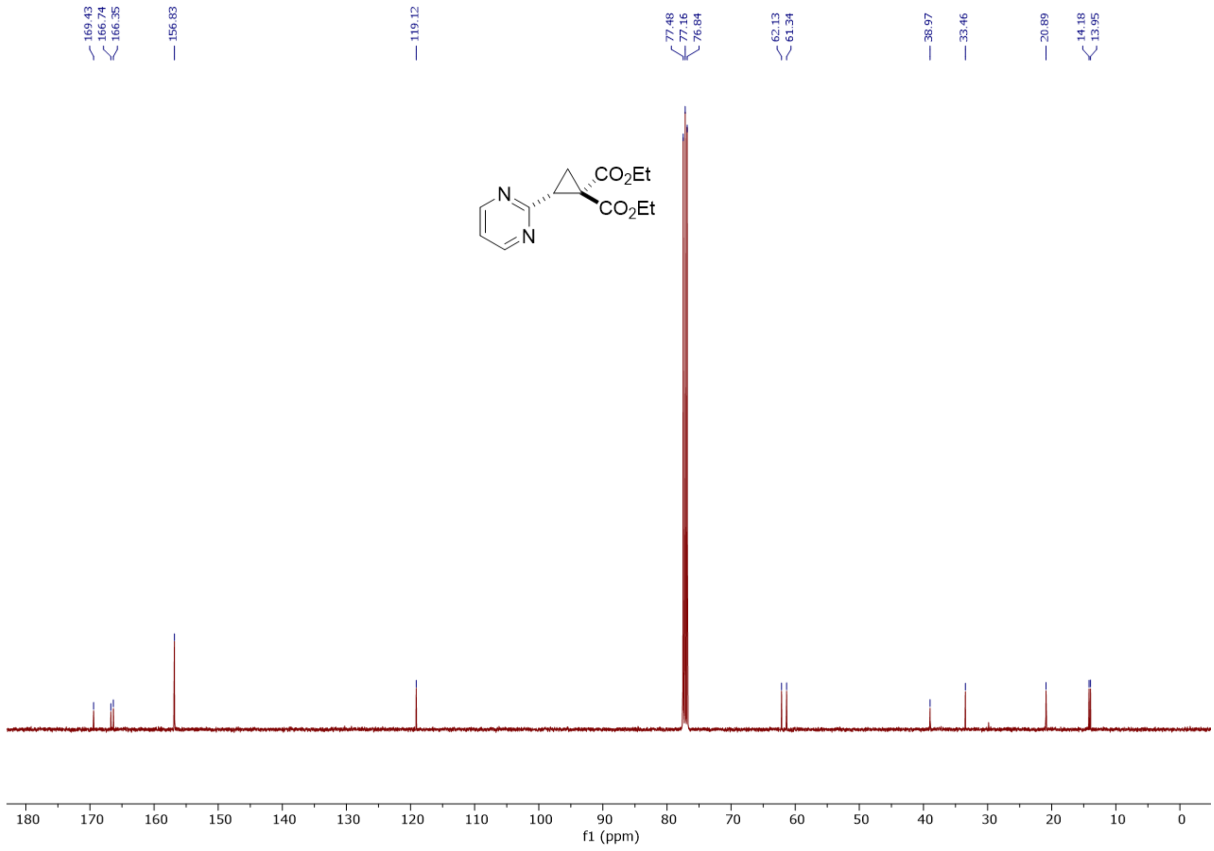
169.43
166.74
166.35
156.83
119.12
77.48
77.16
76.84
62.13
61.34
38.97
33.46
20.89
14.18
13.95
CO2Et
CO2Et
N
N
f1 (ppm)

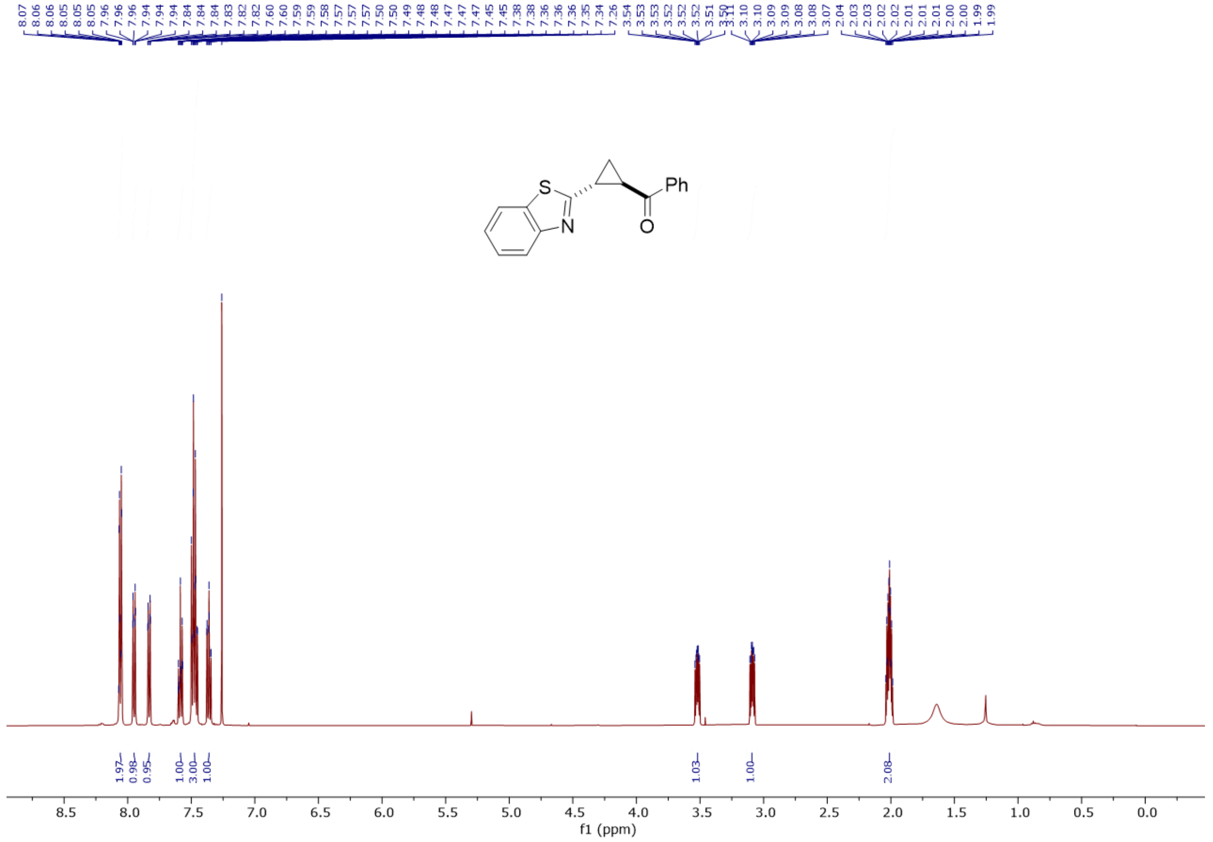

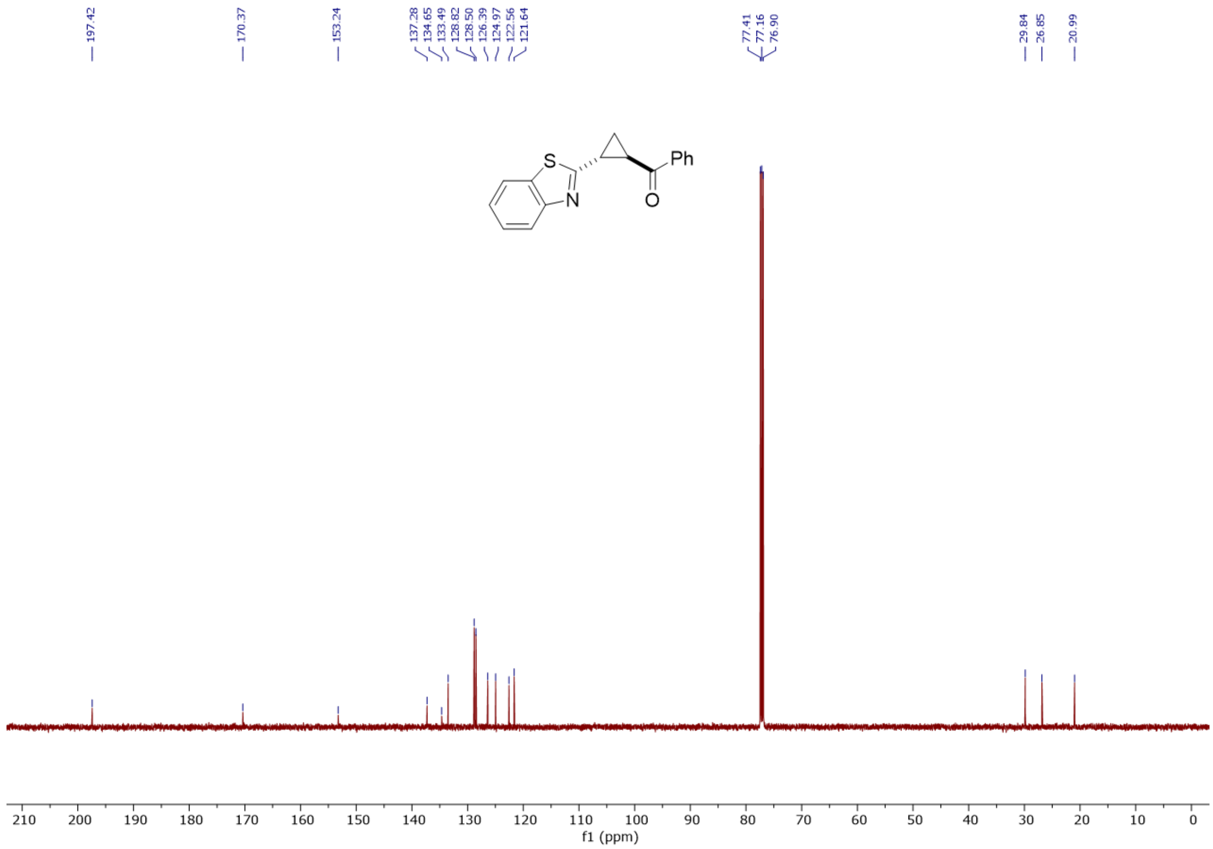

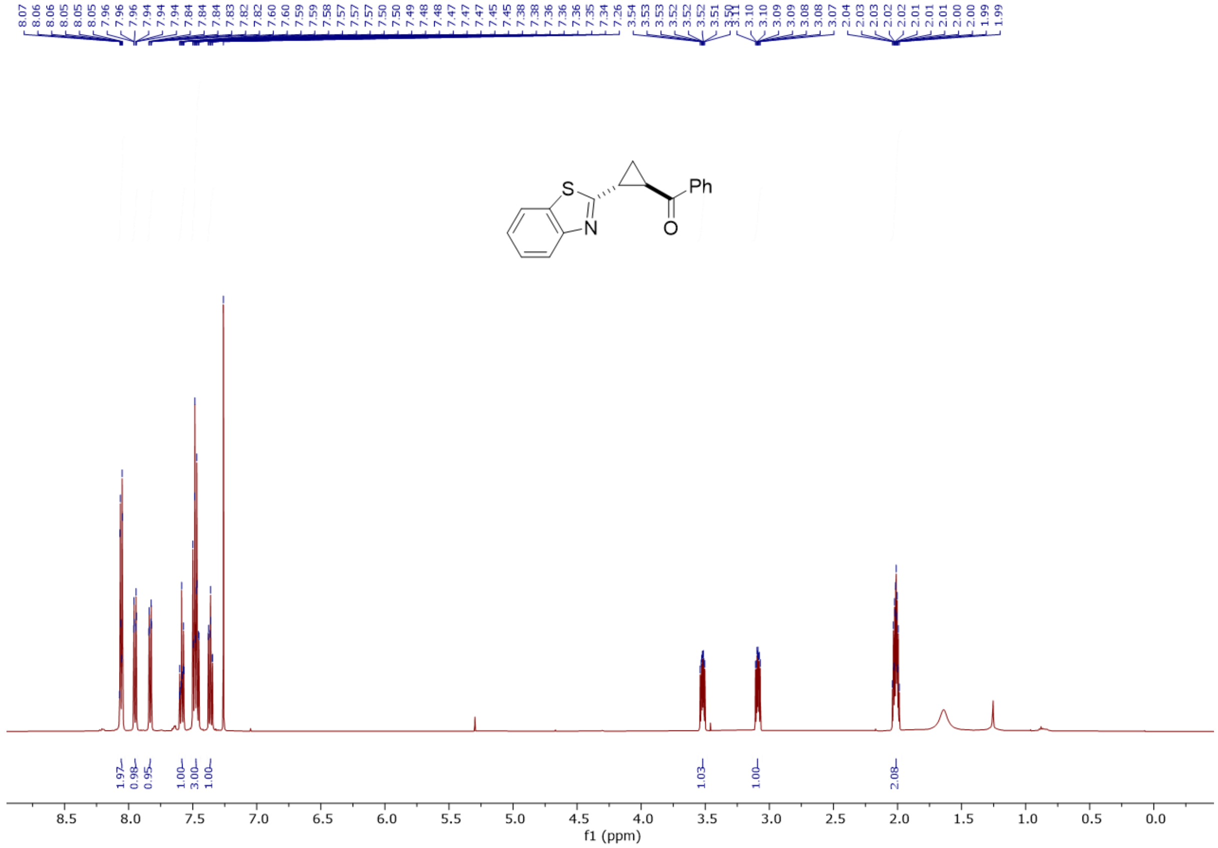
8.07 8.06 8.06 8.05 8.05 8.05 7.96 7.96 7.94 7.94 7.94 7.84 7.84 7.84 7.83 7.82 7.82 7.60 7.60 7.59 7.59 7.58 7.57 7.57 7.57 7.50 7.50 7.49 7.48 7.48 7.47 7.47 7.45 7.45 7.38 7.38 7.36 7.36 7.36 7.35 7.34 7.26 3.54 3.53 3.53 3.52 3.52 3.52 3.51 3.50 3.11 3.10 3.10 3.09 3.09 3.08 3.08 3.07 2.04 2.03 2.03 2.02 2.02 2.01 2.01 2.01 2.00 2.00 1.99 1.99
S
N
Ph
O
1.97 0.98 0.95 1.00 3.00 1.00 1.03 1.00 2.08
f1 (ppm)

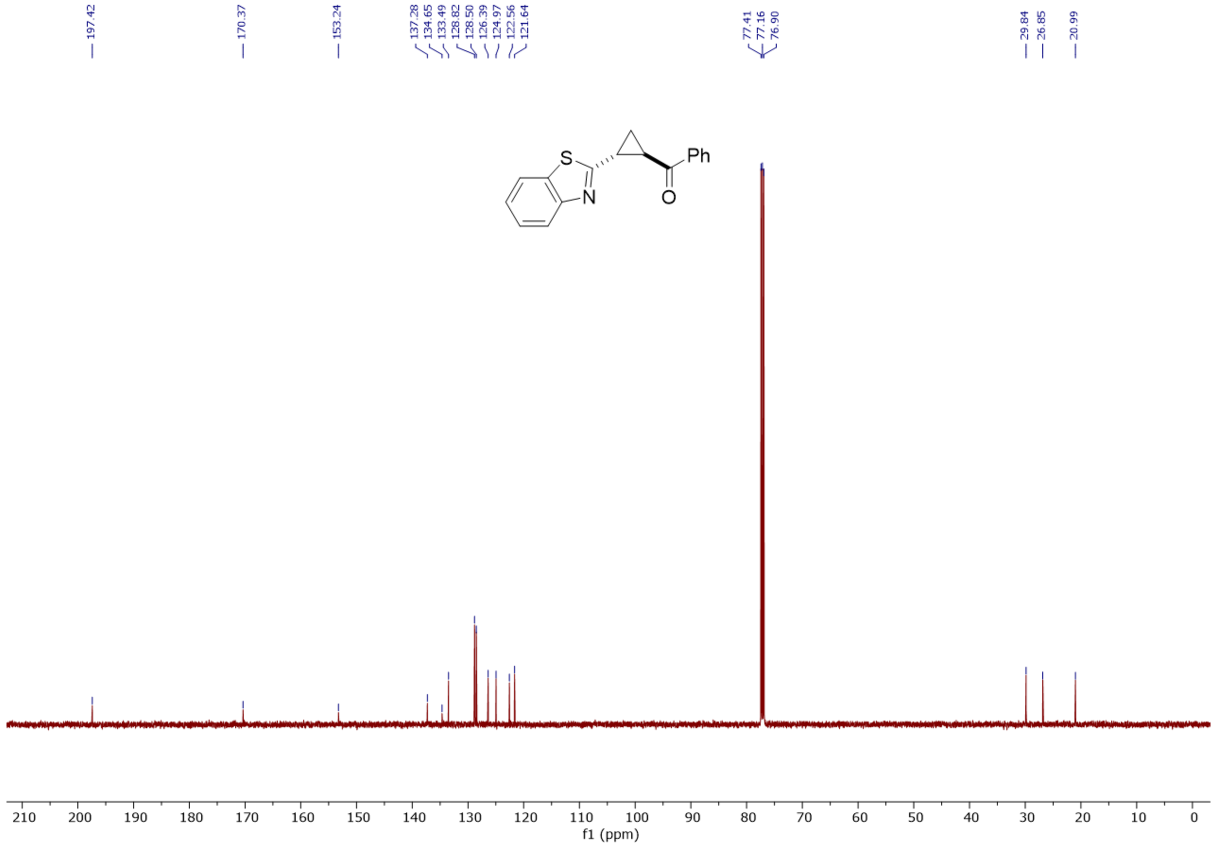
197.42
170.37
153.24
137.28 134.65 133.49 128.82 128.50 126.39 124.97 122.56 121.64
77.41 77.16 76.90
29.84 26.85 20.99
S
N
Ph
O
f1 (ppm)

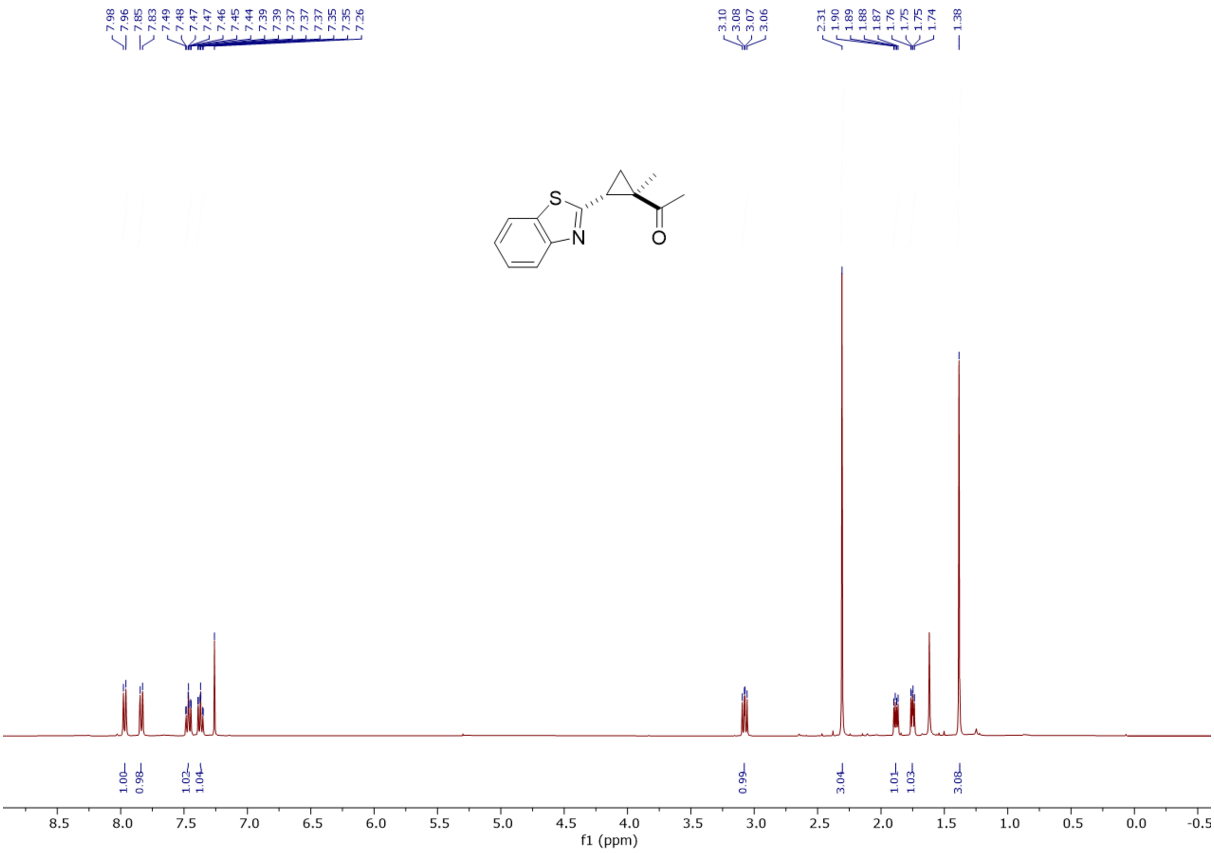
7.98
7.96
7.85
7.83
7.49
7.48
7.47
7.47
7.46
7.45
7.44
7.39
7.39
7.37
7.37
7.37
7.35
7.35
7.26
3.10
3.08
3.07
3.06
2.31
1.90
1.89
1.88
1.87
1.76
1.75
1.75
1.74
1.38
S
N
O
1.00
0.98
1.03
1.04
0.99
3.04
1.01
1.03
3.08
f1 (ppm)

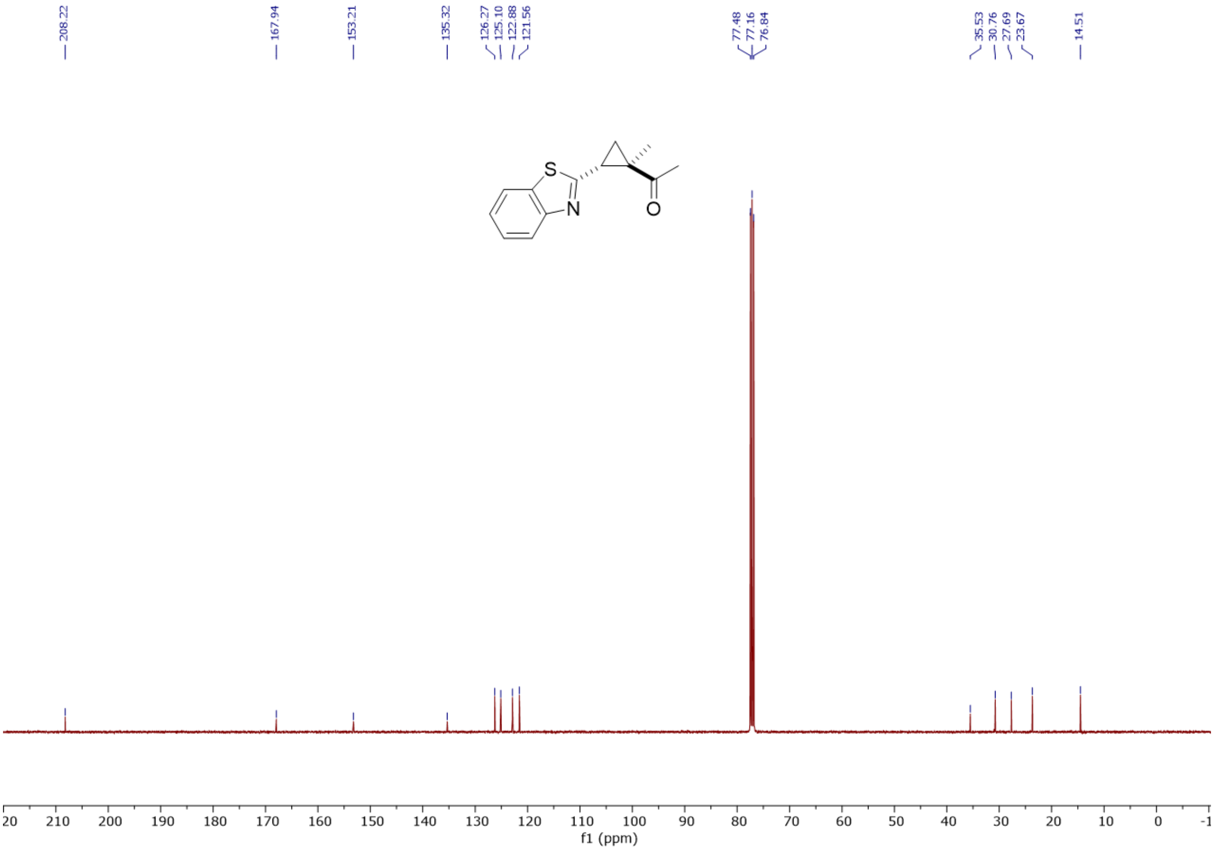
208.22
167.94
153.21
135.32
126.27
125.10
122.88
121.56
77.48
77.16
76.84
35.53
30.76
27.69
23.67
14.51
S
N
O
f1 (ppm)

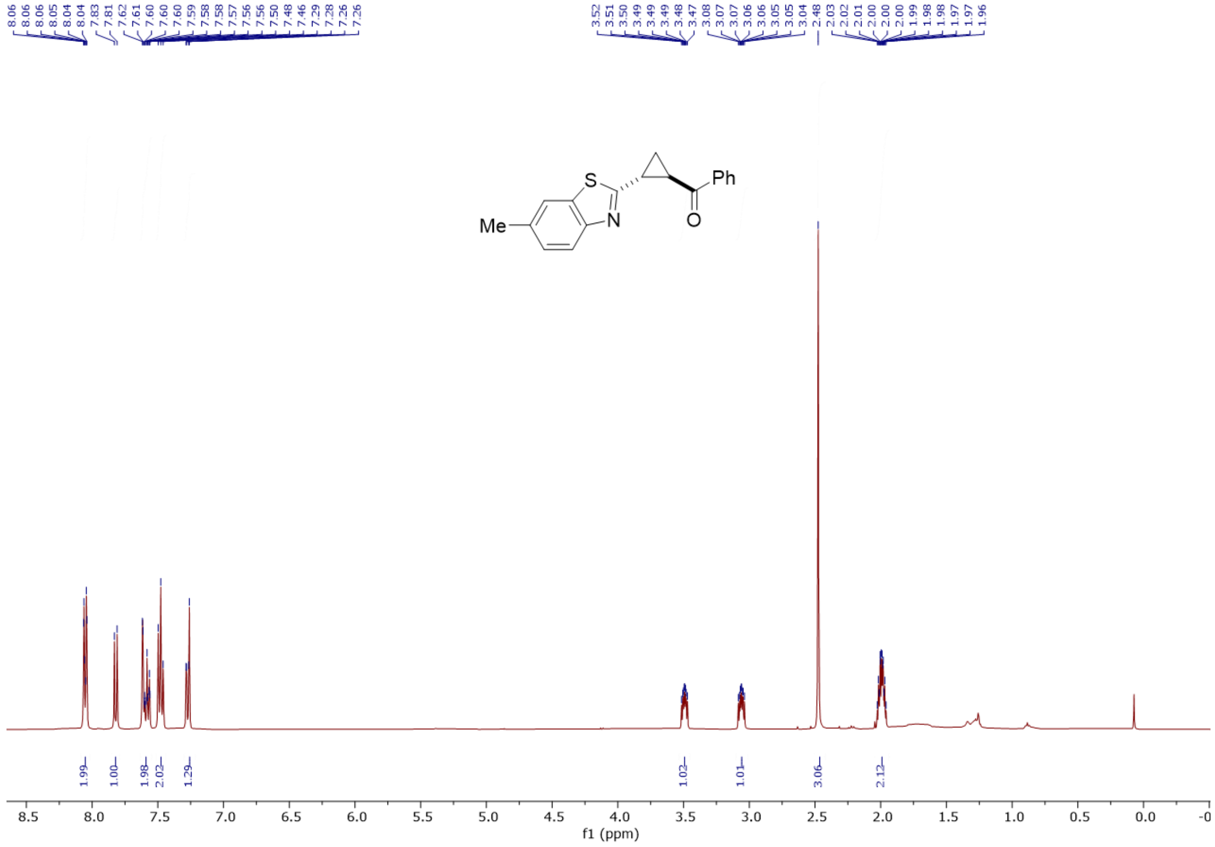
S
N
Me
Ph
O
f1 (ppm)

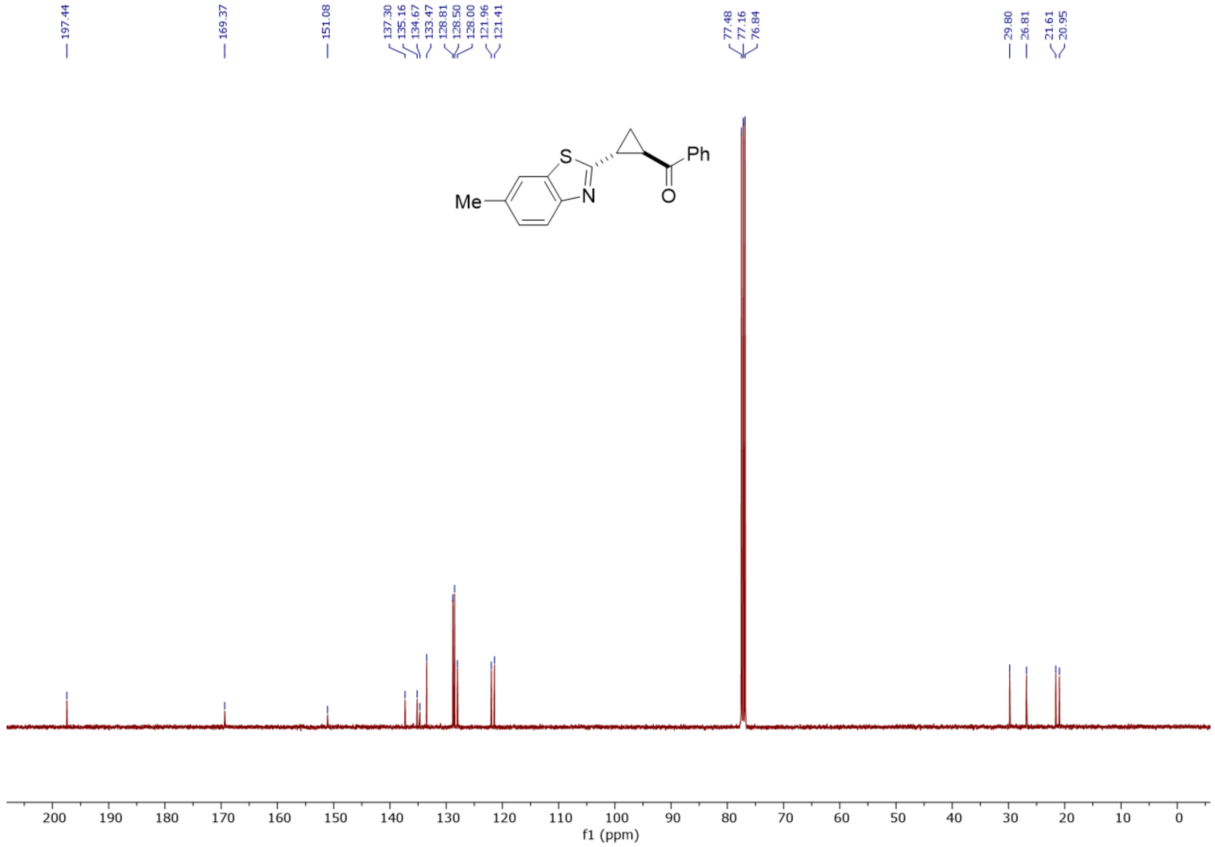
197.44
169.37
151.08
137.30
135.16
134.67
133.47
128.81
128.50
128.00
121.96
121.41
77.48
77.16
76.84
29.80
26.81
21.61
20.95
S
N
Me
Ph
O
f1 (ppm)

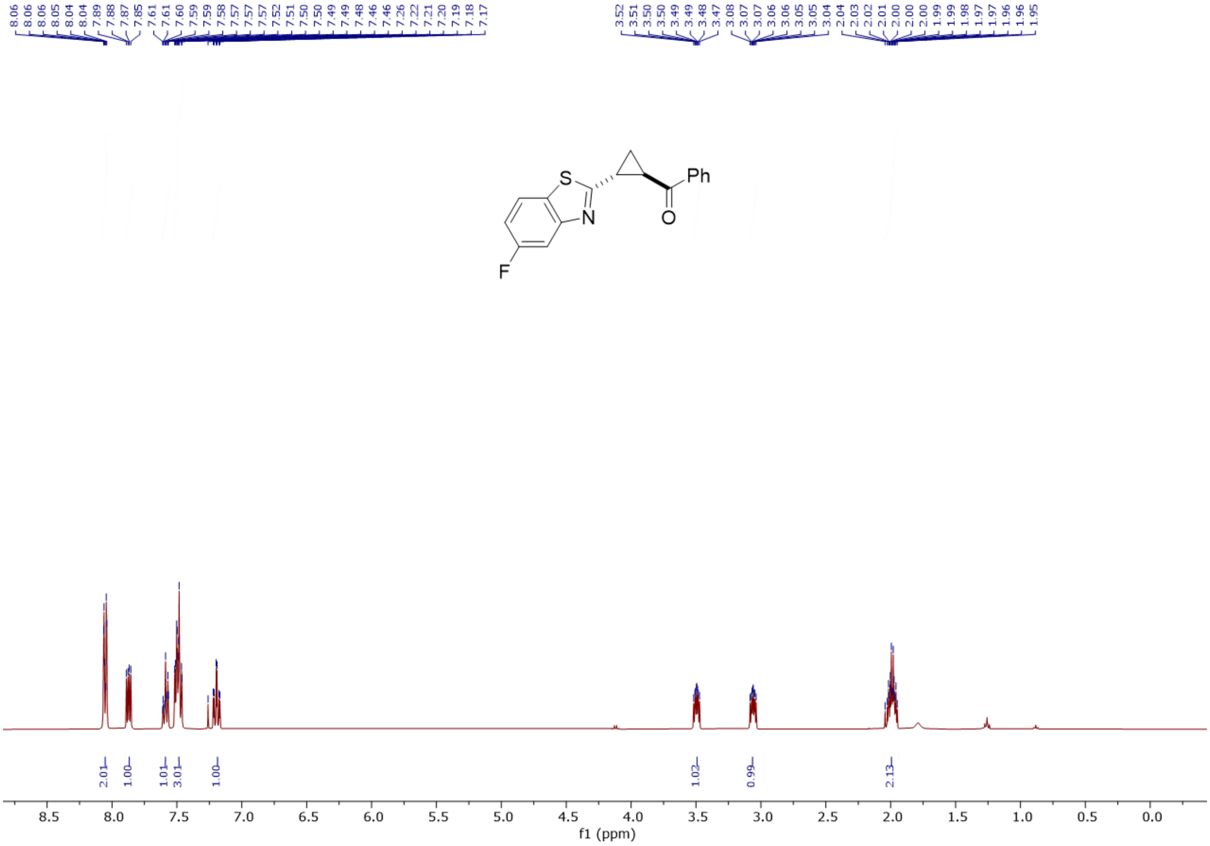
S
N
F
Ph
O
f1 (ppm)

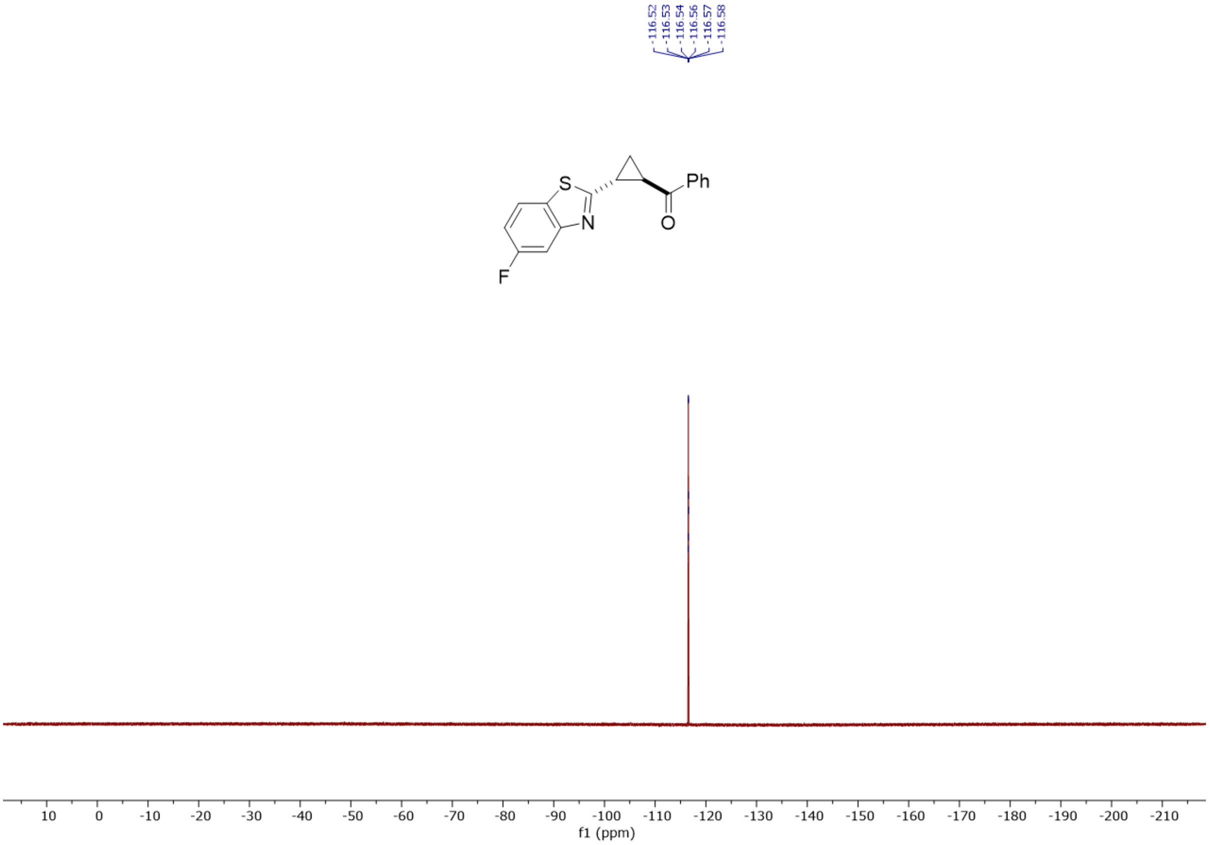
S
N
F
Ph
O
f1 (ppm)

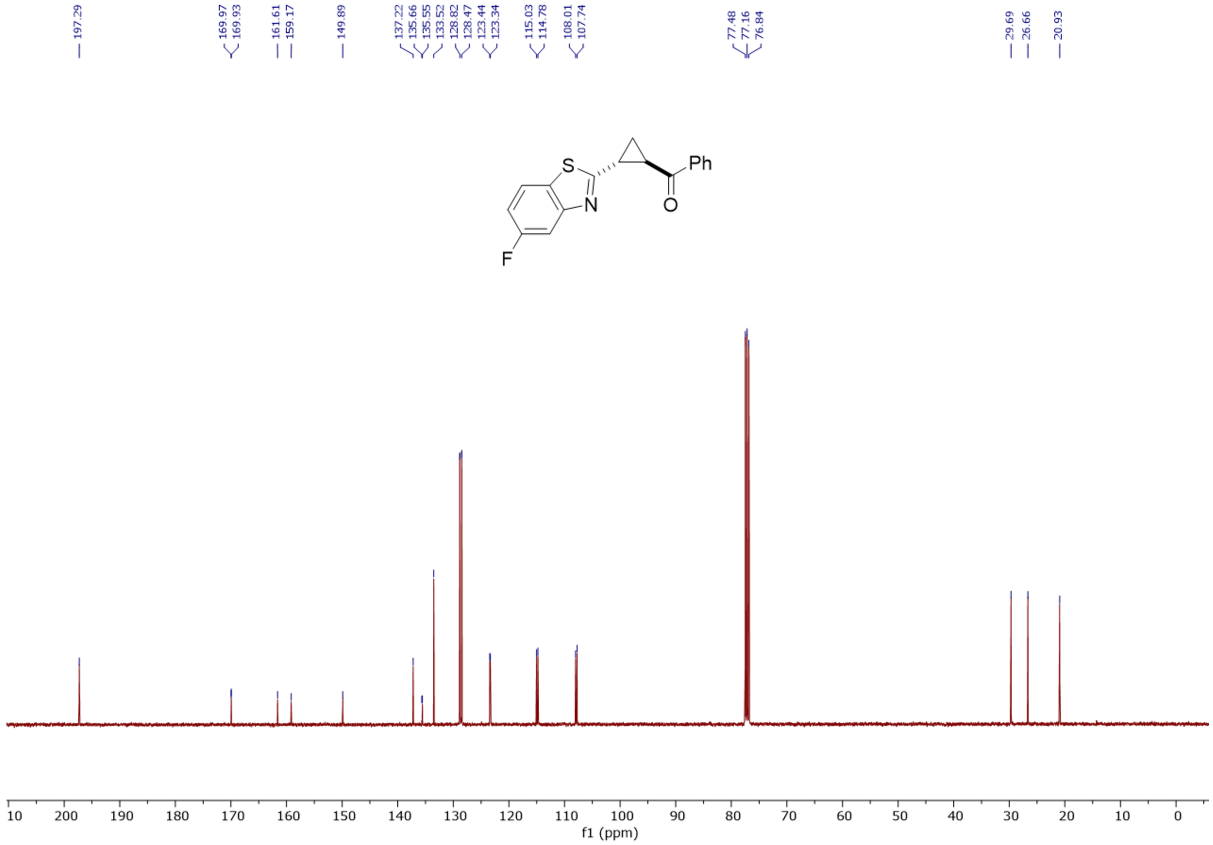
197.29
169.97
169.93
161.61
159.17
149.89
137.22
135.66
135.55
133.52
128.82
128.47
123.44
123.34
115.03
114.78
108.01
107.74
77.48
77.16
76.84
29.69
26.66
20.93
S
N
Ph
O
F
f1 (ppm)

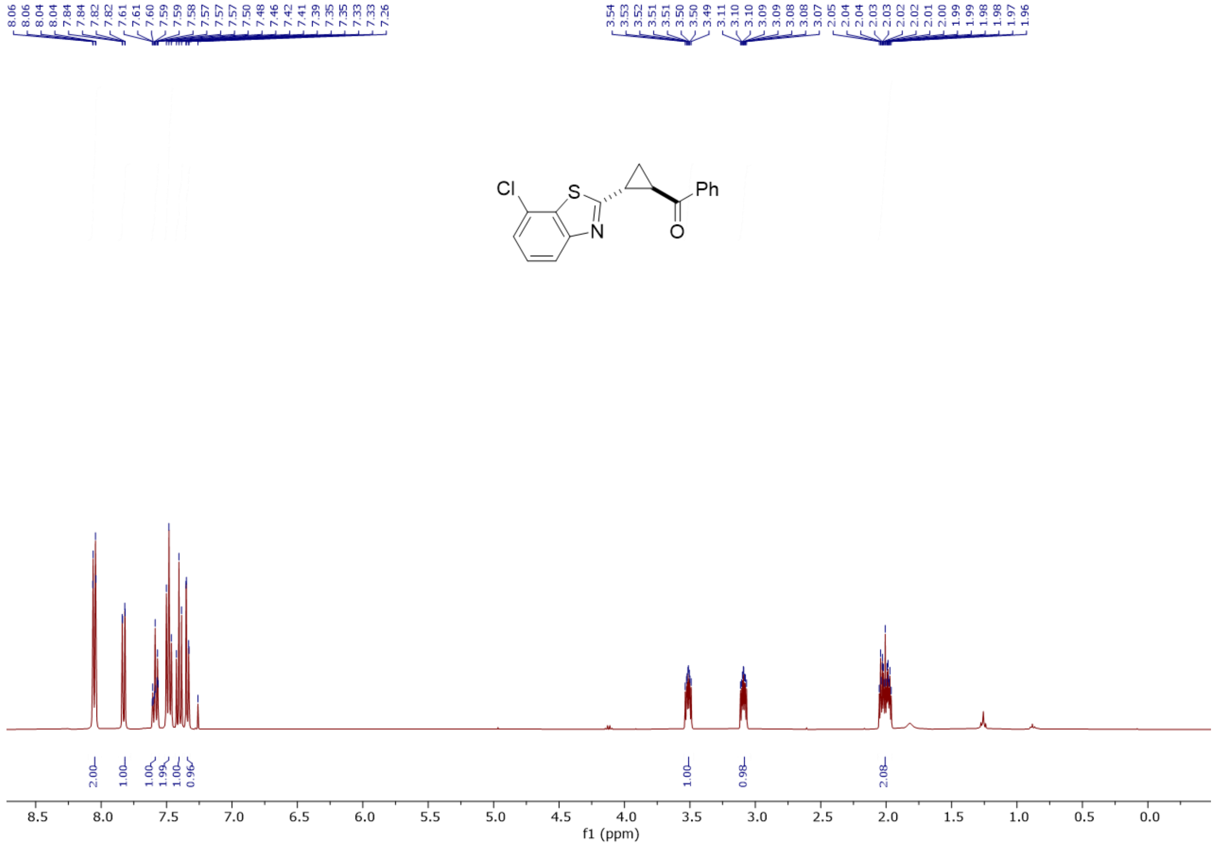
8.06
8.06
8.04
7.84
7.82
7.82
7.61
7.61
7.60
7.59
7.59
7.58
7.57
7.57
7.50
7.48
7.46
7.42
7.41
7.39
7.35
7.35
7.33
7.33
7.26
3.54
3.53
3.52
3.51
3.51
3.50
3.50
3.49
3.11
3.10
3.10
3.09
3.09
3.08
3.08
3.07
2.05
2.04
2.04
2.03
2.03
2.02
2.02
2.01
2.00
1.99
1.99
1.98
1.98
1.97
1.96
Cl
S
N
Ph
O
2.00
1.00
1.00
1.99
1.00
0.96
1.00
0.98
2.08
f1 (ppm)

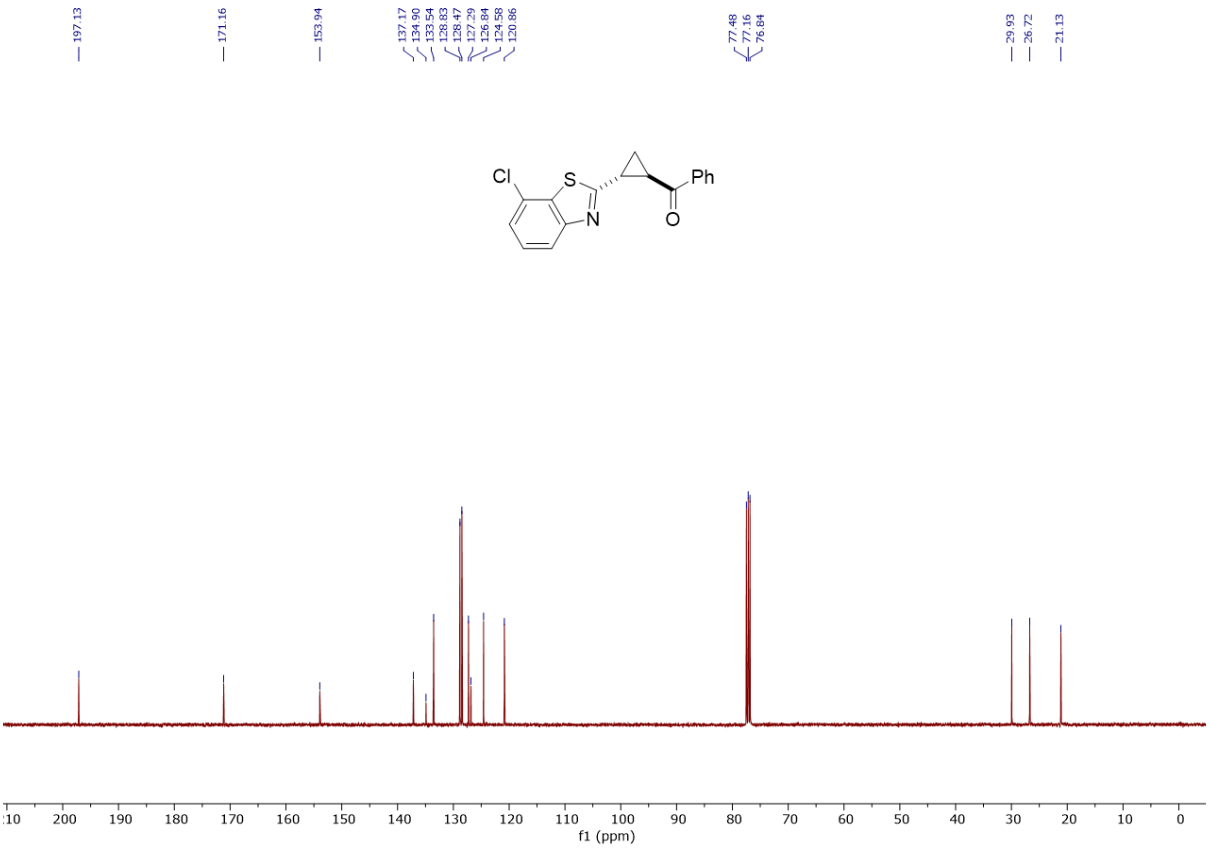
197.13
171.16
153.94
137.17 134.90 133.54 128.83 128.47 127.29 126.84 124.58 120.86
77.48 77.16 76.84
29.93 26.72 21.13
Cl
S
N
Ph
O
f1 (ppm)

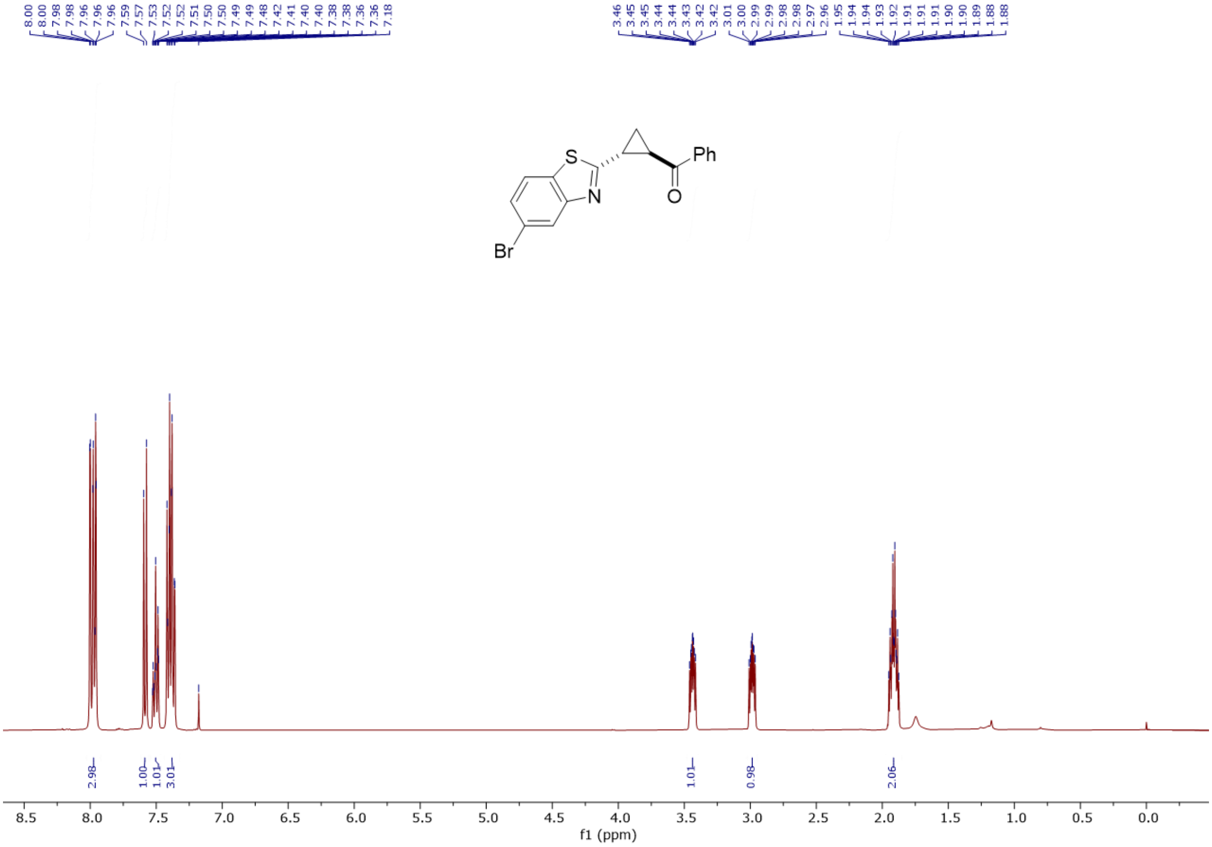
8.00 8.00 7.98 7.98 7.96 7.96 7.96 7.59 7.57 7.53 7.52 7.52 7.51 7.50 7.50 7.49 7.49 7.48 7.42 7.41 7.40 7.40 7.38 7.38 7.36 7.18
3.46 3.45 3.45 3.44 3.44 3.43 3.42 3.42 3.01 3.00 2.99 2.99 2.98 2.98 2.97 2.96
1.95 1.94 1.94 1.93 1.92 1.91 1.91 1.91 1.90 1.90 1.89 1.88 1.88
S
N
Br
Ph
O
2.98
1.00
1.01
3.01
1.01
0.98
2.06
f1 (ppm)

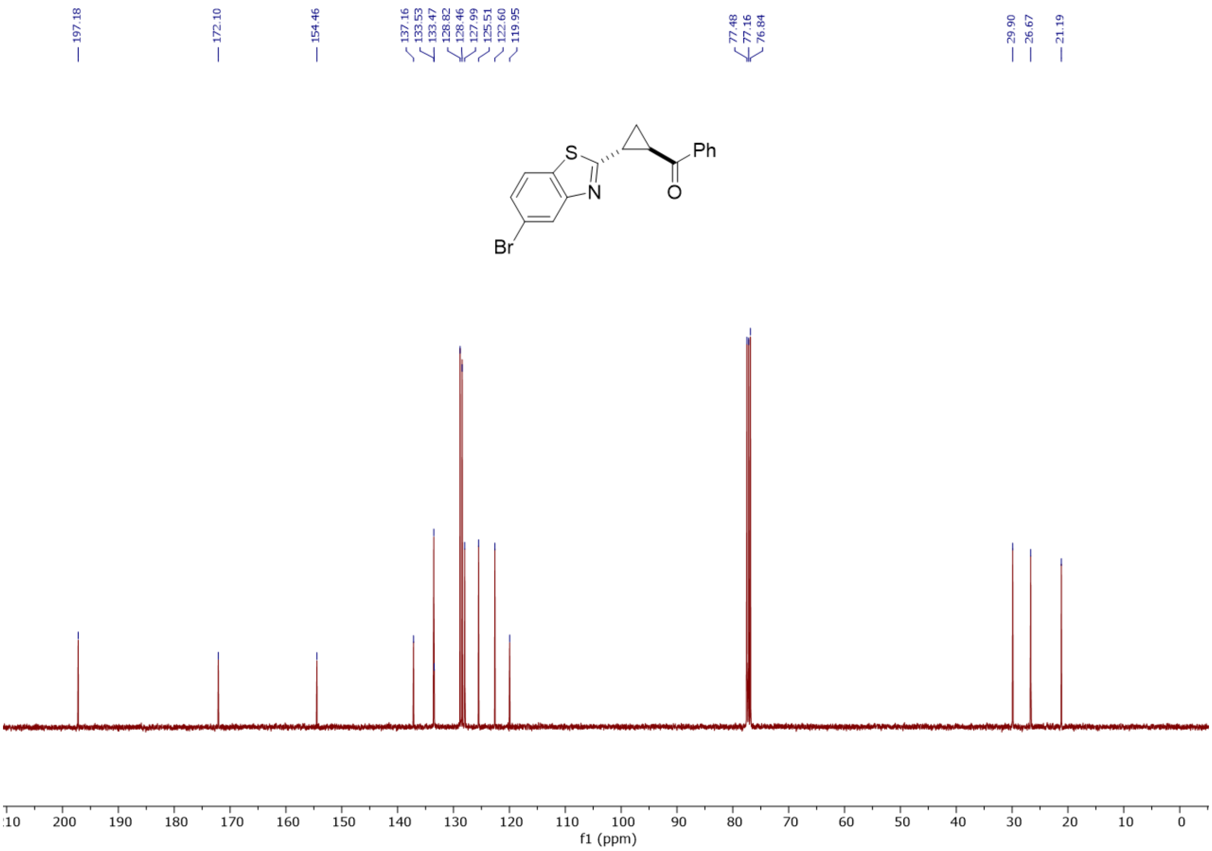
197.18
172.10
154.46
137.16
133.53
133.47
128.82
128.46
127.99
125.51
122.60
119.95
77.48
77.16
76.84
29.90
26.67
21.19
S
N
Ph
O
Br
f1 (ppm)

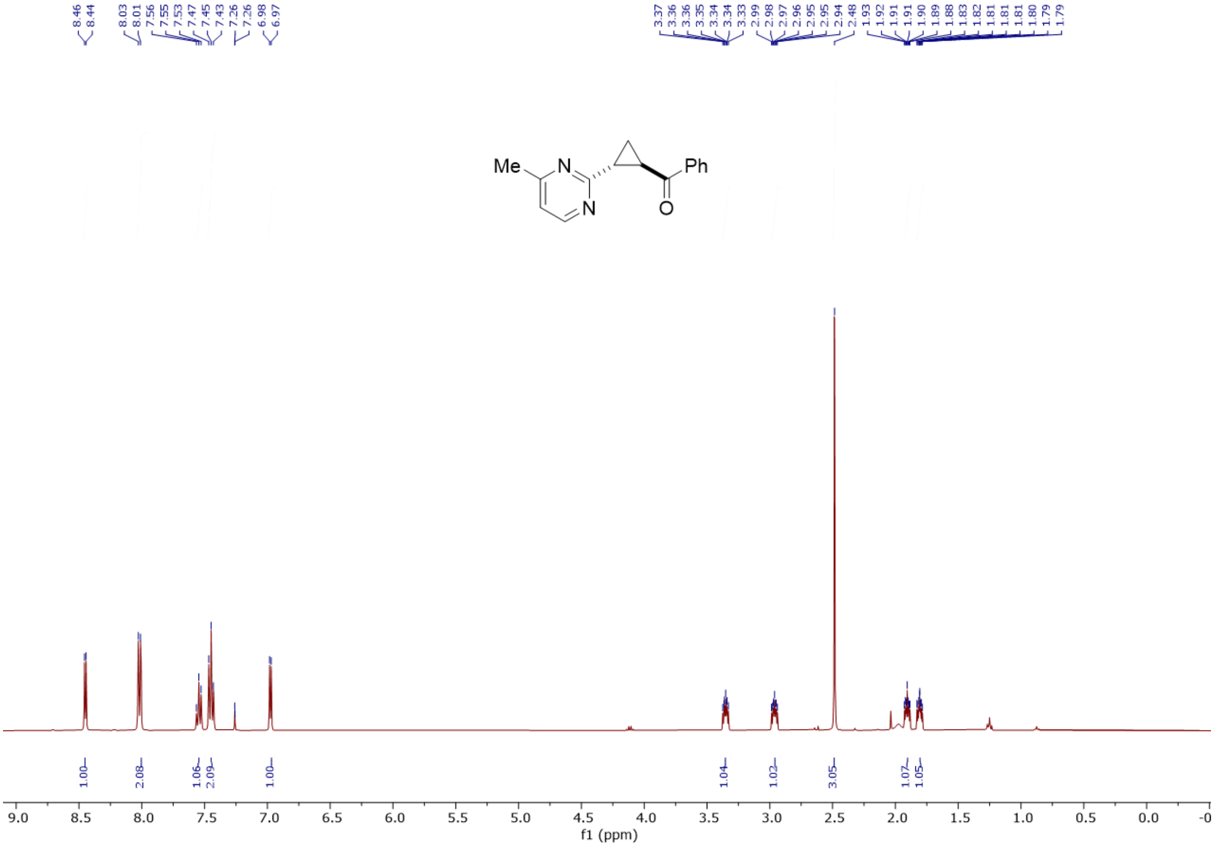
8.46
8.44
8.03
8.01
7.56
7.55
7.53
7.47
7.45
7.43
7.26
7.26
6.98
6.97
3.37
3.36
3.36
3.35
3.34
3.34
3.33
2.99
2.98
2.97
2.96
2.95
2.95
2.94
2.48
1.93
1.92
1.91
1.91
1.90
1.89
1.88
1.83
1.82
1.81
1.81
1.81
1.80
1.79
1.79
Me
N
N
Ph
O
1.00
2.08
1.06
2.09
1.00
1.04
1.02
3.05
1.07
1.05
f1 (ppm)

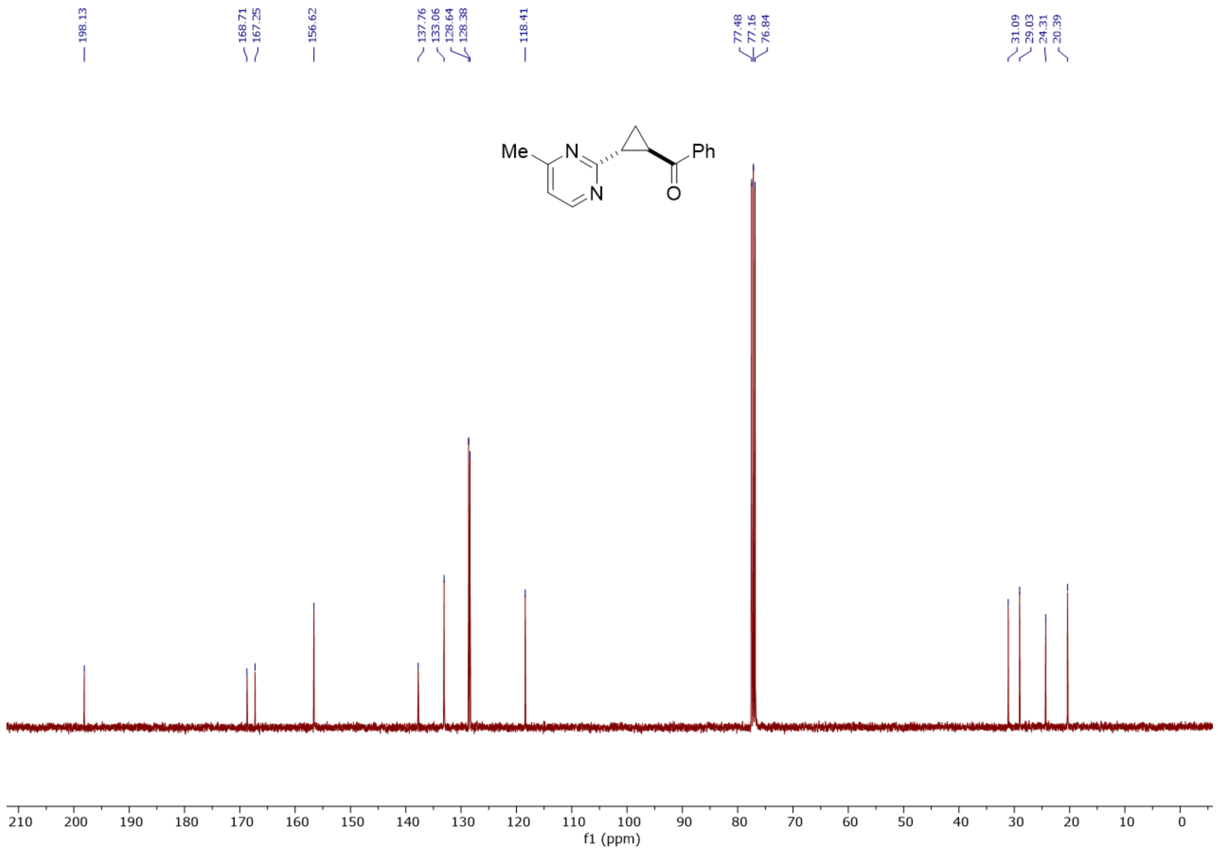
198.13
168.71
167.25
156.62
137.76
133.06
128.64
128.38
118.41
77.48
77.16
76.84
31.09
29.03
24.31
20.39
Me
N
N
Ph
O
f1 (ppm)

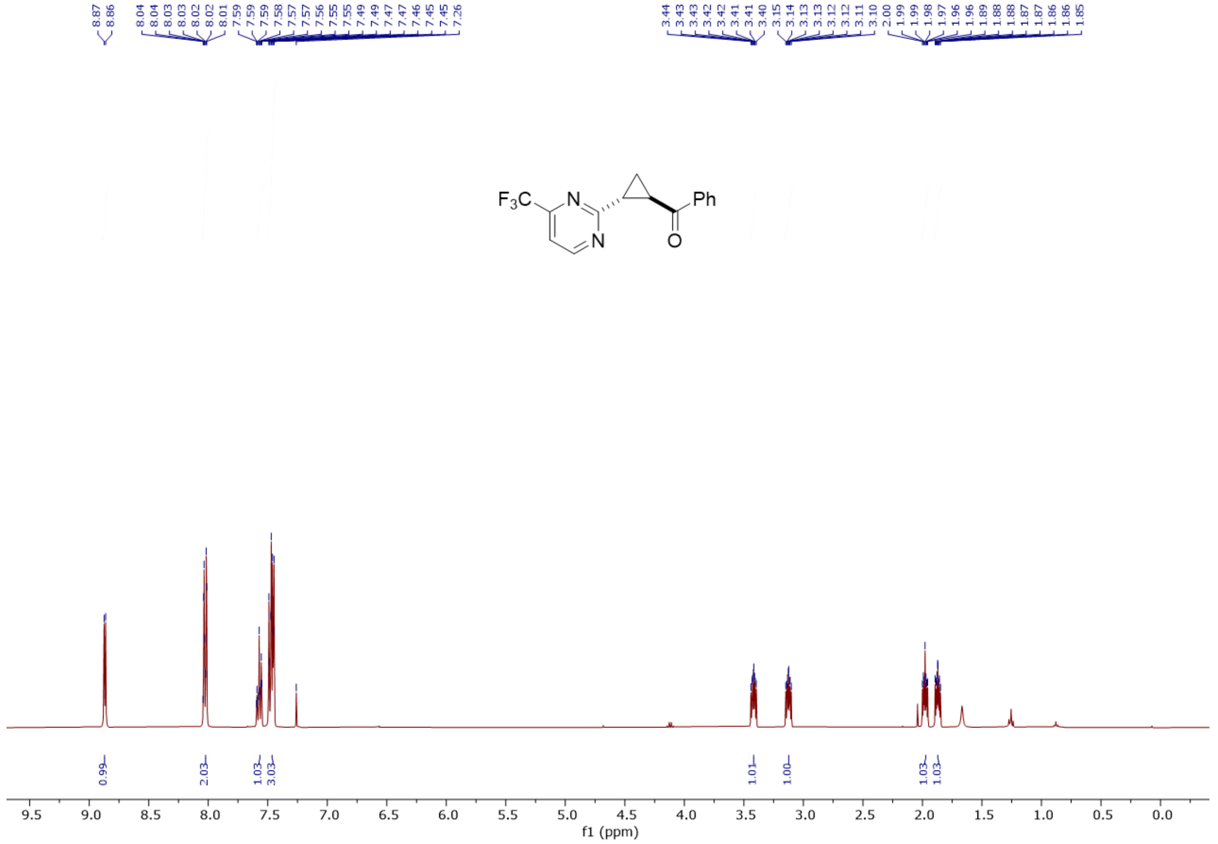
8.87
8.86
8.04
8.04
8.03
8.03
8.02
8.02
8.01
7.59
7.59
7.59
7.58
7.57
7.57
7.56
7.55
7.55
7.49
7.49
7.47
7.47
7.46
7.45
7.45
7.26
3.44
3.43
3.43
3.42
3.42
3.41
3.41
3.40
3.15
3.14
3.13
3.13
3.12
3.12
3.11
3.10
2.00
1.99
1.99
1.98
1.97
1.96
1.96
1.89
1.88
1.88
1.87
1.87
1.86
1.86
1.85
F_3C
N
N
Ph
O
0.99
2.03
1.03
3.03
1.01
1.00
1.03
1.03
f1 (ppm)

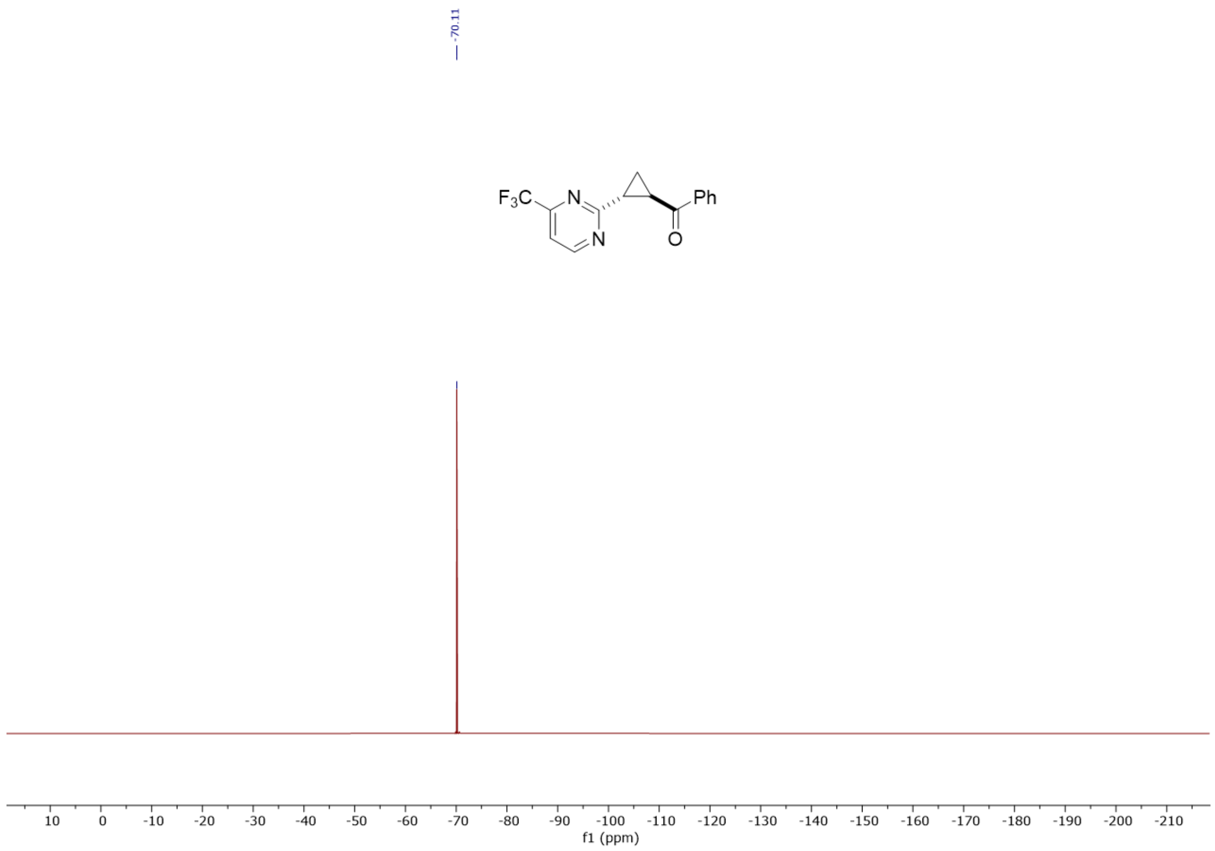
-70.11
F3C
N
N
Ph
O
f1 (ppm)

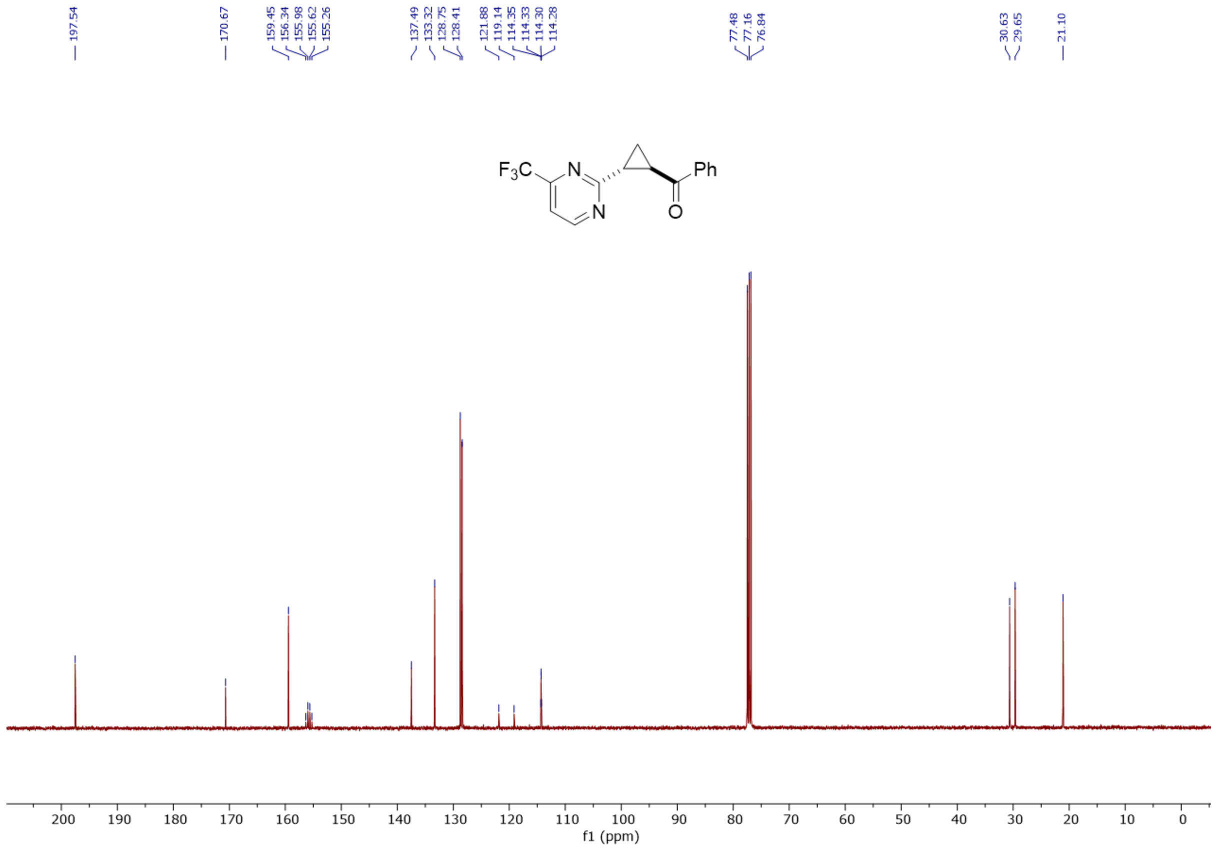
197.54
170.67
159.45
156.34
155.98
155.62
155.26
137.49
133.32
128.75
128.41
121.88
119.14
114.35
114.33
114.30
114.28
77.48
77.16
76.84
30.63
29.65
21.10
F3C
N
N
Ph
O
f1 (ppm)

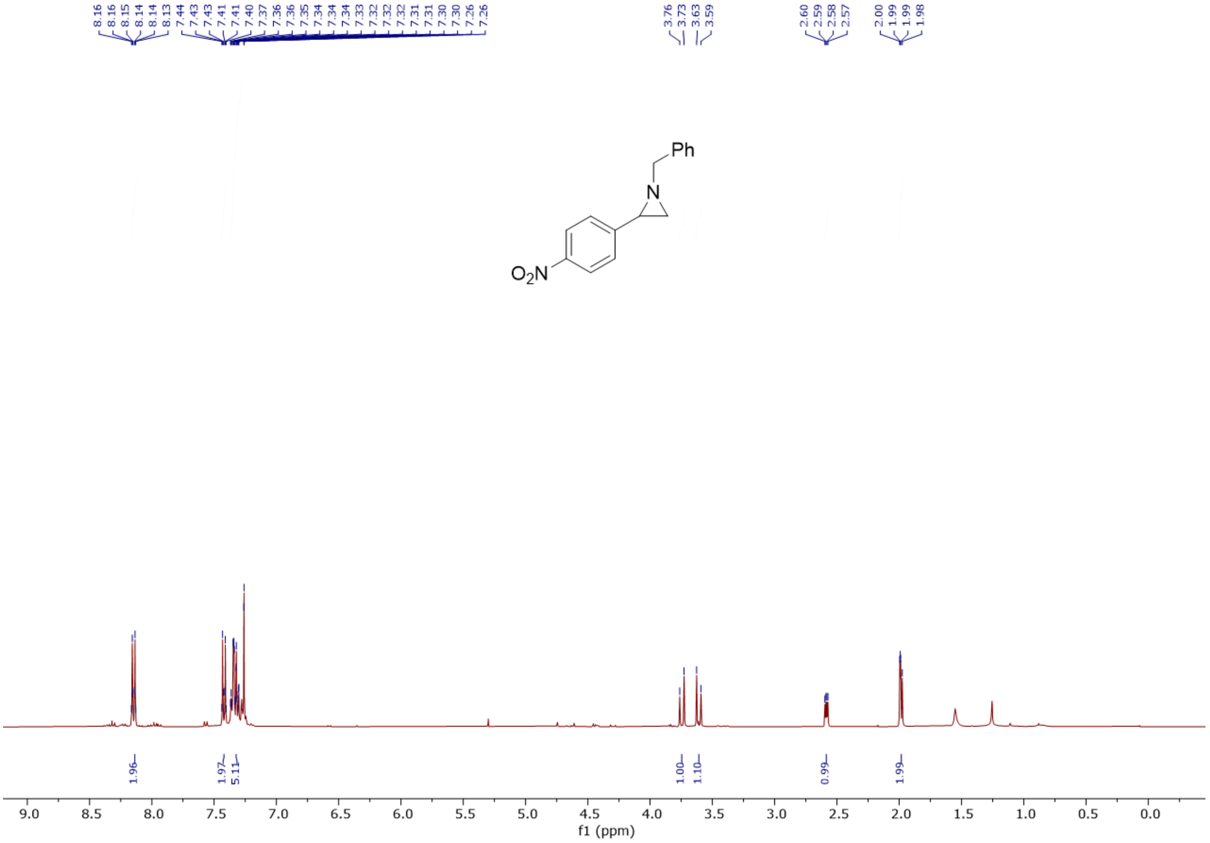
8.16 8.16 8.15 8.14 8.14 8.13 7.44 7.43 7.43 7.41 7.41 7.40 7.37 7.36 7.36 7.35 7.34 7.34 7.34 7.33 7.32 7.32 7.32 7.31 7.31 7.30 7.30 7.26 7.26
3.76 3.73 3.63 3.59
2.60 2.59 2.58 2.57
2.00 1.99 1.99 1.98
Ph
N
O_2N
1.96
1.97
5.11
1.00
1.10
0.99
1.99
9.0 8.5 8.0 7.5 7.0 6.5 6.0 5.5 5.0 4.5 4.0 3.5 3.0 2.5 2.0 1.5 1.0 0.5 0.0
f1 (ppm)

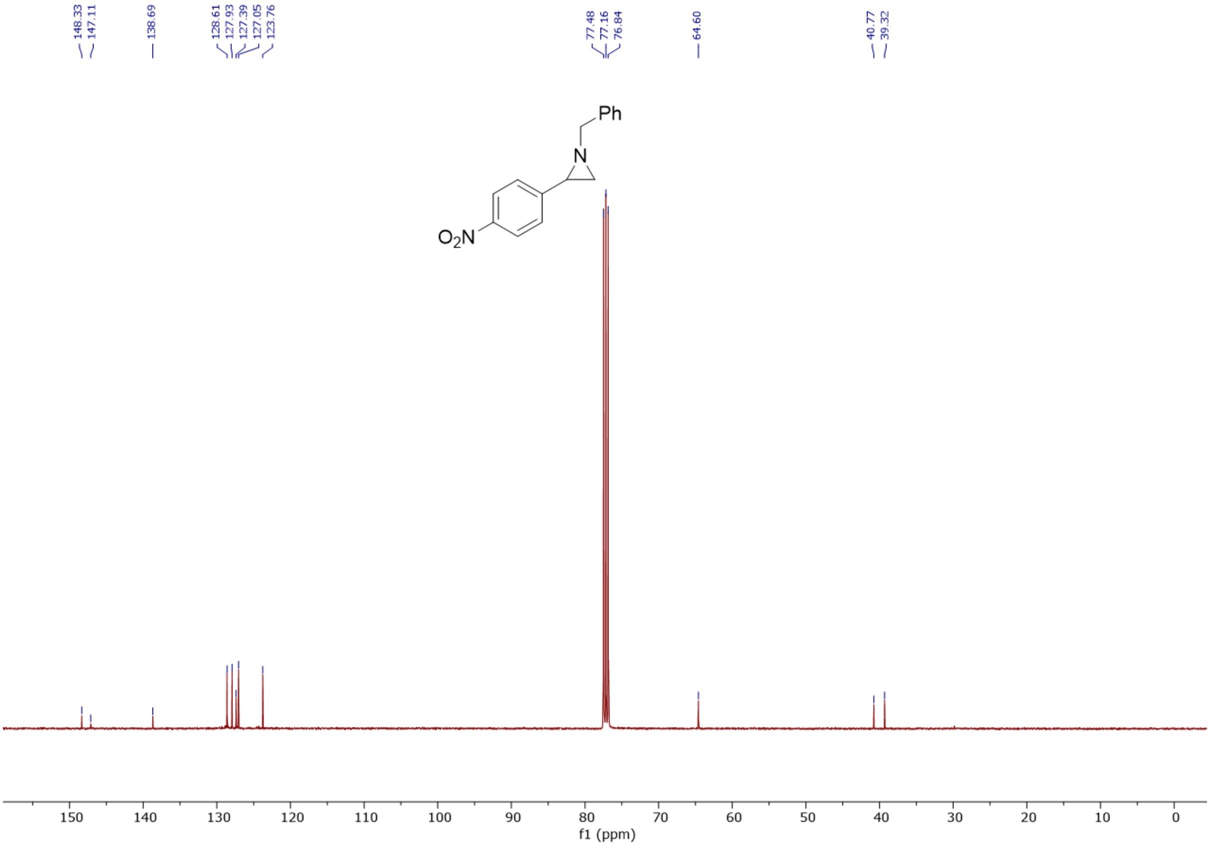
148.33 147.11
138.69
128.61 127.93 127.39 127.05 123.76
77.48 77.16 76.84
64.60
40.77 39.32
Ph
N
O_2N
150 140 130 120 110 100 90 80 70 60 50 40 30 20 10 0
f1 (ppm)

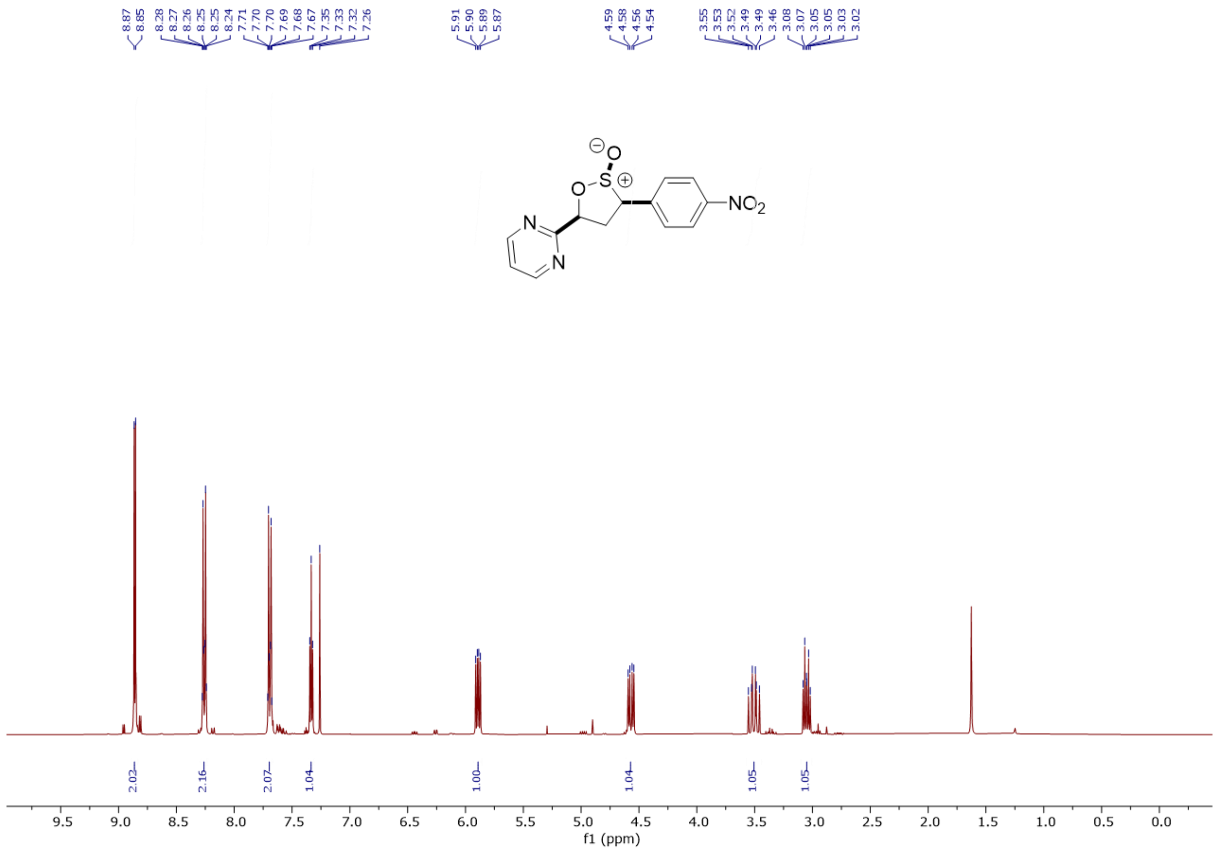

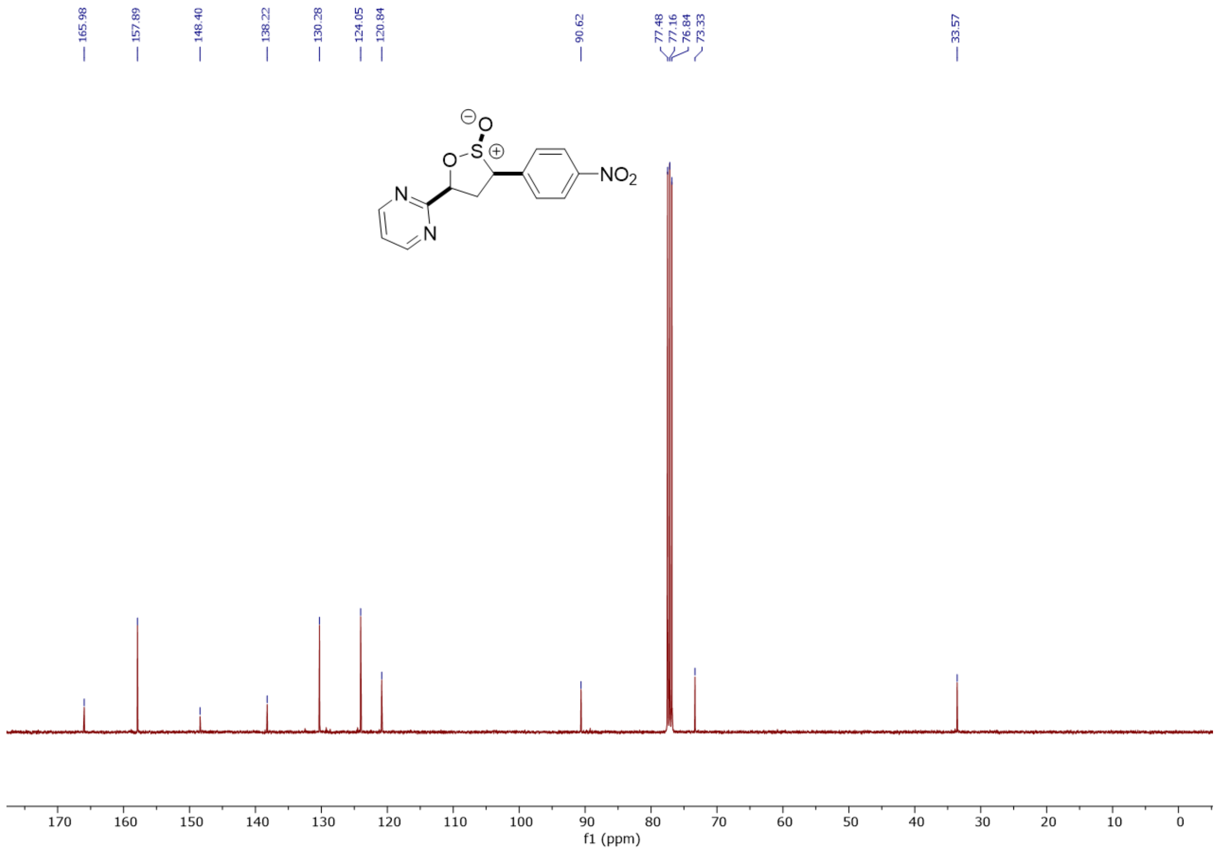

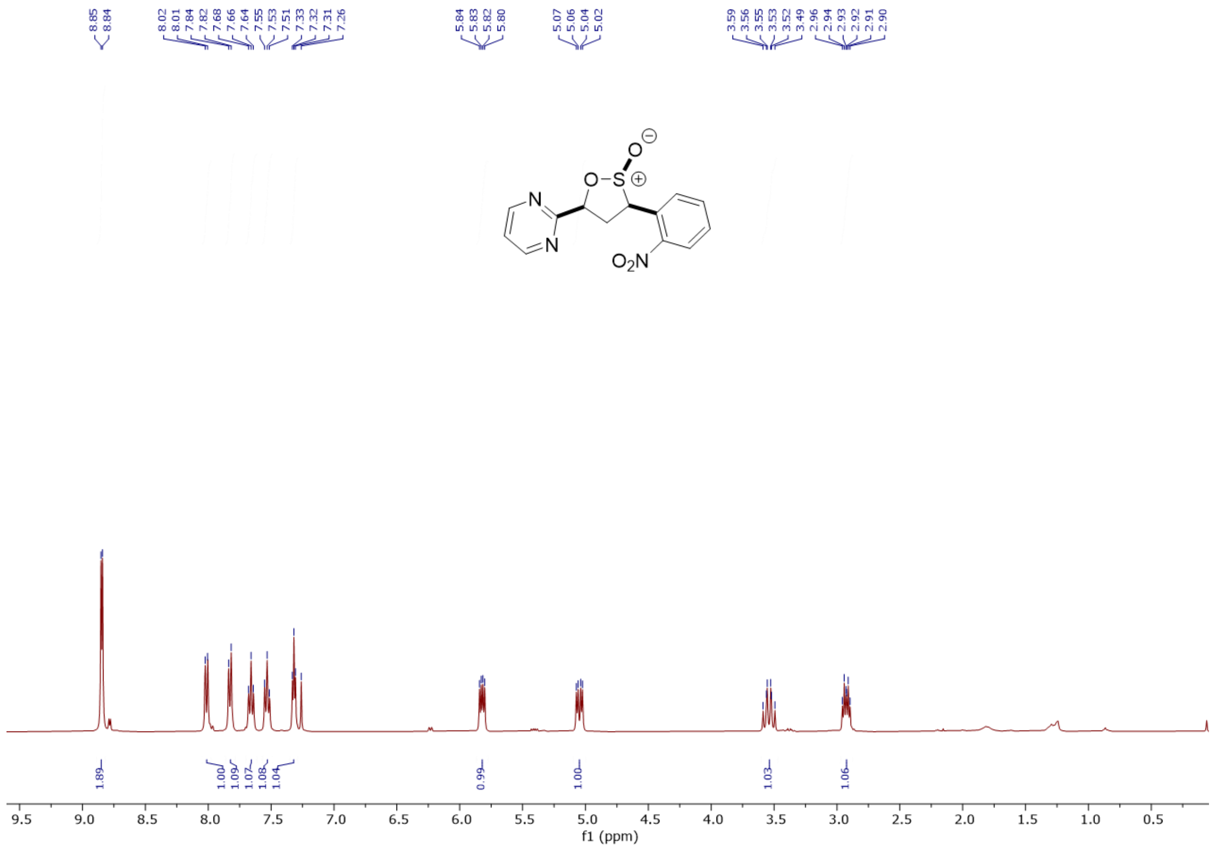
8.85
8.84
8.02
8.01
7.84
7.82
7.68
7.66
7.64
7.55
7.53
7.51
7.33
7.32
7.31
7.26
5.84
5.83
5.82
5.80
5.07
5.06
5.04
5.02
3.59
3.56
3.55
3.53
3.52
3.49
2.96
2.94
2.93
2.92
2.91
2.90
N
N
O
S
O
O_2N
1.89
1.00
1.09
1.07
1.08
1.04
0.99
1.00
1.03
1.06
f1 (ppm)

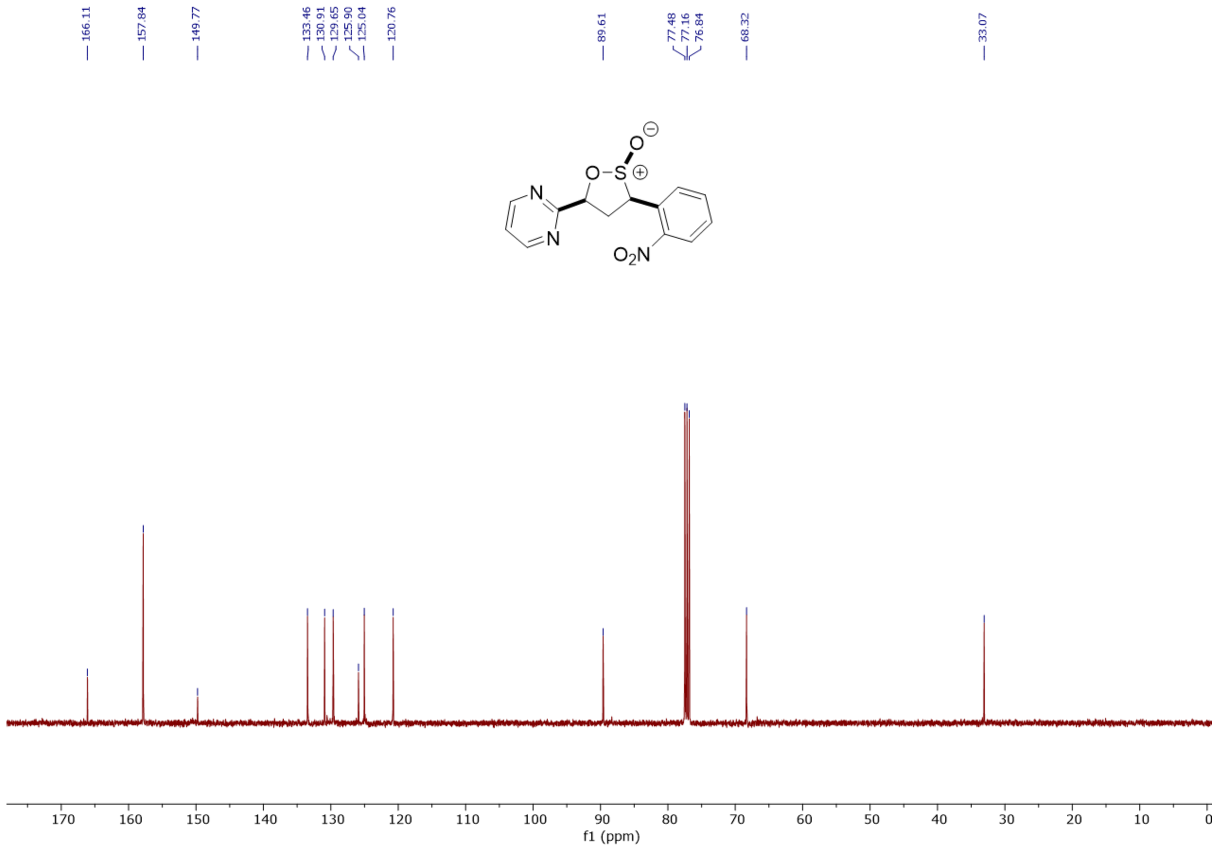
166.11
157.84
149.77
133.46
130.91
129.65
125.90
125.04
120.76
89.61
77.48
77.16
76.84
68.32
33.07
N
N
O
S
O
O_2N
f1 (ppm)

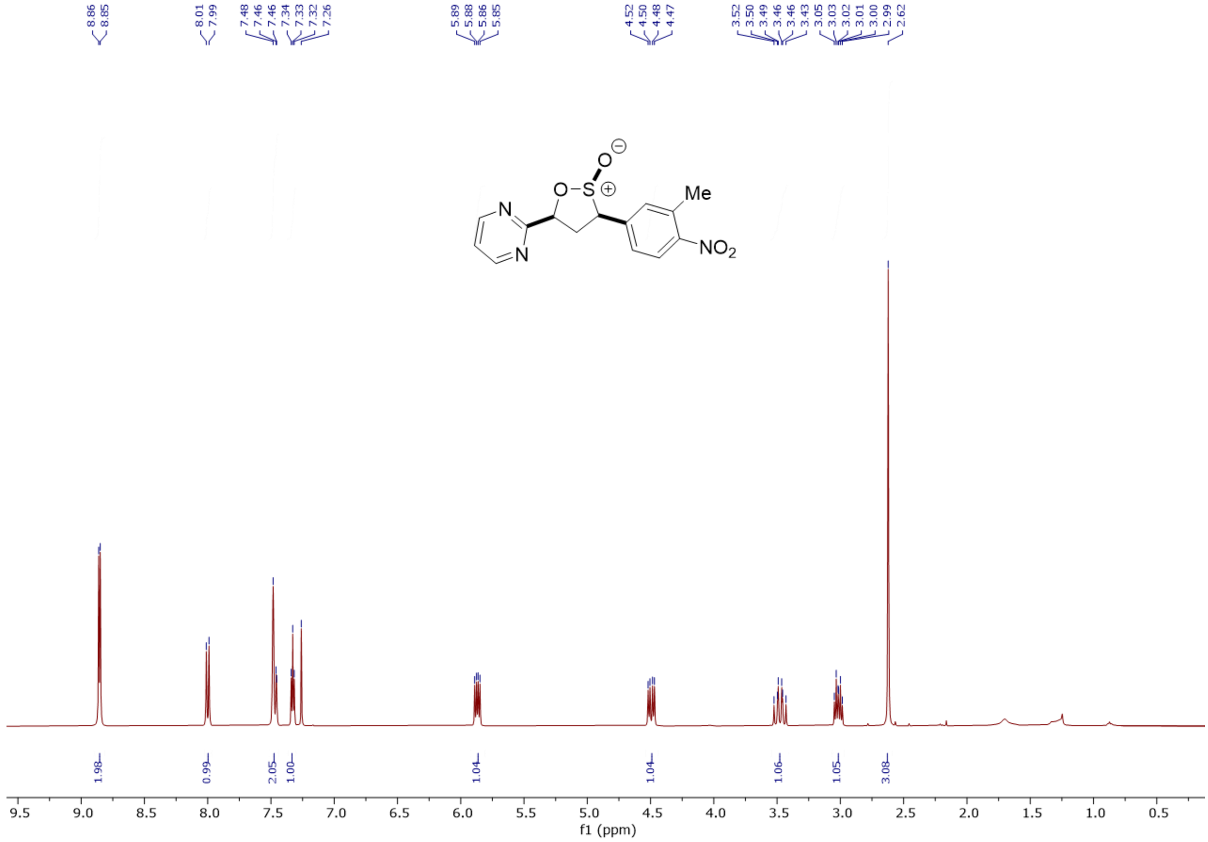
8.86
8.85
8.01
7.99
7.48
7.46
7.46
7.34
7.33
7.32
7.26
5.89
5.88
5.86
5.85
4.52
4.50
4.48
4.47
3.52
3.50
3.49
3.46
3.46
3.43
3.05
3.03
3.02
3.01
3.00
2.99
2.62
O
S
N
N
Me
NO2
1.98
0.99
2.05
1.00
1.04
1.04
1.06
1.05
3.08
f1 (ppm)

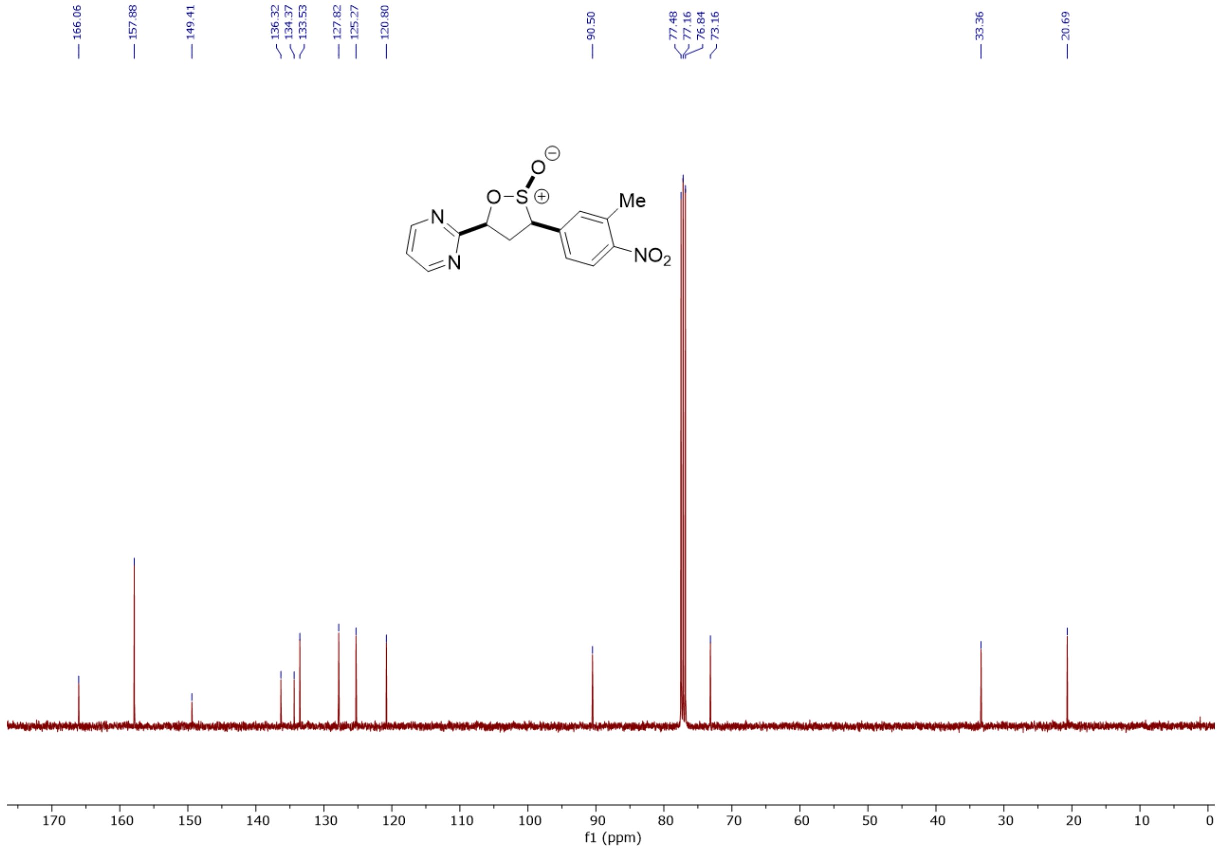
166.06
157.88
149.41
136.32
134.37
133.53
127.82
125.27
120.80
90.50
77.48
77.16
76.84
73.16
33.36
20.69
O
S
N
N
Me
NO2
f1 (ppm)

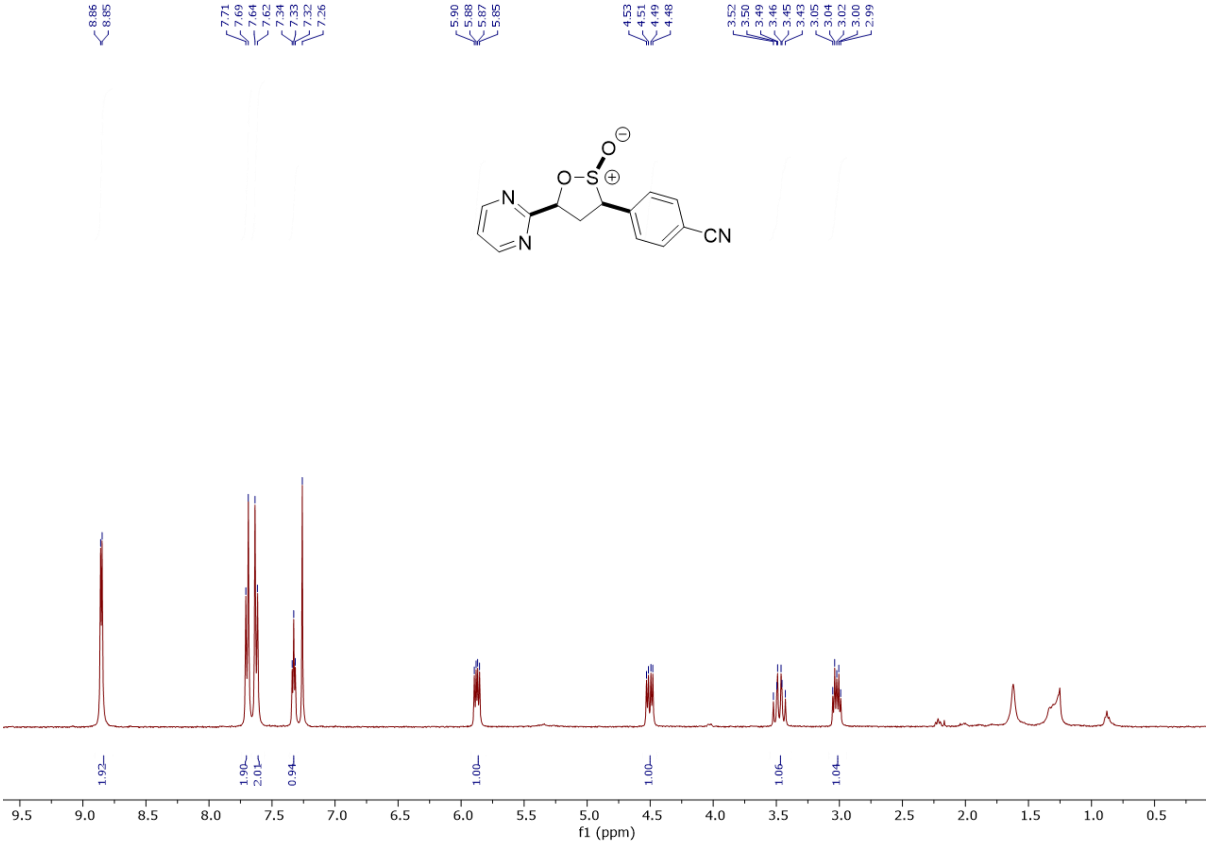
8.86
8.85
7.71
7.69
7.64
7.62
7.34
7.33
7.32
7.26
5.90
5.88
5.87
5.85
4.53
4.51
4.49
4.48
3.52
3.50
3.49
3.46
3.45
3.43
3.05
3.04
3.02
3.00
2.99
CN
1.92
1.90
2.01
0.94
1.00
1.00
1.06
1.04
f1 (ppm)

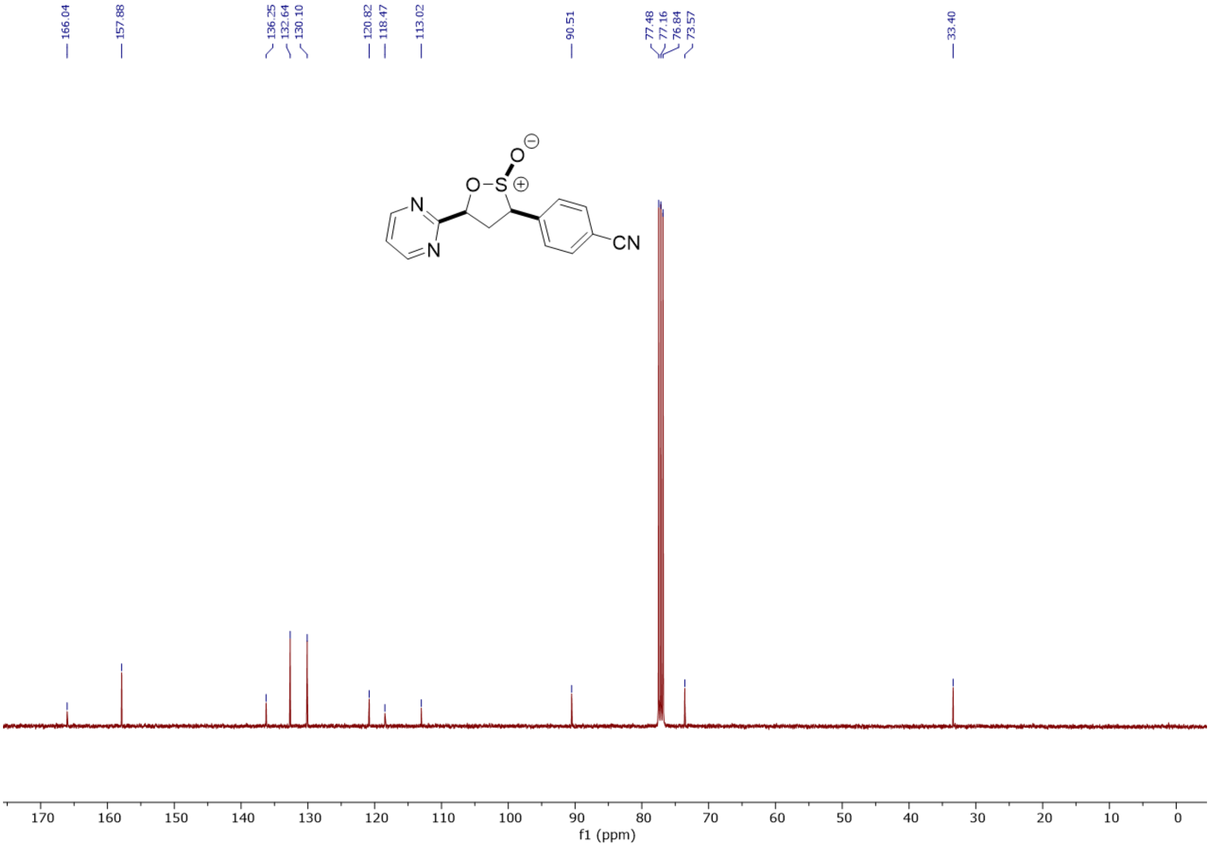
166.04
157.88
136.25
132.64
130.10
120.82
118.47
113.02
90.51
77.48
77.16
76.84
73.57
33.40
CN
f1 (ppm)

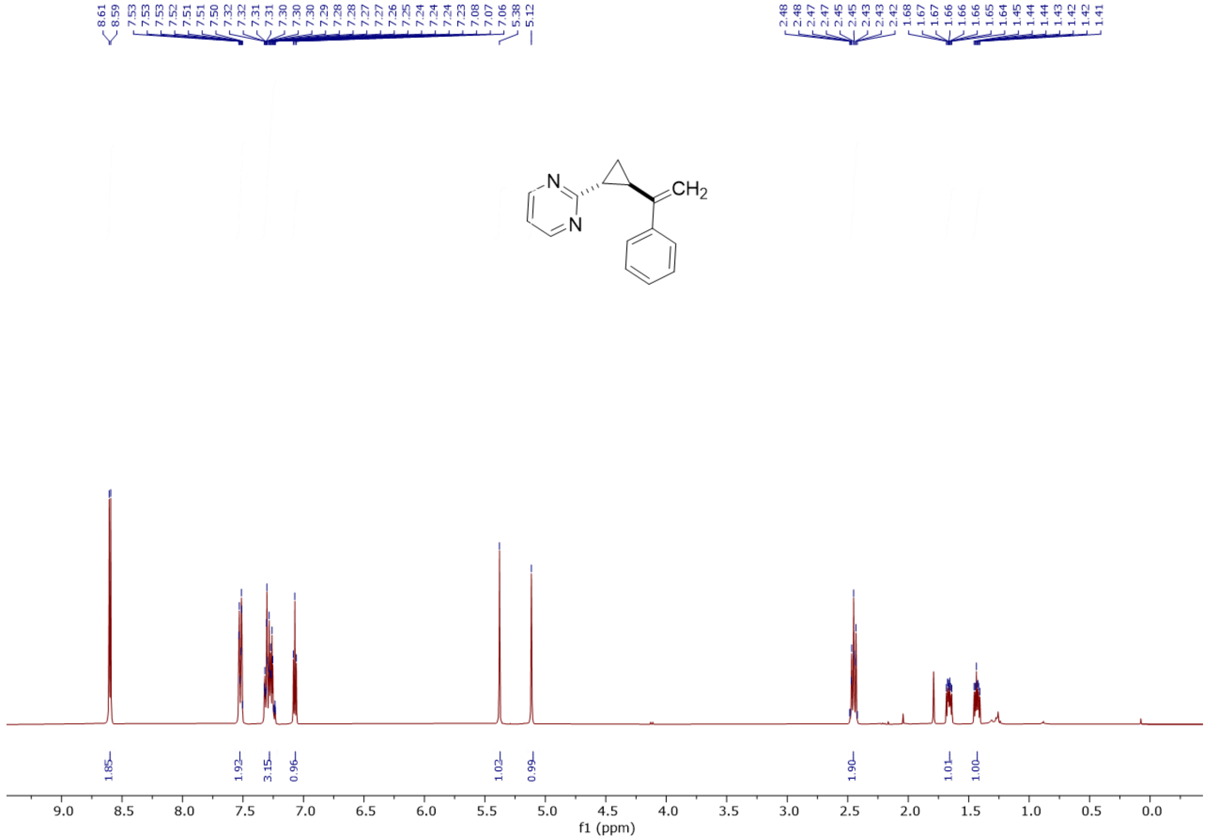
8.61
8.59
7.53
7.53
7.53
7.52
7.51
7.51
7.50
7.32
7.32
7.31
7.31
7.30
7.30
7.30
7.29
7.28
7.28
7.27
7.27
7.26
7.25
7.24
7.24
7.24
7.24
7.23
7.08
7.07
7.06
5.38
5.12
2.48
2.48
2.47
2.47
2.45
2.45
2.43
2.43
2.42
1.68
1.67
1.67
1.66
1.66
1.66
1.65
1.64
1.45
1.44
1.44
1.43
1.42
1.42
1.41
CH2
N
N
1.85
1.93
3.15
0.96
1.02
0.99
1.90
1.01
1.00
f1 (ppm)

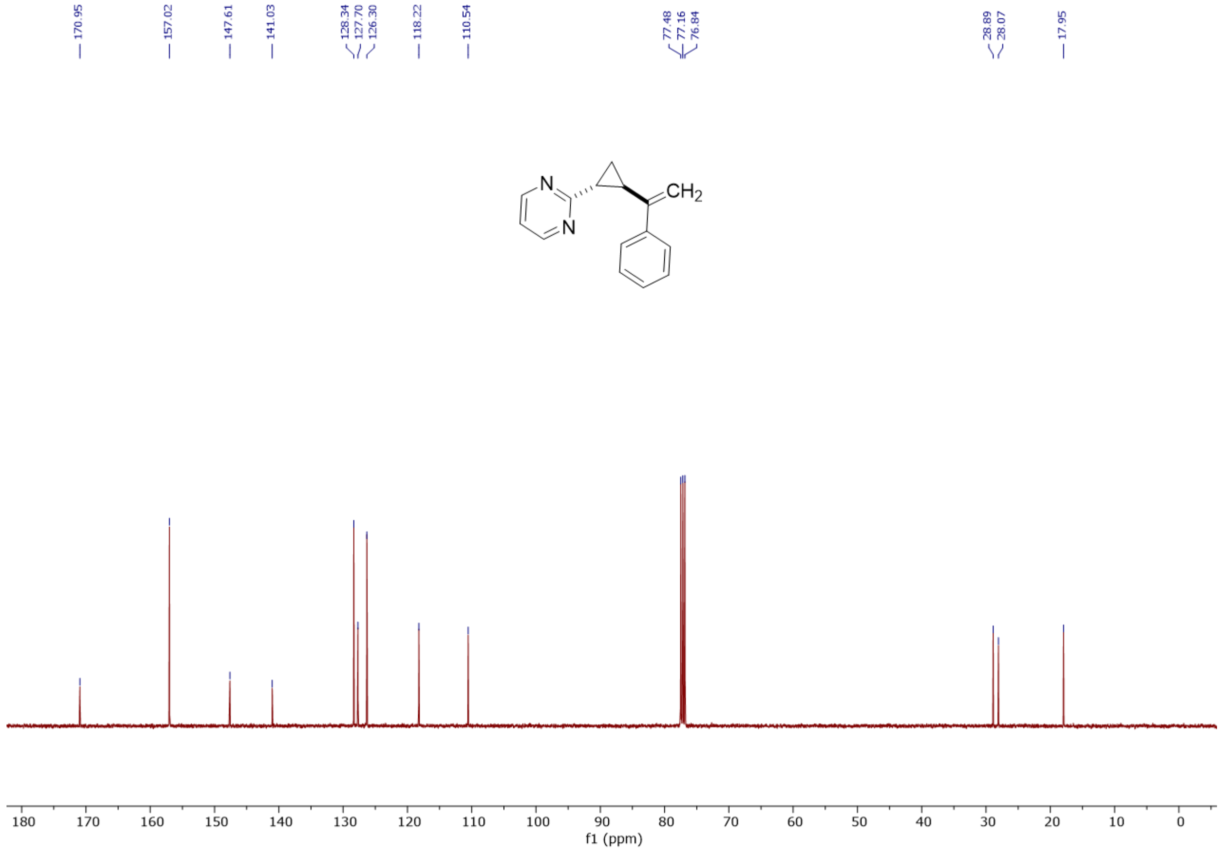
170.95
157.02
147.61
141.03
128.34
127.70
126.30
118.22
110.54
77.48
77.16
76.84
28.89
28.07
17.95
CH2
N
N
f1 (ppm)

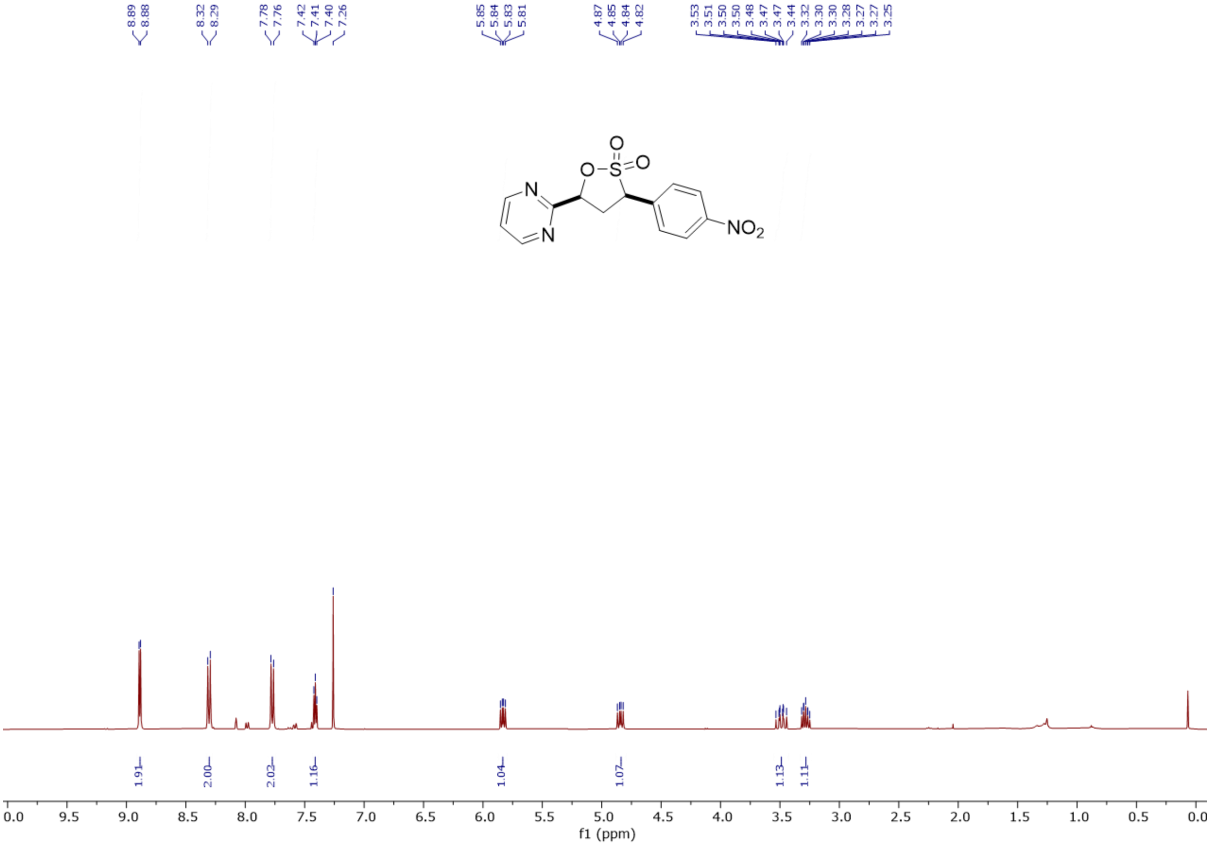
8.89
8.88
8.32
8.29
7.78
7.76
7.42
7.41
7.40
7.26
5.85
5.84
5.83
5.81
4.87
4.85
4.84
4.82
3.53
3.51
3.50
3.50
3.48
3.47
3.47
3.44
3.32
3.30
3.30
3.28
3.27
3.27
3.25
N
N
O
S
O
O
NO2
1.91
2.00
2.03
1.16
1.04
1.07
1.13
1.11
f1 (ppm)

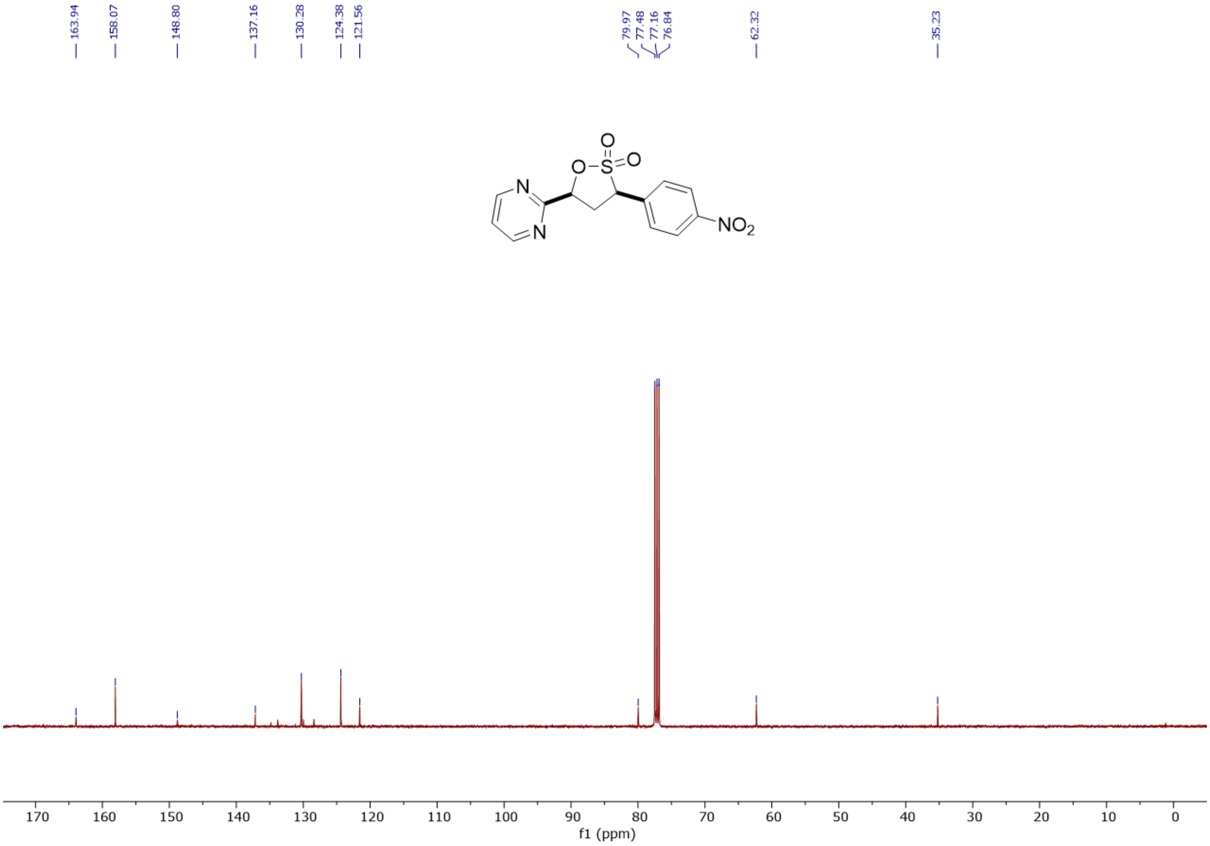
163.94
158.07
148.80
137.16
130.28
124.38
121.56
79.97
77.48
77.16
76.84
62.32
35.23
N
N
O
S
O
O
NO2
f1 (ppm)
